# pi-Turns: types, systematics and the context of their occurrence in protein structures

**DOI:** 10.1186/1472-6807-8-39

**Published:** 2008-09-22

**Authors:** Bhaskar Dasgupta, Pinak Chakrabarti

**Affiliations:** 1 Bioinformatics Centre, Bose Institute, Calcutta, India; 2 Department of Biochemistry, Bose Institute, Calcutta, India

## Abstract

**Background:**

For a proper understanding of protein structure and folding it is important to know if a polypeptide segment adopts a conformation inherent in the sequence or it depends on the context of its flanking secondary structures. Turns of various lengths have been studied and characterized starting from three-residue γ-turn to six-residue π-turn. The Schellman motif occurring at the C-terminal end of α-helices is a classical example of hydrogen bonded π-turn involving residues at (i) and (i+5) positions. Hydrogen bonded and non-hydrogen bonded β- and α-turns have been identified previously; likewise, a systematic characterization of π-turns would provide valuable insight into turn structures.

**Results:**

An analysis of protein structures indicates that at least 20% of π-turns occur independent of the Schellman motif. The two categories of π-turns, designated as π-HB and SCH, have been further classified on the basis of backbone conformation and both have AAAa as the major class. They differ in the residue usage at position (i+1), the former having a large preference for Pro that is absent in the latter. As in the case of shorter length β- and α-turns, π-turns have also been identified not only on the basis of the existence of hydrogen bond, but also using the distance between terminal C^α^-atoms, and this resulted in a comparable number of non-hydrogen-bonded π-turns (π-NHB). The presence of shorter β- and α-turns within all categories of π-turns, the subtle variations in backbone torsion angles along the turn residues, the location of the turns in the context of tertiary structures have been studied.

**Conclusion:**

π-turns have been characterized, first using hydrogen bond and the distance between C^α ^atoms of the terminal residues, and then using backbone torsion angles. While the Schellman motif has a structural role in helix termination, many of the π-HB turns, being located on surface cavities, have functional role and there is also sequence conservation.

## Background

Loops and turns are generally mediated by a stretch of residues with varying backbone conformations. They occupy more than 30% of a globular protein [1] and are often found at the active sites of a protein providing molecular recognition, specific interactions between two molecules and helping to maintain globular shape of the protein [2]. In the context of tertiary structure these are used to connect major secondary structures, like α-helix and β-sheet [3-7].

Turns or shorter loops are more amenable for systematic characterization. Turns of various lengths have been identified, starting from three-residue γ-turn to six-residue π-turn, and the four-residue β-turn has been subjected to rigorous investigation. β-turn was first identified by Venkatachalam [8] and subsequent studies [2,9] include it as one of the major secondary structures in proteins. Though initially turn conformations were thought to be mediated by a hydrogen bond between the CO and NH at two ends of a polypeptide stretch, the presence of such hydrogen bond is not a strict requirement and a limiting distance between two terminal C^α^-atoms has been used to identify the β- and α-turns [9,10]. Exclusively on the basis of hydrogen bond pattern an earlier study has identified six-residue π-turns (largely at the end of α-helices), which were characterized according to the backbone conformation of the residues [11]. A systematic classification of π-turns of both hydrogen-bonded and non-hydrogen-bonded types is necessary in the light of what has been done for shorter α-turns for a proper appraisal of turns in protein structures.

**Figure 1:**
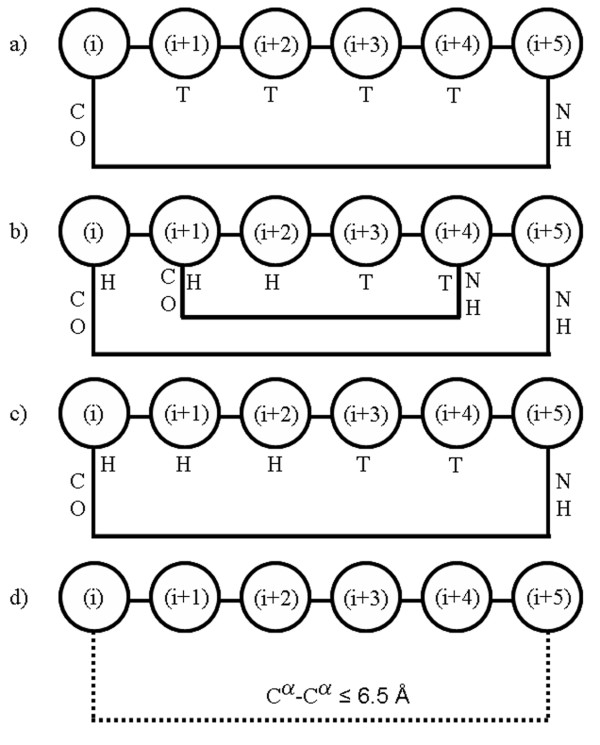
**Schematic representations of different types of π-turns indicating the numbering of the positions.** (i+1) to (i+4) positions are defined as the middle residues. a-c) Hydrogen-bonded turns using main-chain CO and NH groups. The presence of hydrogen bond is shown by solid line connecting (i) and (i+5) residue. For each position the corresponding DSSP notation is shown below. For the π-NHB turns the terminal interaction is shown by a dotted line. a) Isolated π-turn (π-HB), with middle residues in turn (T, by DSSP notation); b) SCH_+β_, where (i) to (i+2) positions are in helical conformation (H, by DSSP notation) and there is an additional hydrogen bond linking (i+1) and (i+4) positions; c) SCH_-β_, which is different from SCH_+β _in that the additional hydrogen bond is missing; d) Non-hydrogen-bonded π-turn (π-NHB) (see text).

By conventional definition π-turns are six-residue long, in which the CO of residue (i) forms a hydrogen bond with the NH of residue (i+5) (Figure 1a). Watson [12] first identified such a turn in the crystal structure of myoglobin. In 1980, Schellman [13] reported that α-helices generally terminate with a particular hydrogen bond pattern at the C-termini, forming (i) ← (i+5) and (i+1) ← (i+4) hydrogen bonds, with the (i+4) residue occurring in the left-handed α-helical region (α_L_). This pattern was subsequently named as Schellman motif [14]. Baker and Hubbard [15], discussing the distortions in α-helices, referred to this conformation as α_C2 _distortion. Subsequently, Milner-White [16] observed that π-turns can occur in either handedness and suggested the name 'paperclips' for the conformation. Further studies [17] reconfirmed that right-handed α-helices generally terminate with a residue in left-handed α-helical conformation (α_L_), which is predominantly Gly and to a lesser extent, Asn. These observations were substantiated by the introduction of simple stereochemical rules for helix termination [14], analysis of helix-stop signals [18,19] and the experimental study of the Schellman motif [20]. All these analyses looked at the π-turn as a helix terminator and the database search was limited to the helices terminated by a residue in α_L _conformation.

Earlier π-turns were identified on the basis of terminal hydrogen bonds [11]. However, for the identification of β-turns a more general approach was taken by considering the cases, in which terminal C^α^-atoms are within 7 Å and the middle residues having restricted φ, ψ angles [9]. This included non-hydrogen-bonded turns also and the occurrence of a large number of turns was useful to detect conformational features of sub-classes of β-turns. Here we have taken a similar approach for identifying π-turns after excluding the ones that also contain parts of other major secondary structural elements (α-helix, 3_10_-helix and β-sheet) in the central positions, (i+1) to (i+4). Previous classification of π-turns [11] was mainly based on the conformation of the (i+4) residue. However, we differentiated π-turns on the basis of backbone torsion angles of all the central residues. We have differentiated π-turns into two major types, hydrogen-bonded (π-HB) and non-hydrogen-bonded (π-NHB) (Figure 1). The identified turns can be considered as isolated turns as they are not part of any other secondary structures. The turns, which occur at the α-helix termini are named as π-turns forming Schellman motif (SCH) (Figure 1b,c) and they form a special case of π-turn, stabilizing terminal residues of α-helix. A typical Schellman motif [13] has two main-chain hydrogen bonds, but if the internal β-turn between (i+1) and (i+4) is absent they are named as SCH_-β_. A true Schellman motif with the β-turn is termed as SCH_+β_.

## Results and Discussion

### Identification of different π-turns

The numbers of occurrences of different types of π-turns are provided in the Table 1 (a detailed list of individual turns appears as Table 5, see Additional file). 597 Hydrogen-bonded π-turns (π-HB) were identified by the program DSSP, where the middle residues did not occur in helical or strand region. These are isolated π-HB turns (Figure 1a). Among 597 turns 350 are in AAAa conformation (see next section, Table 2). 1639 π-turns occur at the C-terminal end of α-helix forming true Schellman motif (SCH_+β_) (Figure 1b). In these π-turns, (i) to (i+2) are in helical conformation with an internal β-turn between (i+1) and (i+4). When we relaxed our criteria of Schellman motif and included those cases where internal β-turn was absent, another 644 turns were obtained (SCH_-β_). The distribution of C^α^-C^α ^distances between the terminal residues in case of π-HB turns is shown in Figure 9 (see Additional file 1), with an average of 5.5 (± 0.6) Å. Consequently the end-to-end distance to detect non-hydrogen-bonded π-turns (π-NHB) was set to 6.5 Å (slightly greater than the one-sigma level). In 3452 cases two or more π-turns were identified, which had overlapping stretches of residues – these were excluded. A total of 2795 non-overlapping turns were obtained with no helical or strand residues in the middle and the terminal residues not connected by hydrogen bond (Figure 1d, Table 1).

**Table 1:** Number of Different types of π-turns

Types of π-turns	Occurrences
Hydrogen-bonded	597
Isolated (π-HB)	
SCH_+β_	1639
SCH_-β_	644
Non-hydrogen-bonded (π-NHB)	2795

**Table 2:** The occurrence of π-turns with different conformational designations and the average backbone torsion angles (°) in the turn region

Class	C^α^-C^α ^distance (Å)	φ_i+1_	ψ_i+1_	φ_i+2_	ψ_i+2_	φ_i+3_	ψ_i+3_	φ_i+4_	ψ_i+4_
π-HB turns									
AAAa (59%)	5.4 (0.4)	-66 (9)	-33 (14)	-71 (13)	-36 (14)	-97 (15)	-6 (12)	65 (16)	38 (17)
PgAA (9%)	6.3 (0.7)	-62 (7)	130 (7)	85 (13)	-8 (17)	-113 (15)	-64 (17)	-101 (19)	-18 (19)
AAAA (6%)	5.8 (1.0)	-63 (9)	-24 (15)	-87 (15)	-15 (23)	-115 (18)	-71 (37)	-99 (26)	-18 (23)
Schellman motif π-turns (SCH_+β_)									
AAAa (98%)	5.3 (0.4)	-65 (5)	-42 (6)	-63 (6)	-30 (8)	-90 (11)	4 (10)	76 (19)	24 (17)
π-NHB turns									
AEAA (11%)	5.8 (0.5)	-85 (19)	-14 (19)	-101 (34)	150 (22)	-62 (11)	-25 (13)	-89 (16)	-3 (17)
EAAa (10%)	5.3 (0.3)	-89 (15)	179 (10)	-64 (9)	-20 (12)	-92 (12)	4 (10)	84 (13)	11 (11)
EAAA (8%)	5.8 (0.5)	-94 (34)	150 (24)	-58 (9)	-30 (12)	-80 (15)	-24 (18)	-110 (21)	-13 (21)
EAAE (6%)	4.8 (0.7)	-120 (28)	177 (19)	-64 (11)	-28 (14)	-105 (19)	-16 (23)	-146 (22)	144 (28)
AAaA (6%)	5.7 (0.4)	-67 (19)	-26 (17)	-92 (19)	-3 (13)	74 (15)	14 (18)	-96 (20)	-17 (19)
AEEa (6%)	5.8 (0.5)	-89 (19)	-12 (17)	-101 (31)	144 (30)	-57 (8)	134 (10)	81 (16)	2 (20)

**Figure 2:**
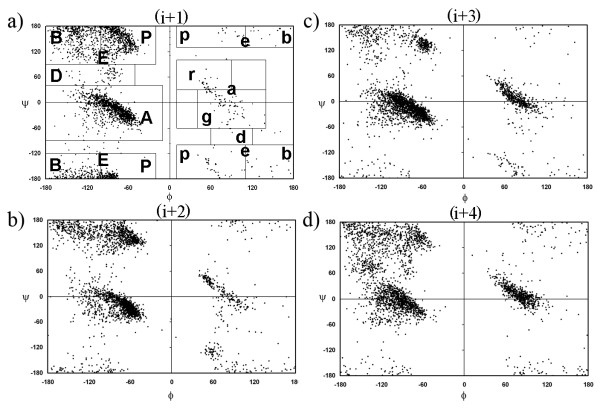
**The distribution of φ, ψ angles for the a) (i+1), b) (i+2), c) (i+3) and d) (i+4) residues in π-HB and π-NHB turns taken together.** The division of the plot into rectangular regions to enclose clusters of points and their labels are shown against (a) (but omitted from the other subfigures for clarity).

The standard deviations are in the parentheses. The classes are defined using the conformational designations shown in Figure 2a.

### Classification of π-HB and π-NHB turns from the distribution of φ, ψ angles and their conformational features

The distribution of φ, ψ angles for the middle four residues of isolated π-turns is shown in Figure 2. The φ, ψ was divided into different rectangular regions, like what was previously done for α-turns [10]. This in general followed the work of Effimov [21] and Rooman et al [22]. It is to be mentioned that region 'A' in the Ramachandran plot (right-handed α-helical region) was not further divided into smaller sections and similarly for the (i+4) residue 'a' region (left-handed α-helical region) was not sub-divided, as the distribution, shown in Figure 2, did not support such divisions. Region 'E' (right-handed, extended region) or 'e' (left-handed, extended region) was divided further into 'B' and 'P' or 'b' and 'p' respectively, for all the residues in π-HB turn. We noted the different combinations of conformational regions of the participating residues. Four-letter codes defining their occurrence in regions of φ, ψ plot were used to denote different π-turns. This resulted in 62 types of isolated π-HB turns, but only 3 conformational types have occurrences greater than 5%. These are AAAa, PgAA and AAAA (Table 2), with AAAa being the major class (59%). When terminal C^α^-C^α ^distances were calculated, PgAA type showed the largest distance (6.3 Å) and AAAa seemed to be the most compact (5.4 Å). If we consider the conformation of the (i+4) position only, it is observed that in 412 cases the residue occur in the left-handed α-helical region (a), while 145 occur in the right-handed α-helical region (A). These numbers are in agreement with the previous study (367 and 111, respectively) [11].

When π-turns occurring at the ends of α-helices, (which forms true Schellman motif with an internal β-turn between residues (i+1) and (i+4) (SCH_+β _type of turns) [13]) were classified, only 4 types were observed, with AAAa being overwhelmingly the largest (>98% out of 1639). When we relaxed our criteria of Schellman motif by taking both SCH_+β _and SCH_-β _cases, we got a total 2283 π-turns occurring at the C-terminal end of helices, which could be grouped into 13 conformational types. AAAa was the most predominant (2230), followed distantly by AAAA (25). All the AAAA cases are present in the SCH_-β _category, indicating that a π-turn with AAAA conformation at the end of α-helix may hinder the formation of an internal β-turn.

An interesting systematics involving the backbone torsion angles can be illustrated using the AAAa type of π-turns (both π-HB and SCH categories) – for the first three residues in the A conformation there is a progressive shift of φ angle towards a more negative value, the shift being quite pronounced (~26°) between the positions (i+2) and (i+3) (Table 2). Such changes, also observed in α-turns of the type AAA, have been explained by the increase in the O⋯O distance between neighboring carbonyl oxygen atoms and the consequent reduction in the electrostatic repulsion [10] – in the absence of intrahelical hydrogen bonds keeping the carbonyl groups parallely oriented within helices, these are frayed when located outside helices. The variation in φ angle is not restricted to AAAa type only, but is also seen in AAAA, where after a reduction of φ along the first three residues there is an increase at the fourth. Indeed, a decrease in the φ angle and an increase in ψ may be a general feature when the conformation is AA involving any two consecutive residues, as can be seen in the turn types under π-NHB category.

For the π-HB turns our study indicates that AAAa conformation to be most frequent, both for the isolated and SCH categories. This observation is a major refinement over the earlier study [11], where isolated and SCH types were not treated separately. The α-helices generally terminate with a residue at the α_L_-region, which correlates well with the presence of AAAa turn with Schellman motif. Placement of (i+4) residue in left-handed region at the ends of α-helices provides a 'helix stop signal' [18,19], which is necessary to terminate a growing helix. But interestingly, isolated π-turns also frequently contain (i+4) residue in the α_L_-region. With the first three residues of π-turn being accommodated as part of α-helix in a Schellman motif, SCH_+β _(Figure 1b), the AAAa conformation of the turn provides the most efficient way of terminating the helix by also fulfilling the hydrogen-bond forming potentials at two pairs of main-chain atoms. As mentioned above, the AAAA type can also terminate a helix in an insignificant number (25) of cases of SCH_-β_, which would have lesser stability as the internal hydrogen bond can not be formed.

For the classification of π-NHB turns from the φ, ψ angle distribution we did not split regions 'A', 'a', 'E', 'e' into smaller subsets [10], such as 'R' and 'G' (from 'A'), 'r' and 'g' (from 'a'), 'B' and 'P' (from 'E'), 'b' and 'p' (from 'e'). Even with these broad regions we got a large number of turn types, but among these only 6 types populate almost half (48%) of all the turns (Table 2). These types are AEAA, EAAa, EAAA, EAAE, AAaA and AEEa. For all the conformational classes of π-NHB turns a good number starts with 'A' or 'E' conformation (43% and 49%, respectively), whereas only 20% (and 15%) of the cases both start and end with 'A' (and 'E'). Terminal C^α^-C^α ^distance is a measure of non-bonding interaction between the two residues and the values provided in Table 2 indicate that EAAE type has the least (i)-(i+5) C^α^-C^α ^distance.

### Presence of shorter turns as part of π-HB and π-NHB turns

**Figure 3:**
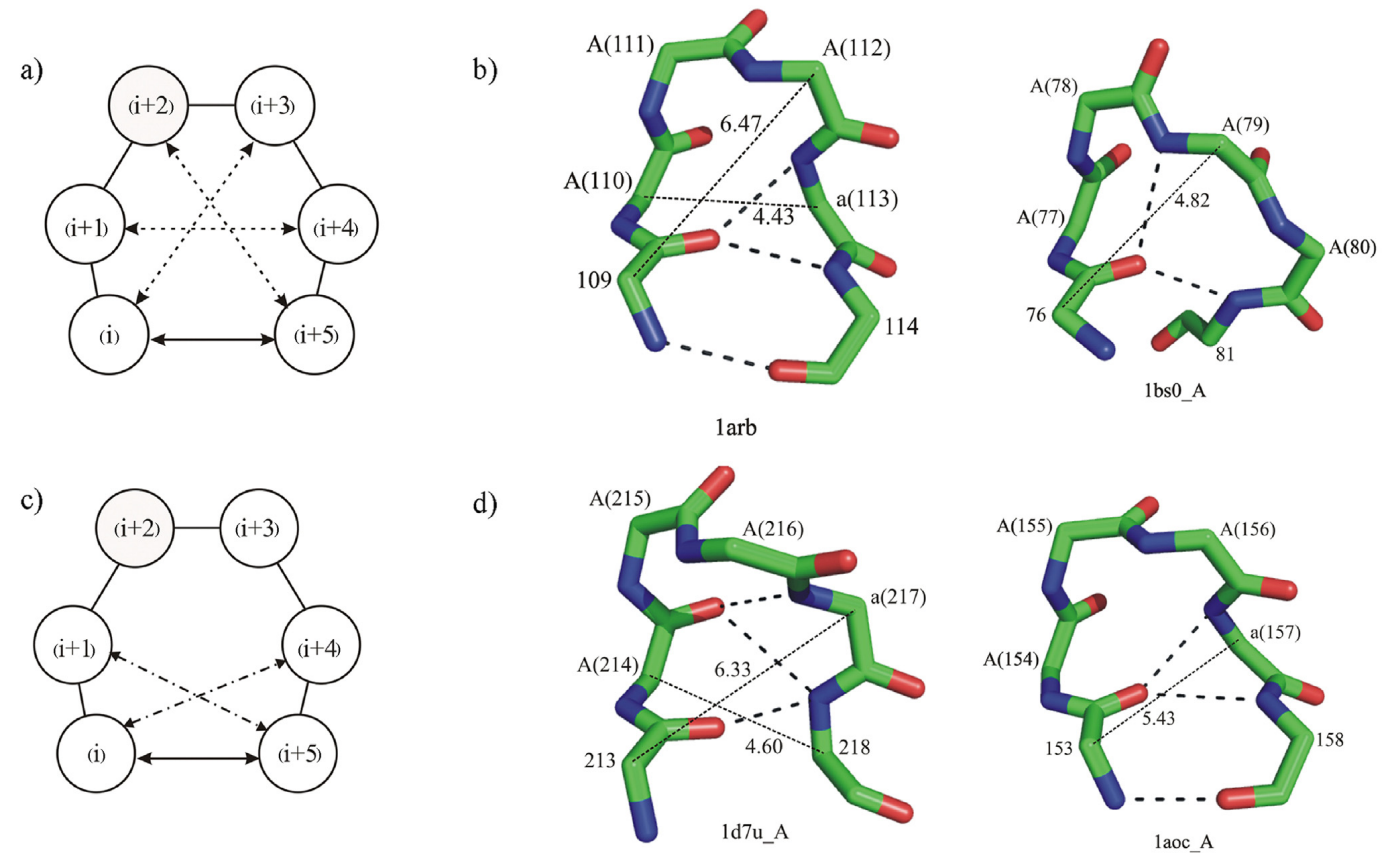
**π-HB turns encompassing multiple a) β- and c) α-turns.** The hydrogen bond in π-turn is indicated by a solid line with two arrow-heads, whereas the dashed lines show the possible β- and α-turns – a maximum of three in (a) and two in (c). b,d) Some representative examples, in which hydrogen bonds are shown as thick dotted lines and C^α^-C^α ^distances are shown as dashed lines with the values of the distances in Å. Residue numbers involving each π-turn are shown in parentheses, preceded by the conformational designation ('A', 'a'). The PDB code of each structure is indicated at the bottom. b) Two examples of π-HB turns are shown with types AAAa (left) and AAAA (right), with two overlapping type-I β-turns involving (i)-(i+3) and (i+1)-(i+4) interactions in the former and only one β-turn involving (i)-(i+3) interaction in the latter. d) Two examples of the occurrence of α-turns within π-HB turn of conformation AAAa. The first one shows two α-turns involving (i)-(i+4) and (i+1)-(i+5) interactions; the next has only (i)-(i+4) interaction.

As π-turns are relatively longer (with four central residues), we looked for the presence of any smaller hydrogen-bonded or non-hydrogen-bonded turns, such as β- and α-, embedded in them. Indeed we find that most of the π-turns are composed of overlapping β-turns. There could be three overlapping β-turns involving (i)-(i+3), (i+1)-(i+4) and (i+2)-(i+5) residues (Figure 3a). We identified the β-turns on the basis of the C^α^-C^α ^distance of the terminal residues and the φ, ψ angles of the middle residues [9]. Only 43 cases could be identified where there is no β-turn within a π-HB turn. The (i+2)-(i+5) residues are usually not involved in β-turns. Various combinations of β-turns occurring within the commonly observed π-turns are reported in Table 3. I-I-X (where X indicates no characteristic β-turn) is the most frequent π-HB. AAAa π-turns are dominantly composed of I-I-X (77%); AAAA π-turns are mostly of the type I-X-X (64%). Two examples of such π-turns having β-turns within are shown in the Figure 3b. When we consider SCH category of turns we observe that the I-I-X combination of β-turns is the dominant (81%) followed by X-I-X (14%). The I-I-X combination correlates with the occurrence of three consecutive residues in the right-handed α-helical region (A); the combination X-I-X is obvious as a true Schellman motif (SCH_+β_) contains an internal β-turn involving the residues (i+1) to (i+4) (Figure 1). It is notable that the major combination of β-turns is I-I-X, which is common to both π-HB and SCH categories, each having AAAa as the major turn type.

**Table 3:** Statistics on the occurrence of different combinations of β-turns within the common π-turns.

π-turn	Multiple β-turns with their designations^a^
π-HB	
AAAa (350)	I-I-X (270), X-I-X (60), I-X-X (17)
PgAA (52)	II-X-X (40), II-X-I (8), X-X-I (1)
AAAA (33)	I-X-X (21), I-I-X (6), I-I-I (2), X-I-I (1), X-I-X (1)
π-NHB	
AEAA (316)	X-X-I (253), VIII-X-I (45), VIII-X-X (4), X-VIa2-I (1)
EAAa (281)	X-I-X (270)
EAAA (219)	X-I-I (156), X-I-X (44), X-X-I (12)
EAAE (176)	X-I-X (130), X-I-VIII (16), VIa1-X-VIII (1), VIa1-I-X (1)
AAaA (171)	I-X-X (146)
AEEa (167)	X-X-II (131), VIII-X-II (20), X-VIb-II (1)

The number of occurrence is given in parentheses.

^a ^X indicates no characteristic β-turn. The conventional nomenclature of β-turns (*viz*., turn types I, II, VI, VIII, etc.) has been used. A multiple turn I-I-X indicates that there is a type I turn involving residues (i)-(i+3), another type I involving (i+1)-(i+4), and the turn type involving (i+2)-(i+5) does not conform to any β-turn type.

Considering π-NHB turns 18% are found to be devoid of any β-turn. In a large number of cases only one type-I β-turn could be identified, with combinations of β-turns designated as X-I-X, I-X-X and X-X-I (Table 3), the first of which occurs with the highest number (593). Types VIII, II, VIa1, VIa2 and VIb β-turns are also observed to different extent. A type VIII β-turn contains two consecutive residues with conformations AE. Similarly type II β-turn appears with Ea conformation of two consecutive residues. Altogether in 55 cases of π-NHB turns type VIa turns are observed and for these the middle residues occur in EA or ED conformation. Likewise in 29 cases of π-NHB turns type VIb turns are observed and they have EE conformation for the middle residues.

Results in Table 3 indicate that two overlapping type-I β-turns (connecting (i) to (i+4) and (i+1) to (i+5)) in π-HB turns occur in most of the cases. In type-I β-turns the middle residues occur in the 'A' region, which could be accommodated in AAAa type of π-turns. This observation also holds good for π-NHB turns. Interestingly, AAAA type of turns is mostly devoid of internal consecutive β-turns and 64% of them can be represented as I-X-X. This implies that AAAa, but not AAAA is amenable to having an additional short-range interaction. For the π-HB turns the less frequent occurrence of AAAA relative to AAAa may be explained on the basis of greater existence of an embedded β-turn in the latter. Type-VIII β-turns are rather frequent in π-NHB turns and this can be correlated to the change of conformation of the middle β-turn forming residues from 'A' to 'E'.

So far we have considered if a π-turn can have one or more β-turn within it. Similarly, one can see if multiple α-turns can form a π-turn. α-turns were found out by checking if distances between C^α^-atoms three residues apart was <6.5 Å [10]. From Figure 3c it is evident that at most two α-turns (spanning residues (i) to (i+4) and (i+1) to (i+5)) can exist within a π-turn. Results indicate that within almost all π-HB turns there is at least one α-turn. Among the 597 π-HB turns 577 contain embedded α-turns. 198 examples have both (i)-(i+4) and (i+1)-(i+5) α-turns. In 324 cases only (i)-(i+4) α-turn is observed and 55 cases have only (i+1)-(i+5) α-turn. Thus the possibility of having (i)-(i+4) interaction is significantly higher than having a (i+1)-(i+5) interaction.

We also looked for conformations of such α-turns within a π-turn. Among the 324 π-turns with only (i)-(i+4) hydrogen-bonded α-turn, the conformation of π-turn is mostly AAAa (258 cases); thus the α-turn is in AAA conformation. Of the 55 cases of π-turns with only (i+1)-(i+5) interaction, 24 are in AAAa conformation, such that the α-turns are in AAa conformation. For the cases with two overlapping α-turns, 57 have the designation AAAa, 47 PgAA and 30 AAAA. The α-turn conformations have the combinations of AAA and AAa in the first; PgA and gAA in the second; and two consecutive AAA turns in the last type of π-turns. Two examples, one with two overlapping α-turns and another containing only a single α-turn are shown in Figure 3d.

The higher number of embedded α-turns with (i)-(i+4) interaction rather than the (i+1)-(i+5) interaction owes to the fact that major conformation of hydrogen-bonded α-turn is AAA that matches with the first three positions of AAAa type of π-HB turns.

### Propensities of residues to be in different π-turns

**Figure 4:**
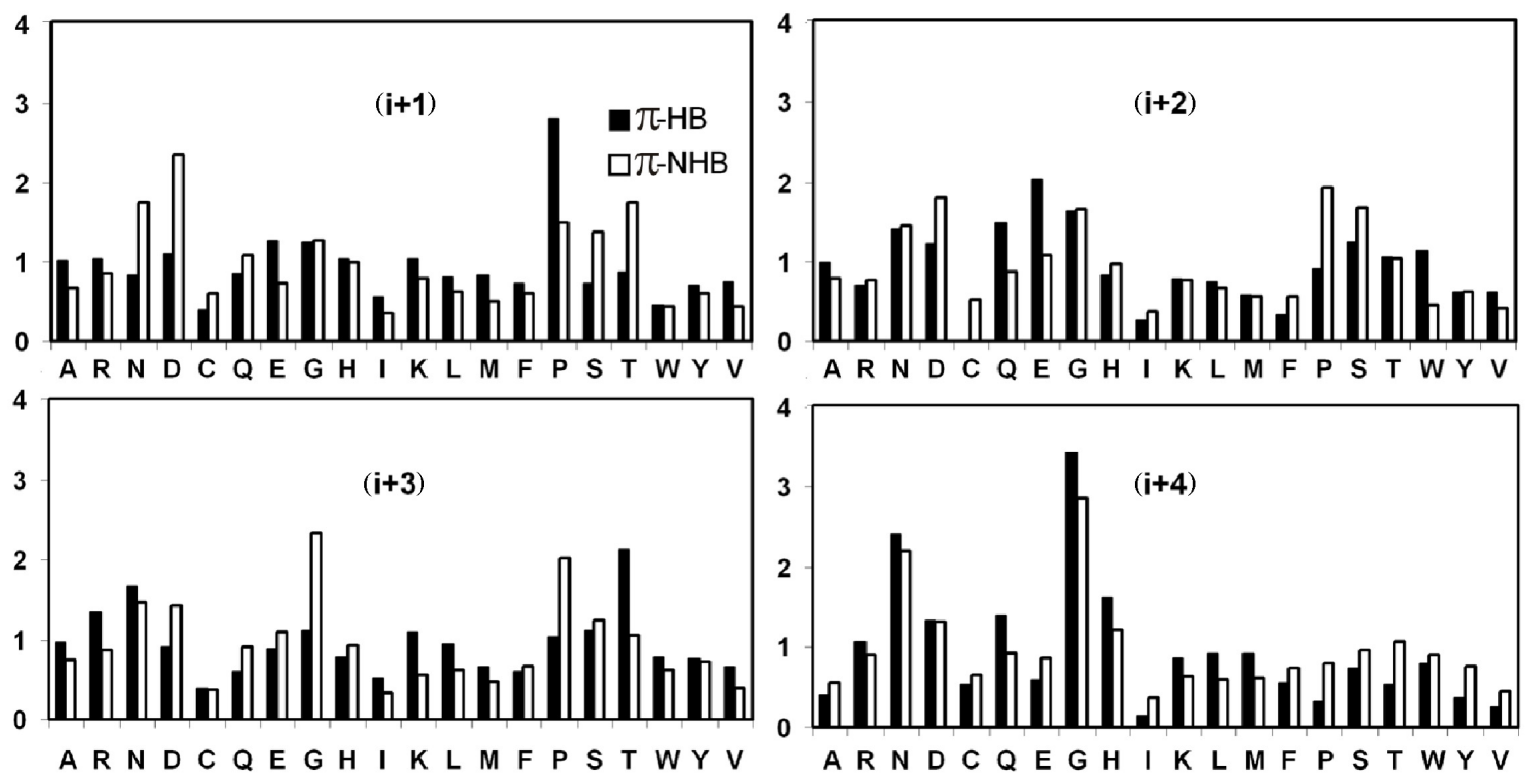
Histogram of residue propensities at different positions of π-HB and π-NHB turns.

Propensity values were calculated from the observed and expected number of occurrences of each residue from (i-1) to (i+6) positions in π-HB and π-NHB turns and the values for the middle positions (from (i+1) to (i+4)) are presented in Figure 4; the results are statistically significant as can be seen from the z-values provided in Figure 10 (see Additional file). At (i+1) position Pro has a high value in both types of turns, and additionally Asn, Asp, Ser and Thr are preferred in π-NHB. The high propensity of Pro, along with that of Gly, is also observed at (i+3) for π-NHB turns. Gly has a high preference for the (i+4) position in both types of turns – this is due to the conformation at this position being in the 'a' region [17]. Of all the residues occurring at (i+4) position of π-NHB Gly has the highest occurrence (22%).

The position i is markedly occupied by Asn, Asp, Cys and Ser for π-HB, as well as π-NHB turns and Pro is conspicuous (1.70) at this position for π-NHB turns (data not shown). The presence of Asn and Asp can be attributed to the fact that these can mimic the backbone chain effectively and provide additional hydrogen bonding using side-chain polar groups to the middle residues of π-turn [23-25]. The (i+5) position is devoid of Pro in π-HB (a Pro at this position can not act as a hydrogen-bond donor, a prerequisite), though π-NHB contains it sufficiently. However, Pro can act as hydrogen-bond acceptor and thus it can be present at the (i) position in π-HB. We also looked for the residue propensities around π-turn and found that aromatic residues like Trp, Tyr and Phe are important at position (i-1) for both types of turns. For the π-NHB, Lys also has a high propensity at this position (data not shown). At (i+6), aromatic residues (Trp, Tyr and Phe) and Lys, Ile, Val and Pro are abundant for both the types. The presence of aromatic residues at the (i+6) position and Pro at (i+5) for π-NHB turns can be explained by the formation of π-stacking interaction involving side-chains of aromatic residues and Pro [26,27].

### Residue composition of π-turns and their resemblance with other secondary structures

**Figure 5:**
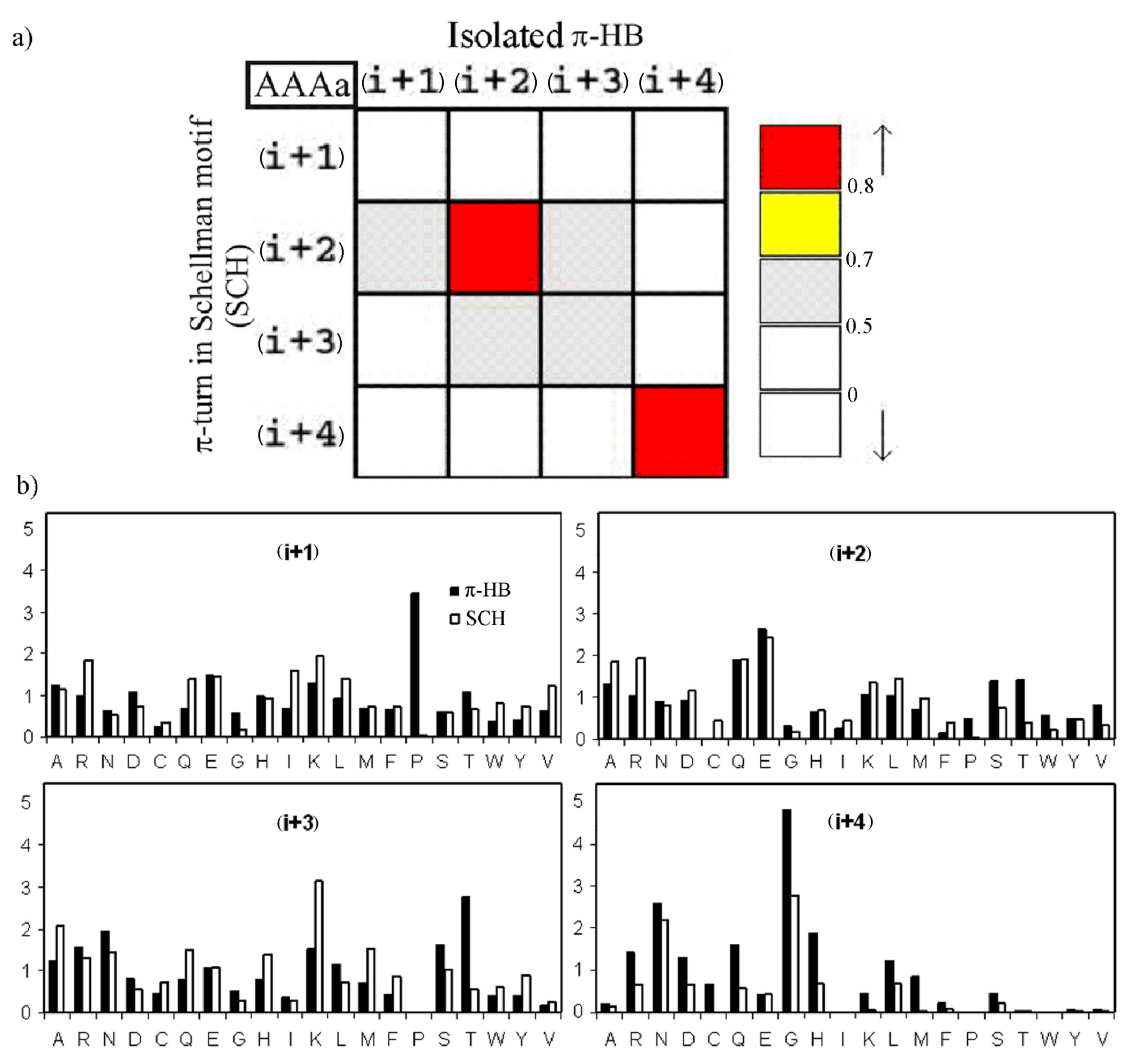
**a) Comparison of residue composition in π-turns of the type AAAa between isolated (π-HB) and those with the Schellman motif (SCH).** Correlation coefficient matrix is color-coded and the range of values associated to each color is shown. b) Position-specific propensity profiles of residues occurring with the conformation AAAa, where they occur in isolation (π-HB) and at the end of helices (SCH).

As type AAAa conformation is common to both π-HB and π-turns occurring at the end of helices (SCH), we looked for any similarity in residue composition between these two types of turns, by calculating the correlation coefficients of percentage composition at different positions. The residue compositions match well except at the position (i+1). Figure 5a shows that (i+2) and (i+4) positions are occupied by residues of similar types, (correlation coefficients are 0.86 and 0.98, respectively). The correlation is relatively less at position (i+3) (0.57) and is only 0.34 at (i+1). We analyzed the position-wise propensity-profile of residues occurring in both types of turns, i.e. π-HB turns and Schellaman motif with AAAa conformation. Figure 5b shows the histogram of propensity values calculated for each of the middle positions (the corresponding z-values are provided in Figure 11, see Additional file). For the (i) position, Asn, Asp, Cys, His and Ser are prominent in the former type and Ala, Cys, Lys, Met are found chiefly for the latter type (data not shown). Pro is particularly favored at the (i+1) position for the π-HB turns; in contrast, Pro is absent at this position in Schellman motif. After (i+1) position Pro marks its presence again at (i+6) (data not shown). Higher correlations in residue compositions at (i+2) and (i+4) positions (Figure 5a) are due to the occurrence of similar residues and similarly the match between the histograms at positions (i+1) is poor (Figure 5b) leading to a poorer correlation.

**Figure 6:**
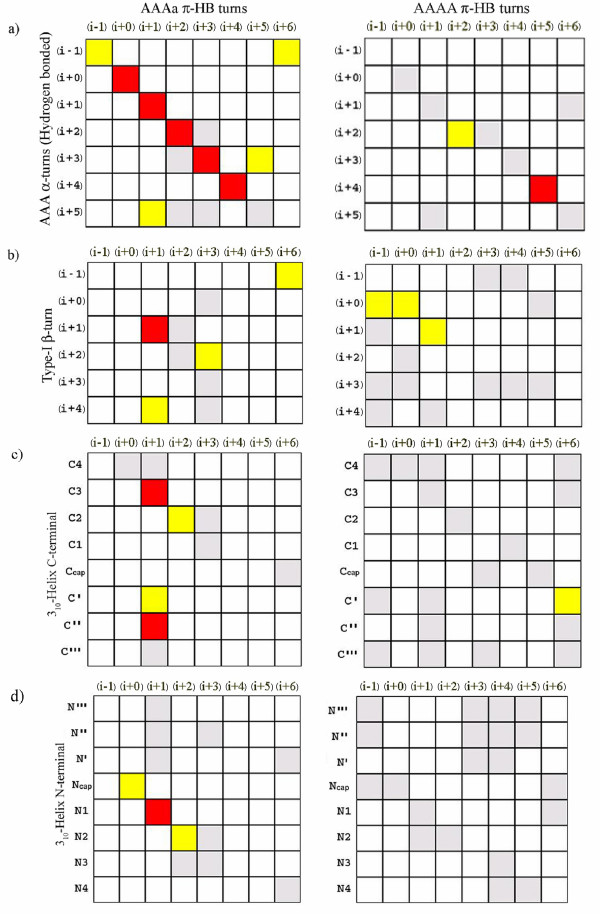
**Correlation coefficient of the percentage compositions of amino acids at different positions of two types of π-HB turns and those in a) α-turn of the type AAA, b) type-I β-turn, c,d) C- and N-termini of 3_10_-helix.** In the left AAAa type of π-HB turns are used, and in the right are AAAA π-HB turns. β-Turns include both hydrogen-bonded and non-hydrogen-bonded categories, while only hydrogen-bonded α-turns are taken. The color code is explained in the Figure 5a.

Next we found out the correlation coefficients at different positions of π-HB turns (the two major types, AAAa and AAAA, considered separately) with other helices and turns. The matrices obtained are represented in Figure 6. When compared with hydrogen-bonded α-turn of the type AAA, a great deal of similarity in residue composition is observed with the AAAa type of π-HB turns, though AAAA π-HB turns are less correlated (only (i+2) and (i+5) positions are correlated) (Figure 6a). The residue usage at (i+1) position of type-I β-turns is found to be similar to both AAAa and AAAA types of π-HB turns (Figure 6b). No particular trend in correlation is seen between π-turns and N- and C-termini of α-helix. The matrix of correlation coefficients between AAAa π-HB and C-terminal of 3_10_-helix is presented in Figure 6c and the matrix for AAAa π-HB and N-terminal of 3_10_-helix is given in Figure 6d. 3_10_-helices are mostly three-residue long and so Ncap and C4, N1 and C3, N2 and C2 and N3 and C1 represent the same positions [28,29]. Thus the observations at the N- and C-termini of 3_10_-helix may indicate the same effect.

Correlation between positional residue compositions in different secondary structural elements may have implications how one type of secondary structure is converted to another. The high degree of similarity between AAAa π-HB turns and AAA hydrogen-bonded α-turns may indicate that the conversion between them is facile.

### Secondary structures around π-turns

**Figure 7:**
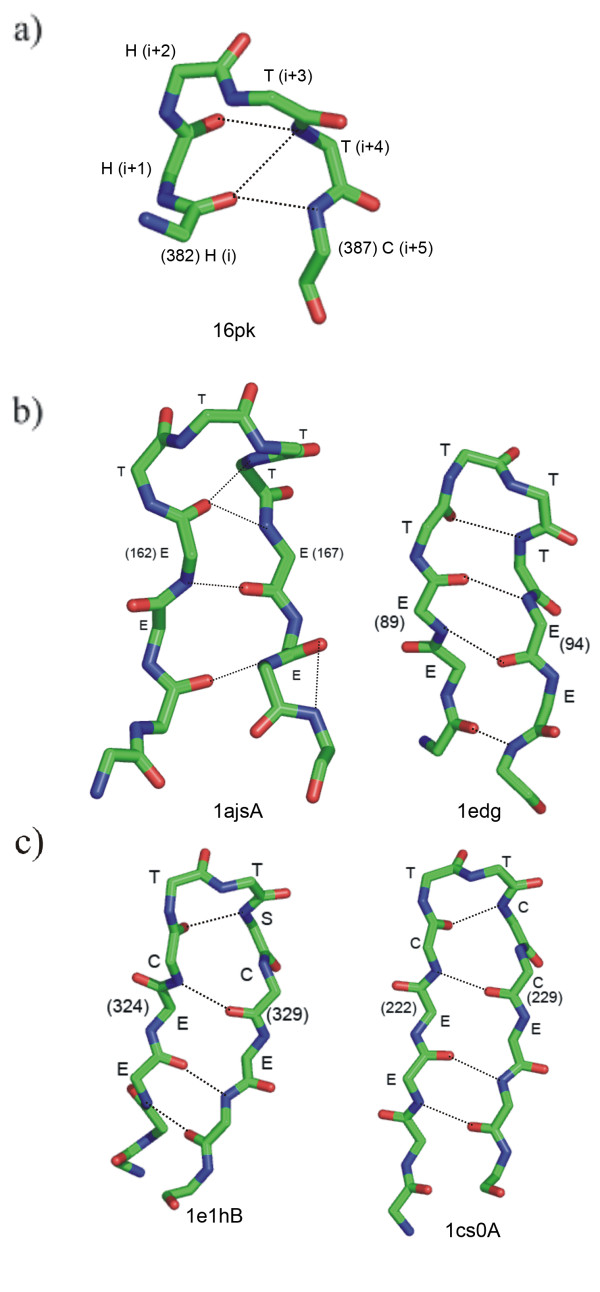
**A few illustrative examples of (a) Schellman motif and (b,c) β-hairpin motifs formed by using some of the π-HB and π-NHB turns of different conformations.** The PDB code of each structure is given in the bottom with chain identity; the starting and end residue numbers are given in parentheses, indicating the location of the π-turn. For each residue in π-turn along with the terminal residues, the secondary structural element is indicated in accordance to DSSP. a) Schellman motif formed at the end of an α-helix. Labels of positions are given in parentheses. Hydrogen bonds are shown as dashed lines (the (i) ← (i+4) hydrogen bond is intrahelical). The residue at (i+4) position is Gly. b) π-HB turns in between β-strands. The left structure is in conformation AAAa and the right pAAa. c) Two examples of π-NHB turns (with EAAa conformation) forming β-hairpin motif.

It has been reported that π-turns of the type π_αL _(bearing (i+4) residue in left-handed α-helical region) are found mostly at the end of α-helices and constitute the Schellman motif [13]. According to our convention the Schellman motif is included in AAAa type of π-turns – an example of the motif with (i) ← (i+5) and another internal (i+1) ← (i+4) hydrogen bonds is shown in Figure 7a. π-turns have also been reported earlier in β-hairpins with four-residue loops [30]. Table 4 indicates that π-HB and π-NHB turns are mostly found in between two β-strands and sometimes they are also engaged in forming hairpin type of structures. Of the total 597 π-HB turns, 306 cases have terminal residues in β-strands with the middle residues mostly in turn conformation. Out of 306 cases, 286 are involved in forming hairpin motif of the type 4:4, according to the nomenclature adopted by Sibanda et al [31]. Considering the conformational classes of π-HB turns we find that 245 out of 350 AAAa turns are in ETTTTE type of secondary structures (where each letter corresponds to the secondary structure designation of DSSP). Out of 245 turns 228 are in hairpin structures. In pAAa type of π-HB turn (not shown in Table 2) ETTTTE type of secondary structure is observed (10 cases out of 24). Two examples are shown with AAAa and pAAa types of π-HB turns in Figure 7b.

**Table 4:** Most common secondary structures involving different types of π-turns and their immediate neighbors^a^

	Secondary structures at positions^b^									
			
Turn	i-1	i	i+1	i+2	i+3	i+4	i+5	i+6	Number	Percentage^c^
π-HB										
AAAa	E	E	T	T	T	T	E	E	245	70
PgAA	C	C	B	T	T	T	B	C	8	15
pAAa	E	E	T	T	T	T	E	E	10	42
π-NHB										
AEAA	E	E	S	S	T	T	S	C	14	4
	H	H	T	S	T	T	C	C	13	4
	E	E	S	S	T	T	C	C	12	4
	C	T	T	S	T	T	C	C	12	4
EAAa	E	E	C	T	T	S	C	E	86	31
	E	E	C	T	T	C	C	E	68	24
	C	B	C	T	T	S	C	B	16	6
EAAA	C	C	C	T	T	T	H	H	13	6
EAAE	E	E	C	S	S	C	E	E	73	41
AAaA	E	C	T	T	S	S	E	E	47	28
	E	C	T	T	S	S	C	E	13	8
AEEa	H	H	T	S	T	T	C	C	10	6
	C	T	T	S	T	T	C	C	10	6

^a^The combination of secondary structures with >10 occurrence are shown. There is no occurrence of isolated AAAA and AAAa types of π-HB turns immediately after the α-helix.

^b^According to DSSP notation E: β-strand, T: hydrogen-bonded turn, B: isolated β-bridge, S: bend and we represent non-regular structure by C.

^c^Based on the number observed with the given combination of secondary structures (given in the previous column) relative the total number in the class.

Of all the major π-NHB turns the preference for a particular type of secondary structure is observed in AAaA, EAAE and EAAa turns. The formation of β-strands at the ends of π-turn is observed in a large number of cases and they are also involved in β-hairpin structure. Apart from the terminal residues, the middle residues form different types of structures in π-NHB turns. In a large number of cases these are found in bend (or S, according to DSSP) structure. Two examples of EAAa turns are shown in Figure 7c, one containing the S structure and the other with a non-regular secondary structure at the (i+4) position.

Thus the above observations suggest that the most common structural motif where π-HB and π-NHB turns are found is β-hairpin (Table 4, Figure 7). Earlier we observed that the five-residue α-turn could be the connectors of two anti-parallel β-strands [10]. Likewise, beyond α-turn, π-turns of both hydrogen-bonded and non-hydrogen-bonded categories could be part of β-hairpins longer by one-residue.

### π-Turns in active sites and residue conservation

**Figure 8:**
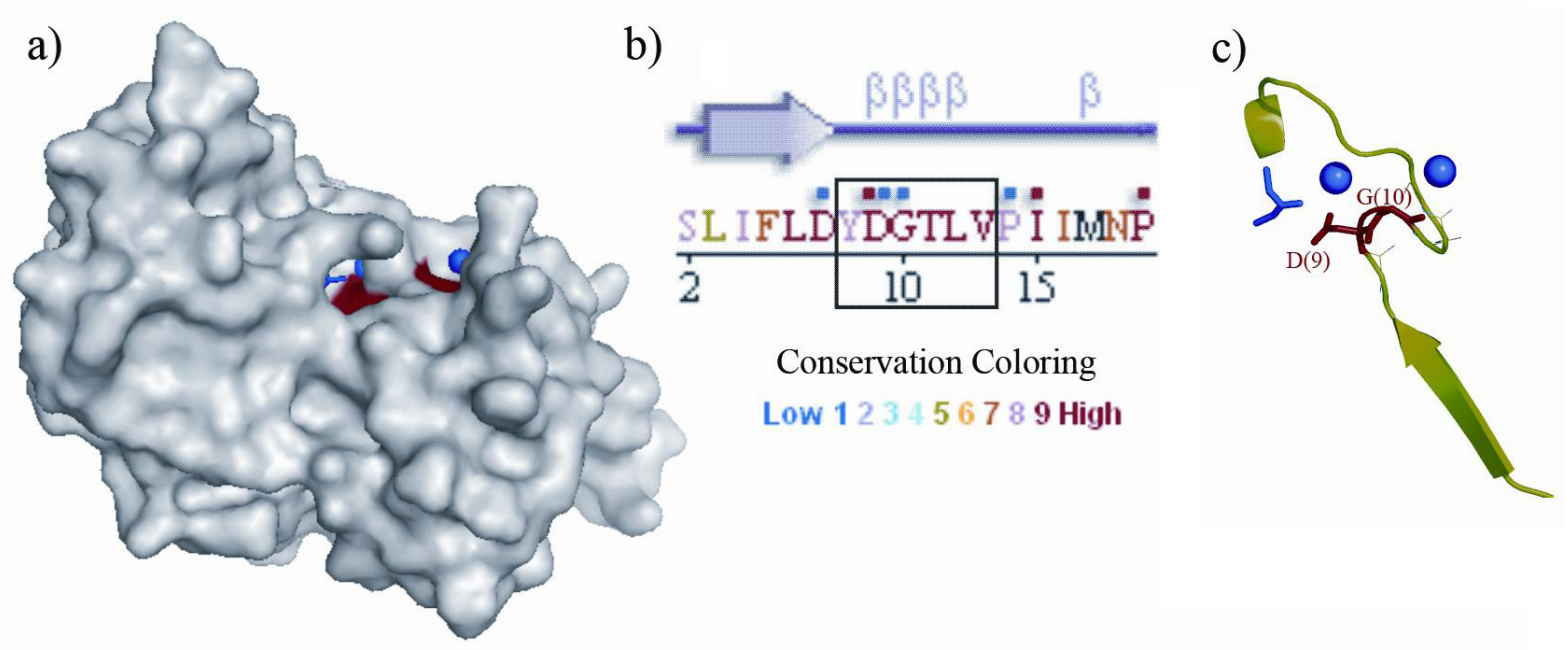
**π-turn with functional importance in the structure with PDB code 1u02A.** The surface representation (with the ligand in blue and active-site residue in red) is shown in (a), the relevant annotation from PDBSUM (with the residues in the π-turn being enclosed in a box) in (b), and a cartoon diagram of the π-turn and its flanking regions (all the atoms are shown in red for the two residues interacting with the ligand/metal, in blue) are displayed in (c). In (b) the coloring of the residues in the sequence is based on conservation (explained in Methods).

An analysis was carried out to ascertain the functional relevance of residues involved in π-turns. As active sites are located on surface pockets we first determined if residues in π-turns are located in such regions of the structure. It was found that close to a third of all turns (37% of π-HB, 35% SCH and 33% π-NHB) have residues that are indeed located in such cavities. As π-HB turns are independent of the Schellman motif that has structural importance in the context of α-helix, and also because they have lesser conformational variability compared to π-NHB, π-HB turns were considered further. Again, a third of π-HB turns in cavities (72 out of 222) are situated in such a way so as to interact with ligand or metal, or harbor residues that are catalytically important or involved in forming disulfide cross-linkage or forms the active site. A detailed list of all such entries is given in Additional file (Table 7) and an example is shown in Figure 8, which indicates the usefulness of π-turn in providing the site of binding of ions (sodium) and ligand. The information on residue conservation is available only for 142 (out of 222) cases, and 53% of them have at least three residues in the turn that are fully conserved (the value increases to 77% if fully and partially conserved residues are considered simultaneously). The result (Table 7, see Additional file) shows that generally a residue, which is functionally important, is also conserved. But in many cases the neighboring residues are also conserved, suggesting the importance of a cluster of π-turn residues in providing the correct environment of the active site.

## Conclusion

π-turns of both hydrogen-bonded and non-hydrogen-bonded types have been analyzed and classified based on the φ, ψ angles of the middle residues. This work generally follows our previous study on α-turns [10]. The presence of shorter four-residue β-turn [9] and five-residue α-turn [10] within the π-turns is observed frequently. The sequence-structure relationship and the context of occurrence of different conformational classes of π-turns enable one to detect subtle features of the turn structures, which are found at the C-terminal end of helices and between two anti-parallel β-strands – thus π-turns have an important role in the termination of both α-helix and β-strands. 37% of π-HB turns occur at surface cavities and 32% of these are involved in binding ligands or constitute active site. Additionally, 77% of π-HB turns have conserved residues among homologous proteins – all these pointing to the functional importance of these turns. The work would be useful in protein structure prediction algorithms [32,33] and in designing sequences [34,35] occurring at the end of helices and in β-hairpin regions.

## Methods

Atomic coordinates were obtained from the Protein Data Bank (PDB) [36] at the Research Collaboratory for Structural Bioinformatics (RCSB). 1608 chains (in 1539 files) were selected using PISCES [37] from PDB files (as of April, 2004) with an R-factor ≤ 20% and resolution of ≤ 2.0 Å and sequence identity between any pair less than 25%. The files are listed in the Additional file (Table 5). Secondary structure assignments were made using the DSSP program [38]. Hydrogen-bonded π-turns (π-HB), distinct from those in Schellman motif, were identified as a stretch of six residues, such that the four central residues were not in helical (G or H, for 3_10_- and α-helices, respectively, according to the DSSP notation) or strand (E) conformation. In the DSSP output the residues in the '5-turn' (as the hydrogen bond is between residues (i) and (i+5)) are marked as '›5555‹', where '›' and '‹' indicate the positions that contribute the carbonyl and amide groups to the hydrogen bond, respectively (the symbols might be replaced by 'X' if both the CO and NH groups of a given residue participated in separate hydrogen bonds). Whether or not a π-turn is involved in the Schellman motif (SCH, Figure 1b,c) was found out by simultaneously noting the secondary structural assignment of the six-residue stretch as HHHTT* (where T corresponds to a turn structure and * indicates any secondary structure type). The SCH turns were further distinguished into SCH_+β _and SCH_-β _types based on the presence or the absence of a β-turn between (i+1) and (i+4)-th residues (given as '›33‹' in DSSP output). π-NHB type of π-turns were identified from the C^α ^(i)-C^α ^(i+5) distance (a cut-off distance of 6.5 Å was used, explained in Results and Discussion).

The propensity of a given residue to occur at a particular location in a secondary structure was calculated as the ratio of the observed number of occurrence to the expected number. A PDB file is mentioned in the text as the four-letter PDB code (in small letter) and if more than one chain is present, the chain identity (ID) is appended (in capital letter). Figures of protein chains were produced using PyMol [39].

The cavities in the structures were determined using VOIDOO [40], run with default conditions. To identify if a residue in a π-turn was important for the function of the protein we used the annotations in PDBSUM [41], which uses CSA [42] for the identification of catalytic residues, PDBSITE [43] for the active-site residues and CONSURF [44] for sequence conservation. CONSURF uses a color code based on the conservation score in the range 9 to 1 (9 being the most conserved and 1 the least). We assigned three levels of conservation, fully conserved (codes 9 and 8), partially conserved (7 and 6), and not conserved (the rest).

## Authors' contributions

PC conceptualized the work that was carried out by BD. BD and PC participated in interpretation of the data and writing the manuscript. Both the authors have read and accepted the final version of the manuscript.

## Supplementary Material

Additional file 1 **One additional file containing three tables, numbered 5 to 7 and three figures, numbered 9 to 11.** Click here for file
