# Supplementary material for: pi-Turns: types, systematics and the context of their occurrence in protein structures
Source: BMC Struct Biol. 2008 Sep 22;8:39. doi: 10.1186/1472-6807-8-39 (PMC2559839; doi:10.1186/1472-6807-8-39)
Supplement: Additional file 1 — One additional file containing three tables, numbered 5 to 7 and three figures, numbered 9 to 11. [file 1472-6807-8-39-S1.doc]

Table 5. List of PDB files used

IDs length Exptl. resolution R-factor FreeRvalue

1CB8A 678 XRAY 1.900 0.20 0.26

7ODCA 424 XRAY 1.600 0.20 0.23

1OUWA 152 XRAY 1.370 0.15 0.18

1WB4A 297 XRAY 1.400 0.11 0.13

1TN6A 382 XRAY 1.800 0.18 0.20

1GAI0 472 XRAY 1.700 0.15 0.17

1L6KA 77 XRAY 2.000 0.19 0.22

2DRI0 271 XRAY 1.600 0.19 1.00

1WVFA 520 XRAY 1.300 0.15 0.17

1W0NA 131 XRAY 0.800 0.13 0.14

1XMBA 418 XRAY 2.000 0.16 0.20

1UJ8A 77 XRAY 1.750 0.19 0.23

1K2XA 177 XRAY 1.650 0.16 0.20

3NUL0 130 XRAY 1.600 0.18 0.20

1JI1A 637 XRAY 1.600 0.18 0.21

1O3UA 135 XRAY 1.750 0.18 0.24

1OD3A 168 XRAY 1.000 0.13 0.15

1ICXA 155 XRAY 1.950 0.20 0.25

1UBKS 267 XRAY 1.180 0.12 0.15

1L3SA 580 XRAY 1.700 0.20 0.23

1OR7A 194 XRAY 2.000 0.20 0.23

1OEWA 329 XRAY 0.900 0.12 0.15

1ED8A 449 XRAY 1.750 0.20 0.23

1G3P0 217 XRAY 1.460 0.19 0.23

1RH6A 55 XRAY 1.700 0.19 0.23

1DMMA 131 XRAY 1.900 0.19 0.25

1JH6A 189 XRAY 1.800 0.19 0.26

1T9HA 307 XRAY 1.600 0.15 0.18

1NI9A 338 XRAY 2.000 0.19 0.24

1M1NB 522 XRAY 1.160 0.12 0.15

2NACA 393 XRAY 1.800 0.15 1.00

1QMYA 167 XRAY 1.900 0.19 0.23

1OJJA 402 XRAY 1.400 0.15 0.17

1PLC0 99 XRAY 1.330 0.15 1.00

1P0HA 318 XRAY 1.600 0.20 0.24

1KPGA 287 XRAY 2.000 0.19 0.23

1J79A 347 XRAY 1.700 0.19 0.26

1DQAA 467 XRAY 2.000 0.17 0.20

1KMVA 186 XRAY 1.050 0.13 0.18

1SQ9A 397 XRAY 1.900 0.19 0.25

1MJ5A 302 XRAY 0.950 0.11 0.14

1SVPA 161 XRAY 2.000 0.18 0.28

1JNRA 643 XRAY 1.600 0.18 0.20

1P9HA 226 XRAY 1.550 0.20 0.20

1MLA0 309 XRAY 1.500 0.18 1.00

1RLJA 139 XRAY 2.000 0.19 0.22

1GSJA 258 XRAY 1.850 0.20 0.23

1MG4A 113 XRAY 1.500 0.15 0.19

1VKBA 161 XRAY 1.900 0.14 0.17

2OLBA 517 XRAY 1.400 0.18 1.00

1OAA0 259 XRAY 1.250 0.20 0.22

1JB3A 131 XRAY 1.600 0.20 0.24

1WQJI 70 XRAY 1.600 0.19 0.26

1HBNC 248 XRAY 1.160 0.12 0.19

1WQJB 80 XRAY 1.600 0.19 0.26

1CS0A 1073 XRAY 2.000 0.19 0.24

1DOWA 205 XRAY 1.800 0.20 0.23

1VZIA 126 XRAY 1.150 0.14 1.00

1AIL0 73 XRAY 1.900 0.18 0.23

1R5ZA 323 XRAY 1.950 0.19 0.25

1CHMA 401 XRAY 1.900 0.18 1.00

1CXPC 466 XRAY 1.800 0.20 0.24

1KDKA 177 XRAY 1.700 0.19 0.24

1O54A 277 XRAY 1.650 0.16 0.19

1XFIA 367 XRAY 1.700 0.17 0.22

1R45A 204 XRAY 1.570 0.17 0.20

1QFTA 175 XRAY 1.250 0.18 1.00

1EAQA 140 XRAY 1.250 0.15 0.17

1F8AB 167 XRAY 1.840 0.06 0.27

5CSMA 256 XRAY 2.000 0.19 0.24

1WWCA 118 XRAY 1.900 0.19 0.29

1GPR0 162 XRAY 1.900 0.16 1.00

1OZ2A 331 XRAY 1.550 0.19 0.22

1QWZA 235 XRAY 1.750 0.19 0.23

1FO8A 343 XRAY 1.400 0.17 0.18

1VP2A 208 XRAY 1.780 0.17 0.20

1UK8A 282 XRAY 1.600 0.18 0.19

1IAB0 200 XRAY 1.790 0.16 1.00

1D3VA 323 XRAY 1.700 0.16 0.18

1J7GA 144 XRAY 1.640 0.19 0.22

1MJ4A 82 XRAY 1.200 0.12 0.14

1RYIA 382 XRAY 1.800 0.18 0.21

1GK9B 557 XRAY 1.300 0.15 0.17

1URQA 63 XRAY 2.000 0.17 0.23

1RQPA 299 XRAY 1.800 0.17 0.22

1LST0 239 XRAY 1.800 0.17 1.00

3CHBD 104 XRAY 1.250 0.13 0.18

1JCDA 52 XRAY 1.300 0.15 0.21

1IXH0 321 XRAY 0.980 0.12 0.14

1PMMA 466 XRAY 2.000 0.18 0.21

1XODA 118 XRAY 1.150 0.15 0.18

1OBOA 169 XRAY 1.200 0.19 0.20

1BM80 99 XRAY 1.710 0.20 0.26

1CCWB 483 XRAY 1.600 0.14 0.17

1I7QB 193 XRAY 1.950 0.18 0.25

1WHZA 70 XRAY 1.520 0.15 0.21

1DBWA 126 XRAY 1.600 0.19 0.22

1WHI0 122 XRAY 1.500 0.19 0.26

1K0MA 241 XRAY 1.400 0.14 0.18

1JNIA 123 XRAY 1.250 0.16 0.21

1H65A 270 XRAY 2.000 0.19 0.23

1RWIA 270 XRAY 1.800 0.19 0.22

1UJ2A 252 XRAY 1.800 0.19 0.22

1TJJA 164 XRAY 2.000 0.19 0.23

3DAAA 277 XRAY 1.900 0.20 0.24

1O5UA 101 XRAY 1.830 0.17 0.22

1M15A 357 XRAY 1.200 0.12 0.14

1DGWA 178 XRAY 1.700 0.20 0.25

1JY3P 48 XRAY 1.600 0.19 0.22

1JU3A 583 XRAY 1.580 0.20 0.21

1GPQA 135 XRAY 1.600 0.18 0.21

2PVBA 107 XRAY 0.910 0.11 0.13

1ATG0 231 XRAY 1.200 0.16 0.18

1CUJ0 214 XRAY 1.600 0.16 1.00

1XTPA 254 XRAY 1.940 0.16 0.22

1EAJA 126 XRAY 1.350 0.14 0.15

1V7ZA 260 XRAY 1.600 0.18 0.20

1LTZA 297 XRAY 1.400 0.16 0.22

1G9GA 629 XRAY 1.900 0.17 0.20

1NA3A 91 XRAY 1.550 0.18 0.21

1F7LA 121 XRAY 1.500 0.19 0.20

1O6AA 96 XRAY 1.850 0.17 0.21

1UPSA 420 XRAY 1.820 0.18 0.20

1IAZA 179 XRAY 1.900 0.19 0.23

1UBKL 534 XRAY 1.180 0.12 0.15

1SJWA 144 XRAY 1.350 0.14 0.18

1JBEA 128 XRAY 1.080 0.10 0.15

1SUUA 312 XRAY 1.750 0.20 0.23

1V93A 296 XRAY 1.900 0.20 0.24

1PBWA 216 XRAY 2.000 0.18 0.23

1GNLA 544 XRAY 1.250 0.13 0.14

1RB90 53 XRAY 0.920 0.07 0.07

1V0EA 666 XRAY 1.900 0.17 0.20

1HBNA 549 XRAY 1.160 0.12 0.19

1W2WB 191 XRAY 1.750 0.17 0.20

1M55A 197 XRAY 1.400 0.18 0.20

1BRT0 277 XRAY 1.500 0.14 0.16

1VIAA 175 XRAY 1.570 0.20 0.23

1TKEA 224 XRAY 1.460 0.18 0.21

1FJ2A 232 XRAY 1.500 0.18 0.24

1POT0 325 XRAY 1.800 0.20 1.00

1T92A 116 XRAY 1.600 0.17 0.20

1Q92A 197 XRAY 1.400 0.14 0.17

1WDEA 294 XRAY 2.000 0.20 0.24

1QOPB 396 XRAY 1.400 0.15 0.18

1UPGA 102 XRAY 1.800 0.19 0.22

1VK5A 157 XRAY 1.600 0.16 0.18

1SU8A 636 XRAY 1.100 0.15 0.18

1WHSB 153 XRAY 2.000 0.17 1.00

1OYGA 447 XRAY 1.500 0.17 0.17

1P1JA 533 XRAY 1.700 0.17 0.19

1NWAA 203 XRAY 1.500 0.16 0.18

1PE9A 361 XRAY 1.600 0.20 0.21

1NH0A 99 XRAY 1.030 0.13 0.17

1FXOA 293 XRAY 1.660 0.14 0.20

1QB7A 236 XRAY 1.500 0.20 0.24

1A6M0 151 XRAY 1.000 0.13 0.16

1DVOA 152 XRAY 2.000 0.20 0.22

1VIE0 62 XRAY 1.700 0.19 1.00

1YN9A 169 XRAY 1.500 0.17 0.19

1X99A 145 XRAY 1.400 0.17 0.19

1WDCC 156 XRAY 2.000 0.19 0.28

1SZWA 379 XRAY 2.000 0.19 0.24

1ITXA 419 XRAY 1.100 0.12 0.14

1GCA0 309 XRAY 1.700 0.19 1.00

1O4WA 147 XRAY 1.900 0.19 0.23

1H6LA 353 XRAY 1.800 0.20 0.22

1NC1A 242 XRAY 2.000 0.19 0.22

1TY9A 222 XRAY 1.800 0.20 0.25

1LUCA 355 XRAY 1.500 0.18 1.00

1G8KA 825 XRAY 1.640 0.15 0.18

1QQFA 277 XRAY 1.450 0.16 0.22

1K5NA 276 XRAY 1.090 0.12 0.15

1OHLA 342 XRAY 1.600 0.19 0.24

1UHKA 191 XRAY 1.600 0.17 0.20

1XD3B 75 XRAY 1.450 0.18 0.19

1J53A 186 XRAY 1.800 0.20 0.23

1UGPA 203 XRAY 1.630 0.18 0.20

1A4IA 301 XRAY 1.500 0.20 0.23

1UV7A 110 XRAY 1.700 0.20 0.24

1C75A 71 XRAY 0.970 0.12 0.11

1BDO0 80 XRAY 1.800 0.19 1.00

1C2AA 120 XRAY 1.900 0.19 0.22

1D8WA 426 XRAY 1.600 0.18 0.24

1U00A 227 XRAY 1.950 0.18 0.21

1OPD0 85 XRAY 1.500 0.18 0.23

1GXUA 91 XRAY 1.270 0.13 0.16

1NYTA 271 XRAY 1.500 0.14 0.17

1NZ0A 118 XRAY 1.200 0.16 0.22

1JPZA 473 XRAY 1.650 0.18 0.19

1LR5A 163 XRAY 1.900 0.20 0.24

2PGD0 482 XRAY 2.000 0.20 1.00

1BEA0 127 XRAY 1.950 0.20 0.29

1ORUA 195 XRAY 1.800 0.20 0.23

1OAIA 59 XRAY 1.000 0.15 0.16

1OGOX 574 XRAY 1.650 0.19 0.21

1NU0A 138 XRAY 1.600 0.19 0.23

1E85A 127 XRAY 1.350 0.19 0.22

1V8AA 265 XRAY 1.850 0.16 0.18

1HDOA 206 XRAY 1.150 0.12 0.16

1ALVA 173 XRAY 1.900 0.19 0.23

1LV7A 257 XRAY 1.500 0.15 0.18

1PWGA 349 XRAY 1.070 0.11 0.15

1MTYB 384 XRAY 1.700 0.18 1.00

1RU4A 400 XRAY 1.600 0.18 0.19

1KT6A 183 XRAY 1.100 0.19 0.22

1O7IA 119 XRAY 1.200 0.19 0.22

1N7HA 381 XRAY 1.800 0.18 0.20

1VR8A 142 XRAY 1.750 0.15 0.18

1VJPA 394 XRAY 1.700 0.18 0.20

1QZRA 418 XRAY 1.900 0.20 0.24

1PRXA 224 XRAY 2.000 0.19 0.26

1SUR0 215 XRAY 2.000 0.19 0.23

1SR4C 166 XRAY 2.000 0.18 0.21

1SDWA 314 XRAY 1.850 0.20 0.23

1W1OA 534 XRAY 1.700 0.20 0.21

1AMX0 180 XRAY 2.000 0.20 0.25

1V4BA 200 XRAY 1.800 0.19 0.23

1P3CA 215 XRAY 1.500 0.16 0.18

1NQEA 594 XRAY 2.000 0.20 0.23

1JM0A 50 XRAY 1.700 0.20 0.25

1O98A 511 XRAY 1.400 0.19 0.20

1M2RA 327 XRAY 1.700 0.19 0.22

1UMHA 184 XRAY 2.000 0.15 0.19

1M3UA 264 XRAY 1.800 0.16 0.19

1UNNC 115 XRAY 1.900 0.18 0.24

1PXVC 111 XRAY 1.800 0.19 0.22

1VQSA 116 XRAY 1.500 0.15 0.18

1UFOA 238 XRAY 1.600 0.17 0.21

1K12A 158 XRAY 1.900 0.19 0.24

1NY1A 240 XRAY 1.800 0.18 0.21

1M93C 41 XRAY 1.650 0.19 0.24

1A1IA 90 XRAY 1.600 0.19 0.22

1KTGA 138 XRAY 1.800 0.19 0.22

1GL2B 65 XRAY 1.900 0.18 0.22

1NSZA 347 XRAY 1.750 0.17 0.22

1CRUA 454 XRAY 1.500 0.18 0.20

1SNRA 341 XRAY 1.310 0.12 0.14

1GYBA 125 XRAY 1.900 0.20 0.23

1CNZA 363 XRAY 1.760 0.20 0.26

1NNFA 309 XRAY 1.100 0.16 0.18

1W9HA 427 XRAY 1.950 0.19 0.24

1I88A 389 XRAY 1.450 0.20 0.22

1UC7A 125 XRAY 1.900 0.19 0.22

1RK6A 496 XRAY 1.430 0.19 0.20

1G1TA 157 XRAY 1.500 0.20 0.22

1UUYA 167 XRAY 1.450 0.16 0.18

1REC0 201 XRAY 1.900 0.19 1.00

1VPBA 451 XRAY 1.750 0.18 0.20

1OBDA 306 XRAY 1.400 0.16 0.20

1TZVA 142 XRAY 1.350 0.20 0.21

2DTR0 226 XRAY 1.900 0.02 1.00

1Q35A 320 XRAY 1.200 0.17 0.20

1EZGA 84 XRAY 1.400 0.16 0.20

1C520 131 XRAY 1.280 0.19 0.22

1EU1A 780 XRAY 1.300 0.12 0.14

1KUGA 203 XRAY 1.370 0.17 0.20

1AF70 274 XRAY 2.000 0.20 0.28

1VPRA 374 XRAY 1.800 0.20 0.23

1NSJ0 205 XRAY 2.000 0.19 0.24

1X7YA 400 XRAY 1.570 0.15 0.17

1RKQA 282 XRAY 1.400 0.18 0.21

1XFOA 357 XRAY 1.960 0.18 0.21

1XB3A 128 XRAY 1.500 0.17 0.21

1NKD0 65 XRAY 1.090 0.10 0.13

1K6ZA 141 XRAY 2.000 0.19 0.25

1E19A 314 XRAY 1.500 0.18 0.22

1E6BA 221 XRAY 1.650 0.20 0.23

1FYEA 229 XRAY 1.200 0.16 0.19

1R6WA 322 XRAY 1.620 0.17 0.20

1SACA 204 XRAY 2.000 0.18 1.00

1MJSA 197 XRAY 1.910 0.20 0.20

1ROCA 155 XRAY 1.500 0.20 0.25

1H72C 296 XRAY 1.800 0.18 0.21

1UCSA 64 XRAY 0.620 0.14 0.15

1TJVA 474 XRAY 2.000 0.19 0.21

1XDWA 331 XRAY 1.980 0.18 0.23

1EB6A 177 XRAY 1.000 0.10 0.13

1PPRM 312 XRAY 2.000 0.18 0.20

1BF20 750 XRAY 2.000 0.16 0.21

1LQTA 456 XRAY 1.050 0.13 0.15

1OCYA 198 XRAY 1.500 0.14 0.15

1CC8A 73 XRAY 1.020 0.14 0.17

1RJ1A 151 XRAY 1.870 0.19 0.23

1VLS0 146 XRAY 1.850 0.20 1.00

1QF5A 431 XRAY 2.000 0.20 0.23

1R9LA 309 XRAY 1.590 0.15 0.19

1H7EA 245 XRAY 1.830 0.16 0.21

1CPO0 299 XRAY 1.900 0.18 0.22

1DGWY 93 XRAY 1.700 0.20 0.25

1NB9A 147 XRAY 1.700 0.18 0.21

1HX6A 394 XRAY 1.650 0.19 0.21

1KQ6A 141 XRAY 1.180 0.13 0.18

1RXQA 178 XRAY 1.700 0.19 0.22

1L6RA 227 XRAY 1.400 0.17 0.20

1QTNB 95 XRAY 1.200 0.17 0.19

1FCJA 322 XRAY 2.000 0.20 0.25

1QXOA 388 XRAY 2.000 0.16 0.22

1O50A 157 XRAY 1.870 0.19 0.26

1KNMA 130 XRAY 1.200 0.13 0.17

1IW0A 215 XRAY 1.400 0.17 0.19

2TGI0 112 XRAY 1.800 0.17 1.00

1D4OA 184 XRAY 1.210 0.17 0.22

1K55A 246 XRAY 1.390 0.15 0.18

1TBFA 347 XRAY 1.300 0.16 0.18

1EPXA 370 XRAY 1.800 0.17 0.21

1Y6XA 93 XRAY 1.250 0.18 0.21

1J34C 46 XRAY 1.550 0.18 0.21

1R4XA 275 XRAY 1.900 0.17 0.21

1SLUA 142 XRAY 1.800 0.19 0.28

1SFXA 109 XRAY 1.550 0.15 0.18

1D3GA 367 XRAY 1.600 0.17 0.19

1O7QA 289 XRAY 1.300 0.12 0.15

1VLPA 441 XRAY 1.750 0.17 0.21

1VQZA 341 XRAY 1.990 0.16 0.22

1JFRA 262 XRAY 1.900 0.14 0.19

1GP6A 356 XRAY 1.750 0.19 0.22

1ES9A 232 XRAY 1.300 0.19 0.22

4MT20 62 XRAY 2.000 0.20 1.00

1JW9B 249 XRAY 1.700 0.18 0.21

1G66A 207 XRAY 0.900 0.11 0.13

1OR0B 528 XRAY 2.000 0.19 0.18

1G2BA 62 XRAY 1.120 0.15 0.20

1OI7A 288 XRAY 1.230 0.18 0.20

1I1WA 303 XRAY 0.890 0.09 0.11

1UFYA 122 XRAY 0.960 0.11 0.13

1CRZA 403 XRAY 1.950 0.19 0.24

1LC5A 364 XRAY 1.460 0.19 0.21

1AYOA 130 XRAY 1.900 0.20 0.24

1JFBA 404 XRAY 1.000 0.10 0.14

1F60A 458 XRAY 1.670 0.19 0.22

1DUVG 333 XRAY 1.700 0.19 0.22

1JRAA 117 XRAY 2.000 0.20 0.23

1MSK0 331 XRAY 1.800 0.20 0.26

1NEPA 130 XRAY 1.700 0.20 0.21

1UWFA 158 XRAY 1.690 0.18 0.22

1IS3A 135 XRAY 1.450 0.20 0.23

1NOX0 205 XRAY 1.590 0.19 0.20

1BD3A 243 XRAY 1.930 0.19 1.00

1H4RA 314 XRAY 1.800 0.19 0.23

1TX4A 198 XRAY 1.650 0.17 0.21

1FX2A 235 XRAY 1.460 0.18 0.20

1EC7A 446 XRAY 1.900 0.19 1.00

3EZMA 101 XRAY 1.500 0.18 0.21

1U60A 310 XRAY 1.610 0.14 0.18

1PZ4A 116 XRAY 1.350 0.19 0.23

1GWUA 309 XRAY 1.310 0.17 0.19

1GPUA 680 XRAY 1.860 0.20 0.23

1KKOA 413 XRAY 1.330 0.16 0.19

1EOKA 290 XRAY 1.800 0.17 0.20

1GXYA 226 XRAY 1.710 0.19 0.22

1PL3A 186 XRAY 1.900 0.17 0.20

1F86A 115 XRAY 1.100 0.14 0.18

1VHNA 318 XRAY 1.590 0.19 0.23

1J2RA 199 XRAY 1.300 0.15 0.17

1TZYB 126 XRAY 1.900 0.19 0.22

1X74A 360 XRAY 1.790 0.16 0.19

1UEKA 275 XRAY 1.700 0.18 0.22

1DG6A 191 XRAY 1.300 0.14 0.20

1H97A 147 XRAY 1.170 0.12 0.17

1QG8A 255 XRAY 1.500 0.16 0.21

1F1EA 154 XRAY 1.370 0.17 0.21

1VLAA 150 XRAY 1.800 0.17 0.22

2BJ4A 252 XRAY 2.000 0.19 0.22

1MTPB 43 XRAY 1.500 0.20 0.22

1D0DA 60 XRAY 1.620 0.19 0.21

1QHDA 398 XRAY 1.950 0.19 0.22

1XQHA 264 XRAY 1.750 0.19 0.22

1PSZA 303 XRAY 2.000 0.18 0.24

1P57A 114 XRAY 1.750 0.19 0.21

8ACN0 754 XRAY 2.000 0.16 1.00

1ORC0 71 XRAY 1.540 0.18 1.00

1HF8A 289 XRAY 2.000 0.19 0.22

1NH8A 304 XRAY 1.800 0.20 0.24

1TAFB 70 XRAY 2.000 0.20 0.24

1M48A 133 XRAY 1.950 0.20 0.27

1Y5IC 225 XRAY 1.900 0.18 0.21

1FSJB 134 XRAY 1.800 0.19 0.22

1VKHA 273 XRAY 1.850 0.14 0.19

1Y0HA 105 XRAY 1.600 0.18 0.22

1VK1A 242 XRAY 1.200 0.17 0.17

1Y0EA 223 XRAY 1.950 0.18 0.22

1T6EX 381 XRAY 1.700 0.18 0.21

1RTQA 299 XRAY 0.950 0.14 0.15

1JR7A 311 XRAY 2.000 0.19 0.24

1ICFI 65 XRAY 2.000 0.18 0.21

1FK5A 93 XRAY 1.300 0.14 0.19

1S4KA 120 XRAY 1.900 0.19 0.22

1O1ZA 234 XRAY 1.600 0.14 0.18

1WKQA 164 XRAY 1.170 0.16 0.18

1E5PA 151 XRAY 1.630 0.19 0.23

1XQOA 256 XRAY 1.030 0.16 0.18

1LL2A 333 XRAY 1.900 0.20 0.23

1NBCA 155 XRAY 1.750 0.19 0.25

1E4CP 215 XRAY 1.660 0.16 0.20

1TUWA 109 XRAY 1.900 0.20 0.24

1TWDA 256 XRAY 1.700 0.19 0.21

1JU2A 536 XRAY 1.470 0.16 0.19

1AQ0A 306 XRAY 2.000 0.17 0.21

1AFWA 393 XRAY 1.800 0.19 0.24

1L9XA 315 XRAY 1.600 0.18 0.20

1QZ5A 375 XRAY 1.450 0.17 0.19

1JAYA 212 XRAY 1.650 0.18 0.21

1RG8A 146 XRAY 1.100 0.15 0.17

1V5VA 401 XRAY 1.500 0.20 0.21

1K7JA 206 XRAY 1.400 0.20 0.20

1SR9A 644 XRAY 2.000 0.17 0.20

1IXKA 315 XRAY 1.900 0.20 0.23

1TJOA 182 XRAY 1.600 0.17 0.21

1IDPA 172 XRAY 1.450 0.20 0.23

1FTRA 296 XRAY 1.700 0.20 0.25

1F9YA 158 XRAY 0.890 0.11 0.13

1Y8AA 332 XRAY 1.400 0.18 0.22

1ATZA 189 XRAY 1.800 0.17 0.24

1X91A 153 XRAY 1.500 0.18 0.21

1MXIA 160 XRAY 1.700 0.20 0.25

1BTEA 97 XRAY 1.500 0.18 0.22

1NLNA 204 XRAY 1.600 0.14 0.18

1SRVA 145 XRAY 1.700 0.19 0.26

2FDN0 55 XRAY 0.940 0.10 0.09

1LKKA 105 XRAY 1.000 0.13 1.00

1WTEA 272 XRAY 1.900 0.17 0.22

1HD2A 161 XRAY 1.500 0.13 0.17

1VQUA 374 XRAY 1.850 0.18 0.22

1HQ0A 295 XRAY 1.830 0.19 0.21

1LUGA 259 XRAY 0.950 0.12 0.14

1SXRA 183 XRAY 1.560 0.18 0.21

1Q6ZA 528 XRAY 1.000 0.12 0.14

1NWWA 149 XRAY 1.200 0.15 0.17

1AYFA 105 XRAY 1.850 0.19 0.25

1W4RA 195 XRAY 1.830 0.16 0.19

1F74A 293 XRAY 1.600 0.17 0.20

1CJWA 166 XRAY 1.800 0.18 0.23

1Q7LB 88 XRAY 1.400 0.13 0.17

1G5TA 196 XRAY 1.800 0.19 0.27

1V6ZA 228 XRAY 2.000 0.20 0.25

1NDBA 596 XRAY 1.800 0.19 0.22

1OTKA 249 XRAY 2.000 0.19 0.23

1GWEA 503 XRAY 0.880 0.09 0.10

1DCIA 275 XRAY 1.500 0.18 0.20

1TAFA 68 XRAY 2.000 0.20 0.24

4EUGA 229 XRAY 1.400 0.18 0.18

1C1DA 355 XRAY 1.250 0.20 0.24

1FIPA 98 XRAY 1.900 0.20 1.00

1DS1A 324 XRAY 1.080 0.14 0.17

1W6SA 599 XRAY 1.200 0.15 0.18

1LK2B 99 XRAY 1.350 0.15 0.16

1DJ0A 264 XRAY 1.500 0.16 0.22

1UZBA 516 XRAY 1.400 0.18 0.19

1NVMA 345 XRAY 1.700 0.19 0.23

1S99A 200 XRAY 1.650 0.17 0.22

7A3HA 303 XRAY 0.950 0.11 0.13

1A4YA 460 XRAY 2.000 0.19 0.29

1OLLA 188 XRAY 1.930 0.20 0.25

1W96A 554 XRAY 1.800 0.20 0.23

1OOYA 481 XRAY 1.700 0.17 0.19

1L5OA 356 XRAY 1.600 0.17 0.20

1QNRA 344 XRAY 1.400 0.12 0.17

1KQFC 217 XRAY 1.600 0.18 0.20

1N13B 113 XRAY 1.400 0.20 0.21

1CEWI 108 XRAY 2.000 0.20 1.00

1NQJA 119 XRAY 1.000 0.14 0.17

1PBYB 337 XRAY 1.700 0.19 0.22

1F46A 140 XRAY 1.500 0.20 0.22

1E29A 135 XRAY 1.210 0.15 0.21

1KLLA 130 XRAY 1.500 0.12 0.20

1UT1A 148 XRAY 1.700 0.17 0.21

1O4SA 389 XRAY 1.900 0.16 0.20

1U6DX 308 XRAY 1.850 0.15 0.18

2BKXA 242 XRAY 1.400 0.12 0.17

1J6OA 268 XRAY 1.800 0.19 0.22

1UPQA 123 XRAY 1.480 0.18 0.24

2RN20 155 XRAY 1.480 0.20 1.00

1TJYA 316 XRAY 1.300 0.16 0.17

1S3EA 520 XRAY 1.600 0.20 0.22

1H1NA 305 XRAY 1.120 0.13 0.17

1HXN0 219 XRAY 1.800 0.18 1.00

2FDX0 138 XRAY 1.650 0.19 1.00

1YDGA 211 XRAY 2.000 0.19 0.24

1V3EA 431 XRAY 1.890 0.18 0.22

1Q8FA 313 XRAY 1.700 0.15 0.17

1K3IA 656 XRAY 1.400 0.18 0.19

1O6SB 105 XRAY 1.800 0.17 0.22

1XG0A 76 XRAY 0.970 0.11 0.13

1VL7A 157 XRAY 1.500 0.16 0.18

1VKNA 351 XRAY 1.800 0.18 0.21

1I24A 404 XRAY 1.200 0.19 0.20

1NKZA 53 XRAY 2.000 0.17 0.19

1F20A 435 XRAY 1.900 0.19 0.21

1NO5A 114 XRAY 1.800 0.20 0.28

1TYV0 554 XRAY 1.800 0.18 1.00

1M93B 245 XRAY 1.650 0.19 0.24

1DWKA 156 XRAY 1.650 0.14 0.18

1KHBA 625 XRAY 1.850 0.18 0.23

1UEHA 253 XRAY 1.730 0.18 0.21

1P0ZA 131 XRAY 1.600 0.17 0.19

1OJRA 274 XRAY 1.350 0.11 0.14

1QFMA 710 XRAY 1.400 0.19 0.21

1DQGA 135 XRAY 1.700 0.20 0.21

1PUC0 105 XRAY 1.950 0.19 0.24

1VPMA 169 XRAY 1.660 0.17 0.20

1VHWA 253 XRAY 1.540 0.16 0.19

1FVIA 297 XRAY 2.000 0.19 0.27

1RFXA 94 XRAY 2.000 0.18 0.22

1M40A 263 XRAY 0.850 0.09 0.11

1SQSA 242 XRAY 1.500 0.20 0.22

1JBOA 162 XRAY 1.450 0.15 0.19

1C4QA 69 XRAY 1.520 0.17 0.19

1W2YA 229 XRAY 1.650 0.15 0.19

1P42A 270 XRAY 2.000 0.20 0.21

1DQZA 280 XRAY 1.500 0.19 0.18

1C7KA 132 XRAY 1.000 0.15 0.18

1T3IA 420 XRAY 1.800 0.19 0.22

1XRKA 124 XRAY 1.500 0.17 0.19

1HUW0 191 XRAY 2.000 0.18 1.00

1GL2A 65 XRAY 1.900 0.18 0.22

1LWDA 413 XRAY 1.850 0.20 0.21

1YJFA 237 XRAY 1.350 0.14 0.20

1L2HA 153 XRAY 1.540 0.15 0.19

1TI6B 274 XRAY 2.000 0.17 0.20

1UOZA 315 XRAY 1.100 0.11 0.13

1ARB0 268 XRAY 1.200 0.15 1.00

1WDJA 187 XRAY 2.000 0.19 0.24

1P99A 295 XRAY 1.700 0.20 0.24

1QY1A 174 XRAY 1.700 0.18 0.21

1VMEA 410 XRAY 1.800 0.15 0.18

1J9LA 247 XRAY 1.900 0.20 0.24

1B25A 619 XRAY 1.850 0.17 0.22

1KEXA 155 XRAY 1.900 0.19 0.27

1T5HX 504 XRAY 2.000 0.18 0.21

4BCL0 366 XRAY 1.900 0.18 1.00

1IZMA 184 XRAY 1.950 0.19 0.26

1M33A 258 XRAY 1.700 0.15 0.19

1OD6A 160 XRAY 1.500 0.20 0.20

1TVXA 75 XRAY 1.750 0.20 0.25

2VHBA 146 XRAY 1.760 0.20 1.00

1IUAA 83 XRAY 0.800 0.10 0.11

1VP6A 138 XRAY 1.700 0.19 0.22

1PTQ0 50 XRAY 1.950 0.20 1.00

1TQHA 247 XRAY 1.630 0.17 0.23

1OX0A 430 XRAY 1.300 0.14 0.16

1KHCA 147 XRAY 1.800 0.19 0.24

1K3XA 262 XRAY 1.250 0.15 0.18

1GA8A 311 XRAY 2.000 0.19 0.23

1Y0BA 197 XRAY 1.800 0.18 0.23

1KPTA 105 XRAY 1.750 0.17 0.22

1T06A 235 XRAY 1.900 0.20 0.24

1Y21A 282 XRAY 1.750 0.17 0.21

1MDC0 132 XRAY 1.750 0.17 1.00

1BX4A 345 XRAY 1.500 0.19 0.23

1P3DA 475 XRAY 1.700 0.17 0.19

1ON3A 523 XRAY 1.900 0.17 0.21

1Y4JA 284 XRAY 1.860 0.17 0.20

1ISPA 181 XRAY 1.300 0.19 0.23

1X82A 190 XRAY 1.500 0.16 0.21

1TEN0 90 XRAY 1.800 0.20 1.00

1VDKA 466 XRAY 1.800 0.19 0.20

1LM8C 96 XRAY 1.850 0.20 0.24

1R690 69 XRAY 2.000 0.19 1.00

1LSHA 1056 XRAY 1.900 0.19 0.26

1O91A 178 XRAY 1.900 0.18 0.21

2BM3A 166 XRAY 1.800 0.19 0.26

1J1TA 233 XRAY 2.000 0.19 0.25

1I3CA 149 XRAY 1.900 0.19 0.22

1U7GA 385 XRAY 1.400 0.14 0.17

1DUSA 194 XRAY 1.800 0.19 0.23

1E2WA 251 XRAY 1.600 0.20 0.23

1V0AA 178 XRAY 1.980 0.20 0.23

1T6UA 117 XRAY 1.300 0.16 0.20

1J6PA 418 XRAY 1.900 0.17 0.20

1TVZA 388 XRAY 2.000 0.17 0.19

1OSYA 115 XRAY 1.700 0.18 0.21

1D02A 202 XRAY 1.700 0.18 0.21

1NZJA 298 XRAY 1.500 0.15 0.17

1QQP2 218 XRAY 1.900 0.16 1.00

1TL2A 236 XRAY 2.000 0.16 0.20

1I27A 73 XRAY 1.020 0.13 0.15

1KCQA 104 XRAY 1.650 0.19 0.23

1LQVA 193 XRAY 1.600 0.19 0.22

1SBYA 254 XRAY 1.100 0.13 0.17

1ESWA 500 XRAY 1.900 0.19 0.22

1NR0A 611 XRAY 1.700 0.20 0.23

1JY1A 464 XRAY 1.690 0.20 0.23

1HFUA 503 XRAY 1.680 0.18 0.21

1P5XA 245 XRAY 2.000 0.18 0.20

1GKMA 509 XRAY 1.000 0.12 0.14

1OBFO 335 XRAY 1.700 0.17 0.19

1A34A 159 XRAY 1.810 0.18 0.18

1O4VA 183 XRAY 1.770 0.15 0.18

1H05A 146 XRAY 1.500 0.14 0.19

1V5IB 76 XRAY 1.500 0.18 0.19

1R2QA 170 XRAY 1.050 0.12 0.17

1MTYG 162 XRAY 1.700 0.18 1.00

1UWCA 261 XRAY 1.080 0.12 0.14

1VMHA 144 XRAY 1.310 0.16 0.18

1AMF0 233 XRAY 1.750 0.16 0.20

4UBPB 126 XRAY 1.550 0.15 0.19

1PQHA 143 XRAY 1.290 0.15 0.17

1N9EA 787 XRAY 1.650 0.16 0.19

1W2WA 211 XRAY 1.750 0.17 0.20

1CXPA 104 XRAY 1.800 0.20 0.24

1IV2A 152 XRAY 1.550 0.17 0.25

1ECA0 136 XRAY 1.400 0.18 1.00

1VL5A 260 XRAY 1.950 0.18 0.21

1A8VA 121 XRAY 2.000 0.19 0.26

1YGTA 111 XRAY 1.700 0.20 0.23

1M7GA 211 XRAY 1.430 0.18 0.20

1REGX 122 XRAY 1.900 0.18 0.21

1UA4A 455 XRAY 1.900 0.17 0.20

1GGXA 223 XRAY 1.900 0.19 0.21

1RWRA 301 XRAY 1.720 0.15 0.19

1M9XC 146 XRAY 1.700 0.17 0.23

1GA6A 372 XRAY 1.000 0.12 0.13

2ENG0 210 XRAY 1.500 0.10 0.15

1W23A 360 XRAY 1.080 0.12 0.14

1EDG0 380 XRAY 1.600 0.19 0.22

1JG1A 235 XRAY 1.200 0.15 0.20

1J30A 144 XRAY 1.700 0.19 0.22

1I0DA 332 XRAY 1.300 0.19 0.22

1QQP4 85 XRAY 1.900 0.16 1.00

1Q9UA 130 XRAY 1.800 0.20 0.24

1CSEI 71 XRAY 1.200 0.18 1.00

1B4PA 217 XRAY 1.700 0.18 1.00

1UI0A 205 XRAY 1.500 0.18 0.20

1MVLA 209 XRAY 2.000 0.17 0.20

1B65A 375 XRAY 1.820 0.17 0.21

1LML0 478 XRAY 1.860 0.19 0.21

1EF8A 261 XRAY 1.850 0.18 0.24

1M2DA 110 XRAY 1.050 0.14 0.16

1AL30 324 XRAY 1.800 0.18 0.25

1J1YA 136 XRAY 1.700 0.18 0.19

1UGPB 226 XRAY 1.630 0.18 0.20

1V0WA 506 XRAY 1.350 0.16 0.19

1GX5A 536 XRAY 1.700 0.19 0.22

1RDQE 350 XRAY 1.260 0.13 0.16

1RW1A 114 XRAY 1.020 0.13 0.14

1V74B 87 XRAY 2.000 0.19 0.23

1VKEA 133 XRAY 1.560 0.16 0.18

1EUWA 152 XRAY 1.050 0.14 0.16

1K4IA 233 XRAY 0.980 0.18 0.20

1UAYA 242 XRAY 1.400 0.18 0.20

1R8SA 164 XRAY 1.460 0.16 0.17

1HT6A 405 XRAY 1.500 0.14 0.16

1RE9A 414 XRAY 1.450 0.16 0.22

1KVEA 63 XRAY 1.800 0.17 1.00

1GUTA 68 XRAY 1.500 0.18 0.22

1TV9A 335 XRAY 2.000 0.18 0.24

1GP1A 198 XRAY 2.000 0.17 1.00

1DTDB 61 XRAY 1.650 0.19 0.23

1NOGA 177 XRAY 1.550 0.20 0.21

1BGF0 124 XRAY 1.450 0.19 0.22

1OGSA 497 XRAY 2.000 0.20 0.23

1HYP0 80 XRAY 1.800 0.19 1.00

1IZCA 339 XRAY 1.700 0.18 0.20

1O94D 320 XRAY 2.000 0.17 0.21

1H4XA 117 XRAY 1.160 0.13 0.16

1AY7B 89 XRAY 1.700 0.16 0.19

1KJQA 391 XRAY 1.050 0.19 0.21

1PKHA 204 XRAY 1.420 0.19 0.20

1JFXA 217 XRAY 1.650 0.15 0.18

1VJUA 309 XRAY 1.400 0.15 0.18

1QLMA 316 XRAY 2.000 0.20 0.22

1DP4A 435 XRAY 2.000 0.20 0.23

1XVWA 160 XRAY 1.900 0.18 0.21

1RXZA 245 XRAY 2.000 0.19 0.26

1DLJA 402 XRAY 1.800 0.18 0.21

1V05A 96 XRAY 1.430 0.19 0.20

1OYJA 231 XRAY 1.950 0.18 0.23

1XV2A 237 XRAY 2.000 0.19 0.24

1RJDA 334 XRAY 1.800 0.18 0.21

7AHLA 293 XRAY 1.890 0.20 0.26

1T8UA 272 XRAY 1.950 0.20 0.22

1JNDA 420 XRAY 1.300 0.18 0.20

1PZTA 286 XRAY 1.920 0.19 0.23

1UUJA 88 XRAY 1.750 0.19 0.25

1JDRA 294 XRAY 1.500 0.20 0.23

1R4VA 171 XRAY 1.900 0.18 0.21

1Y12A 165 XRAY 1.950 0.18 0.23

1CMCA 104 XRAY 1.800 0.18 1.00

1LFWA 470 XRAY 1.800 0.17 1.00

1GE7A 167 XRAY 2.000 0.18 0.22

1IOW0 306 XRAY 1.900 0.16 0.23

1QW9A 502 XRAY 1.200 0.17 0.18

1QU9A 128 XRAY 1.200 0.16 1.00

1BS0A 384 XRAY 1.650 0.18 0.21

1SUZA 244 XRAY 1.800 0.20 0.24

1H0AA 158 XRAY 1.700 0.19 0.23

1SWXA 209 XRAY 1.650 0.17 0.23

1Y0UA 96 XRAY 1.600 0.17 0.20

1N8KA 374 XRAY 1.130 0.14 0.17

1J0PA 108 XRAY 0.910 0.11 0.14

1T3TA 1303 XRAY 1.900 0.19 0.21

1SFP0 114 XRAY 1.900 0.17 0.14

1NLS0 237 XRAY 0.940 0.13 0.15

1SFSA 240 XRAY 1.070 0.10 0.14

1WLGA 299 XRAY 1.800 0.19 0.24

1YM3A 215 XRAY 1.750 0.18 0.23

1UWZA 136 XRAY 1.990 0.19 0.21

1GUQA 348 XRAY 1.800 0.17 1.00

2BKAA 242 XRAY 1.700 0.17 0.19

1R29A 127 XRAY 1.300 0.13 0.17

1V3HA 495 XRAY 1.600 0.16 0.18

1S1DA 331 XRAY 1.600 0.17 0.19

1LR0A 129 XRAY 1.910 0.18 0.22

1SG4A 260 XRAY 1.300 0.16 0.20

1R6XA 395 XRAY 1.400 0.20 0.22

1UKKA 142 XRAY 1.600 0.18 0.23

1MWQA 101 XRAY 0.990 0.11 0.13

1B68A 191 XRAY 2.000 0.20 0.26

1M4IA 181 XRAY 1.500 0.18 0.20

1ISUA 62 XRAY 1.500 0.17 1.00

1XO7A 166 XRAY 1.610 0.18 0.20

1V2BA 177 XRAY 1.600 0.19 0.21

1FM0D 81 XRAY 1.450 0.15 0.18

1IV8A 720 XRAY 1.900 0.20 0.26

1LRIA 98 XRAY 1.450 0.13 0.19

1CTF0 74 XRAY 1.700 0.17 1.00

1F0LA 535 XRAY 1.550 0.19 0.24

1WOQA 267 XRAY 1.800 0.19 0.22

1BXEA 113 XRAY 1.900 0.19 0.22

1SMBA 154 XRAY 1.550 0.19 0.20

1S0AA 429 XRAY 1.710 0.19 0.21

1LZLA 323 XRAY 1.300 0.15 0.21

1UASA 362 XRAY 1.500 0.16 0.18

1B6A0 478 XRAY 1.600 0.19 0.22

1S9RA 410 XRAY 1.600 0.17 0.20

1EEXA 554 XRAY 1.700 0.16 0.23

1VJVA 415 XRAY 1.740 0.18 0.21

1VM9A 111 XRAY 1.480 0.16 0.18

1XDNA 277 XRAY 1.200 0.13 0.15

1M6SA 347 XRAY 1.800 0.20 0.21

1O9GA 250 XRAY 1.500 0.17 0.20

1JI7A 89 XRAY 1.450 0.18 0.20

1FC3A 120 XRAY 2.000 0.20 0.26

1GMXA 108 XRAY 1.100 0.13 0.15

1DOZA 309 XRAY 1.800 0.18 0.22

1N62B 809 XRAY 1.090 0.14 0.17

1EJ0A 180 XRAY 1.500 0.20 0.23

1BYI0 224 XRAY 0.970 0.12 0.11

1CHD0 203 XRAY 1.750 0.18 0.23

1IJBA 202 XRAY 1.800 0.19 0.21

1G5AA 628 XRAY 1.400 0.19 0.20

1NWZA 125 XRAY 0.820 0.12 0.14

3C2C0 112 XRAY 1.680 0.17 1.00

2DNJA 260 XRAY 2.000 0.17 1.00

1CV80 174 XRAY 1.750 0.18 0.24

1U14A 172 XRAY 1.680 0.18 0.21

1HDKA 141 XRAY 1.800 0.20 0.22

1T0TV 248 XRAY 1.750 0.16 0.19

1USGA 346 XRAY 1.530 0.19 0.22

1X9IA 302 XRAY 1.160 0.15 0.16

1O4YA 288 XRAY 1.480 0.15 0.18

1M0WA 491 XRAY 1.800 0.17 0.20

1NRJB 218 XRAY 1.700 0.20 0.23

1U8VA 490 XRAY 1.600 0.17 0.21

1M4LA 307 XRAY 1.250 0.10 0.14

1QB5D 99 XRAY 1.900 0.18 0.24

1VHUA 211 XRAY 1.340 0.15 0.18

1D7UA 433 XRAY 1.950 0.19 1.00

1BKRA 109 XRAY 1.100 0.14 0.19

1USLA 170 XRAY 1.880 0.17 0.20

2SICI 107 XRAY 1.800 0.18 1.00

1K7CA 233 XRAY 1.120 0.11 0.13

1LBU0 213 XRAY 1.800 0.16 1.00

1FT5A 211 XRAY 1.600 0.19 0.21

1WPNA 188 XRAY 1.300 0.14 0.17

1VKKA 154 XRAY 1.350 0.16 0.19

1GSA0 316 XRAY 2.000 0.19 1.00

1MJUL 219 XRAY 1.220 0.12 0.15

1W0HA 204 XRAY 1.590 0.17 0.20

1UNKA 87 XRAY 1.800 0.18 0.25

1SVB0 395 XRAY 1.900 0.18 0.22

1PN2A 280 XRAY 1.950 0.18 0.21

1K77A 260 XRAY 1.630 0.20 0.21

1RV9A 259 XRAY 1.530 0.20 0.21

1K3YA 221 XRAY 1.300 0.14 0.20

1E5KA 201 XRAY 1.350 0.18 0.21

1W78A 422 XRAY 1.820 0.17 0.20

1LO7A 141 XRAY 1.500 0.16 0.20

1GUIA 155 XRAY 1.900 0.17 0.20

1EUVA 221 XRAY 1.600 0.19 0.25

1VKPA 383 XRAY 1.530 0.15 0.17

1FN9A 365 XRAY 1.800 0.17 0.20

1GL2C 65 XRAY 1.900 0.18 0.22

1V7WA 807 XRAY 1.600 0.16 0.18

1GYUA 140 XRAY 1.810 0.18 0.22

1HDHA 536 XRAY 1.300 0.20 0.23

1RQBA 539 XRAY 1.900 0.16 0.19

1U07A 90 XRAY 1.130 0.14 0.18

1DK8A 147 XRAY 1.570 0.17 0.20

1INLA 296 XRAY 1.500 0.20 0.21

1WTQA 66 XRAY 1.700 0.18 0.23

1XG0C 177 XRAY 0.970 0.11 0.13

1BKF0 107 XRAY 1.600 0.19 1.00

1M65A 245 XRAY 1.570 0.18 0.21

1JM1A 204 XRAY 1.110 0.11 0.12

1FCYA 236 XRAY 1.300 0.13 0.16

1GXMA 332 XRAY 1.320 0.13 0.16

1PZXA 289 XRAY 2.000 0.20 0.25

1S3ZA 165 XRAY 2.000 0.17 0.21

1OJHA 65 XRAY 1.800 0.18 0.22

1MUWA 386 XRAY 0.860 0.13 0.14

1F94A 63 XRAY 0.970 0.12 0.18

1N2ZA 245 XRAY 2.000 0.19 0.21

1I19A 561 XRAY 1.700 0.18 0.20

1MNNA 340 XRAY 1.400 0.20 0.21

1EVFA 264 XRAY 1.700 0.19 0.21

2HTS0 92 XRAY 1.830 0.19 0.25

1K92A 455 XRAY 1.600 0.16 0.19

1PX5A 349 XRAY 1.740 0.19 0.22

1DK0A 188 XRAY 1.770 0.17 0.21

1PGS0 314 XRAY 1.800 0.17 1.00

1FLTX 95 XRAY 1.700 0.20 0.26

1Q7EA 428 XRAY 1.600 0.16 0.19

1VK4A 298 XRAY 1.910 0.17 0.22

1O9RA 162 XRAY 1.450 0.18 0.20

1T0HB 224 XRAY 1.970 0.19 0.21

1MML0 265 XRAY 1.800 0.20 0.26

1QWGA 251 XRAY 1.600 0.17 0.19

1VFJA 116 XRAY 1.700 0.20 0.23

1F8EA 388 XRAY 1.400 0.19 0.22

1KQPA 271 XRAY 1.030 0.11 0.14

1VI9A 299 XRAY 1.960 0.20 0.26

1VFYA 73 XRAY 1.150 0.17 0.18

1VL1A 232 XRAY 1.550 0.17 0.20

1ITUA 369 XRAY 2.000 0.19 0.25

1KW3B 292 XRAY 1.450 0.16 0.18

1WP5A 323 XRAY 1.790 0.16 0.21

1JY3N 53 XRAY 1.600 0.19 0.22

1A8L0 226 XRAY 1.900 0.19 0.22

1TU9A 134 XRAY 1.200 0.16 0.19

1U36A 106 XRAY 1.890 0.19 0.22

1VIMA 200 XRAY 1.360 0.17 0.20

1EYEA 280 XRAY 1.700 0.18 0.24

1FP3A 402 XRAY 2.000 0.17 0.24

1PFVA 551 XRAY 1.700 0.19 0.20

1UGHI 82 XRAY 1.900 0.20 0.26

1M1FA 110 XRAY 1.400 0.16 0.18

1T4BA 367 XRAY 1.600 0.17 0.20

1VNS0 609 XRAY 1.660 0.18 0.22

2GDM0 153 XRAY 1.700 0.16 1.00

1O69A 394 XRAY 1.840 0.18 0.23

1FLEI 57 XRAY 1.900 0.20 1.00

1XX1A 285 XRAY 1.750 0.20 0.23

1DXRC 336 XRAY 2.000 0.19 0.22

1SR4B 261 XRAY 2.000 0.18 0.21

1R0VA 305 XRAY 2.000 0.18 0.24

1OFLA 481 XRAY 1.700 0.14 0.18

1RA4A 120 XRAY 1.860 0.17 0.22

1ITWA 741 XRAY 1.950 0.19 0.23

2MHR0 118 XRAY 1.300 0.16 1.00

1X6OA 174 XRAY 1.600 0.18 0.21

1SRA0 151 XRAY 2.000 0.20 0.25

1W53A 84 XRAY 1.600 0.20 0.23

1KP6A 79 XRAY 1.800 0.16 0.21

1GWMA 153 XRAY 1.150 0.13 0.16

1H2CA 140 XRAY 1.600 0.17 0.18

1QAZA 351 XRAY 1.780 0.18 0.23

1TAG0 324 XRAY 1.800 0.19 0.21

1TQGA 105 XRAY 0.980 0.18 0.21

1TXGA 335 XRAY 1.700 0.15 0.19

1MOF0 55 XRAY 1.700 0.17 0.23

1G8QA 90 XRAY 1.600 0.19 0.24

1G8MA 593 XRAY 1.750 0.20 0.22

1KOE0 172 XRAY 1.500 0.19 0.22

1V7CA 351 XRAY 2.000 0.20 0.23

1WEHA 171 XRAY 1.800 0.18 0.22

1UG6A 431 XRAY 0.990 0.12 0.13

1UWKA 557 XRAY 1.190 0.14 0.18

1Q5YA 85 XRAY 1.400 0.16 0.22

1TZYD 103 XRAY 1.900 0.19 0.22

2BGRA 738 XRAY 2.000 0.16 0.20

1O9IA 266 XRAY 1.330 0.12 0.14

1JF4A 147 XRAY 1.400 0.19 0.21

1W85I 49 XRAY 2.000 0.18 0.21

1K2EA 156 XRAY 1.800 0.18 0.22

1EJGA 46 XRAY 0.540 0.09 0.09

1LYVA 306 XRAY 1.360 0.14 0.19

1OIHA 301 XRAY 1.890 0.17 0.19

1TZYC 136 XRAY 1.900 0.19 0.22

1J8EA 44 XRAY 1.850 0.19 0.22

1MUGA 168 XRAY 1.800 0.20 0.25

1XFFA 240 XRAY 1.800 0.16 0.00

1M2XA 223 XRAY 1.500 0.19 0.21

1FC6A 388 XRAY 1.800 0.17 0.24

1OQVA 192 XRAY 1.300 0.12 0.17

1S9UA 207 XRAY 1.380 0.16 0.18

1U7PA 164 XRAY 1.900 0.19 0.24

1T6T1 118 XRAY 1.800 0.18 0.21

1CZPA 98 XRAY 1.170 0.14 0.18

1YR0A 175 XRAY 2.000 0.19 0.23

1DC1A 323 XRAY 1.700 0.19 0.25

256BA 106 XRAY 1.400 0.16 1.00

1LAM0 484 XRAY 1.600 0.17 0.20

1W1HA 151 XRAY 1.450 0.16 0.21

1A8E0 329 XRAY 1.600 0.18 1.00

2NLRA 234 XRAY 1.200 0.11 0.14

1TUHA 156 XRAY 1.850 0.19 0.21

1N4WA 504 XRAY 0.920 0.10 0.12

1AOHA 147 XRAY 1.700 0.19 0.01

1TIF0 78 XRAY 1.800 0.19 0.23

1SBP0 310 XRAY 1.700 0.18 1.00

1GVNA 90 XRAY 1.950 0.20 0.24

1PBE0 394 XRAY 1.900 0.16 1.00

1M22A 503 XRAY 1.400 0.18 0.20

1PA1A 310 XRAY 1.600 0.19 0.20

8ABP0 306 XRAY 1.490 0.17 1.00

1TG7A 971 XRAY 1.900 0.17 0.18

1A3AA 148 XRAY 1.800 0.19 0.24

1N0QA 93 XRAY 1.260 0.17 0.19

1PBYC 79 XRAY 1.700 0.19 0.22

1TXJA 171 XRAY 2.000 0.20 0.23

1TZPA 255 XRAY 1.400 0.18 0.21

1E6IA 121 XRAY 1.870 0.19 0.21

1QLWA 328 XRAY 1.090 0.14 0.16

1K8WA 327 XRAY 1.850 0.18 0.21

1RX0A 393 XRAY 1.770 0.18 0.19

1S2OA 244 XRAY 1.400 0.18 0.20

1FJJA 159 XRAY 1.660 0.18 0.20

1PQ7A 224 XRAY 0.800 0.11 0.11

1C9OA 66 XRAY 1.170 0.12 0.18

1O1YA 239 XRAY 1.700 0.15 0.17

1RYQA 69 XRAY 1.380 0.19 0.20

1YQHA 109 XRAY 1.700 0.16 0.20

1YPQA 135 XRAY 1.400 0.18 0.19

1MOLA 94 XRAY 1.700 0.17 1.00

1SZOA 257 XRAY 1.900 0.17 0.20

1RYAA 160 XRAY 1.300 0.17 0.19

1X8QA 184 XRAY 0.850 0.10 0.13

1LATA 82 XRAY 1.900 0.20 0.28

1VP4A 425 XRAY 1.820 0.18 0.20

1R26A 125 XRAY 1.400 0.17 0.20

1A680 95 XRAY 1.800 0.20 0.24

2KINB 100 XRAY 2.000 0.19 0.25

1WHSA 255 XRAY 2.000 0.17 1.00

1LB3A 182 XRAY 1.210 0.13 0.16

1H03P 125 XRAY 1.700 0.19 0.23

1IX9A 205 XRAY 0.900 0.11 0.13

1XEDA 117 XRAY 1.900 0.18 0.24

1XBBA 291 XRAY 1.570 0.19 0.22

1PDO0 135 XRAY 1.700 0.19 0.24

1VM0A 130 XRAY 1.800 0.18 0.22

1UNQA 125 XRAY 0.980 0.15 0.18

1J98A 157 XRAY 1.200 0.13 0.15

1MFMA 153 XRAY 1.020 0.12 1.00

1IU8A 206 XRAY 1.600 0.19 0.21

1HX0A 496 XRAY 1.380 0.11 0.13

1VYIA 112 XRAY 1.500 0.17 0.20

1DP7P 76 XRAY 1.500 0.19 0.23

1IPC0 217 XRAY 2.000 0.19 0.23

1TN6B 437 XRAY 1.800 0.18 0.20

1HBNB 442 XRAY 1.160 0.12 0.19

1JQ5A 370 XRAY 1.700 0.15 0.19

1B93A 152 XRAY 1.900 0.18 0.20

1NQUA 154 XRAY 1.750 0.14 0.16

1NPK0 154 XRAY 1.800 0.20 1.00

1O08A 221 XRAY 1.200 0.14 0.18

1NTHA 458 XRAY 1.550 0.18 0.19

1GV9A 260 XRAY 1.460 0.19 0.21

1R5RA 119 XRAY 1.600 0.18 0.21

1QWNA 1045 XRAY 1.200 0.17 0.19

1M0KA 262 XRAY 1.430 0.13 0.18

1R8SE 203 XRAY 1.460 0.16 0.17

1RKUA 206 XRAY 1.470 0.19 0.22

1HN0A 1021 XRAY 1.900 0.16 0.21

1NM1G 125 XRAY 1.800 0.20 0.23

1VMBA 140 XRAY 1.700 0.18 0.24

1HNJA 317 XRAY 1.460 0.17 0.19

2SLI0 679 XRAY 1.800 0.18 0.22

1ONEA 436 XRAY 1.800 0.18 1.00

1T61A 229 XRAY 1.500 0.20 0.20

1X7DA 350 XRAY 1.600 0.17 0.19

1HE1A 135 XRAY 2.000 0.17 0.22

1ZRN0 232 XRAY 1.830 0.20 0.26

1VKIA 181 XRAY 1.600 0.15 0.18

1XMTA 103 XRAY 1.150 0.15 0.17

1I7QA 519 XRAY 1.950 0.18 0.25

1GXQA 106 XRAY 2.000 0.20 0.27

1MK0A 97 XRAY 1.600 0.20 0.22

3CLA0 213 XRAY 1.750 0.16 1.00

1FLMA 122 XRAY 1.300 0.15 0.18

1B3AA 67 XRAY 1.600 0.17 0.24

1QL0A 241 XRAY 1.100 0.13 0.15

1DY5A 124 XRAY 0.870 0.10 0.12

1MPGA 282 XRAY 1.800 0.19 0.25

1V9YA 167 XRAY 1.320 0.16 0.22

2BBKH 355 XRAY 1.750 0.17 1.00

1HW1A 239 XRAY 1.500 0.20 0.22

1O7JA 327 XRAY 1.000 0.11 0.13

1LLFA 534 XRAY 1.400 0.14 0.17

1OQ1A 223 XRAY 1.700 0.18 0.21

1LJ8A 493 XRAY 1.700 0.17 0.20

1V1HA 103 XRAY 1.900 0.18 0.24

1THFD 253 XRAY 1.450 0.20 0.21

1AZ90 440 XRAY 2.000 0.16 0.19

1NE9A 335 XRAY 1.700 0.19 0.21

1IB2A 349 XRAY 1.900 0.20 0.24

1TZYA 129 XRAY 1.900 0.19 0.22

1B5EA 246 XRAY 1.600 0.19 0.21

1O22A 170 XRAY 2.000 0.19 0.24

1DMHA 311 XRAY 1.700 0.18 0.22

1AE9A 179 XRAY 1.900 0.20 0.23

1NC5A 373 XRAY 1.600 0.19 0.21

1N40A 396 XRAY 1.060 0.13 0.15

1UMKA 275 XRAY 1.750 0.19 0.21

1LBWA 252 XRAY 2.000 0.19 0.20

1LU4A 136 XRAY 1.120 0.15 0.22

1Y7RA 133 XRAY 1.700 0.17 0.22

1H4GA 207 XRAY 1.100 0.16 0.18

2BEPA 159 XRAY 1.800 0.17 0.22

1D2NA 272 XRAY 1.750 0.20 0.22

1S5DA 240 XRAY 1.750 0.16 0.19

1QF8A 182 XRAY 1.740 0.19 0.22

1Y0KA 209 XRAY 1.750 0.19 0.22

2POR0 301 XRAY 1.800 0.19 1.00

1VPSA 289 XRAY 1.900 0.18 0.20

1P4CA 380 XRAY 1.350 0.18 0.19

2ARCA 164 XRAY 1.500 0.18 0.23

1OR7C 90 XRAY 2.000 0.20 0.23

1FE6A 52 XRAY 1.800 0.20 0.22

1JUHA 350 XRAY 1.600 0.16 0.19

16PK0 415 XRAY 1.600 0.19 0.23

1QZMA 94 XRAY 1.900 0.18 0.28

1WDCA 64 XRAY 2.000 0.19 0.28

1LWBA 122 XRAY 1.050 0.10 0.14

1QVEA 126 XRAY 1.540 0.14 0.18

1CXQA 162 XRAY 1.020 0.13 0.16

1GK9A 260 XRAY 1.300 0.15 0.17

1UMGA 362 XRAY 1.800 0.18 0.20

1VKMA 297 XRAY 1.900 0.16 0.20

1QZ9A 416 XRAY 1.850 0.15 0.19

1DQSA 393 XRAY 1.800 0.17 0.22

1P9GA 41 XRAY 0.840 0.07 0.08

1FJLA 81 XRAY 2.000 0.20 1.00

1QOPA 268 XRAY 1.400 0.15 0.18

1UW1A 80 XRAY 1.940 0.20 0.23

1I71A 83 XRAY 1.450 0.17 0.20

1QOYA 318 XRAY 2.000 0.20 0.25

1UJ6A 227 XRAY 1.740 0.20 0.21

1E1HA 287 XRAY 1.800 0.20 0.24

1FJSL 52 XRAY 1.920 0.20 0.26

1JIXA 351 XRAY 1.650 0.20 0.21

1T0BA 252 XRAY 1.700 0.14 0.17

1R7AA 504 XRAY 1.770 0.16 0.20

1NNHA 294 XRAY 1.650 0.17 0.19

1GTKA 313 XRAY 1.660 0.20 0.25

1VD5A 377 XRAY 1.800 0.17 0.19

1XJUA 163 XRAY 1.070 0.13 0.15

3PCGA 200 XRAY 1.960 0.17 1.00

1XOCA 520 XRAY 1.550 0.18 0.21

1OAPA 109 XRAY 1.930 0.20 0.23

1OXXK 353 XRAY 1.450 0.17 0.21

1R6DA 337 XRAY 1.350 0.18 0.22

1R3DA 264 XRAY 1.900 0.18 0.22

1R89A 437 XRAY 1.800 0.19 0.23

1EX0A 731 XRAY 2.000 0.19 0.23

1VPDA 299 XRAY 1.650 0.12 0.14

1OJXA 263 XRAY 1.900 0.15 0.18

1MF7A 194 XRAY 1.250 0.17 0.22

1RA0A 430 XRAY 1.120 0.17 0.17

1UWWA 191 XRAY 1.400 0.19 0.21

1J1NA 492 XRAY 1.600 0.19 0.21

2CB5A 453 XRAY 1.850 0.18 0.21

1KL9A 182 XRAY 1.900 0.20 0.23

1S3CA 141 XRAY 1.250 0.15 0.24

2AYH0 214 XRAY 1.600 0.14 1.00

2A0B0 125 XRAY 1.570 0.19 0.24

1MHNA 59 XRAY 1.800 0.15 0.19

1TQJA 230 XRAY 1.600 0.17 0.22

1WDDS 128 XRAY 1.350 0.16 0.18

1O7NB 194 XRAY 1.400 0.19 0.20

1QREA 247 XRAY 1.460 0.19 0.21

1VKYA 347 XRAY 2.000 0.18 0.21

1S7ZA 117 XRAY 1.830 0.17 0.24

1QTNA 164 XRAY 1.200 0.17 0.19

1VC4A 254 XRAY 1.800 0.19 0.22

1W9AA 147 XRAY 1.800 0.14 0.17

1BUPA 386 XRAY 1.700 0.19 0.22

1E6CA 173 XRAY 1.800 0.19 0.23

1Y4WA 518 XRAY 1.550 0.17 0.20

1AJSA 412 XRAY 1.600 0.17 1.00

1WHO0 96 XRAY 1.900 0.18 1.00

1KNGA 156 XRAY 1.140 0.12 0.15

1PVMA 184 XRAY 1.500 0.19 0.23

1LK9A 448 XRAY 1.530 0.19 0.22

1ARU0 344 XRAY 1.600 0.18 1.00

1G6XA 58 XRAY 0.860 0.11 0.14

1Q8DA 108 XRAY 1.800 0.19 0.21

1NUYA 337 XRAY 1.300 0.16 0.21

1T82A 155 XRAY 1.700 0.19 0.22

1DXRM 323 XRAY 2.000 0.19 0.22

1WPCA 485 XRAY 1.900 0.17 0.21

1KQFA 1015 XRAY 1.600 0.18 0.20

1LF2A 331 XRAY 1.800 0.20 0.26

1OI2A 366 XRAY 1.750 0.17 0.20

1NS5A 155 XRAY 1.680 0.14 0.21

1XY7A 166 XRAY 1.800 0.18 0.23

1Q2OA 416 XRAY 1.740 0.19 0.22

1CNV0 299 XRAY 1.650 0.17 1.00

1GVEA 327 XRAY 1.380 0.16 0.18

1FCQA 350 XRAY 1.600 0.19 0.23

1R6JA 82 XRAY 0.730 0.07 0.09

1AVWB 177 XRAY 1.750 0.19 0.21

1QXYA 252 XRAY 1.040 0.14 0.17

1Y08A 323 XRAY 1.930 0.20 0.23

1SFDA 105 XRAY 0.990 0.12 0.15

1TT8A 164 XRAY 1.000 0.12 0.15

1EVXA 162 XRAY 2.000 0.19 0.23

1R0RI 51 XRAY 1.100 0.16 0.18

1YQGA 263 XRAY 1.900 0.20 0.24

1A8D0 452 XRAY 1.570 0.19 0.26

1YGE0 839 XRAY 1.400 0.20 0.24

1XD3A 230 XRAY 1.450 0.18 0.19

1QWYA 291 XRAY 1.300 0.16 0.18

1JHGA 101 XRAY 1.300 0.13 0.17

2BJIA 277 XRAY 1.240 0.15 0.19

2SAK0 121 XRAY 1.800 0.18 0.24

1SBXA 106 XRAY 1.650 0.18 0.21

1F60B 94 XRAY 1.670 0.19 0.22

1AOCA 175 XRAY 2.000 0.19 0.28

1UX6A 350 XRAY 1.900 0.20 0.23

1H4AX 173 XRAY 1.150 0.16 0.18

2LISA 136 XRAY 1.350 0.14 0.18

1LM4A 194 XRAY 1.450 0.17 0.20

1SBZA 197 XRAY 2.000 0.18 0.22

1H70A 255 XRAY 1.800 0.19 0.22

1G3MA 294 XRAY 1.700 0.19 0.22

1G61A 228 XRAY 1.300 0.13 0.18

1UYLA 236 XRAY 1.400 0.20 0.23

1ONWA 390 XRAY 1.650 0.18 0.22

1B2PA 119 XRAY 1.700 0.19 0.21

1N1FA 159 XRAY 1.950 0.17 0.24

1O8XA 146 XRAY 1.300 0.18 0.21

1R5MA 425 XRAY 1.550 0.19 0.20

1M1ZA 513 XRAY 1.950 0.20 0.23

1RQWA 207 XRAY 1.050 0.13 0.15

1KWGA 645 XRAY 1.600 0.17 0.18

1GU7A 364 XRAY 1.700 0.17 0.19

1J27A 102 XRAY 1.700 0.18 0.22

2BAA0 243 XRAY 1.800 0.18 1.00

1RY9A 145 XRAY 1.820 0.18 0.21

1LKI0 180 XRAY 2.000 0.19 1.00

1PSRA 100 XRAY 1.050 0.11 0.14

1IOMA 377 XRAY 1.500 0.17 0.18

1VLRA 350 XRAY 1.830 0.16 0.20

1M1NA 491 XRAY 1.160 0.12 0.15

1P5ZB 263 XRAY 1.600 0.17 0.20

1UALA 274 XRAY 1.800 0.18 0.21

1C3CA 429 XRAY 1.800 0.18 0.21

1R4PA 297 XRAY 1.770 0.15 0.18

1L7AA 318 XRAY 1.500 0.19 0.19

1TG5A 424 XRAY 1.900 0.19 0.23

1E58A 249 XRAY 1.250 0.12 0.17

1GPPA 237 XRAY 1.350 0.15 0.19

1T2DA 322 XRAY 1.100 0.14 0.15

1R2MA 71 XRAY 1.000 0.14 0.15

1P5UB 149 XRAY 1.990 0.17 0.22

1BX70 55 XRAY 1.200 0.18 0.23

1KM4A 247 XRAY 1.500 0.15 0.18

1EEXG 173 XRAY 1.700 0.16 0.23

1Q40B 219 XRAY 1.950 0.18 0.22

1CF3A 583 XRAY 1.900 0.19 0.24

1K5CA 335 XRAY 0.960 0.11 0.14

1UQ5A 263 XRAY 1.400 0.18 0.21

1Q08A 99 XRAY 1.900 0.18 0.21

1RXEA 131 XRAY 1.700 0.20 0.21

1P5FA 189 XRAY 1.100 0.14 0.16

1K75A 434 XRAY 1.750 0.19 0.23

1LOUA 101 XRAY 1.950 0.20 0.25

1FD3A 41 XRAY 1.350 0.16 0.24

1MIXA 206 XRAY 1.750 0.20 0.24

1MGTA 174 XRAY 1.800 0.17 0.22

1KGDA 180 XRAY 1.310 0.19 0.21

1HBKA 94 XRAY 2.000 0.20 0.24

1MDL0 359 XRAY 1.850 0.18 1.00

1GU2A 124 XRAY 1.190 0.15 0.19

1NKZB 41 XRAY 2.000 0.17 0.19

1SW5A 275 XRAY 1.800 0.19 0.21

2ABK0 211 XRAY 1.850 0.18 0.22

1NPSA 88 XRAY 1.800 0.20 0.23

1RKIA 102 XRAY 1.600 0.19 0.23

1ANF0 370 XRAY 1.670 0.18 1.00

1YWMA 200 XRAY 1.860 0.19 0.23

1NFP0 228 XRAY 1.600 0.17 1.00

1PBYA 489 XRAY 1.700 0.19 0.22

1CY5A 97 XRAY 1.300 0.17 0.20

1KS8A 433 XRAY 1.400 0.18 0.19

1BYRA 155 XRAY 2.000 0.19 0.25

1YKDA 398 XRAY 1.900 0.18 0.21

1JYHA 157 XRAY 1.800 0.20 0.22

1DF4A 68 XRAY 1.450 0.20 0.24

1JZTA 246 XRAY 1.940 0.20 0.24

1KMTA 141 XRAY 1.300 0.16 0.20

1B5QA 472 XRAY 1.900 0.19 0.23

1VZYA 291 XRAY 1.970 0.20 0.23

1RHS0 296 XRAY 1.360 0.17 0.23

1CS1A 386 XRAY 1.500 0.20 0.26

1RWJA 82 XRAY 1.700 0.20 0.22

1HS6A 611 XRAY 1.950 0.18 0.25

1DEKA 241 XRAY 2.000 0.19 1.00

1WDDA 477 XRAY 1.350 0.16 0.18

1K30A 368 XRAY 1.900 0.19 0.22

1L3KA 196 XRAY 1.100 0.16 0.19

1SMXA 96 XRAY 1.800 0.20 0.23

1N13A 52 XRAY 1.400 0.20 0.21

1VP8A 201 XRAY 1.300 0.15 0.17

1RWHA 757 XRAY 1.250 0.11 0.14

2BKQA 212 XRAY 2.000 0.20 0.25

1PA7A 130 XRAY 1.450 0.17 0.19

1O6VA 466 XRAY 1.500 0.15 0.18

1RI6A 343 XRAY 2.000 0.17 0.20

1DFMA 223 XRAY 1.500 0.19 0.22

1UKUA 102 XRAY 1.450 0.17 0.19

1EZWA 349 XRAY 1.650 0.20 0.21

1SENA 164 XRAY 1.200 0.16 0.18

1J8BA 112 XRAY 1.750 0.18 0.26

1JO0A 98 XRAY 1.370 0.14 0.22

1N3LA 372 XRAY 1.180 0.18 0.22

1JDW0 423 XRAY 1.900 0.20 0.23

1NPYA 271 XRAY 1.750 0.18 0.22

1GARA 212 XRAY 1.960 0.17 0.29

1T4FM 110 XRAY 1.900 0.20 0.23

1DCS0 311 XRAY 1.300 0.13 0.15

1YOCA 147 XRAY 1.700 0.17 0.20

1JTGB 165 XRAY 1.730 0.17 0.20

1X9DA 538 XRAY 1.410 0.15 0.16

1TWYA 290 XRAY 1.650 0.19 0.23

1NZYA 269 XRAY 1.800 0.19 1.00

1IRDB 146 XRAY 1.250 0.16 0.21

1TJXA 159 XRAY 1.040 0.17 0.18

1FIUA 286 XRAY 1.600 0.17 0.20

1SGWA 214 XRAY 1.700 0.20 0.22

1DBXA 158 XRAY 1.800 0.18 0.26

1GQ8A 319 XRAY 1.750 0.18 0.19

1QQP3 220 XRAY 1.900 0.16 1.00

1JB9A 316 XRAY 1.700 0.17 0.23

1QNAA 200 XRAY 1.800 0.19 0.24

1EY4A 149 XRAY 1.600 0.19 0.26

1VJOA 393 XRAY 1.700 0.15 0.20

1FM0E 150 XRAY 1.450 0.15 0.18

1CCWA 137 XRAY 1.600 0.14 0.17

2PTD0 298 XRAY 2.000 0.20 0.27

1US5A 314 XRAY 1.500 0.18 0.20

1GCI0 269 XRAY 0.780 0.10 0.10

1PCFA 66 XRAY 1.740 0.20 0.23

1RLIA 184 XRAY 1.800 0.20 0.23

1ESGA 213 XRAY 1.900 0.19 0.23

1P1XA 260 XRAY 0.990 0.14 0.17

1OWLA 484 XRAY 1.800 0.19 0.21

2IGD0 61 XRAY 1.100 0.10 0.12

3LZT0 129 XRAY 0.930 0.09 0.11

1EJDA 419 XRAY 1.550 0.18 0.21

1LSHB 319 XRAY 1.900 0.19 0.26

1Y7BA 542 XRAY 1.600 0.16 0.21

1QWOA 442 XRAY 1.500 0.17 0.19

1VLYA 338 XRAY 1.300 0.14 0.17

1GQIA 708 XRAY 1.480 0.13 0.17

1JV1A 505 XRAY 1.900 0.18 0.22

1V73A 342 XRAY 1.820 0.18 0.23

1UIUA 502 XRAY 1.850 0.20 0.25

1PU6A 218 XRAY 1.640 0.15 0.18

1GBS0 185 XRAY 1.500 0.18 1.00

1J2JB 45 XRAY 1.600 0.20 0.23

1RGZA 363 XRAY 1.370 0.14 0.20

1J31A 262 XRAY 1.600 0.17 0.19

1U5KA 244 XRAY 2.000 0.20 0.21

1KA1A 357 XRAY 1.300 0.13 0.17

1KWFA 363 XRAY 0.940 0.09 0.11

1MTPA 323 XRAY 1.500 0.20 0.22

1K07A 263 XRAY 1.650 0.17 0.20

1GNYA 153 XRAY 1.630 0.15 0.18

1VYRA 364 XRAY 0.900 0.12 0.14

1MMIA 366 XRAY 1.850 0.19 0.24

1K2XB 143 XRAY 1.650 0.16 0.20

1JRLA 190 XRAY 1.950 0.20 0.23

1XUBA 298 XRAY 1.300 0.14 0.16

1A3C0 181 XRAY 1.600 0.19 0.23

1MOQ0 368 XRAY 1.570 0.18 1.00

1VGGA 161 XRAY 1.750 0.18 0.20

1YC9A 442 XRAY 1.800 0.19 0.22

1RMWA 212 XRAY 1.250 0.14 0.17

1NKIA 135 XRAY 0.950 0.15 0.18

1M9ZA 111 XRAY 1.050 0.16 0.17

1BY20 119 XRAY 2.000 0.19 0.22

1H32A 261 XRAY 1.500 0.20 0.23

1FDR0 248 XRAY 1.700 0.18 0.25

1GL2D 65 XRAY 1.900 0.18 0.22

1MUN0 225 XRAY 1.200 0.12 0.17

1QDDA 144 XRAY 1.300 0.13 0.16

1WD3A 482 XRAY 1.750 0.19 0.21

1EUR0 365 XRAY 1.820 0.17 1.00

1G2RA 100 XRAY 1.350 0.16 0.18

1V6PA 62 XRAY 0.870 0.12 0.15

1EX2A 189 XRAY 1.850 0.20 0.22

1NF9A 207 XRAY 1.500 0.16 0.23

1AKO0 268 XRAY 1.700 0.17 0.20

1KPEA 126 XRAY 1.800 0.20 0.22

1VH5A 148 XRAY 1.340 0.19 0.23

1J5WA 298 XRAY 1.950 0.20 0.25

1MOGA 67 XRAY 1.700 0.18 0.21

1T6CA 315 XRAY 1.530 0.16 0.22

1Q7FA 286 XRAY 1.950 0.20 0.25

1YD0A 96 XRAY 1.500 0.17 0.18

1AYL0 541 XRAY 1.800 0.20 0.23

1IQZA 81 XRAY 0.920 0.10 0.11

1QD1A 325 XRAY 1.700 0.19 0.21

1GQZA 274 XRAY 1.750 0.17 0.20

1N55A 251 XRAY 0.830 0.10 0.11

1AHO0 64 XRAY 0.960 0.16 1.00

1HZTA 190 XRAY 1.450 0.19 0.22

1U5PA 216 XRAY 2.000 0.19 0.23

1SEQH 225 XRAY 1.780 0.19 0.23

1DANT 80 XRAY 2.000 0.19 0.22

1A12A 413 XRAY 1.700 0.19 0.22

3TDT0 274 XRAY 2.000 0.15 0.22

1NTYA 311 XRAY 1.700 0.20 0.21

1XM8A 254 XRAY 1.740 0.14 0.19

1T7RA 269 XRAY 1.400 0.20 0.20

1TQMA 282 XRAY 1.990 0.17 0.23

1EQCA 394 XRAY 1.850 0.16 0.19

1CVRA 435 XRAY 2.000 0.16 0.21

1QSGA 265 XRAY 1.750 0.17 0.21

1WOJA 214 XRAY 1.800 0.19 0.23

1GKPA 458 XRAY 1.290 0.15 0.18

1N2EA 300 XRAY 1.600 0.19 0.21

1Q8IA 783 XRAY 2.000 0.20 0.24

1TDZA 272 XRAY 1.800 0.18 0.21

1OGQA 313 XRAY 1.700 0.19 0.24

1LKEA 184 XRAY 1.900 0.19 0.25

2PTH0 193 XRAY 1.200 0.20 0.21

1D1QA 161 XRAY 1.700 0.17 0.21

1V74A 107 XRAY 2.000 0.19 0.23

1Q6OA 216 XRAY 1.200 0.14 0.16

1RJOA 646 XRAY 1.670 0.16 0.18

1JZ8A 1023 XRAY 1.500 0.17 0.21

1WU9A 80 XRAY 1.540 0.18 0.22

1LM8V 160 XRAY 1.850 0.20 0.24

1UOYA 64 XRAY 1.500 0.16 0.18

1I0VA 104 XRAY 1.230 0.18 0.19

1WM3A 72 XRAY 1.200 0.12 0.18

1KICA 339 XRAY 1.600 0.18 0.19

1YLLA 200 XRAY 1.640 0.20 0.24

2BBKL 125 XRAY 1.750 0.17 1.00

1KV7A 488 XRAY 1.400 0.18 0.22

1DXRH 258 XRAY 2.000 0.19 0.22

1SVFA 64 XRAY 1.400 0.18 0.20

1VPJA 192 XRAY 1.690 0.18 0.21

1D4TA 104 XRAY 1.100 0.13 0.17

1FI2A 201 XRAY 1.600 0.19 0.23

1QMGA 524 XRAY 1.600 0.20 0.22

1JIWI 106 XRAY 1.740 0.18 0.20

1G5HA 454 XRAY 1.950 0.18 0.22

1V58A 241 XRAY 1.700 0.19 0.21

1XKIA 162 XRAY 1.800 0.19 0.25

1H16A 759 XRAY 1.530 0.14 0.16

1OH4A 179 XRAY 1.350 0.14 0.17

3SIL0 379 XRAY 1.050 0.12 0.15

1N62C 288 XRAY 1.090 0.14 0.17

1ODZA 386 XRAY 1.400 0.15 0.18

1XGKA 352 XRAY 1.400 0.19 0.24

1FKMA 396 XRAY 1.900 0.20 0.23

1QO2A 241 XRAY 1.850 0.19 0.25

1Q5ZA 177 XRAY 1.800 0.19 0.22

1KVEB 77 XRAY 1.800 0.17 1.00

1TW6A 133 XRAY 1.710 0.16 0.17

1RMG0 422 XRAY 2.000 0.17 0.21

1SYYA 346 XRAY 1.700 0.15 0.20

1EWFA 456 XRAY 1.700 0.20 0.25

1P5DX 463 XRAY 1.600 0.16 0.18

1MSC0 129 XRAY 2.000 0.20 1.00

1I4UA 181 XRAY 1.150 0.15 0.19

1GVDA 52 XRAY 1.450 0.18 0.20

1L6PA 125 XRAY 1.650 0.14 0.23

1LS1A 295 XRAY 1.100 0.14 0.17

1OF1A 376 XRAY 1.950 0.18 0.22

1URQC 80 XRAY 2.000 0.17 0.23

1OS6A 71 XRAY 1.450 0.18 0.23

1NVMB 312 XRAY 1.700 0.19 0.23

1WDVA 152 XRAY 1.700 0.17 0.21

2MCM0 112 XRAY 1.500 0.15 1.00

1PBJA 125 XRAY 1.400 0.19 0.20

1UCDA 190 XRAY 1.300 0.20 0.20

7HBIA 146 XRAY 1.600 0.19 0.24

1Y42X 392 XRAY 1.950 0.18 0.23

1TCA0 317 XRAY 1.550 0.16 1.00

1USEA 45 XRAY 1.300 0.17 0.20

1UAIA 224 XRAY 1.200 0.17 0.19

1YOYA 175 XRAY 2.000 0.19 0.24

1NOFA 383 XRAY 1.420 0.11 0.16

1YLKA 172 XRAY 2.000 0.18 0.23

1VSRA 136 XRAY 1.800 0.20 0.22

1HYOA 421 XRAY 1.300 0.18 0.20

1GYXA 76 XRAY 1.350 0.14 0.16

1M1QA 91 XRAY 0.970 0.14 0.15

1NAR0 290 XRAY 1.800 0.16 1.00

1QTWA 285 XRAY 1.020 0.12 0.15

1VYBA 238 XRAY 1.800 0.19 0.22

1RL0A 255 XRAY 1.400 0.18 0.21

1DIN0 236 XRAY 1.800 0.15 1.00

1Q74A 303 XRAY 1.700 0.20 0.23

1U7IA 136 XRAY 1.400 0.15 0.18

1DLWA 116 XRAY 1.540 0.13 0.18

1PB7A 292 XRAY 1.350 0.19 0.22

1Q7LA 198 XRAY 1.400 0.13 0.17

1VKFA 188 XRAY 1.650 0.19 0.21

1Q9BA 43 XRAY 1.500 0.13 0.14

1T9IA 163 XRAY 1.600 0.19 0.21

1QJPA 171 XRAY 1.650 0.15 0.20

1YGHA 164 XRAY 1.900 0.20 0.24

1GVFA 286 XRAY 1.450 0.13 0.17

1JK3A 158 XRAY 1.090 0.17 0.20

1I40A 175 XRAY 1.100 0.12 0.15

1O2DA 371 XRAY 1.300 0.14 0.17

1P90A 145 XRAY 1.800 0.19 0.26

1US0A 316 XRAY 0.660 0.09 0.10

1EW4A 106 XRAY 1.400 0.19 0.21

1L8AA 886 XRAY 1.850 0.20 0.24

1Y80A 210 XRAY 1.700 0.18 0.21

1T8KA 77 XRAY 1.100 0.13 0.15

1QGIA 259 XRAY 1.600 0.19 0.23

2KINA 238 XRAY 2.000 0.19 0.25

1PFBA 55 XRAY 1.400 0.20 0.22

1PX0A 254 XRAY 1.900 0.18 0.21

1ODMA 331 XRAY 1.150 0.14 0.15

1OGDA 131 XRAY 1.950 0.20 0.21

1C3PA 375 XRAY 1.800 0.20 0.24

1XSVA 113 XRAY 1.700 0.19 0.22

1FVKA 189 XRAY 1.700 0.20 0.23

1E1HB 174 XRAY 1.800 0.20 0.24

1S95A 333 XRAY 1.600 0.17 0.21

1CDCA 99 XRAY 2.000 0.19 1.00

1A8I0 842 XRAY 1.780 0.18 0.23

1J2LA 70 XRAY 1.700 0.19 0.24

1I0SA 169 XRAY 1.650 0.19 0.21

1XU9A 286 XRAY 1.550 0.16 0.18

1MKKA 96 XRAY 1.320 0.17 0.20

1I2TA 61 XRAY 1.040 0.15 0.17

1JBMA 86 XRAY 1.850 0.20 0.24

1TP6A 128 XRAY 1.500 0.20 0.23

1VQB0 87 XRAY 1.800 0.20 1.00

1YLEA 342 XRAY 1.700 0.19 0.22

1NXUA 333 XRAY 1.800 0.20 0.23

1Y66A 52 XRAY 1.650 0.19 0.23

1BQCA 302 XRAY 1.500 0.12 0.18

1EL5A 389 XRAY 1.800 0.17 0.21

1C5EA 95 XRAY 1.100 0.10 0.13

1GD0A 122 XRAY 1.500 0.19 0.21

1WC1A 226 XRAY 1.930 0.20 0.23

1R5YA 386 XRAY 1.200 0.17 0.20

1IFC0 132 XRAY 1.190 0.17 1.00

1RCQA 357 XRAY 1.450 0.15 0.21

1D3YA 301 XRAY 2.000 0.20 0.24

1OB8A 135 XRAY 1.800 0.20 0.25

1VIOA 243 XRAY 1.590 0.20 0.22

1EEXB 224 XRAY 1.700 0.16 0.23

1NUUA 252 XRAY 1.900 0.20 0.24

1OE4A 247 XRAY 2.000 0.18 0.22

1QQP1 213 XRAY 1.900 0.16 1.00

1DANU 121 XRAY 2.000 0.19 0.22

1HQ1A 105 XRAY 1.520 0.16 0.20

1RUTX 188 XRAY 1.300 0.16 0.19

1OK0A 74 XRAY 0.930 0.10 0.13

1RQ2A 382 XRAY 1.860 0.19 0.22

1T0HA 132 XRAY 1.970 0.19 0.21

1VHH0 162 XRAY 1.700 0.19 0.26

1NRJA 158 XRAY 1.700 0.20 0.23

1XU1R 42 XRAY 1.900 0.17 0.20

1TR9A 341 XRAY 1.800 0.17 0.20

1YFQA 342 XRAY 1.100 0.15 0.19

1R5LA 262 XRAY 1.500 0.19 0.21

1JAKA 512 XRAY 1.750 0.18 0.19

1IULA 140 XRAY 2.000 0.20 0.27

1VR7A 142 XRAY 1.200 0.12 0.15

1OF8A 370 XRAY 1.500 0.13 0.17

1PG4A 652 XRAY 1.750 0.18 0.21

1E25A 282 XRAY 1.900 0.14 0.18

1JIDA 128 XRAY 1.800 0.19 0.22

1DYPA 271 XRAY 1.540 0.18 0.19

1YO3A 102 XRAY 1.650 0.18 0.21

1E42A 258 XRAY 1.700 0.20 0.25

1JUBA 311 XRAY 1.400 0.19 0.20

1VA0A 239 XRAY 1.970 0.20 0.23

1R8HA 87 XRAY 1.900 0.20 0.26

1BDMA 327 XRAY 1.800 0.17 1.00

1B8OA 284 XRAY 1.500 0.18 0.23

2TPSA 227 XRAY 1.250 0.18 0.22

2BK7B 222 XRAY 1.600 0.15 0.18

1LUQA 127 XRAY 0.960 0.14 0.17

2BEMA 170 XRAY 1.550 0.16 0.17

1HZ4A 373 XRAY 1.450 0.19 0.21

1FNLA 175 XRAY 1.800 0.18 0.22

1N62A 166 XRAY 1.090 0.14 0.17

1SDIA 213 XRAY 1.650 0.15 0.18

1MTYD 512 XRAY 1.700 0.18 1.00

1KYFA 247 XRAY 1.220 0.15 0.21

1ZIN0 217 XRAY 1.600 0.17 0.22

1E6UA 321 XRAY 1.450 0.14 0.17

1MC2A 122 XRAY 0.850 0.10 0.12

1XG4A 295 XRAY 1.600 0.18 0.20

1R0MA 375 XRAY 1.300 0.15 0.17

1OKSA 56 XRAY 1.800 0.20 0.24

1E30A 155 XRAY 1.500 0.18 0.22

1I2KA 269 XRAY 1.790 0.16 0.25

1POC0 134 XRAY 2.000 0.19 1.00

1Q0RA 298 XRAY 1.450 0.17 0.19

1L9LA 74 XRAY 0.920 0.14 0.19

1SX7A 164 XRAY 1.060 0.12 0.14

1PJCA 361 XRAY 2.000 0.20 0.26

2PVIA 157 XRAY 1.760 0.19 0.26

1UOLA 219 XRAY 1.900 0.19 0.23

1ZPDA 568 XRAY 1.860 0.16 0.20

1HFES 123 XRAY 1.600 0.16 0.18

1X6IA 91 XRAY 1.200 0.17 0.21

1SH8A 154 XRAY 1.500 0.20 0.22

1YIIA 320 XRAY 1.420 0.19 0.21

1MUSA 477 XRAY 1.900 0.19 0.25

1MKAA 171 XRAY 2.000 0.16 0.21

1LVK0 762 XRAY 1.900 0.20 1.00

1WU4A 396 XRAY 1.350 0.18 0.20

1Y7YA 74 XRAY 1.690 0.17 0.22

1YACA 208 XRAY 1.800 0.17 0.20

1HTRP 43 XRAY 1.620 0.18 1.00

1VR5A 547 XRAY 1.730 0.15 0.18

1QJ4A 257 XRAY 1.100 0.12 0.14

1X7YB 342 XRAY 1.570 0.15 0.17

1ABA0 87 XRAY 1.450 0.17 1.00

1SAUA 115 XRAY 1.120 0.11 0.14

1KSOA 101 XRAY 1.700 0.19 0.23

1JY3O 56 XRAY 1.600 0.19 0.22

1N1JA 93 XRAY 1.670 0.18 0.21

1HP1A 516 XRAY 1.700 0.18 0.20

2ILK0 160 XRAY 1.600 0.16 1.00

4UBPA 101 XRAY 1.550 0.15 0.19

1R0UA 148 XRAY 1.750 0.18 0.21

1LNIA 96 XRAY 1.000 0.12 1.00

1T2WA 145 XRAY 1.800 0.20 0.23

1K1EA 180 XRAY 1.670 0.18 0.23

1E7LA 157 XRAY 1.320 0.14 0.19

1G0SA 209 XRAY 1.900 0.19 0.24

1M2JA 249 XRAY 1.700 0.18 0.22

1CXLA 686 XRAY 1.810 0.15 0.19

1KOLA 398 XRAY 1.650 0.17 0.21

1IGQA 62 XRAY 1.700 0.20 0.23

2EBN0 289 XRAY 2.000 0.16 1.00

1GTVA 214 XRAY 1.550 0.19 0.21

1P7TA 731 XRAY 1.950 0.20 0.29

1V82A 253 XRAY 1.850 0.20 0.23

1TR0A 108 XRAY 1.800 0.16 0.20

1UZEA 589 XRAY 1.820 0.19 0.21

1NBUA 119 XRAY 1.600 0.18 0.26

1UZVA 114 XRAY 1.000 0.12 0.14

1QXMA 300 XRAY 1.700 0.18 0.20

1HM9A 468 XRAY 1.750 0.18 0.22

1CT5A 256 XRAY 2.000 0.20 0.24

1YVIA 149 XRAY 2.000 0.17 0.23

1O20A 427 XRAY 2.000 0.16 0.20

1OFZA 312 XRAY 1.500 0.14 0.18

1QKRA 188 XRAY 1.800 0.20 0.25

1MXRA 375 XRAY 1.420 0.16 0.18

1OOHA 126 XRAY 1.250 0.16 0.18

1H0HB 214 XRAY 1.800 0.17 0.20

3VUB0 101 XRAY 1.400 0.19 0.22

1UXAA 194 XRAY 1.500 0.18 0.20

1VMGA 95 XRAY 1.460 0.14 0.15

1UV4A 293 XRAY 1.500 0.17 0.20

1H1DA 221 XRAY 2.000 0.17 0.20

2ERL0 40 XRAY 1.000 0.13 0.17

1PJXA 314 XRAY 0.850 0.11 0.13

1O83A 70 XRAY 1.640 0.20 0.21

1N93X 375 XRAY 1.760 0.16 0.19

1U4GA 301 XRAY 1.400 0.18 0.20

1PXFA 111 XRAY 1.870 0.16 0.20

1FSGA 233 XRAY 1.050 0.12 0.15

1SSXA 198 XRAY 0.830 0.09 0.10

1B0NB 57 XRAY 1.900 0.20 0.24

1MN8A 100 XRAY 1.000 0.13 0.17

1OEJA 193 XRAY 1.810 0.18 0.25

1VQ3A 94 XRAY 1.900 0.18 0.23

1UCRA 78 XRAY 1.200 0.14 0.18

1L7LA 121 XRAY 1.500 0.19 0.20

1HXIA 121 XRAY 1.600 0.19 0.22

1U0FA 564 XRAY 1.600 0.18 0.21

1CI9A 392 XRAY 1.800 0.18 0.23

1QVZA 237 XRAY 1.850 0.19 0.21

1W3OA 216 XRAY 1.600 0.16 0.21

1W44A 331 XRAY 2.000 0.18 0.23

2PSPA 106 XRAY 1.900 0.20 0.26

1W6SB 74 XRAY 1.200 0.15 0.18

1TWIA 434 XRAY 2.000 0.16 0.20

1LQ9A 112 XRAY 1.300 0.14 0.17

2SCPA 174 XRAY 2.000 0.18 1.00

2UTGA 70 XRAY 1.640 0.19 1.00

1HZ6A 72 XRAY 1.700 0.19 0.22

1G6SA 427 XRAY 1.500 0.15 0.17

1LMIA 131 XRAY 1.500 0.20 0.25

1D5TA 433 XRAY 1.040 0.17 0.21

1NXMA 197 XRAY 1.300 0.14 0.17

1U02A 239 XRAY 1.920 0.20 0.22

1FS7A 485 XRAY 1.600 0.18 0.21

1WS8A 109 XRAY 1.600 0.19 0.21

1N8VA 112 XRAY 1.390 0.18 0.20

1ME4A 215 XRAY 1.200 0.10 0.12

1PMI0 440 XRAY 1.700 0.18 1.00

1HJSA 332 XRAY 1.870 0.19 0.21

1R4UA 301 XRAY 1.650 0.16 0.18

1COZA 129 XRAY 2.000 0.20 0.26

3SEB0 238 XRAY 1.480 0.18 0.17

1OW4A 129 XRAY 1.600 0.18 0.21

Table 6. List of individual turns and their characteristics

(a) π-HB turns

PDBID START END CA-CA DIHEDRAL ANGLES OF THE MIDDLE RESIDUES SEQ SSE CONFOR-

DIST (i-1) (i-1) MATION

-(i+6) -(i+6)

PHI1 PSI1 PHI2 PSI2 PHI3 PSI3 PHI4 PSI4

1a68_ 103 108 5.482 -67.60 -26.00 -69.10 -47.50 -96.50 -8.90 55.10 56.60 YDPLRNEY *** EETTTTEE AAAa

1a8d_ 296 301 5.421 -66.30 -30.50 -60.40 -41.10 -101.00 -3.00 56.70 51.50 TNGKLNIY *** EETTTTEE AAAa

1ajsA 162 167 5.217 -59.90 -30.70 -72.40 -63.10 -72.90 -12.50 55.70 53.40 WDTEKRGL *** EETTTTEE AAAa

1ako_ 214 219 5.375 -62.10 -65.00 -47.00 -46.30 -80.50 -2.60 57.80 41.80 FDYRSKGF *** TTTTHH AAAa

1amx_ 255 260 5.365 -64.30 -26.90 -75.90 -47.10 -80.40 -17.80 67.10 42.30 VDNTKNTI *** EETTTTEE AAAa

1aocA 113 118 5.075 -53.70 -45.80 -60.40 -46.30 -69.90 -22.80 102.40 14.50 QAPRAGFR *** BTTTTB AAAa

1aocA 153 158 5.238 -63.30 -44.80 -60.00 -66.60 -80.90 14.50 41.00 36.20 YNLEKDGF *** EETTTTEE AAAa

1aohA 74 79 5.198 -56.70 -37.70 -63.60 -44.90 -87.50 -2.30 59.80 44.60 VYPDRKMI *** EETTTTEE AAAa

1arb_ 109 114 5.566 -74.10 -31.00 -67.50 -46.70 -96.10 8.10 55.40 43.20 TYATSDFT *** EETTTTEE AAAa

1ayl_ 194 199 5.378 -81.50 -10.50 -82.20 -42.90 -90.00 -14.20 58.40 60.20 FNLTERMQ *** EETTTTEE AAAa

1ayl_ 475 480 5.289 -60.70 157.50 75.60 -60.40 -85.10 -16.60 54.00 42.60 TLPMFNLA *** EETTTTEE PdAa

1b25A 255 260 5.190 51.70 74.30 52.70 44.80 63.50 16.10 -55.80 -42.30 PTRNFSDG *** BTTTTBS rrgA

1bea_ 7 12 5.531 -54.60 136.20 102.40 -60.10 -68.50 -104.30 -81.20 -17.20 aVPGWAIP *** SBTTTB PdAA

1bea_ 100 105 4.784 -65.20 -16.90 -71.10 -37.40 -101.90 -108.20 -79.50 -18.20 VTEAEdNL *** TSTTTT AAAA

1bf2_ 547 552 4.652 -44.60 -58.60 -68.60 -34.90 -72.20 -12.10 86.20 0.20 QGMSAGTG *** TTTTTTS AAAa

1bqcA 165 170 5.472 59.40 47.60 57.20 39.30 79.90 -10.70 -90.70 -10.10 APNWGQDW *** TTTTT T rrgA

1brt_ 173 178 5.954 -64.40 125.60 98.10 -19.40 -120.10 -63.40 -97.60 -10.00 ENLGTRIS *** HHBTTTB PgAA

1bs0A 76 81 4.611 -61.50 -20.20 -96.20 -1.80 -120.60 -116.00 -102.20 10.90 GSGHVSGY *** SBTTTT AAAA

1byrA 9 14 5.165 -120.70 151.80 -80.50 10.90 -50.90 -41.80 117.20 -22.40 GYSPEGSA *** EEETTTHH BAAa

1c1dA 18 23 5.500 -56.70 -41.30 -57.70 -44.30 -94.20 -19.70 81.10 7.80 FDAMTGAH *** EETTTTEE AAAa

1c9oA 10 15 5.652 -62.80 -38.60 -63.20 -43.90 -83.50 -20.50 78.70 29.00 FNNEKGYG *** EETTTTEE AAAa

1cb8A 343 348 5.151 -63.90 -28.90 -87.10 -39.20 -82.30 -3.40 49.00 54.40 QFWNGDYV *** EETTTTEE AAAa

1cb8A 614 619 5.258 -57.30 -56.50 -59.10 -34.20 -92.20 -6.90 47.60 51.90 YHQQLDMV *** EETTTTEE AAAa

1cc8A 39 44 5.305 -69.90 -30.40 -71.40 -14.40 -123.80 -5.90 53.70 44.00 ISLEKQLV *** EETTTTEE AAAa

1ccwB 472 477 5.448 -64.80 -38.30 -61.60 -29.00 -91.00 2.60 110.80 9.60 FAVGKGRL *** HHTTTTSS AAAa

1cewI 102 107 5.102 -71.10 6.60 -102.90 0.00 -114.00 -4.40 61.50 41.30 SIPWLNQI *** EETTTTEE AAAa

1chd_ 342 347 6.088 -75.90 -178.50 65.60 -127.40 -104.30 17.30 -57.60 -45.70 ISAGQAIR *** HTTTTTB PpAA

1ci9A 58 63 5.065 -66.20 -46.40 -62.00 -34.30 -87.50 -7.30 67.30 32.30 ADREAGRP *** EETTTTEE AAAa

1cnv_ 55 60 5.888 -53.20 104.20 115.90 -17.60 -107.90 -5.00 60.00 30.80 cGPSVGNP *** BBTTTTB PgAa

1cnzA 290 295 6.457 -49.40 129.40 81.90 3.80 -99.80 1.90 58.80 35.70 DIAGKNIA *** GGTTTT S PgAa

1cpo_ 99 104 6.093 -63.40 153.00 50.40 42.30 -134.50 -108.40 -84.90 -41.10 AEPHAFEH *** G TTTTS PrAA

1cpo_ 289 294 6.739 -129.00 1.30 -134.00 74.30 -90.90 179.90 -78.20 155.60 YAQDPTLG *** BTTB ADBP

1cruA 122 127 5.474 -65.70 -33.10 -72.20 -34.60 -103.50 -7.60 54.90 57.60 YNKSTDTL *** EETTTTEE AAAa

1cs1A 196 201 5.693 -62.40 -16.70 -98.20 -115.40 -63.90 -38.00 -57.90 -47.80 SCTXYLNG *** ETTTTTT AXAA

1cseI 57 62 5.398 -65.30 143.50 87.10 -37.10 -86.90 -23.20 53.80 39.70 YNPGTNVV *** EETTTTEE PgAa

1cuj_ 152 157 4.658 -71.80 -43.70 -63.50 -39.40 -97.50 10.60 50.80 44.40 KNLQNRGR *** TTTTTTT AAAa

1czpA 9 14 5.224 -67.00 -43.90 -60.10 -38.70 -89.70 -12.70 60.40 48.90 INEAEGTK *** EETTTTEE AAAa

1d2nA 620 625 5.460 -62.10 -24.00 -74.70 107.70 130.10 -73.60 -79.40 177.10 YVPIGPRF *** BTTTTB APxP

1d7uA 213 218 5.274 -82.30 -59.60 -57.90 -33.30 -70.60 -9.40 118.40 30.80 ILSSGGII *** EETTTTSE AAAa

1d7uA 247 252 6.044 -70.80 164.10 61.30 52.00 76.00 -3.60 -58.20 -33.20 QTGVGRTG *** TTTTTTTS PrgA

1d7uA 368 373 5.421 -59.00 14.70 -134.70 -32.80 -88.70 -28.80 62.30 37.50 KDRRTKEP *** S TTTT B AAAa

1dc1A 204 209 5.575 -32.80 -51.50 -72.90 -41.40 -83.80 -21.50 59.40 41.40 TVSLVKKN *** EETTTTEE AAAa

1dljA 86 91 5.647 -62.70 -28.10 -68.60 -45.90 -89.60 1.40 54.80 44.50 YNSRINYF *** EETTTTEE AAAa

1dqaA 522 527 5.941 -64.70 134.30 72.20 12.50 -148.20 -39.00 -124.40 -19.90 LVMGACCE *** HHTTTT S PgAA

1dqaA 784 789 6.252 89.50 163.30 -47.40 -40.60 -102.60 2.70 58.20 39.10 ASGPTNED *** EETTTT E pAAa

1dqgA 10 15 5.654 -61.70 -47.10 -58.90 -44.00 -84.70 -15.30 54.50 48.00 YNEDHKRa *** EETTTTEE AAAa

1dqgA 49 54 5.505 -65.40 -41.90 -67.90 -32.10 -106.30 -16.30 54.40 41.90 MSVAFKLb *** EETTTTEE AAAa

1dqzA 15 20 5.500 -63.50 -41.30 -64.40 -30.90 -104.80 1.40 59.80 38.80 PSASMGRD *** EETTTTEE AAAa

1dqzA 84 89 5.636 -85.50 110.50 137.50 -44.70 -99.50 12.70 72.70 13.20 PSQSNGQN *** S TTTT PgAa

1duvG 82 87 5.544 -45.60 128.70 75.00 -49.70 -82.30 -42.10 -110.90 -13.20 SQIGHKES *** S BTTTB PgAA

1dxrH 174 179 5.143 -80.50 -14.70 -79.60 -35.30 -114.50 -6.10 61.50 63.60 VDRSEHYF *** EETTTTEE AAAa

1dxrM 284 289 6.321 -70.90 125.10 87.00 -43.40 -63.90 -54.00 -127.30 -11.70 LLTGTFVD *** HHBTTTBS PgAA

1dy5A 111 116 5.636 100.40 179.70 -134.80 104.00 -72.40 157.90 -90.10 106.40 cEGNPYVP *** EETTTTEE pBPB

1e30A 143 148 5.424 -67.70 -35.30 -64.20 -24.50 -105.40 18.50 106.80 3.90 GHAATGQF *** TTTTTT E AAAa

1e5pA 39 44 5.140 56.00 65.30 46.80 48.70 50.50 30.60 -78.10 -14.70 aYKNaSEX *** EETTTTEE rrrA

1e6bA 40 45 5.369 -68.10 -32.80 -63.00 -45.00 -75.40 -2.10 70.50 33.80 VNLLKGDQ *** TTTTGG AAAa

1edg_ 89 94 5.346 78.30 170.20 -69.00 -19.20 -94.30 3.70 50.70 48.80 VSGSDYKI *** EETTTTEE pAAa

1edg_ 356 361 5.421 -61.70 -22.80 -66.60 -50.50 -99.80 0.40 58.60 43.40 FDRRSCQF *** EETTTTEE AAAa

1eexB 70 75 5.744 -66.50 126.60 72.00 5.60 -116.70 -99.90 -66.90 -23.60 PAFGLAQT *** TTBTTTB PgAA

1eqcA 311 316 5.221 -65.40 -32.10 -57.10 -35.60 -105.30 22.40 63.50 40.10 GARYEGAY *** TTBT S AAAa

1euvA 521 526 5.304 -67.30 -34.00 -70.90 -40.70 -87.90 -5.30 57.00 52.30 IDLKKKTI *** EETTTTEE AAAa

1euwA 13 18 6.877 -66.30 125.10 84.10 -19.60 -95.60 -64.70 -94.10 -10.30 PRVGKEFP *** TTBTTTB PgAA

1evfA 169 174 6.344 -54.40 -40.40 -77.80 -51.80 -81.30 -46.80 -92.40 -33.60 XDVFLGLP *** EETTTTHH AAAA

1evxA 127 132 5.016 -64.70 -19.50 -113.90 3.90 -113.80 5.20 117.20 17.30 PGPNGGCV *** TTTT AAAa

1ew4A 54 59 5.081 -46.00 -56.50 -50.30 -42.60 -89.10 -5.50 54.80 43.70 RQEPLHQV *** EETTTTEE AAAa

1ewfA 122 127 5.435 -56.40 -30.20 -78.40 -47.40 -86.80 -15.80 71.10 18.10 SNPTSGKP *** EETTTTEE AAAa

1ezwA 277 282 4.893 -84.60 160.10 79.90 -23.50 -100.80 -9.40 79.40 7.00 EAIGKGDF *** HHTTTT H PgAa

1f60A 196 201 7.420 -58.30 128.80 60.10 22.70 -85.70 -65.40 -113.40 -33.50 NGDNMIEA *** T BTTTB PgAA

1f60A 278 283 5.290 -112.70 126.50 -102.70 -19.20 -63.80 -42.30 89.60 18.70 TFAPAGVT *** EEETTTEE BAAa

1f86A 18 23 5.482 -77.90 -11.40 -79.90 -44.30 -93.60 -19.90 69.70 45.50 LDAVRGSP *** EETTTTEE AAAa

1f8eA 306 311 5.294 -74.40 -7.10 -92.10 -51.10 -76.90 -16.40 56.80 54.80 IDPVAMTH *** EETTTTEE AAAa

1fcyA 280 285 5.435 -64.00 -33.30 -66.10 -26.10 -111.90 -1.30 50.10 50.30 YTPEQDTM *** EETTTTEE AAAa

1fjjA 55 60 4.694 -83.50 71.00 -122.00 -13.70 101.20 -18.10 -142.90 21.40 DAPTGSGW *** TSTTTT E DAgA

1fn9A 51 56 5.408 -83.20 0.80 -90.80 -19.10 -134.50 -9.10 62.40 33.40 VCMHCLGV *** EETTTTEE AAAa

1fp3A 219 224 5.078 56.40 61.10 46.30 47.40 68.00 22.60 -107.20 1.40 VQRDGQAV *** EETTTTEE rrgA

1fviA 178 183 7.609 -65.20 -43.90 -59.40 -38.70 -64.00 -34.40 55.40 31.90 STLKEGIL *** TTTTSS AAAa

1fvkA 4 9 7.072 -63.00 137.10 88.40 -12.90 -98.00 -96.40 -83.10 1.50 YEDGKQYT *** S BTTTEE PgAA

1g0sA 66 71 5.163 -68.10 -29.50 -74.50 -49.40 -84.70 -5.80 52.90 54.40 FDPVRDEV *** EETTTTEE AAAa

1g5aA 143 148 5.069 56.60 46.80 58.50 37.60 90.60 -6.40 -77.70 0.70 KSDGGYAV *** STTTTS rrgA

1g6xA 24 29 5.604 -74.60 -25.70 -66.90 -47.20 -74.90 -25.80 68.30 43.50 YNAKAGLc *** EETTTTEE AAAa

1g8mA 199 204 5.665 -60.00 129.80 79.30 -2.00 -110.60 -75.60 -112.10 3.60 YSKGVSQL *** H BTTTEE PgAA

1g9gA 378 383 5.065 62.20 14.20 68.70 18.80 -136.30 20.20 53.30 41.80 SWNGRYEA *** SBTTTTB ggAa

1g9gA 403 408 5.310 -125.10 -0.20 -130.10 79.10 -86.40 172.60 -55.20 128.20 VYADPGSN *** S BTTBTT ADPP

1gai_ 97 103 4.277 -48.50 -49.00 -97.40 2.80 -122.60 23.10 114.50 -8.00 GDLSSGGL *** B TTTTGG AAAa

1gai_ 263 268 5.559 -68.20 -38.10 -60.30 -29.80 -109.80 9.20 51.20 45.70 cDDSTFQP *** S TTTT T AAAa

1gbs_ 91 96 5.258 79.10 173.50 -49.30 -43.40 -90.60 0.70 52.70 57.60 GFGLMQVD *** EETTTTEE pAAa

1ggxA 36 41 5.472 -49.10 -32.10 -92.50 -5.00 -109.00 -9.60 79.50 27.70 GRPYEGHN *** EETTTTEE AAAa

1ggxA 134 139 4.880 -68.90 -53.20 -72.10 -23.10 -97.20 5.60 47.20 56.70 GPVMQKKT *** STTTTT E AAAa

1gk9B 130 135 5.216 -68.30 -23.90 -63.90 -47.00 -95.40 -2.10 54.40 56.50 TDQTTQTA *** EETTTTEE AAAa

1gkmA 52 57 6.243 -49.40 127.30 81.60 2.30 -110.50 -64.20 -132.10 22.20 TAYGINTG *** TTTTB PgAA

1gkmA 317 322 5.175 -63.40 -27.90 -66.20 -33.50 -100.20 -16.10 83.50 18.20 VFAAEGDV *** EETTTTEE AAAa

1gkmA 409 414 5.118 43.30 96.60 15.70 70.30 42.00 5.30 -68.80 -37.90 PTSANQED *** BTTTTB rrgA

1gkpA 155 160 5.115 -58.80 138.40 69.90 -3.30 -119.00 -55.70 -119.80 -24.10 SYKNFFGV *** STTTTB PgAA

1gnlA 252 257 6.120 -56.10 126.50 80.10 2.40 -92.80 -17.70 85.60 3.60 QTEGTGVD *** HHTTTT E PgAa

1gq8A 76 81 5.446 -73.80 -36.00 -58.70 -33.40 -91.70 -3.20 125.60 -6.20 KNVQDGST *** TTTT AAAa

1gqiA 392 397 4.684 -56.60 -38.30 -113.70 -0.20 -118.00 18.30 101.10 29.20 TQEYFGFA *** S TTTTTT AAAa

1gqiA 561 566 4.641 -55.10 -26.40 -89.90 -2.50 -122.40 -126.20 -76.70 -12.20 RTKTGSNA *** STTTT G AABA

1guqA 15 20 5.362 -64.20 -16.80 -77.40 -31.60 -109.60 -19.30 76.00 21.00 YNPLTGQW *** EETTTTEE AAAa

1gveA 14 19 5.751 -67.00 110.30 100.50 -4.30 -110.70 -98.60 -75.30 -14.60 MEMGRRMD *** TTBTTTB PgAA

1gwmA 79 84 5.250 -69.90 -29.60 -66.20 -43.00 -91.30 -7.80 52.00 46.90 ENSEADEK *** EETTTTEE AAAa

1gx5A 148 153 4.761 -51.10 -38.90 -57.90 -3.40 -139.90 -155.00 -63.30 14.40 VQPEKGGR *** BTTTTBS AABA

1gxqA 140 145 5.047 -58.60 -28.80 -77.00 -49.60 -96.40 -11.80 63.90 32.50 LDPTSHRV *** EETTTTEE AAAa

1h1nA 24 29 6.867 -53.90 139.60 81.00 -10.50 -116.60 -59.40 -94.40 -24.20 GVEGKDYI *** BTTTB PgAA

1h32A 98 103 6.012 -53.90 -47.10 -65.00 -18.80 -105.40 -17.60 57.80 27.20 YVESAGKV *** EETTTTEE AAAa

1h32A 170 175 5.326 78.30 174.10 -72.70 -21.70 -77.40 -15.40 63.70 56.20 RYGQLDLS *** BTTTTB pAAa

1h32A 206 211 6.220 -66.70 -23.60 -64.30 -45.80 -114.10 4.20 53.10 39.60 YRLKNARL *** EETTTTEE AAAa

1h4gA 88 93 5.149 -128.90 148.00 56.10 59.10 -87.40 155.40 -75.20 113.70 WTVDPLVE *** EEETTTEE BrPP

1h6lA 135 140 5.348 -77.70 -17.20 -73.30 -41.40 -98.80 -24.90 80.80 37.10 IDGKNGTL *** EETTTTEE AAAa

1h6lA 216 221 5.175 -62.40 -31.40 -82.90 -49.00 -83.60 -18.80 76.50 34.60 ADDEYGSL *** EETTTTEE AAAa

1h72C 155 160 4.913 -125.90 157.70 40.40 69.10 -90.20 155.80 -66.10 134.90 TNYEPLEV *** EETTTTEE BrBP

1hdhA 124 129 5.360 -61.50 -29.10 -85.10 -22.20 -115.70 36.60 112.80 -4.60 TPHARGFE *** STTTTT S AAAa

1hfuA 79 84 6.574 -55.80 137.20 88.70 -15.50 -119.10 -57.20 -101.80 -22.50 GADGVNQa *** BTTTB PgAA

1hjsA 140 145 4.850 41.10 61.90 -67.00 149.90 -89.40 -4.80 -53.80 -45.60 GLLWPTGR *** EETBTTEE rPAA

1hn0A 914 919 5.281 -79.00 -18.90 -70.10 -41.00 -106.00 -10.50 65.30 38.00 LDKLSNVT *** EETTTTEE AAAa

1hp1A 144 149 5.561 -60.80 -45.50 -69.90 -18.50 -122.40 -13.60 67.70 23.00 YQKSTGER *** EETTTTEE AAAa

1hs6A 28 33 4.867 -74.80 -16.30 -84.50 -39.30 -92.00 -5.30 52.30 53.90 VDFTRRTL *** EETTTTEE AAAa

1hs6A 135 140 4.663 -67.10 134.40 -85.70 -52.60 -124.70 53.30 -108.50 -5.60 QCQAIHCR *** TTTTGG PADA

1hx0A 51 56 5.342 -102.50 -14.00 -131.00 100.80 -76.20 179.50 -82.20 108.40 VVTNPSRP *** TTTTS ABPP

1hx0A 126 131 4.961 -65.30 -17.10 -76.50 -10.60 -86.90 -56.60 -96.10 148.70 EFPAVPYS *** BBTTTTB AAAB

1i0dA 158 163 5.505 54.70 -136.90 -107.10 22.30 -89.90 -16.90 78.80 -2.30 GIEDTGIR *** BTTTTB pAAa

1i0sA 36 41 5.264 -113.80 -22.20 -122.30 88.50 -85.00 177.60 -72.60 121.00 LTSKPVQI *** EETTTTEE ADPP

1i0vA 33 38 5.338 86.70 160.50 -67.50 -21.50 -92.80 5.80 52.90 68.10 TVGSNSYP *** BTTTTBS pAAa

1i71A 40 45 7.450 -57.70 -22.40 -111.30 18.70 -96.80 -4.70 60.20 38.90 YYPNGGLT *** T TTTT AAAa

1i7qA 185 190 5.013 -73.40 -21.90 -76.50 -38.10 -95.10 -12.40 64.70 45.70 LDHQRGSA *** EETTTTEE AAAa

1i7qA 315 320 5.355 -69.20 -16.20 -63.90 -52.00 -108.80 9.10 55.40 23.80 YDAGNRQI *** EETTTTEE AAAa

1i7qB 161 166 5.096 -61.60 -42.80 -56.80 -50.90 -92.00 -5.50 57.30 52.00 RDDRRRVC *** EETTTTEE AAAa

1iab_ 80 85 6.977 -50.40 -51.40 -108.70 103.60 142.00 -23.30 -134.40 -21.60 LQANGbVY *** E TTTT S ABxA

1iab_ 195 200 6.707 -67.10 -40.20 -69.90 -48.80 -101.50 93.40 -75.60 74.00 YTNEaSL# *** TTTTTT AABD

1icfI 228 233 5.683 -55.20 -35.10 -61.90 -42.00 -99.70 -11.10 77.20 49.50 hYGSIGYh *** EETTTTEE AAAa

1idpA 72 77 4.731 -64.40 -14.00 -79.00 -12.90 -118.40 -87.40 -119.90 -0.60 SSKQVLGD *** HSTTTT AAAA

1iomA 21 26 4.977 -59.60 -38.90 -60.80 -39.00 -101.60 0.80 69.70 42.80 IDGQQGKL *** EETTTTEE AAAa

1iomA 302 307 4.985 -57.10 -46.70 -61.00 -26.50 -86.60 -1.20 76.80 21.40 VLNPRGIY *** HHTTTT AAAa

1iow_ 50 55 6.351 -72.30 -44.40 -64.70 -11.10 -91.70 -11.20 98.20 7.50 QLKSMGFQ *** GTTTTTEE AAAa

1iqzA 35 40 4.628 -76.40 -18.50 -58.10 -29.40 -156.50 26.00 54.60 40.80 YVTLDDNQ *** EETTTTTS AAAa

1ituA 73 78 4.748 -67.60 133.10 67.30 20.60 -132.00 -107.00 -114.50 29.00 DTQNKDAV *** GGTTTTHH PgAA

1itwA 333 338 5.020 -60.50 -39.40 -60.40 -34.80 -96.80 2.10 67.90 25.70 VNSDKGIT *** EETTTTEE AAAa

1itxA 390 395 5.515 -81.70 -7.20 -86.80 -53.20 -75.60 -17.20 61.20 48.70 WNDTAKVP *** EETTTTEE AAAa

1iv8A 59 64 5.382 -75.30 -28.10 -65.40 -23.60 -89.20 -11.70 129.10 -9.20 INDELGGE *** E TTTTHH AAAa

1j1nA 349 354 6.381 -61.80 137.90 84.20 -8.10 -117.50 -55.80 -112.80 -22.60 GVPGVTYD *** BTTTEE PgAA

1j6oA 23 28 4.949 -58.10 -51.30 -58.00 -28.30 -95.80 12.70 63.90 28.70 SFEENNIE *** TTTTTTEE AAAa

1j7gA 66 71 5.339 -72.10 -46.90 -64.10 -32.70 -79.50 -3.10 61.30 43.70 NVQQAQGE *** TTTTT E AAAa

1jakA 388 393 6.837 -62.20 -24.30 -101.30 22.00 -137.50 -59.60 -111.30 9.60 SPADRTYL *** TTTT T AAAA

1jb3A 29 34 5.172 -59.60 -34.00 -71.10 -37.80 -105.80 4.20 60.40 47.90 VDPVHHTY *** EETTTTEE AAAa

1jb9A 100 105 5.354 78.20 173.20 -67.50 -16.90 -103.10 11.70 58.80 25.60 RYGDNFDG *** TTTTTTSS pAAa

1jdrA 58 63 5.550 -57.90 -25.20 -66.60 -37.90 -133.00 8.60 59.00 28.40 WDKHDNTG *** BTTTTBS AAAa

1jf4A 18 23 4.089 55.40 -137.50 -106.30 7.10 -120.20 15.40 53.70 41.20 IAGSDNGA *** HHTTTTTH pAAa

1ji1A 52 57 5.731 -68.80 -13.80 -66.40 -30.90 -135.80 12.60 46.90 53.70 WDTADNAF *** EETTTTEE AAAa

1ji1A 562 567 4.896 -75.70 -17.60 -73.30 -51.60 -98.80 0.80 59.00 45.00 TDDTNKIY *** EETTTTEE AAAa

1jidA 28 33 5.856 -62.90 -43.10 -59.50 -32.90 -78.40 -5.70 119.70 -8.80 KTIAEGRR *** S TTTT AAAa

1jm1A 127 132 5.753 90.10 178.30 -63.50 -24.70 -93.20 -11.40 73.10 20.90 GVGPNGTI *** BTTTT E pAAa

1jm1A 162 167 5.166 -63.40 -43.60 -61.60 -34.70 -100.70 -4.40 51.40 44.00 LYAKGADL *** EETTTTEE AAAa

1jnrA 45 50 4.889 -55.70 -28.10 -61.60 -25.90 -127.50 10.90 59.10 32.80 WAKLGGLK *** HHTTTT AAAa

1jnrA 622 627 5.314 -62.00 -39.00 -62.40 -47.80 -88.90 -12.00 57.10 48.30 YDAEKDEW *** EETTTTEE AAAa

1jq5A 312 317 4.981 -59.50 -39.50 -65.30 -27.50 -98.10 21.50 68.10 28.90 TLEDIKLK *** STTTTT T AAAa

1jr7A 128 133 5.629 -64.90 -26.30 -70.20 -31.70 -112.40 -19.20 77.90 15.40 FDAMSGQY *** BTTTTBS AAAa

1jrlA 74 79 5.232 -52.40 -55.50 -65.40 -19.00 -91.20 10.50 83.70 13.70 NDGLRGFQ *** TTTTTT AAAa

1ju2A 465 470 6.056 -67.60 131.30 95.40 -13.00 -119.20 -43.90 -122.00 -34.60 CLVGKVLD *** S BTTTB PgAA

1ju3A 339 344 5.453 -73.90 160.80 97.00 -68.30 -79.70 -15.30 49.60 57.20 FVMGIDEW *** EEBTTTEE PdAa

1jubA 202 207 5.522 -59.80 -32.20 -65.50 -50.00 -84.60 -8.30 54.60 41.30 IDPEAESV *** EETTTTEE AAAa

1jw9B 227 232 5.124 -68.90 -25.20 -62.10 -37.00 -113.30 6.80 50.00 52.90 YDAMTCQF *** EETTTTEE AAAa

1jy1A 526 531 4.991 57.50 62.40 41.10 56.50 67.00 19.80 -101.10 -12.00 LEKNGTQL *** EETTTTEE rrgA

1k12A 99 104 5.350 63.50 60.90 67.00 18.60 84.80 -12.20 -58.60 -29.60 LASNGVNN *** TTTTTTS rggA

1k1eA 15 20 6.564 -71.90 123.40 97.80 -13.40 -118.20 -62.90 -82.20 -10.60 DVDGVLTD *** E TTTTS PgAA

1k1eA 142 147 5.857 -55.90 145.60 72.90 -1.20 -114.20 20.80 103.50 19.60 THGGKGAF *** S TTTTHH PgAa

1k2xB 213 218 6.663 -55.80 129.30 87.90 -16.80 -97.60 -69.80 -102.40 -5.80 PLVGAGCY *** TSBTTTEE PgAA

1k3iA 177 182 5.711 -57.80 -36.60 -72.50 -26.40 -106.00 -25.10 79.60 9.40 IEPTSGRV *** EETTTTEE AAAa

1k3iA 303 308 5.555 -65.50 -27.80 -75.30 -46.00 -93.10 -8.30 53.70 54.80 YSPSSKTW *** EETTTTEE AAAa

1k3iA 390 395 5.054 -66.00 -31.20 -62.90 -40.30 -95.30 3.40 66.30 29.60 YDAVKGKI *** EETTTTEE AAAa

1k4iA 33 38 5.504 -59.00 -23.60 -85.90 -4.40 -106.90 -112.40 -85.40 -22.90 DDPSRENE *** TTTT AAAA

1k55A 152 157 6.087 -63.70 -30.50 -88.60 -8.90 -103.00 -45.90 -139.90 5.00 DKFWLEGQ *** TTTTTTSS AAAA

1k5nA 175 180 7.457 -63.20 -23.40 -62.40 -37.80 -110.40 -50.20 -62.20 -29.30 NGKETLQR *** HTTTTTT AAAA

1k6zA 1081 1086 5.590 -57.30 -41.80 -78.80 -42.50 -72.70 -19.60 67.20 40.30 WDEVGGHP *** EETTTTEE AAAa

1k8wA 285 290 5.072 24.50 -152.10 -76.40 -41.60 -59.60 -10.10 57.70 40.80 TEGENGKF *** EETTTTEE pAAa

1kgdA 778 783 7.937 -62.70 124.00 97.40 -0.10 -85.40 -68.50 -112.20 -21.60 EENGKNYY *** BTTTBE PgAA

1kl9A 35 40 4.721 -68.90 -20.40 -93.90 -11.10 -122.50 45.40 52.90 39.00 SLLEYNNI *** EETTTTTE AADa

1kl9A 76 81 5.132 -63.60 -33.80 -68.30 -53.20 -70.50 -28.50 98.10 10.10 VDKEKGYI *** EETTTTEE AAAa

1kp6A 45 50 5.327 -63.90 -20.80 -71.60 -45.00 -93.30 -16.70 70.90 34.90 YDARAGHd *** EETTTTEE AAAa

1kpgA 57 62 7.554 -73.80 -7.70 -98.30 -1.60 -97.10 1.00 55.60 44.00 ALGKLGLQ *** HHTTTT AAAa

1kpgA 177 182 5.608 -73.30 -37.50 -61.80 -47.00 -70.20 2.50 79.40 15.50 EIHERGLP *** HHTTTT S AAAa

1kq6A 59 64 5.009 -65.30 -53.50 -70.70 -14.80 -80.70 -9.80 76.50 16.80 FPIEAGAI *** TTTTTSS AAAa

1kqfA 327 332 5.711 -59.20 -19.80 -85.30 -36.30 -92.90 -14.40 51.00 54.60 YDAEKRQY *** EETTTTEE AAAa

1kqfC 135 140 5.264 -106.00 147.10 -85.60 -12.70 -62.40 -50.70 -115.60 -32.50 IWRPYFAQ *** T TTTTGG BAAA

1kqpA 169 174 5.728 -60.70 121.00 63.00 16.40 -104.10 -110.40 -66.40 -19.60 FTKYGDGG *** S TTTTT PgAA

1kv7A 175 180 5.380 -56.50 126.50 90.30 -2.50 -121.10 -72.30 -111.40 4.00 KQWGIDDV *** BTTTEE PgAA

1kwfA 141 146 4.760 -109.30 -14.90 -82.00 -15.50 -133.90 23.40 63.10 38.40 VTSHDGGD *** B TTTTTT AAAa

1kwgA 143 148 4.394 -55.40 149.00 66.70 31.30 58.00 51.80 -109.20 7.30 YGCHDTVR *** TTTTTTS PrrA

1kyfA 909 914 5.069 -69.80 -37.50 -75.60 -32.90 -84.70 -10.40 56.90 45.80 PNLQAQMY *** EETTTTEE AAAa

1lam_ 77 82 5.847 -55.50 -37.80 -79.60 -30.20 -97.50 -9.50 58.50 41.40 IDEQENWH *** EETTTTEE AAAa

1lc5A 215 220 4.496 -70.90 -51.80 -71.80 -36.30 -100.70 6.40 55.30 46.40 LTKFYAIP *** STTTTT T AAAa

1lf2A 22 27 5.354 96.90 148.80 -62.80 -12.40 -94.00 4.00 50.60 59.70 EVGDNQQP *** EETTTTEE pAAa

1lf2A 189 194 6.745 -89.80 67.80 -136.60 -88.70 -86.60 171.40 -89.30 74.20 NHDLYWQI *** EEETTEEE DAPD

1lf2A 314 319 5.435 -75.80 -39.30 -61.30 -40.80 -102.20 3.50 47.70 59.00 FDYDNHSV *** EETTTTEE AAAa

1lfwA 152 157 4.559 -63.00 -30.80 -74.70 -10.30 -126.50 -91.50 -110.20 13.60 TNEETNWV *** S TTTT H AAAA

1lfwA 313 318 5.256 -64.40 -48.00 -70.00 -28.80 -71.00 -22.50 95.20 2.20 YGKKLGIF *** TSTTTT AAAa

1lj8A 139 144 5.266 -63.80 -23.00 -66.90 -37.50 -122.80 2.80 70.40 15.20 IDDSNGEF *** EETTTTEE AAAa

1lk9A 350 355 5.662 -72.00 -16.70 -82.90 -55.40 -74.10 -20.30 64.10 43.60 YCNYFRRM *** EETTTTEE AAAa

1ll2A 225 230 5.446 -62.40 -20.70 -73.50 -63.40 -79.80 -7.10 53.60 54.60 YDTKTKSV *** EETTTTEE AAAa

1lmiA 17 22 5.267 -75.10 -17.50 -65.30 -40.80 -90.60 -2.00 57.10 39.70 TDTVGQVV *** EETTTTEE AAAa

1lml_ 449 454 5.031 -74.60 -29.40 -61.30 -30.30 -93.90 12.40 58.00 32.50 VSAFMDYd *** S TTTTT AAAa

1lml_ 516 521 5.446 -65.70 -23.90 -70.60 -21.80 -128.30 -0.10 52.80 46.40 hDTATRTY *** EETTTTEE AAAa

1lqtA 83 88 7.039 -74.50 130.20 103.30 -14.30 -118.80 -68.90 -75.40 -39.20 VVVGEHVQ *** BTTTB PgAA

1lqtA 335 340 5.486 -55.10 -45.40 -62.00 -46.00 -88.70 -17.90 64.30 33.70 FDDQSGTI *** BTTTTB AAAa

1lqvA 127 132 5.327 -72.40 -19.80 -54.90 -49.10 -115.60 9.30 58.40 54.20 FRPERALW *** EETTTTEE AAAa

1ls1A 106 111 7.415 -66.40 129.00 108.60 -16.40 -76.20 -19.00 84.60 13.30 GLQGSGKT *** TTTTHH PgAa

1lshA 633 638 5.607 -66.40 -32.50 -70.70 -42.50 -82.20 -18.50 54.30 39.30 FNARTMAG *** EETTTTEE AAAa

1lshA 903 908 5.569 -52.50 -40.30 -77.20 -33.80 -78.90 -2.30 51.30 44.30 MNARESNF *** EETTTTEE AAAa

1lshA 1017 1022 5.286 -79.50 -50.20 -47.80 -35.20 -109.60 -4.10 55.60 33.60 KSAALSSK *** EETTTTEE AAAa

1lvk_ 519 524 4.369 -99.50 -42.90 -149.90 116.60 -59.90 155.20 -71.60 143.70 DGRQPPGI *** H TTTT H ABPP

1lwdA 387 392 6.713 -53.70 138.70 64.00 0.80 -109.00 -52.30 -113.40 -20.30 VKLNEHFL *** BTTTB PgAA

1m0kA 192 197 4.786 -70.60 -18.60 -80.00 -21.00 -117.80 -113.80 -77.30 -15.30 IGSEGAGI *** HSTTTT AAAA

1m1nA 103 108 5.486 -66.40 130.60 89.70 -7.80 -119.30 -66.50 -117.30 -4.60 GTTGVNAF *** S BTTTB PgAA

1m1nB 511 516 5.409 -60.90 128.30 58.70 30.50 -138.80 -66.20 -117.40 -7.20 RGMQATDY *** TTTTGG PrAA

1m1zA 196 201 5.482 -54.80 -43.60 -55.30 -59.10 -86.30 -14.90 73.10 20.30 IDLGSGTA *** EETTTTEE AAAa

1m2dA 67 72 5.192 -112.80 159.40 -75.50 -0.90 -53.50 -35.80 77.40 29.10 VVYPDGVW *** EEETTTEE BAAa

1m2rA 58 63 5.123 -71.10 1.30 -89.00 -24.90 -146.90 11.80 65.80 18.40 INVNNNEK *** EETTTTEE AAAa

1m2rA 165 170 5.033 -63.80 -32.20 -64.20 -45.60 -90.40 -5.90 55.40 46.00 IDHELRKL *** EETTTTEE AAAa

1m2xA 118 123 6.782 -49.90 -31.40 -84.10 1.30 -127.90 -46.20 -106.70 -1.80 SHDDRAGG *** SSTTTTTT AAAA

1m2xA 205 216 5.235 -57.80 -42.80 -64.40 -45.50 -82.90 -14.90 62.00 44.60 WFPKEKVL *** EETTTTEE AAAa

1m55A 14 19 6.117 -63.60 -21.40 -92.00 -53.60 -77.40 -30.70 -113.80 -41.10 FDVEEHLP *** TTTT T AAAA

1m6sA 138 143 5.071 -69.70 -56.10 -54.70 -30.30 -79.50 -5.00 104.30 33.90 THNRSGGR *** SBTTTTSB AAAa

1m7gA 65 70 5.732 -79.40 -3.50 -115.20 7.40 -129.20 -100.70 -48.00 -46.10 NIRFGLNK *** HHTTTTTT AAAA

1m7gA 165 170 5.210 -49.80 126.40 92.30 -14.20 -121.50 -47.20 -139.20 17.70 EFTGISAP *** S BTTTB PgAA

1mdl_ 271 276 5.174 -74.70 -50.00 -73.20 -25.90 -75.10 -9.80 109.40 18.60 DAMKIGGV *** BTTTTTHH AAAa

1mhnA 117 122 5.284 -67.80 -35.90 -64.00 -36.00 -94.90 -5.70 60.00 38.70 IDFKRETC *** EETTTTEE AAAa

1mhnA 127 132 5.741 -61.80 110.60 83.20 6.30 -114.60 -2.10 67.00 17.50 VYTGYGNR *** EETTTTEE PgAa

1mkaA 46 51 5.826 81.50 171.80 -60.40 -17.80 -117.20 8.70 55.50 30.30 TGGNFDKG *** S TTTTS pAAa

1mml_ 174 179 5.319 -56.50 -30.50 -63.20 -38.40 -74.70 -19.30 83.60 22.00 RDPEMGIS *** EETTTTEE AAAa

1moq_ 362 367 6.600 -60.30 -18.00 -106.70 -0.40 -103.10 3.80 85.60 29.90 LSKELGYL *** HHTTTT S AAAa

1mpgA 53 58 5.189 -52.50 -54.20 -58.60 -43.10 -88.50 2.50 41.60 52.10 PDIARHTL *** EETTTTEE AAAa

1mtyG 118 123 5.050 -50.80 134.30 -50.60 145.90 -83.60 26.30 -78.70 -63.90 AYKPPIMP *** HHTTTTS PPAA

1n4wA 391 396 5.399 -63.50 -26.50 -61.90 -49.40 -97.00 -4.70 59.70 44.40 YDAATDRA *** EETTTTEE AAAa

1n4wA 453 458 6.629 -54.30 121.60 87.10 -6.80 -109.80 -69.30 -79.60 -20.10 CVLGKATD *** S TTTTB PgAA

1n62B 227 232 5.202 -78.20 -15.50 -68.50 -44.40 -93.50 -15.90 69.00 35.80 MDKIKGEL *** EETTTTEE AAAa

1n93X 283 288 5.073 -65.70 -45.80 -62.80 -35.00 -92.60 6.10 53.70 43.00 FLGAIRHP *** GTTTTT G AAAa

1n9eA 336 341 5.304 -66.00 -35.00 -73.40 -29.30 -109.90 2.30 45.60 51.60 YDGEEEYF *** EETTTTEE AAAa

1n9eA 410 415 7.308 -55.50 150.80 77.70 -34.30 -104.00 -53.70 -72.50 -26.20 LVPGYDcP *** BTTTB PgAA

1n9eA 738 743 5.144 -56.30 -30.30 -76.70 -66.40 -66.10 -28.10 70.60 31.30 YDDETEES *** EETTTTEE AAAa

1ndbA 114 119 5.480 -65.60 131.80 71.80 -53.40 -76.50 -18.60 -136.10 -50.50 PVVIYSSP *** SBTTTB PgAA

1ndbA 313 318 5.081 -59.60 -27.30 -92.00 -0.80 -113.30 -100.60 -126.10 8.10 GGSKFNSG *** S TTTTTT AAAA

1nh8A 38 43 5.112 -59.80 -36.20 -63.60 -45.10 -97.90 -5.00 56.70 52.40 IDPVNNVE *** EETTTTEE AAAa

1ni9A 309 314 5.211 -69.20 -30.50 -67.20 -44.10 -92.20 -8.50 57.60 58.10 IRGKSRTI *** EETTTTEE AAAa

1nlnA 61 66 5.144 -72.30 -17.40 -86.90 -36.20 -95.80 -2.90 53.90 46.60 WNPRSKTC *** EETTTTEE AAAa

1nls_ 55 60 6.891 -78.50 -12.70 -73.70 -53.10 -83.50 -26.80 56.40 52.80 YNSVDKRL *** EETTTTEE AAAa

1nnhA 209 214 5.178 -79.60 178.50 62.60 25.50 82.30 2.20 -69.90 -21.60 ILPYGYGE *** EETTTTEE PggA

1nqeA 88 93 4.843 -62.80 -49.60 -71.00 -50.10 -81.30 -13.40 65.40 38.60 LAGVSGSA *** GGTTTTS AAAa

1nqeA 447 452 5.633 -73.00 -17.40 -58.80 -45.90 -118.50 2.80 56.80 34.70 YDDHTLKY *** EETTTTEE AAAa

1nr0A 176 181 5.667 175.60 -166.20 -88.10 155.10 -71.70 149.30 -80.00 136.90 FEGPPFKF *** EETTTBEE bPPP

1nr0A 267 272 5.540 -60.00 -42.10 -62.70 -43.80 -92.00 -14.30 63.30 25.70 WNVATLKV *** EETTTTEE AAAa

1nr0A 310 315 5.243 -65.60 -22.00 -77.70 -51.50 -79.60 -20.50 72.70 49.10 VNPELGSI *** EETTTTEE AAAa

1nrjA 80 85 4.945 -95.70 -37.60 -135.70 98.90 -83.20 174.90 -70.40 113.80 LNKQPELY *** EEETTEEE ABPP

1nrjB 227 232 5.118 -62.70 -46.70 -59.40 -33.70 -94.50 5.50 53.30 46.50 GSINKRKI *** BTTTTB AAAa

1nszA 28 33 5.300 -74.60 -11.60 -73.90 95.10 86.00 16.50 39.90 62.30 FTNLGARI *** EETBTTEE APga

1nthA 77 82 5.598 -63.60 -30.20 -78.50 -34.20 -100.60 -11.50 58.00 35.60 YCTDTHRI *** EETTTTEE AAAa

1nthA 430 435 5.435 -52.00 -40.00 -59.60 -40.80 -104.90 -1.00 53.50 52.10 YDVKTVTP *** EETTTTEE AAAa

1nu0A 21 26 5.704 -65.90 -30.40 -58.70 -40.50 -101.20 -14.20 77.00 19.20 GQRITGTA *** EETTTTEE AAAa

1nuyA 1051 1056 4.858 -69.10 -29.30 -73.00 -30.80 -90.70 5.30 61.20 40.20 KAGIAHLY *** TTTTTT AAAa

1nuyA 1125 1130 5.173 -60.70 -57.60 -70.90 -17.60 -98.80 13.40 67.10 18.80 SNIDCLVS *** GGTTTT AAAa

1nuyA 1187 1192 5.579 -63.60 -22.60 -77.30 -29.80 -115.00 -4.50 63.60 45.90 LDPAIGEF *** EETTTTEE AAAa

1nxmA 45 50 5.405 -57.70 -50.90 -63.80 -19.60 -81.70 -5.40 113.20 -1.70 KMLPLGFP *** HHTTTT AAAa

1o08A 1009 1014 6.771 -67.70 120.90 84.40 2.50 -121.10 -53.90 -84.50 -33.60 DLDGVITD *** BTTTBT PgAA

1o20A 373 378 5.046 -58.60 -44.70 -67.00 -32.50 -88.80 7.50 80.40 17.20 DGGQFGFG *** STTTTT S AAAa

1o4sA 233 238 4.387 -91.20 -44.20 -60.10 -37.50 -119.20 15.30 47.20 51.10 FSKSHSMT *** STTTTT G AAAa

1o4vA 52 57 5.190 -59.10 -50.10 -57.80 -32.60 -84.30 3.00 93.00 1.00 NAEERGIE *** HTTTTT AAAa

1o4yA 175 180 5.034 -114.90 145.80 55.80 66.60 -88.70 166.70 -86.30 129.00 FIRNPFTD *** EETTTTEE BrPP

1o6sB 40 45 5.749 -63.60 118.60 105.50 -12.90 -115.40 -84.90 -101.60 -5.30 TGQGADTP *** EEBTTTBS PgAA

1o8xA 58 63 4.910 -76.10 -53.40 -69.50 -31.70 -99.70 5.30 52.30 61.40 FHESKNFE *** HTTTTTEE AAAa

1o91A 646 651 5.788 -57.60 -27.20 -75.70 -17.30 -122.00 -21.20 75.90 10.80 YNPQTGIF *** EETTTTEE AAAa

1o94D 134 139 5.005 49.90 62.30 55.80 43.20 70.20 14.50 -81.90 -16.20 GGYNQKVN *** EETTTTEE rrgA

1obdA 223 228 5.292 -73.70 -22.10 -72.60 -29.50 -115.70 -3.30 53.70 43.00 IDEKTNEI *** EETTTTEE AAAa

1obfO 87 92 5.278 -60.10 -48.00 -63.30 -35.70 -84.60 -0.70 55.50 47.20 PWGALKVD *** TTTTT S AAAa

1oewA 23 28 5.068 87.20 -179.30 -133.50 117.40 -60.90 157.80 -60.50 136.50 QIGTPAQT *** EETTTTEE pBPP

1oewA 244 249 5.274 -64.80 -41.40 -74.40 -39.00 -77.60 -27.60 95.50 32.10 SSSSVGGY *** EETTTTEE AAAa

1ofzA 219 224 5.349 76.60 -179.20 -92.40 -13.10 -55.20 -47.20 -90.30 178.10 SWGSTPNI *** EETTTTEE pAAB

1ogoX 306 311 4.202 -70.90 -9.60 -104.00 7.60 -152.00 58.90 60.30 26.60 ANAGDNYI *** BTTTTTB AADa

1ogoX 449 454 5.459 -115.80 -31.80 -60.30 -42.40 -117.50 92.90 -57.70 121.30 SETVVPSA *** TTTT E AABP

1ogqA 32 37 7.723 -57.90 -20.70 -90.70 -5.60 -98.50 28.10 71.40 17.20 TDabNRTW *** S TTTT S AAAa

1ogsA 110 115 5.147 -68.50 -23.90 -80.10 -6.70 -120.00 -138.40 -108.30 19.70 FSEEGIGY *** HSTTTT AABA

1oh4A 100 105 4.892 -143.30 149.90 -55.90 154.90 111.20 -14.60 42.90 58.60 VLNPGWVK *** EEETTTEE BPga

1oi2A 39 44 5.392 -127.00 148.40 59.70 66.00 -92.60 171.70 -79.40 131.40 LHQDPVYV *** EETTTTEE BrBP

1oi2A 268 273 5.580 -76.70 -19.10 -72.30 -41.10 -92.60 -15.50 66.40 44.30 WDYQQGSW *** EETTTTEE AAAa

1on3A 72 77 3.992 -71.00 156.00 45.80 52.10 80.50 0.40 -71.80 -16.90 TTLFGMDK *** TTTTTT PrgA

1oq1A 134 139 5.116 -125.90 166.80 -60.50 137.10 133.10 -16.20 53.90 34.50 RKSRGFHL *** EEETTTEE BPga

1ouwA 36 41 5.517 81.00 9.50 69.40 15.60 -140.10 -159.10 -86.20 43.60 YGGGGNNP *** EETTTTEE ggBD

1p1jA 254 259 6.237 -55.50 140.00 85.10 -11.20 -108.60 -67.20 -132.80 28.50 VSPGVNDT *** TTTTSS PgAA

1p3cA 51 56 5.281 -69.10 -30.80 -66.10 -46.10 -95.70 -5.20 53.00 46.90 YNTASRSY *** EETTTTEE AAAa

1p3dA 155 160 5.652 -61.20 -34.90 -75.00 -6.40 -111.20 -22.90 68.70 22.00 LVKSAGKN *** EETTTTEE AAAa

1p57A 22 27 5.518 -65.50 -23.20 -76.30 -17.10 -112.80 -28.40 82.20 25.10 FDKTEGTW *** EETTTTEE AAAa

1p5dX 22 27 5.446 -69.00 131.80 94.10 -17.50 -113.50 -88.30 -93.00 -3.50 GVVGDTLT *** EEBTTTB PgAA

1p5dX 332 337 6.745 -95.50 -40.50 -60.40 127.20 62.90 15.20 -151.60 -152.60 FFKERWFG *** EETTTT S APgB

1p5xA 61 66 4.519 42.30 62.50 53.00 40.50 70.40 15.00 -98.10 -18.00 YYDNSTFA *** TTTTTT G rrgA

1p5xA 72 77 5.438 -60.70 -25.70 -72.20 -31.40 -122.90 -5.80 67.10 28.70 YDPDNGKT *** BTTTTB AAAa

1p5xA 127 132 4.835 -72.80 -22.60 -91.10 -1.60 -124.80 7.60 58.20 43.50 PMHAANFT *** TTTTTT AAAa

1p9hA 44 49 6.574 -57.00 -14.30 -87.90 -6.60 -123.30 -26.70 72.40 16.10 ADPALGLE *** EETTTTEE AAAa

1pa1A 574 579 5.612 -58.60 -53.10 -62.60 -45.00 -72.30 -22.00 63.90 41.20 KMEEAQRS *** EETTTTEE AAAa

1pa1A 635 640 5.225 -63.30 -41.90 -61.90 -38.70 -108.00 2.80 56.00 53.40 IFEDTNLK *** EETTTTEE AAAa

1pa1A 662 667 5.572 -65.90 -16.70 -79.70 -35.20 -111.20 -12.70 66.90 14.60 ENLTTQET *** EETTTTEE AAAa

1pbyA 139 144 5.701 -74.20 -7.00 -88.50 7.30 -159.00 171.90 -82.20 82.00 LARDRDWW *** TTTTTTHH AABD

1pbyB 17 22 5.543 -61.40 -31.70 -71.50 -46.70 -77.30 -14.50 55.90 45.00 IDTEKMAV *** EETTTTEE AAAa

1pbyB 52 57 5.248 54.30 62.60 58.70 28.50 70.10 10.80 -88.60 -8.30 TVNKSESL *** EETTTTEE rggA

1pbyB 121 126 5.451 -63.40 -46.50 -61.10 -44.70 -98.00 -10.00 54.50 39.60 YDAETLSR *** EETTTTEE AAAa

1pbyB 160 165 5.310 -59.10 -30.00 -73.90 -43.80 -87.80 -20.00 68.90 46.30 MDPEAGTL *** EETTTTEE AAAa

1pbyB 192 197 5.115 -48.50 -42.10 -69.90 -23.30 -119.60 -18.80 77.60 15.60 QHESSGVM *** TTTTEE AAAa

1pbyB 266 271 5.430 -75.40 -8.40 -88.70 -32.40 -105.40 -13.20 54.30 54.90 FDLEKNAS *** EETTTTEE AAAa

1pbyC 49 54 5.533 -80.00 -10.10 -64.90 -34.10 -143.00 -63.30 -62.90 -44.20 ADTLNTYP *** TTTT T AAAA

1pfvA 129 134 5.292 -68.10 -25.80 -75.60 -57.20 -73.00 -28.60 77.40 53.20 YDPEKGMF *** EETTTTEE AAAa

1pfvA 334 339 4.998 -61.60 -43.80 -63.70 -32.00 -90.10 -4.80 76.00 25.50 MSKSRGTF *** TTTT AAAa

1pgs_ 203 208 5.604 -98.40 146.30 -110.30 4.80 -66.70 -41.80 -106.20 -25.90 RGbAEWbF *** EEEETTS BAAA

1pjxA 194 199 5.495 -57.70 -64.90 -69.70 -21.80 -83.30 -3.50 64.50 32.30 AETPTKKL *** EETTTTEE AAAa

1pjxA 243 248 5.785 -61.90 126.20 97.90 -11.60 -101.00 -10.40 68.80 11.50 ANWGSSHI *** EEETTTEE PgAa

1pjxA 286 291 5.555 -108.30 4.40 -72.20 -51.50 -102.90 -9.20 59.00 60.70 TEHENNAV *** EETTTTEE AAAa

1pl3A 20 25 5.285 -74.90 -17.00 -73.20 -50.30 -106.90 -3.80 56.60 43.90 TDPVHDVT *** EETTTTEE AAAa

1plc_ 87 92 5.479 -58.00 -48.20 -60.20 -33.60 -73.40 -11.40 100.00 15.10 PHQGAGMV *** GGTTTT E AAAa

1pmi_ 61 66 4.711 -59.00 -36.00 -91.10 -15.80 -103.80 48.30 71.30 25.10 KAIDLNNQ *** EETTTTTE AADa

1pmmA 273 278 6.475 -64.50 -34.00 -80.50 7.00 -102.20 -114.10 -77.20 1.40 ASGHKFGL *** EETTTTT AAAA

1pn2A 258 263 5.277 -59.10 -43.20 -60.50 -34.30 -118.70 -1.70 62.00 41.10 HVVDRGTI *** EETTTTEE AAAa

1pprM 74 79 5.716 87.30 171.40 -74.10 -16.60 -93.90 -6.00 67.70 42.00 ISGPNGVT *** BTTTTB pAAa

1pszA 110 115 6.329 -59.60 135.50 64.80 20.60 -132.50 -53.20 -108.60 -37.00 KTENKDYF *** BTTTEE PgAA

1px5A 137 142 5.636 -61.00 -32.50 -80.30 6.10 -137.70 -9.00 58.10 47.50 SSPQLQQE *** EETTTTEE AAAa

1pxvC 33 38 5.348 -73.10 -33.50 -68.70 -39.50 -85.20 -10.60 49.00 49.40 SNHQLQKS *** EETTTTEE AAAa

1pxvC 59 64 4.869 -64.30 -30.90 -62.90 -36.30 -111.70 9.10 57.30 63.90 IDTAHQRI *** EETTTTEE AAAa

1pz4A 47 52 5.073 -88.30 -0.70 -81.90 -49.50 -93.70 -12.90 72.70 70.30 MDLKNVKL *** EETTTTEE AAAa

1pzxA 38 43 6.283 -47.40 124.10 77.60 -0.10 -123.00 -52.30 -117.20 9.90 YKDGITIE *** EEBTTTB PgAA

1q2oA 371 376 5.357 -60.30 -33.60 -94.30 12.00 -120.80 -134.80 -64.30 -25.30 CDPHRYNI *** H TTTT AABA

1q35A 108 113 6.149 -55.40 -48.10 -79.20 -37.70 -103.70 -6.60 -133.90 -50.30 TSKDRVGK *** EETTTT S AAAA

1q74A 57 62 4.903 -58.70 -22.30 -77.30 -12.90 -124.30 -107.40 -77.30 -21.20 LTADHADQ *** GSTTTT AAAA

1q7fA 836 841 5.126 -45.70 -48.10 -69.90 -28.40 -107.80 -23.50 88.00 0.60 VVRNSGDI *** EETTTTEE AAAa

1q7fA 889 894 5.221 -103.70 -174.80 62.90 -18.10 -145.40 5.70 34.80 93.80 VECKVMRV *** EETTTTEE BgAa

1q7lA 110 115 5.320 -63.90 -32.80 -99.70 -37.10 -128.50 90.60 -141.40 30.50 RGAQDMKC *** TTTTTTHH AABA

1q8iA 362 367 7.628 -72.20 -32.30 -52.00 -26.60 157.40 -17.30 -38.20 -52.90 LPVDRHGG *** S TTTT AAxA

1q92A 42 47 5.647 -69.60 128.20 86.80 -4.20 -128.90 -54.20 -95.40 -40.70 DMDGVLAD *** SBTTTB PgAA

1qd1A 41 46 5.752 -71.20 -33.50 -76.90 -45.30 -71.20 -25.20 51.80 53.90 SGPSTNRT *** EETTTTEE AAAa

1qd1A 226 231 5.630 -61.10 -35.40 -70.40 -18.00 -115.90 -2.10 50.20 52.30 YLDEKNLA *** EETTTTEE AAAa

1qfmA 157 162 5.112 -59.40 -50.20 -64.50 109.20 90.40 0.80 46.00 50.60 MKVDGAKE *** EETTTTEE APga

1qftA 57 62 5.356 -62.60 -39.60 -57.00 -37.70 -99.70 -10.40 56.80 45.80 VNEDEKSI *** EETTTTEE AAAa

1qgiA 61 66 5.357 80.90 -160.50 -83.10 -23.30 -122.40 24.50 75.40 22.60 TIGLFGAT *** EETTTTEE pAAa

1qmgA 265 270 3.931 -61.00 -23.30 -78.60 -18.80 -124.40 -146.20 -97.40 32.70 QGKEVNGA *** HHTTTT AABA

1qnaA 64 69 5.834 -79.90 -31.80 -146.00 116.80 -70.40 144.90 -62.20 108.80 RIREPKTT *** EETTTTEE ABPP

1qnaA 155 160 5.177 -90.80 -30.40 -137.60 103.30 -75.60 165.70 -75.20 92.00 RMKVPKIV *** EETTTTEE ABPP

1qnrA 85 90 6.084 -66.60 -23.60 -99.50 18.80 -130.10 -128.00 -66.00 -14.80 TGADGLQT *** STTTTHH AABA

1qoyA 180 185 5.693 -64.20 -44.10 -65.40 -39.00 -65.80 -32.50 82.30 4.40 AGAAAGVV *** HTTTTTEE AAAa

1qsgA 177 182 5.522 -54.20 -52.10 -57.30 -28.30 -88.50 -1.90 104.30 22.70 AMGPEGVR *** HHTTTTEE AAAa

1qtnB 455 460 4.851 -79.60 3.70 -82.70 -46.90 -109.00 7.20 54.80 48.70 DDKKNMGK *** BTTTTBB AAAa

1qw9A 96 101 5.272 -75.70 -18.80 -63.20 -49.20 -117.60 14.60 52.00 46.70 LDLAWKSV *** EETTTTEE AAAa

1qw9A 415 420 5.163 -69.40 -38.90 -61.20 -41.60 -89.10 -8.60 51.20 56.30 YNEEKEEV *** EETTTTEE AAAa

1qwnA 470 475 7.906 -71.90 5.60 -120.90 9.00 -95.70 -87.10 -57.90 -25.80 QHHDGITG *** TBTTTTTS AAAA

1qwnA 529 534 7.426 -124.80 9.10 -150.80 126.80 -67.50 -18.30 106.80 154.10 DSRWPGSG *** STTTTTT ABAp

1qwnA 601 606 5.335 -69.60 -12.40 -88.30 -51.10 -85.00 -9.90 61.90 40.90 HDTLTKTI *** EETTTTEE AAAa

1qwyA 237 242 5.002 -81.80 -0.40 -109.20 19.80 124.80 -33.20 -76.70 -9.60 WSNYGGGN *** EETTTTEE AAgA

1qxmA 53 58 5.409 -60.90 -38.70 -59.60 -41.10 -91.00 -15.50 58.70 37.60 YDTNKQAY *** EETTTTEE AAAa

1qxmA 191 196 5.536 -62.40 -38.80 -62.90 -43.20 -92.80 -13.50 57.80 43.30 YNETKSAY *** EETTTTEE AAAa

1qxmA 201 206 5.620 -61.30 -29.50 -67.90 -39.60 -127.50 7.80 55.50 30.70 KCQENNRY *** EETTTTEE AAAa

1qz9A 81 86 4.618 -60.70 -21.10 -96.50 -6.80 -133.50 18.40 66.60 35.00 LATLIGAR *** HHTTTT AAAa

1qzrA 43 48 5.401 -78.30 -24.90 -70.50 -26.50 -119.80 -3.70 57.90 46.30 YDEETDCM *** EETTTTEE AAAa

1qzrA 89 94 5.233 -60.50 -50.10 -57.40 -35.30 -91.10 -1.80 55.60 29.80 IHAEEHTI *** EETTTTEE AAAa

1qzrA 162 167 5.562 -78.70 -18.20 -67.00 -40.00 -99.60 -17.50 68.50 51.50 ADLNVGQK *** EETTTTEE AAAa

1qzrA 174 179 4.935 53.90 63.00 59.60 42.60 70.60 1.50 -78.10 -23.30 WENNMSIC *** EETTTTEE rrgA

1r0mA 270 275 5.100 -74.10 -35.80 -55.30 -35.70 -95.00 0.00 101.00 8.50 KVARVGGH *** TTTTTSH AAAa

1r4pA 83 88 5.244 -79.40 -10.70 -78.40 -42.50 -93.80 -3.20 57.30 47.00 VNTATNTF *** EETTTTEE AAAa

1r5mA 289 294 5.435 -66.30 -25.60 -75.10 -45.50 -85.40 -19.30 71.60 33.10 WNVISGTV *** EETTTTEE AAAa

1r5mA 368 373 5.408 -73.50 -5.60 -93.20 -52.70 -88.80 -3.20 58.80 34.90 FNDTNKLL *** EETTTTEE AAAa

1r5mA 428 433 5.409 -77.00 -36.10 -64.90 -34.50 -107.10 -1.90 48.60 58.00 WSLKQNTL *** EETTTTEE AAAa

1r8sA 159 164 5.323 -69.30 -26.80 -70.90 -29.20 -91.90 -24.50 92.50 -5.50 TCATSGDG *** BTTTTBT AAAa

1r9lA 198 203 5.974 -55.70 133.20 89.80 -6.60 -126.00 -62.80 -96.40 -37.10 LKPGKDVV *** S BTTTEE PgAA

1regX 26 31 5.346 -79.80 -27.90 -66.90 -15.10 -133.30 24.90 41.60 63.00 ANNKDKVL *** EETTTTEE AAAa

1rg8A 16 21 5.301 -55.80 -29.70 -56.80 -64.10 -105.20 -1.20 73.70 40.40 YCSNGGHF *** EETTTTEE AAAa

1rgzA 47 52 5.088 -63.40 -50.10 -68.70 -31.70 -88.30 -2.40 51.80 47.20 ADIAANKP *** EETTTTEE AAAa

1ri6A 56 61 5.612 -149.80 168.30 -71.20 -1.80 -107.70 95.40 -53.10 110.10 GVRPEFRV *** EETTTTEE BABP

1ri6A 102 107 5.406 -67.30 -55.20 -61.40 -48.80 -73.40 -19.90 64.30 53.00 GSYNAGNV *** EETTTTEE AAAa

1ri6A 196 201 5.149 -63.70 -50.30 -69.50 -20.50 -129.00 13.70 42.30 62.20 VNELNSSV *** EETTTTEE AAAa

1ri6A 249 254 5.627 -69.30 -25.70 -79.90 -49.30 -81.40 -11.40 51.00 54.00 CDRTASLI *** EETTTTEE AAAa

1ri6A 307 312 5.467 57.50 -163.90 -68.10 -44.20 -64.30 -26.00 68.40 30.60 IVGEQGLL *** EETTTTEE pAAa

1rjdA 28 33 4.550 -70.20 -57.30 -70.80 -31.90 -92.40 -0.90 54.00 44.60 SSGLQRLS *** TTTTT AAAa

1rjoA 78 83 5.315 -74.60 -26.80 -61.40 -46.70 -96.00 -8.40 67.60 35.50 VSVTNGTV *** EETTTTEE AAAa

1rjoA 180 185 5.172 -81.60 -4.70 -97.10 -54.70 -75.20 -13.10 59.60 57.50 VDVVSKEV *** EETTTTEE AAAa

1rjoA 312 317 6.930 -73.00 135.80 81.40 -5.50 -116.90 -62.20 -85.10 -37.00 LELGCDaL *** BTTTB PgAA

1rkqA 255 260 5.158 -60.50 -34.80 -64.10 -32.70 -101.00 24.70 65.50 41.30 KSNLEDGV *** TTTTHH AAAa

1rkuA 8 13 6.287 -73.70 131.30 88.10 -18.70 -107.40 -47.20 -115.00 -29.30 DLEGVLVP *** ESBTTTB PgAA

1rmwA 45 50 5.994 -74.70 124.30 65.40 11.20 -110.90 -56.60 -112.00 -26.00 DIDDTVLF *** BTTTEE PgAA

1rqpA 97 102 5.442 86.50 -174.90 -66.10 -28.60 -100.40 -32.60 88.90 78.20 KGGARGQW *** BTTTTB pAAa

1rqpA 210 215 4.889 -77.50 149.90 -90.70 11.80 -87.10 -28.60 78.40 28.80 IDHPFGNV *** EETTTTEE PAAa

1ru4A 200 205 4.737 -67.40 -25.60 -52.60 -50.40 -119.60 32.90 51.70 40.30 YDPKKNGS *** TTTTTS AAAa

1ru4A 355 360 4.727 -64.40 -18.50 -100.10 4.90 -134.10 -103.40 -92.20 -8.00 SNSWDTGP *** SSTTTTS AAAA

1rwhA 660 665 5.274 -60.40 -51.90 -62.80 -42.60 -79.80 -4.60 45.40 47.60 EFKTAKTT *** EETTTTEE AAAa

1rwiA 209 214 5.535 -77.30 -19.70 -116.30 52.20 -156.30 -1.20 59.30 34.80 TEHNTNQV *** EETTTTEE ADAa

1rwjA 35 40 5.969 -105.50 130.70 -100.70 1.40 -52.90 -42.70 -103.50 -38.90 ECHTKLFA *** GTBTTTB BAAA

1rwjA 63 68 5.293 -80.50 71.00 -119.90 8.90 88.50 -5.20 -121.90 -44.10 ACHNGKDA *** GTTTTTSS DAgA

1rwrA 194 199 5.571 -65.10 -35.50 -73.20 -37.30 -97.10 -11.70 52.00 40.20 YDHATRRA *** EETTTTEE AAAa

1s0aA 50 55 4.465 50.30 45.20 68.30 73.60 75.70 -39.30 -64.90 -41.60 MSSWWAAI *** STTTTT T rrgA

1s0aA 157 162 4.812 -87.00 -56.70 -57.70 -39.10 -91.10 2.40 52.30 50.80 DPDNSMHS *** TTTTTGG AAAa

1s0aA 249 254 6.385 -64.80 159.50 64.70 44.60 94.20 -12.00 -53.60 -40.60 ATGFGRTG *** TTTTTTTS PrgA

1s1dA 133 138 5.279 59.90 -130.30 -83.00 11.40 88.10 2.90 -105.20 10.50 SDGDGTVE *** EETTTTEE pAgA

1s1dA 219 224 5.535 -64.30 -35.70 -63.10 -44.70 -100.10 -8.60 53.80 46.40 WSDTLQRW *** EETTTTEE AAAa

1s2oA 65 70 5.376 47.10 61.50 55.40 43.70 71.80 7.60 -77.00 -22.00 LTAVGSEI *** EETTTTEE rrgA

1sg4A 10 15 5.386 -56.50 -31.70 -67.30 -38.20 -79.80 -17.10 77.90 16.30 PDAGAGVA *** EETTTTEE AAAa

1sg4A 22 27 6.768 -65.80 161.10 -81.10 -12.30 -140.60 18.70 50.70 42.70 KNPPVNSL *** TTTTEE PAAa

1sluA 64 69 5.029 -60.70 142.90 75.80 0.10 -127.20 -34.60 76.60 26.50 TLEGWGYD *** EETTTTEE PgAa

1sq9A 213 218 5.444 -62.20 -31.40 -78.40 -43.60 -95.20 -17.80 64.40 35.70 SELSTLRP *** EETTTTEE AAAa

1sq9A 319 324 5.453 -59.90 -45.70 -56.00 -50.20 -88.50 -9.70 60.10 31.90 WDVKTKER *** EETTTTEE AAAa

1sqsA 108 113 6.447 -42.00 -43.10 -110.70 18.60 -108.60 -7.90 52.60 39.60 GWSHLFRL *** GGTTTTTT AAAa

1srvA 207 212 5.474 -66.60 -7.80 -74.70 -51.20 -85.30 -13.60 56.80 39.30 TNPETMEA *** SBTTTTBE AAAa

1srvA 302 307 5.518 -65.30 -40.00 -63.50 -26.90 -90.10 12.20 93.40 -0.70 ISEELGFK *** E TTTT AAAa

1t2wA 162 167 6.592 -46.70 -41.50 -118.90 130.50 77.10 -21.40 -85.80 -33.10 VKPTDVGV *** E TTTT ABgA

1t2wA 188 193 5.379 -59.30 -28.70 -68.40 -35.30 -107.80 -12.70 69.60 29.90 YNEKTGVW *** EETTTTEE AAAa

1t3tA 590 595 5.424 -66.00 -25.20 -72.90 -49.00 -93.80 -3.30 53.70 59.60 HDNHFDNQ *** EETTTTEE AAAa

1t3tA 670 675 5.018 68.30 -175.60 -58.10 -30.90 -91.20 1.20 44.20 60.40 MVGPWQVP *** EETTTTEE pAAa

1t5hX 23 28 4.903 -62.60 -35.60 -66.00 -34.20 -92.60 -7.10 66.30 26.90 AVPARGLR *** EETTTTEE AAAa

1t6eX 361 366 5.250 -81.30 -4.30 -91.10 -46.80 -83.00 -5.00 51.70 53.70 FDMEKKRL *** EETTTTEE AAAa

1t8kA 27 32 6.325 -54.10 -49.90 -77.70 -49.20 -79.60 -30.50 -108.40 -35.80 ASFVEDLG *** BTTTTT AAAA

1t8uA 186 191 6.265 -78.00 -48.00 -76.40 -7.40 -115.20 -45.40 -107.20 -43.40 PHFFDRSY *** TTTT G AAAA

1t8uA 344 349 5.660 -56.30 -48.60 -51.90 -53.20 -77.10 -25.10 81.60 16.20 FNKTKGFP *** EETTTTEE AAAa

1t9hA 70 75 5.405 -93.00 -45.70 -132.80 117.70 -64.90 160.90 -76.90 95.80 ELIRPPIC *** EETTTTEE ABPP

1t9hA 87 92 4.794 -141.40 165.70 59.30 53.40 -93.70 171.20 -58.00 136.70 SAVQPSFS *** ESTTTT BrBP

1tg5A 305 310 4.874 -51.60 -54.90 -83.80 -2.90 -121.50 45.70 105.80 -6.70 KRSSIGGF *** HTTTTTS AADa

1thfD 142 147 5.121 55.10 57.10 51.80 48.10 67.60 15.80 -101.40 -9.90 FTYSGKKN *** EETTTTEE rrgA

1ti6B 117 122 5.453 -60.90 -46.20 -64.20 -41.40 -91.70 -10.80 50.60 59.00 WNEEENVA *** EETTTTEE AAAa

1tjjA 100 105 4.641 -60.80 -45.20 -59.40 -42.30 -95.80 12.50 62.40 34.70 PLRTYGLP *** TTTTTT AAAa

1tjoA 17 22 5.216 -80.40 -33.70 -55.80 -44.50 -90.10 3.00 51.00 48.10 GSDALRMD *** BTTTTB AAAa

1tjvA 203 208 3.992 66.40 -156.50 -79.70 -27.80 -167.10 -49.90 -90.90 -0.30 GSGALAGN *** T TTTT pAAA

1tjxA 283 288 5.148 -58.20 -42.10 -58.80 -45.90 -85.90 -16.60 63.50 40.00 YVPTAGKL *** EETTTTEE AAAa

1tu9A 78 83 4.966 -54.70 -33.70 -77.40 -5.60 -130.80 -110.20 -67.00 -27.20 HSRAALDI *** TSTTTT AAAA

1tvzA 360 365 5.407 -74.70 -51.80 -60.70 -41.60 -81.40 -5.10 47.40 53.00 DEEQDAVH *** TTTTTGG AAAa

1txgA 47 52 5.795 -65.30 -20.20 -93.20 0.30 -123.00 -26.30 70.60 21.80 EHPRLGVK *** BTTTTB AAAa

1ty9A 111 116 5.356 -55.30 -41.60 -61.50 -37.20 -111.00 5.90 45.50 57.50 YWRETSQQ *** EETTTTEE AAAa

1tyv_ 341 346 4.332 72.70 179.30 -59.00 -31.30 -68.60 -8.00 52.80 50.20 NGGFERDG *** B TTTT pAAa

1u02A 8 13 6.366 -63.40 126.60 79.30 -3.70 -116.10 -53.40 -101.60 -25.20 DYDGTLVP *** E BTTTB PgAA

1u0fA 90 95 5.080 -75.20 -26.40 -74.00 -27.70 -106.20 -5.00 51.60 58.70 INYTEDRA *** BTTTTB AAAa

1u4gA 16 21 6.414 -74.10 129.70 104.70 -7.10 -131.70 -72.60 -76.50 -29.30 YTYGSDYG *** EEETTTB PgAA

1u60A 218 223 5.349 -74.30 -8.60 -93.40 -33.10 -101.10 -6.20 54.10 52.70 VNPLTHKR *** EETTTTEE AAAa

1u6dX 346 351 5.493 -59.10 -23.40 -64.40 -45.40 -118.10 4.40 70.80 17.70 YNPSDGTW *** EETTTTEE AAAa

1u6dX 397 402 5.811 -58.00 -28.50 -70.80 -28.40 -108.30 -12.10 55.60 39.70 YNPMTNQW *** EETTTTEE AAAa

1u6dX 444 449 5.547 -54.20 -38.20 -61.20 -31.40 -108.30 -4.40 47.60 55.70 YEPERDEW *** EETTTTEE AAAa

1u6dX 491 496 5.517 -67.70 -35.70 -69.10 -39.40 -82.90 -19.30 60.40 61.90 YYPERNEW *** EETTTTEE AAAa

1u6dX 538 543 5.866 -70.40 -17.30 -81.30 -53.70 -82.40 -14.40 67.00 27.30 YDVETETW *** EETTTTEE AAAa

1u6dX 585 590 5.718 -69.00 -24.80 -65.90 -40.70 -100.90 -4.30 51.30 50.20 YDPDTDTW *** EETTTTEE AAAa

1u7pA 12 17 6.518 -59.30 127.90 61.10 21.00 -126.00 -54.90 -101.90 -26.40 DLDYTLWP *** BTTTBS PgAA

1u7pA 20 25 7.614 -65.60 -20.60 -95.00 6.20 -133.60 -54.00 -80.80 -11.50 FWVDTHVD *** S TTTT AAAA

1uaiA 79 84 5.436 57.40 58.00 68.60 25.40 74.90 15.10 -79.90 -28.10 MTDGGEEK *** EETTTTEE rggA

1uasA 332 337 5.385 -68.70 -28.90 -89.90 -30.60 -86.10 -19.20 57.70 53.00 RDLWAHSS *** EETTTTEE AAAa

1ubkL 339 344 5.988 -56.80 -33.60 -109.40 19.70 -115.40 162.90 -84.90 59.70 HVRHSWYE *** E TTBTBS AABD

1ubkS 27 32 5.567 -130.30 123.30 49.10 67.00 -84.80 147.70 -79.40 168.70 RAFEPYID *** T TTTTHH BrPP

1uekA 138 143 4.944 64.50 -129.70 -95.10 14.80 110.70 -3.60 -96.00 -13.50 ARGVGERL *** EETTTTEE pAgA

1ufoA 183 188 6.528 -106.90 -8.30 -77.70 -46.00 -121.70 95.70 -56.00 142.30 RDHIVPLA *** T TTTTHH AABP

1ug6A 407 412 5.259 -56.40 -40.40 -57.50 -38.70 -100.20 -13.30 64.70 13.80 VDFPSQRR *** EETTTTEE AAAa

1ughI 48 53 5.030 -65.30 -41.20 -49.40 -54.50 -96.30 -9.00 65.90 38.30 YDESTDEN *** EETTTTEE AAAa

1uj2A 113 118 5.308 -71.80 -24.10 -68.60 -36.90 -109.70 2.90 57.10 40.90 YDFVSHSR *** EETTTTEE AAAa

1umgA 16 21 5.722 59.40 18.20 133.30 -157.90 -89.00 46.30 -90.70 -59.30 SLAGHHIV *** BBTTTTB gbDA

1umgA 172 177 5.338 -65.00 -15.10 -92.20 -47.40 -79.80 -23.10 75.00 32.50 LDVYQGEA *** EETTTTEE AAAa

1umhA 188 193 5.575 -66.80 -43.50 -61.00 -28.40 -82.10 6.20 93.10 10.70 DLQAEGYW *** ETTTTT AAAa

1uolA 104 109 4.540 68.40 -169.80 -66.50 -22.70 -98.30 2.30 64.60 44.10 YQGSYGFR *** B TTTT E pAAa

1uolA 127 132 5.161 -64.50 -38.90 -60.50 -34.40 -102.80 -7.20 51.40 51.10 YSPALNKL *** EETTTTEE AAAa

1uolA 242 247 5.115 -67.50 139.90 80.30 -5.70 -120.90 -124.30 -56.80 -30.00 SCMGGMNR *** T TTTTTT PgBA

1uozA 355 360 4.256 61.40 23.00 78.40 9.40 -134.80 10.30 67.10 27.00 GTbGRGEP *** TTTT ggAa

1uuyA 38 43 5.209 -70.60 -42.60 -61.90 -24.00 -91.70 -1.10 93.70 12.70 SSEKLGGA *** TTTTTTSE AAAa

1uv4A 108 113 7.118 -69.60 -38.70 -64.50 -29.50 -76.40 -30.80 79.30 16.60 TSISSGGW *** S TTTT AAAa

1uv4A 177 182 4.447 -60.10 -28.30 -105.80 7.90 -132.70 42.30 68.70 15.90 ARPNNGGA *** TTTTT AADa

1uv4A 270 275 4.553 -66.20 -15.10 -98.60 3.30 -148.70 66.50 59.10 27.30 YDANDNGI *** EETTTTTE AADa

1uwcA 23 28 4.571 -63.80 -15.70 -81.90 -10.50 -138.90 50.70 55.00 37.40 QAAYADLa *** HHTTTTTT AADa

1uwcA 43 48 5.554 -74.70 -28.40 -74.00 -37.40 -103.00 -13.30 55.10 34.60 YNAQTDIN *** EETTTTEE AAAa

1uwcA 55 60 5.417 -73.30 -33.70 -57.40 -50.30 -93.70 5.60 52.00 58.60 RDDTSKEI *** EETTTTEE AAAa

1uwkA 452 457 5.202 -61.90 -15.50 -103.80 10.70 -147.90 -106.60 -65.40 -26.00 SSPNAETE *** E TTTTTT AAAA

1uwkA 552 557 6.745 -53.10 -32.80 -84.20 -6.60 -116.80 -49.60 -99.10 -7.50 DLPMITG# *** B TTTT AAAA

1ux6A 1097 1102 5.005 -51.50 -37.10 -56.90 -40.90 -106.50 -12.90 86.10 9.30 HRPKTGFI *** EETTTTEE AAAa

1uylA 83 88 5.270 -73.10 -26.20 -71.10 -41.30 -88.30 -9.20 56.70 48.70 PNKQDRTL *** EETTTTEE AAAa

1uzeA 513 518 4.768 -71.50 -62.50 -68.70 -22.90 -81.70 -6.80 53.40 49.40 FHIPSSVP *** TTTTTT AAAa

1v0wA 25 30 6.427 -66.50 129.20 106.90 -35.80 -111.60 -51.80 -102.60 -18.50 GLEGDVWE *** GGBTTTEE PgAA

1v0wA 162 167 4.872 -70.00 -45.80 -69.60 -32.00 -92.00 2.40 55.70 49.60 TSKTAFSW *** SBTTTTB AAAa

1v3eA 552 557 5.378 -48.70 -34.00 -46.70 -57.30 -95.90 1.70 58.60 34.00 NHKSLNTL *** EETTTTEE AAAa

1v4bA 113 118 6.492 -58.90 144.60 62.10 4.60 -122.30 -62.00 -103.50 -7.90 ARAGVTFR *** BTTTEE PgAA

1v5iB 59 64 5.741 -60.40 129.30 93.60 -12.00 -114.30 -78.60 -88.70 -34.40 GLQGDLID *** HTBTTTEE PgAA

1v5vA 262 267 6.620 -65.40 134.10 107.80 -24.20 -108.80 -68.90 -83.00 -43.40 TLYGNETK *** BTTTB PgAA

1v5vA 282 287 4.811 -72.80 -22.90 -91.50 -11.30 -113.90 8.80 60.10 41.80 TPLQANLE *** TTTTT G AAAa

1v73A 295 300 5.112 51.40 61.80 53.70 42.70 61.50 21.20 -76.00 -20.30 GLFHNKVI *** EETTTTEE rrgA

1v7wA 7 12 5.278 -61.80 -40.30 -58.80 -38.90 -103.70 3.90 52.60 50.50 FDNDNREY *** EETTTTEE AAAa

1v7wA 86 91 6.724 -76.30 157.50 -85.80 -15.90 -69.40 -33.00 56.10 52.00 SWQPVAKS *** SSTTTT PAAa

1v7wA 548 553 5.501 -80.40 -1.60 -90.70 -44.80 -83.90 -34.10 86.80 50.70 WDDEGGWY *** EETTTTEE AAAa

1vd5A 125 130 5.508 -48.80 -43.70 -66.10 -25.70 -108.60 -10.50 63.90 38.10 WRADAGII *** EETTTTEE AAAa

1vd5A 339 344 5.006 -53.00 -55.30 -63.40 -24.90 -86.80 -2.90 65.90 26.70 YHVRGGIS *** SBTTTTBS AAAa

1vhh_ 56 61 4.814 -56.10 -30.40 -76.90 -15.50 -104.30 -118.20 -97.30 -0.50 KTLGASGR *** TSTTTT AAAA

1vjoA 157 162 5.421 -58.20 -24.70 -81.50 -11.00 -129.00 -18.80 84.00 10.60 AETSTGAR *** EETTTTEE AAAa

1vjoA 195 200 5.274 -68.30 -47.50 -55.40 -36.10 -83.90 -2.70 63.60 33.50 FLDAWGVD *** TTTTT S AAAa

1vjvA 320 325 5.515 -69.00 -47.20 -53.70 -34.10 -115.10 -3.10 49.30 47.70 WKRSTNKK *** EETTTTEE AAAa

1vkbA 45 50 5.472 84.50 174.20 -65.90 -21.10 -95.10 2.10 51.70 50.00 IAGEHNIP *** EETTTTEE pAAa

1vkkA 37 42 5.364 -61.00 -40.90 -65.10 -41.70 -82.90 -13.10 53.40 53.80 VDKDRQMV *** EETTTTEE AAAa

1vknA 293 298 5.084 -67.00 -37.20 -66.30 -32.40 -112.30 -1.80 61.80 44.40 XEERTNTL *** EETTTTEE AAAa

1vkpA 218 223 5.065 -61.30 -39.10 -65.90 -33.90 -102.10 0.80 66.40 40.40 GDEDTNGH *** T TTTT AAAa

1vkyA 32 37 5.166 -65.40 -44.70 -70.20 -34.00 -95.10 -17.90 65.90 21.50 LHRKTQRI *** EETTTTEE AAAa

1vl1A 160 165 5.669 70.80 -160.20 -136.30 120.20 -63.40 151.20 -86.80 88.60 PSGDPKVP *** SBTTTTB pBPD

1vlrA 55 60 5.394 -57.70 -34.20 -65.60 -46.60 -93.90 -5.90 60.90 52.90 ESARDKII *** EETTTTEE AAAa

1vmeA 10 15 5.014 -95.30 -39.80 -139.00 106.80 -65.80 155.20 -75.50 107.00 IFDDPEIY *** EETTTEEE ABPP

1vns_ 419 424 7.716 -61.30 122.30 103.30 -23.40 -96.90 -49.70 -89.50 -27.80 YYNGRVGT *** HHBTTTB PgAA

1vp2A 121 126 5.289 -78.00 -5.30 -85.90 -50.70 -79.90 -19.60 58.70 51.70 FDPVENTL *** EETTTTEE AAAa

1vp4A 246 251 6.045 -60.30 -40.40 -96.50 -20.60 -108.90 -13.20 -132.00 -67.60 TFSKVLAP *** ESTTTT G AAAA

1vprA 1131 1136 5.433 -58.10 -35.10 -73.50 -15.20 -116.10 -15.70 76.10 11.90 WEXESGRC *** EETTTTEE AAAa

1vpsA 65 70 4.974 -75.30 1.70 -120.30 -4.80 -113.40 9.20 126.70 -19.40 ESLTEGGQ *** TTTTGG AAAa

1vpsA 146 151 5.023 -81.60 -16.70 -68.30 -38.80 -117.40 -2.90 63.00 58.80 TDTVNTKG *** EETTTTEE AAAa

1vr5A 65 70 5.326 -73.50 -13.60 -78.10 -33.80 -115.80 -3.10 59.80 57.80 YDPLNDKF *** EETTTTEE AAAa

1vsrA 66 71 4.889 -70.70 -54.70 -66.80 -32.00 -101.80 14.20 58.80 54.70 GCFWHHHH *** TTTTT S AAAa

1vybA 195 200 5.323 -66.30 -33.70 -54.20 -35.20 -112.80 0.70 50.00 51.40 FSAPHHTY *** EETTTTEE AAAa

1vyrA 31 36 4.984 -121.40 140.40 -73.80 155.20 112.50 -14.70 54.60 49.90 SIEPGDIP *** BTTTTB BPga

1vyrA 355 360 6.538 -65.90 128.10 74.70 17.60 -132.30 -65.30 -108.70 5.10 GAEGYTDY *** SSTTTT S PgAA

1vziA 63 68 5.925 -124.30 -134.30 -57.60 -32.70 -111.10 129.30 -62.90 126.50 KVGAVAHP *** EETTTT BABP

1vzyA 9 14 4.851 45.40 57.80 55.50 43.90 74.70 2.90 -79.40 -19.00 LAYDGKVR *** EETTTTEE rrgA

1w1hA 488 493 5.353 -72.80 -22.40 -76.00 -54.30 -89.80 -3.70 58.50 54.70 VDPVNKVL *** EETTTTEE AAAa

1w1oA 41 46 5.166 -66.90 -36.90 -67.00 -32.10 -83.50 4.30 76.80 14.50 ALALDGKL *** TTTTT E AAAa

1w23A 151 156 5.446 -56.90 -37.10 -76.50 -34.60 -97.10 -15.00 74.80 32.70 NNTIYGTQ *** EETTTTEE AAAa

1w23A 194 199 5.109 -74.80 19.70 -116.40 -57.10 -118.90 -3.10 -119.30 -18.60 GAQKNLGP *** ETTTTTS AAAA

1w2wB 232 237 5.688 -63.20 162.20 -86.80 -13.60 -78.80 -22.40 68.70 40.90 ETRPYNQG *** TTTTHH PAAa

1w3oA 62 67 5.505 -65.00 -33.40 -66.20 -47.80 -63.50 -22.90 72.70 35.40 YRPEQGDL *** EETTTTEE AAAa

1w3oA 144 149 6.698 -63.80 129.40 96.20 -9.40 -127.80 -72.80 -57.10 -43.80 LKVGETTR *** BTTTB PgAA

1w6sA 249 254 5.989 -55.50 -27.10 -77.10 -23.10 -102.40 -17.70 60.70 22.80 YDPGTNLI *** EETTTTEE AAAa

1w6sA 401 406 5.645 -66.80 -26.50 -77.00 -38.90 -81.90 -15.70 56.30 43.60 YDPKRELF *** EETTTTEE AAAa

1w6sA 445 450 5.460 -71.30 -10.10 -101.70 -22.50 -91.60 -7.10 61.90 48.10 GDRQNYEG *** SBTTTTB AAAa

1w78A 222 227 6.024 -61.00 132.60 94.10 -15.30 -121.90 -82.60 -71.10 -26.80 QRRGVEWN *** EEBTTTBE PgAA

1w78A 294 299 5.134 -125.00 164.00 46.40 66.10 -86.00 161.50 -69.80 123.30 VSESPRVI *** EETTTEEE BrPP

1w9aA 41 46 5.340 -75.10 -14.70 -86.80 -47.60 -83.60 -15.10 57.30 61.00 FDPRKLLI *** EETTTTEE AAAa

1wddS 104 109 5.746 -80.40 -12.20 -76.70 -51.20 -84.20 -12.00 52.10 64.20 FDNVRQVQ *** EETTTTEE AAAa

1wdjA 146 151 5.399 -67.00 -20.00 -80.20 -27.10 -103.00 -5.90 54.20 50.20 VDPYARAV *** EETTTTEE AAAa

1whsA 23 28 4.886 -60.50 -52.30 -50.40 -53.70 -78.20 -25.40 81.90 41.10 VDEGAGRS *** EETTTTEE AAAa

1wlgA 121 126 5.435 89.50 179.20 -137.40 111.10 -69.80 150.00 -58.00 140.30 ATGTPPTI *** BTTTTB pBPP

1wm3A 73 78 4.935 -65.00 -42.20 -60.60 -38.30 -88.00 7.20 58.30 39.60 TPAQLEME *** TTTTT AAAa

1woqA 30 35 5.678 -67.50 -11.40 -76.50 -33.60 -108.80 -15.40 73.20 27.50 VDLKKGKL *** EETTTTEE AAAa

1ws8A 91 96 5.700 -63.10 -40.00 -66.00 -24.70 -80.70 -3.30 103.70 7.10 GHaQLGQK *** TTTTTT E AAAa

1wu4A 61 66 5.436 -50.10 -32.30 -78.30 -38.90 -97.40 0.90 53.10 43.40 LDTGNLDV *** BTTTTBE AAAa

1wvfA 92 97 4.105 -57.90 131.50 87.20 -5.40 -128.10 10.10 118.90 -5.70 RNFGYGSA *** TTTTTT PgAa

1wvfA 120 125 5.380 -79.10 -10.80 -93.20 -49.50 -84.40 -5.60 54.70 66.10 IDPEMCYA *** EETTTTEE AAAa

1x74A 215 220 4.910 -64.00 -34.60 -118.50 11.00 -113.40 7.00 107.30 -12.90 ANMLDGGA *** STTTT S AAAa

1x7yA 152 157 5.221 -67.70 -42.70 -62.90 -29.50 -108.00 17.90 55.20 47.20 GCKERHFV *** BTTTTB AAAa

1x7yB 47 52 5.261 -81.10 -23.20 -63.30 -44.80 -78.60 3.00 116.80 -7.00 EDVAFGGV *** TTTTTT T AAAa

1x9dA 275 280 5.080 -74.00 -32.60 -68.70 -39.70 -90.20 -4.60 57.90 50.90 LKPVSRSF *** EETTTTEE AAAa

1xd3B 56 61 4.962 -58.50 -42.60 -55.00 -37.60 -93.40 8.60 56.20 43.50 TLSDYNIQ *** BTTTTT AAAa

1xedA 69 74 5.277 -48.80 -46.80 -53.10 -43.40 -79.60 -24.60 76.80 39.60 NFPENGTF *** EETTTTEE AAAa

1xfoA -3 2 5.025 -82.00 68.70 -104.50 -137.80 -64.10 78.40 -1.80 125.70 #RGSHMEV *** TTTT DBDX

1xjuA 126 131 5.394 -66.30 -38.30 -61.40 -36.30 -99.70 -11.80 72.30 31.60 YSKARGMR *** EETTTTEE AAAa

1xmbA 150 155 6.409 -67.40 -35.80 -70.80 -32.80 -85.50 4.40 62.70 22.50 PAEEGLSG *** TTTT H AAAa

1xmtA 31 36 5.030 58.10 59.30 55.40 41.10 78.60 10.50 -90.00 -14.10 MRNNGKVM *** EETTTTEE rrgA

1xtpA 208 213 5.324 -81.30 -3.10 -82.60 -53.50 -104.70 5.70 60.90 65.20 VDKEDSSL *** EETTTTEE AAAa

1xu1R 80 85 5.618 -77.20 -9.70 -83.30 -49.30 -82.30 -17.10 60.50 48.30 YDHLLRDd *** EETTTTEE AAAa

1xvwA 93 98 5.278 -87.20 -46.60 -154.90 113.90 -57.90 141.30 -69.60 112.20 SDFWPHGA *** E TTTTTH ABPP

1xvwA 108 113 5.397 -63.40 -25.90 -79.50 -40.30 -94.90 -15.40 73.30 29.00 FNEQAGIA *** EETTTTEE AAAa

1y0bA 22 27 6.067 -71.10 -12.80 -80.10 -17.30 -141.20 -15.00 -123.70 -45.50 KVDSFLNH *** E TTTTSS AAAA

1y0bA 46 51 7.283 -63.60 -22.90 -116.90 31.20 -111.80 -1.10 61.00 43.20 RFAKDGIT *** HTTTTT AAAa

1y0bA 98 103 5.304 -69.60 -35.30 -70.20 -36.50 -117.20 5.50 47.10 48.60 YSFTKQTE *** EETTTTEE AAAa

1y12A 155 160 5.338 -66.10 -46.70 -53.10 -44.50 -103.30 0.80 57.80 43.10 WNIRQNVQ *** EETTTTEE AAAa

1y21A 48 53 7.239 -61.00 -45.80 -63.60 -53.70 -60.40 -31.50 47.10 62.60 FGFQTDKP *** S TTTTSS AAAa

1y4wA 195 200 5.446 -53.70 -46.00 -64.50 -35.10 -106.80 -3.40 51.00 54.70 WHDESQKW *** EETTTTEE AAAa

1y4wA 440 445 5.269 -67.30 -42.00 -68.90 -38.20 -88.20 -12.60 48.70 56.40 YDFAKQQI *** EETTTTEE AAAa

1y4wA 493 498 5.101 64.20 -126.50 -87.50 -0.60 113.20 10.40 -69.70 -20.40 FGGQGETT *** EETTTTEE pAgA

1y7bA 164 169 5.602 -64.00 -38.20 -52.80 -53.70 -86.50 -5.80 55.00 48.20 YSDKEKKL *** EETTTTEE AAAa

1y8aA 6 11 6.042 -83.50 110.60 122.90 -67.60 -63.00 -38.20 -126.00 -48.00 DWEGPWIL *** SBTTTB PxAA

1yacA 60 65 6.380 -59.20 -17.40 -98.70 62.00 -171.10 -160.30 -56.20 -32.10 SAETGPNG *** ESTTTTT ADBA

1yfqA 41 46 5.224 -76.50 -11.30 -83.10 -50.20 -89.20 -8.90 55.20 59.80 FDIQAKNV *** EETTTTEE AAAa

1yfqA 279 284 5.162 -74.40 -19.40 -77.50 -36.20 -99.20 -7.30 59.20 50.80 WNLQTRKK *** EETTTTEE AAAa

1yge_ 116 121 5.515 -62.10 90.70 -165.60 -101.70 -59.50 -115.30 -62.00 -1.00 EAISNQGT *** TTTTS PXAA

1yiiA 276 281 5.338 -50.90 -32.40 -114.10 19.60 -123.30 -65.20 -143.20 19.30 SRSEIDLL *** HHTTTTHH AAAA

1yjfA 135 140 4.736 -82.10 -65.50 -71.10 -19.30 -87.90 0.70 52.90 56.30 GNILGHKL *** STTTTT B AAAa

1ykdA 110 115 5.361 -73.30 -21.20 -70.80 -37.10 -101.40 -4.20 53.10 53.20 LDEEKQEL *** EETTTTEE AAAa

1ykdA 298 303 5.267 -65.80 -38.50 -56.10 -51.80 -89.40 -5.60 58.50 52.70 IDRDRHEL *** EETTTTEE AAAa

1yleA 99 104 5.295 -62.70 -40.50 -81.40 -23.50 -94.50 -18.50 65.40 26.20 ASRSLSIH *** EETTTTEE AAAa

1zpdA 72 77 6.358 -60.80 132.10 70.30 -72.50 -63.10 -31.70 -86.50 -43.50 VTYSVGAL *** E TTTTHH PdAA

1zpdA 105 110 5.917 -80.30 140.80 52.80 43.40 -131.20 2.50 62.40 37.90 NDHAAGHV *** GGTTTT B PrAa

1zpdA 220 225 5.711 -69.80 -37.70 -62.50 -32.70 -78.70 -7.10 65.20 31.30 KLRAAGAE *** TTTTTT H AAAa

1zrn_ 11 16 6.127 -67.60 114.00 97.40 -15.70 -104.50 -52.20 -105.50 -35.60 DLYGTLFD *** SBTTTEE PgAA

2baa_ 114 119 5.315 74.90 -172.60 -59.70 -34.00 -102.60 5.50 49.90 53.70 GRGPIQLS *** BTTTTB pAAa

2bbkH 52 57 5.453 -64.40 -25.50 -69.00 -47.00 -89.50 -22.20 72.00 44.40 IDGEAGRV *** EETTTTEE AAAa

2bbkH 153 158 5.202 -73.90 -29.20 -74.70 -42.20 -87.50 -6.70 58.30 54.50 VDLEGKAF *** EETTTTEE AAAa

2bbkH 221 226 5.281 -63.80 -38.90 -65.80 -30.30 -92.80 -19.30 70.40 21.80 YSQKAGRL *** EETTTTEE AAAa

2bbkH 275 280 5.175 -63.30 -59.90 -60.10 -36.60 -88.20 -8.30 54.00 53.60 YHRALDRI *** EETTTTEE AAAa

2bbkH 337 342 5.451 -69.80 -38.60 -70.00 -39.90 -81.80 -10.60 52.90 41.00 LSTGDKTL *** EETTTTEE AAAa

2bbkL 63 68 5.417 -60.30 -33.70 -58.60 -44.70 -112.30 2.30 67.40 19.60 YNPTDGQS *** EETTTTEE AAAa

2bemA 31 36 5.367 -84.20 -27.90 -153.90 116.80 -67.80 131.80 -55.50 125.00 YVESPASR *** EEEETB H ABPP

2bemA 74 79 4.884 -74.60 -49.00 -63.10 -28.80 -92.50 -2.10 55.00 27.50 GHIASADK *** T TTTTT AAAa

2bgrA 136 141 5.202 -74.70 -26.30 -76.30 -45.00 -95.00 9.10 42.30 59.10 YDLNKRQL *** EETTTTEE AAAa

2bgrA 331 336 5.297 -67.30 -27.00 -81.00 -29.20 -100.30 -16.90 67.60 20.50 YDESSGRW *** EETTTTEE AAAa

2bgrA 484 489 5.278 -69.30 -33.50 -58.90 -41.00 -126.10 19.90 45.40 44.00 HSSVNDKG *** EETTTTEE AAAa

2bgrA 675 680 4.793 -51.40 -36.30 -73.90 -19.00 -113.40 -93.80 -133.00 22.10 PTPEDNLD *** STTTTHH AAAA

2bjiA 1124 1129 5.054 -60.80 -42.00 -87.20 -16.30 -98.90 -12.70 53.10 51.00 YSCLEDKM *** EETTTTEE AAAa

2bk7B 186 191 5.383 -55.80 -38.30 -63.30 -34.90 -104.90 -10.10 65.80 10.00 HNIASQEI *** EETTTTEE AAAa

2bkqA 109 114 5.471 -63.30 -37.20 -51.40 -42.10 -120.20 28.70 39.20 46.10 YLQDKNSF *** EETTTTEE AAAa

2cb5A 333 338 5.419 -65.90 -38.20 -63.50 -42.60 -87.30 -14.50 70.60 43.30 FNSKLGLS *** EETTTTEE AAAa

2dri_ 53 58 6.061 -61.20 -20.20 -113.90 12.90 -100.10 6.30 82.10 24.80 DLTVRGTK *** HHTTTTEE AAAa

2ebn_ 52 57 5.072 -66.30 -30.50 -60.90 -57.10 -97.70 9.70 43.70 51.40 YDAANDKV *** EETTTTEE AAAa

2ebn_ 223 228 4.888 -76.20 -17.50 -84.20 -52.40 -73.10 -17.90 72.10 47.40 QEFNQGRY *** EETTTTB AAAa

2igd_ 51 56 5.421 -69.90 -23.60 -69.80 -35.30 -109.50 -2.50 57.60 41.10 YDDATKTF *** EETTTTEE AAAa

2mcm_ 76 81 5.360 81.90 -177.80 -84.90 -18.60 -91.10 -3.80 64.60 58.10 VVGADGTP *** EETTTTEE pAAa

2pgd_ 222 227 5.886 -69.00 -6.80 -101.90 4.40 -107.40 -155.20 -61.90 -22.20 EWNKTELD *** HHTTTTT AABA

2por_ 89 94 5.982 83.50 152.40 -66.00 -25.60 -95.40 -0.60 64.70 84.00 EVGYTDLD *** TTTT pAAa

2sak_ 93 98 5.418 -66.60 -32.60 -79.70 -16.90 -110.90 -15.30 56.10 39.50 YDKNKKKE *** EETTTTEE AAAa

2sli_ 198 203 5.157 -63.00 -43.20 -57.30 -41.90 -98.60 3.20 49.20 53.60 ADAANKQC *** EETTTTEE AAAa

2sli_ 381 386 5.417 -60.80 -20.40 -75.00 -48.30 -94.80 -13.20 70.00 9.80 EDKLTKRI *** EETTTTEE AAAa

2sli_ 442 447 5.368 -68.40 -31.00 -66.90 -40.60 -100.40 -11.20 59.20 29.00 YNDATNQP *** EETTTTEE AAAa

3cla_ 97 102 5.536 -65.20 -33.10 -65.00 -41.50 -103.40 -11.40 58.60 44.30 FHQETETF *** EETTTTEE AAAa

3lzt_ 53 58 5.147 81.50 -168.90 -61.50 -27.10 -109.50 9.50 50.50 53.50 DYGILQIN *** EETTTTEE pAAa

3lzt_ 59 64 6.721 -81.70 -2.80 -76.40 -62.30 -85.10 -31.00 -105.40 -45.30 INSRWWcN *** EETTTT B AAAA

3tdt_ 167 172 4.600 -86.80 -46.20 -156.70 117.40 -65.50 155.40 -65.90 136.00 GVLEPLQA *** SBTTB S ABPP

7ahlA 29 34 5.319 -64.50 -44.90 -58.40 -49.90 -88.30 -8.60 59.50 53.90 YDKENGMH *** EETTTTEE AAAa

7ahlA 285 290 5.281 -71.00 -19.60 -83.10 -49.50 -85.70 -9.00 56.90 61.90 IDWEKEEM *** EETTTTEE AAAa

7odcA 321 326 5.225 -69.10 -19.30 -85.50 -18.10 -98.90 -128.40 -62.90 -29.60 DGVYGSFN *** TTTTTH AABA

(b) SCH turns

PDBID START END CA-CA DIHEDRAL ANGLES OF THE MIDDLE RESIDUES SEQ SSE CONFOR-

DIST (i-1) (i-1) MATION

-(i+6) -(i+6)

PHI1 PSI1 PHI2 PSI2 PHI3 PSI3 PHI4 PSI4

16pk_ 50 55 5.457 -59.80 -43.30 -61.10 -36.80 -81.70 -2.90 92.30 22.40 KVLTEGGS *** HHHHTT E AAAa

16pk_ 77 82 5.341 -62.30 -33.30 -63.30 -39.10 -72.90 -9.60 89.80 -1.10 KIRSTGGV *** HHHHTT AAAa

16pk_ 275 280 5.105 -61.30 -45.80 -56.90 -40.90 -84.00 1.20 63.90 32.40 KAEDRKVQ *** HHHHTT E AAAa

16pk_ 382 387 4.748 -62.00 -51.50 -63.40 -35.70 -88.70 5.40 74.30 17.00 AAELSGEA *** HHHHTT T AAAa

16pk_ 403 408 5.536 -63.10 -43.80 -66.90 -22.20 -77.90 -10.10 95.00 13.50 LELLEGKT *** HHHHTT AAAa

1a3aA 32 37 5.166 -66.90 -56.00 -54.00 -39.20 -75.80 -0.60 83.30 7.30 QLVKGGYV *** HHHHTTSB AAAa

1a4iA 134 139 4.848 -59.40 -44.00 -65.40 -22.10 -107.60 10.00 86.10 11.60 GRLARGDL *** HHHHTT AAAa

1a4iA 185 190 5.493 -62.60 -45.00 -77.80 -3.90 -98.40 13.70 74.30 21.70 LLLWNNAT *** HHHHTT E AAAa

1a68_ 124 129 5.058 -72.80 -47.20 -61.40 -34.80 -87.20 -3.90 78.80 27.30 YFYQSGGR *** HHHHTTS AAAa

1a8e_ 51 56 4.770 -72.10 -40.80 -64.30 -34.80 -88.00 7.00 68.60 31.30 RAIAANEA *** HHHHTTS AAAa

1a8e_ 194 199 5.400 -78.90 -52.50 -56.30 -38.40 -72.30 -18.80 94.50 12.60 KCLKDGAG *** HHHHTTS AAAa

1a8i_ 112 117 5.192 -65.70 -41.90 -63.20 -24.80 -84.90 0.20 86.10 15.30 ATYQLGLD *** HHHHTT AAAa

1a8i_ 147 152 5.524 -72.00 -41.10 -64.30 -27.20 -94.50 11.00 66.60 29.50 SMATLGLA *** HHHHTT AAAa

1a8i_ 681 686 5.438 -63.20 -40.70 -69.20 -24.50 -98.80 13.90 87.10 24.80 XFMLNGAL *** HHHHTT E AAAa

1a8l_ 71 76 4.768 -56.50 -41.70 -65.50 -32.70 -98.80 15.20 54.40 41.20 LAKRYRID *** HHHHTT AAAa

1a8l_ 163 168 5.700 -70.10 -43.50 -55.80 -43.20 -62.70 -13.10 96.80 3.10 ENTKAGKG *** HHHHTT AAAa

1a8l_ 185 190 5.201 -63.40 -36.40 -56.00 -35.50 -106.40 21.90 46.30 53.00 WADQYNVM *** HHHHTT AAAa

1a8vA 18 23 5.205 -63.50 -37.70 -61.50 -33.80 -74.70 -2.90 97.00 12.20 LGENMGLE *** HHHHTT AAAa

1aba_ 24 29 5.069 -62.20 -46.20 -64.10 -31.90 -81.90 4.10 55.70 44.30 LLTVKKQP *** HHHHTT AAAa

1afwA 107 112 5.638 -58.00 -49.70 -61.00 -34.80 -74.30 -5.70 103.40 -1.10 ACLASGIP *** HHHHTT AAAa

1afwA 138 143 4.941 -68.10 -44.10 -60.50 -37.60 -84.70 -0.10 87.30 2.50 NKIKVGQI *** HHHHTTS AAAa

1afwA 194 199 4.360 -67.00 -49.40 -57.40 -30.00 -124.10 23.20 45.50 73.80 VAANFKIS *** HHHHTT AAAa

1afwA 218 223 5.346 -64.90 -56.10 -54.90 -38.80 -72.60 -1.20 76.30 25.90 KAKNEGLF *** HHHHTTSS AAAa

1afwA 291 296 4.805 -64.80 -44.20 -60.40 -38.80 -89.80 0.90 54.00 55.80 VANQLNLP *** HHHHTT AAAa

1aho_ 26 31 5.330 -74.50 -27.20 -59.20 -37.10 -86.50 8.00 74.90 18.00 EdTKLKGE *** HHHHTT S AAAa

1ail_ 22 27 5.631 -62.90 -39.60 -67.70 -21.10 -88.70 6.00 62.10 38.40 QVVDQELG *** HHHHTT AAAa

1ajsA 94 99 5.329 -72.80 -40.60 -63.90 -40.00 -82.30 5.10 53.80 40.00 PALQEKRV *** HHHHTT E AAAa

1ajsA 147 152 5.846 -63.00 -45.70 -63.60 -33.40 -70.90 -12.30 111.10 -4.90 VFTTAGFK *** HHHHTT S AAAa

1ajsA 243 248 5.711 -61.80 -48.70 -60.10 -28.10 -81.10 2.30 82.60 20.10 YFVSEGFE *** HHHHTT AAAa

1ajsA 341 346 5.435 -77.20 -36.30 -67.70 -26.30 -86.60 -2.40 58.30 48.60 RLEALKTP *** HHHHTT S AAAa

1ako_ 47 52 6.041 -62.60 -42.10 -60.60 -32.30 -74.30 -8.10 94.20 20.10 EVAKLGYN *** HHHHTT E AAAa

1al3_ 137 142 4.959 -65.10 -41.40 -66.60 -40.10 -73.60 -11.00 83.00 16.90 EAVSKGNA *** HHHHTT AAAa

1amf_ 66 71 5.551 -61.40 -42.00 -64.60 -30.80 -85.10 11.30 61.10 31.40 YAVDKKAI *** HHHHTT B AAAa

1amf_ 133 138 5.085 -74.40 -39.60 -67.90 -30.20 -90.60 6.20 68.30 9.90 ALQKLGAW *** HHHHTT H AAAa

1amf_ 158 163 5.065 -75.40 -40.40 -61.10 -40.30 -83.90 8.60 54.00 37.00 ALVERNEA *** HHHHTTSS AAAa

1amf_ 225 230 5.540 -62.60 -43.70 -59.20 -29.50 -93.30 7.60 92.90 4.60 IFKRYGFT *** HHHHTT E AAAa

1anf_ 50 55 5.555 -71.60 -27.00 -60.80 -37.80 -79.20 6.20 81.70 7.80 QVAATGDG *** HHHHTT S AAAa

1anf_ 70 75 5.262 -58.60 -49.00 -61.10 -31.40 -81.80 -0.80 67.90 40.40 GYAQSGLL *** HHHHTT B AAAa

1anf_ 161 166 5.500 -76.90 -24.00 -48.30 -52.50 -91.90 14.60 127.00 -6.10 LIAADGGY *** HHHHTT E AAAa

1anf_ 198 203 5.140 -73.10 -53.60 -40.50 -50.60 -87.40 16.10 61.20 29.50 DLIKNKHM *** HHHHTTSS AAAa

1anf_ 216 221 5.151 -70.00 -37.30 -67.10 -27.20 -92.80 0.00 84.40 13.20 AAFNKGET *** HHHHTT E AAAa

1anf_ 349 354 4.973 -75.90 -38.80 -69.90 -36.50 -74.70 -11.30 74.60 17.20 INAASGRQ *** HHHHTTSS AAAa

1aq0A 113 118 4.954 -59.70 -52.60 -56.00 -28.70 -86.80 -4.50 83.90 22.90 ALVAAGLG *** HHHHTT T AAAa

1aru_ 65 70 5.218 -56.60 -45.10 -61.40 -34.30 -83.70 15.30 80.70 6.20 ALTAAGQF *** HHHHTTS AAAa

1atg_ 43 48 5.430 -63.90 -45.60 -60.80 -31.00 -86.40 23.20 76.60 13.60 AQIVNGAP *** HHHHTT AAAa

1atg_ 64 69 4.957 -64.00 -48.80 -61.60 -37.20 -85.10 -1.10 85.00 13.00 KLDNQGFA *** HHHHTT B AAAa

1atg_ 126 131 5.013 -64.20 -42.00 -58.30 -38.40 -84.60 -5.50 70.70 30.10 VLTHLGLL *** HHHHTT H AAAa

1atg_ 135 140 5.357 -69.90 -45.50 -61.80 -37.80 -77.90 -2.70 59.00 35.90 KLTAQERI *** HHHHTT E AAAa

1atg_ 153 158 5.026 -73.70 -40.60 -63.10 -33.00 -86.50 -9.10 84.80 1.40 SQTASGAA *** HHHHTTSS AAAa

1atg_ 223 228 5.826 -67.20 -35.00 -64.50 -26.00 -84.90 4.10 93.70 1.20 IIKAAGYV *** HHHHTT B AAAa

1atzA 1052 1057 4.823 -55.50 -52.80 -66.90 -19.00 -98.30 9.10 67.80 28.80 AARSNRVT *** HHHHTTEE AAAa

1ay7B 21 26 4.943 -75.00 -51.30 -73.10 -25.60 -102.20 0.40 57.00 52.40 LKKELALP *** HHHHTT AAAa

1ay7B 60 65 4.966 -57.60 -80.60 -37.90 -40.00 -89.80 -7.40 77.20 10.20 SKQLTENG *** HHHHTTTH AAAa

1ay7B 77 82 5.679 -66.80 -39.20 -68.90 -18.60 -88.00 2.10 98.50 7.40 EAKAEGCD *** HHHHTT AAAa

1ayfA 33 38 5.084 -69.20 -49.40 -62.10 -40.20 -89.40 8.70 48.90 45.30 VVVQNNLD *** HHHHTT AAAa

1ayl_ 461 466 4.911 -70.00 -50.70 -66.30 -40.40 -80.70 -3.10 88.40 5.00 DAILNGSL *** HHHHTTTG AAAa

1az9_ 219 224 5.030 -58.10 -45.50 -64.10 -18.80 -97.10 7.60 89.20 11.00 EFNRHGAR *** HHHHTT AAAa

1az9_ 338 343 5.113 -63.60 -44.80 -59.30 -28.60 -102.70 17.00 47.90 47.60 ELIAQNAH *** HHHHTTTT AAAa

1b0nB 22 27 5.364 -62.80 -46.60 -65.60 -23.60 -86.00 9.90 62.60 32.90 EAKEANIS *** HHHHTT AAAa

1b25A 101 106 5.958 -72.50 -40.60 -70.30 -14.00 -85.00 -10.20 107.00 16.20 HLRRAGYD *** HHHHTT S AAAa

1b25A 164 169 5.149 -69.70 -45.80 -56.40 -40.40 -87.30 10.30 56.90 37.10 PAGENLVK *** HHHHTT T AAAa

1b25A 191 196 5.537 -65.70 -34.20 -66.50 -19.10 -95.20 0.80 63.80 26.60 VMGSKKLK *** HHHHTTEE AAAa

1b25A 247 252 4.701 -63.80 -40.10 -63.90 -31.60 -104.10 19.10 52.80 37.90 WCNTNYAL *** HHHHTT S AAAa

1b25A 352 357 5.014 -70.50 -43.60 -64.30 -36.50 -82.20 0.50 78.60 1.50 EAVERGIL *** HHHHTTSS AAAa

1b25A 376 381 4.707 -70.50 -47.40 -59.90 -39.90 -96.80 1.20 47.90 51.80 LDIAYRKG *** HHHHTT H AAAa

1b25A 538 543 5.255 -55.50 -56.40 -61.60 -21.70 -89.30 3.80 58.60 45.30 WVREFNGK *** HHHHTTT AAAa

1b25A 600 605 4.714 -65.40 -38.00 -61.40 -40.90 -89.30 8.10 67.10 38.40 TLKELDLD *** HHHHTT T AAAa

1b4pA 20 25 5.126 -66.30 -52.00 -54.10 -45.60 -81.00 -4.60 66.90 28.70 LLEYTDSS *** HHHHTT AAAa

1b4pA 80 85 4.559 -62.60 -43.70 -66.40 -28.10 -115.00 12.20 57.90 46.10 LARKHHLC *** HHHHTT S AAAa

1b5eA 18 23 5.120 -70.60 -40.40 -61.60 -31.70 -95.90 4.10 55.90 41.50 LALKEKDF *** HHHHTT AAAa

1b5qA 23 28 5.113 -60.20 -40.20 -61.60 -25.20 -85.30 0.60 94.40 2.50 RLSEAGIT *** HHHHTT AAAa

1b5qA 270 275 6.556 -66.70 -32.10 -74.20 -18.60 -85.00 -7.60 75.60 6.80 GVLQSDLI *** HHHHTTSS AAAa

1b65A 368 373 5.029 -62.00 -43.30 -60.40 -25.30 -106.90 14.50 64.90 38.20 VLRQYGRL *** HHHHTT AAAa

1b68A 122 127 7.533 -63.60 -37.20 -65.40 -25.50 -72.50 -14.70 52.70 44.70 EVQAMLGQ *** HHHHTTT AAAa

1b6a_ 420 425 5.612 -58.20 -41.90 -69.50 -21.90 -83.60 4.40 87.80 9.50 WLDRLGES *** HHHHTT AAAa

1b6a_ 435 440 5.281 -70.50 -42.60 -67.30 -31.10 -74.30 -12.00 77.00 12.60 NLCDLGIV *** HHHHTTSE AAAa

1b8oA 139 144 5.133 -62.80 -32.00 -59.70 -30.20 -91.80 -6.70 104.50 -6.80 LPGFSGEN *** HHHHTT AAAa

1b8oA 178 183 7.325 -60.00 -33.30 -71.50 -20.40 -78.80 -15.30 55.20 48.20 TWKQMGEQ *** HHHHTT S AAAa

1b8oA 209 214 5.883 -64.90 -41.90 -65.10 -17.60 -80.30 -8.20 97.50 15.60 LLRNLGAD *** HHHHTT S AAAa

1b8oA 228 233 5.065 -64.10 -42.30 -55.10 -42.80 -92.40 14.40 86.50 9.70 VARHCGLR *** HHHHTT E AAAa

1b93A 77 82 5.025 -62.70 -45.00 -64.50 -24.30 -93.10 4.80 73.60 22.10 ALISEGKI *** HHHHTT AAAa

1b93A 109 114 5.887 -72.80 -46.10 -60.50 -44.10 -85.30 7.70 50.10 47.50 LATVWNIP *** HHHHTT AAAa

1bd3A 180 185 5.492 -64.20 -31.60 -71.00 -22.80 -88.00 -4.70 101.70 4.70 VLLRLGVK *** HHHHTT AAAa

1bdmA 22 27 5.692 -65.30 -48.80 -63.30 -22.70 -81.80 -5.50 88.70 17.30 FRIAAGEM *** HHHHTTTT AAAa

1bdmA 326 331 5.572 -55.20 -47.50 -46.30 -44.90 -78.00 6.90 51.80 50.50 QVKALGLI *** HHHHTT AAAa

1bf2_ 66 71 5.604 -67.00 -34.40 -65.70 -30.80 -76.10 -8.00 109.50 -0.70 SIKAAGIT *** HHHHTT AAAa

1bf2_ 282 287 5.046 -60.10 -33.40 -68.40 -31.90 -82.90 -1.60 81.90 17.10 AFHNAGIK *** HHHHTT E AAAa

1bf2_ 483 488 4.669 -56.70 -55.30 -61.80 -31.80 -92.10 12.80 90.90 19.70 ANDFSGSS *** HHHHTT H AAAa

1bm8_ 40 45 6.111 -65.00 -35.80 -65.20 -30.50 -77.40 3.30 69.80 27.70 ILKAANFA *** HHHHTT AAAa

1bm8_ 87 92 5.096 -69.20 -49.80 -52.40 -48.20 -94.70 25.70 44.70 54.60 LAEKFSVY *** HHHHTT H AAAa

1bqcA 41 46 5.936 -64.10 -38.50 -59.90 -25.30 -93.40 6.20 103.00 -4.60 DIKSHGAN *** HHHHTT S AAAa

1bqcA 74 79 4.893 -63.70 -42.60 -63.90 -29.20 -96.50 9.90 57.60 48.90 LaKQNRLI *** HHHHTT E AAAa

1bqcA 151 156 5.504 -64.10 -41.40 -70.20 -25.70 -82.00 0.20 94.30 9.50 RLRAAGFE *** HHHHTT AAAa

1brt_ 46 51 5.655 -66.70 -37.70 -70.40 -27.90 -83.50 -2.10 98.00 24.30 ALLDAGYR *** HHHHTT E AAAa

1brt_ 165 170 5.029 -88.60 -21.90 -68.10 -36.00 -107.30 -0.50 49.90 59.70 FNDFYNLD *** HHHHTTHH AAAa

1bs0A 194 199 4.954 -61.10 -43.60 -59.60 -20.80 -117.10 17.60 63.00 33.50 VTQQHNGW *** HHHHTT E AAAa

1bs0A 220 225 5.151 -52.70 -54.60 -45.60 -42.50 -99.30 17.10 51.10 47.50 SCWLQKVK *** HHHHTT AAAa

1bs0A 338 343 5.070 -62.00 -46.40 -54.90 -30.80 -98.90 8.20 98.20 14.10 KLRQQGCW *** HHHHTTEE AAAa

1bupA 162 167 5.271 -60.60 -41.10 -60.80 -36.60 -75.70 -0.30 87.80 15.40 AGTIAGLN *** HHHHTT E AAAa

1bupA 180 185 5.354 -64.30 -36.30 -56.40 -37.50 -90.50 9.30 70.20 20.60 AAIAYGLD *** HHHHTTTT AAAa

1bupA 351 356 5.133 -62.60 -45.70 -55.00 -39.20 -91.90 22.30 54.10 40.30 LQDFFNGK *** HHHHTTT AAAa

1bx4A 28 33 4.957 -63.90 -39.40 -58.70 -35.10 -109.80 13.40 52.30 45.40 FLDKYSLK *** HHHHTT AAAa

1bx4A 103 108 5.689 -68.00 -45.10 -62.80 -23.90 -84.40 -2.20 66.70 27.20 KAAEAHVD *** HHHHTT E AAAa

1bx4A 186 191 4.581 -63.80 -41.10 -68.90 -34.60 -103.10 22.20 43.30 44.30 HASENNRI *** HHHHTT E AAAa

1bx4A 231 236 5.085 -58.90 -53.90 -63.60 -27.60 -93.00 1.80 70.40 26.60 FAREQGFE *** HHHHTT AAAa

1by2_ 44 49 5.189 -63.90 -38.00 -63.70 -36.80 -82.80 6.80 89.20 17.90 VbRALGFE *** HHHHTT S AAAa

1byi_ 26 31 5.569 -62.00 -44.20 -60.60 -32.60 -77.40 -8.60 104.00 10.10 AAKAAGYR *** HHHHTT AAAa

1byi_ 162 167 5.814 -63.10 -38.90 -63.80 -25.50 -73.80 -6.50 88.20 9.00 VIQHAGLT *** HHHHTT AAAa

1byrA 49 54 5.430 -55.00 -48.00 -58.10 -30.00 -74.00 -9.30 102.10 -1.80 AAKKRGVD *** HHHHTT E AAAa

1byrA 77 82 5.926 -62.60 -40.60 -57.70 -22.80 -88.00 0.20 84.30 11.50 YIANSGIP *** HHHHTT AAAa

1c1dA 67 72 4.876 -57.30 -51.40 -68.00 -21.70 -97.30 8.50 58.20 39.30 KMAVSNLP *** HHHHTT S AAAa

1c1dA 165 170 5.439 -62.50 -51.60 -58.60 -34.00 -73.50 -11.90 85.90 25.90 TVAHRGLG *** HHHHTT AAAa

1c1dA 194 199 5.524 -62.80 -43.90 -62.20 -32.40 -77.90 1.00 99.50 0.80 LAAEAGAQ *** HHHHTT E AAAa

1c1dA 214 219 5.365 -63.40 -37.50 -62.30 -30.60 -83.60 4.60 100.60 -3.00 HAVALGHT *** HHHHTT E AAAa

1c1dA 274 279 5.123 -61.70 -46.20 -65.60 -32.30 -82.40 -3.80 79.60 18.90 ILHARGIL *** HHHHTT E AAAa

1c3cA 34 39 5.100 -63.80 -42.70 -68.80 -25.30 -88.20 3.80 71.60 24.40 AYEELGMI *** HHHHTTSS AAAa

1c3cA 199 204 5.036 -59.20 -37.60 -63.10 -30.50 -87.10 -2.80 83.20 19.20 ALSYLGLK *** HHHHTT E AAAa

1c3cA 361 366 5.842 -63.90 -46.10 -52.80 -37.10 -88.10 22.70 124.30 -50.10 KLIEKGLT *** HHHHTT AAAa

1c3pA 35 40 4.953 -70.90 -47.30 -57.30 -34.70 -85.70 -1.60 55.90 43.40 FKDAMNLI *** HHHHTT AAAa

1c3pA 116 121 5.424 -61.40 -41.30 -69.80 -15.10 -86.60 -8.30 99.80 7.40 EEFLKGNV *** HHHHTT E AAAa

1c3pA 154 159 5.637 -59.80 -40.90 -64.60 -28.70 -76.60 -7.60 106.20 1.60 YLRKKGFK *** HHHHTT AAAa

1cb8A 187 192 5.054 -66.10 -49.20 -60.00 -44.70 -89.30 13.70 44.80 35.80 RALLTSDE *** HHHHTT H AAAa

1cb8A 324 329 5.025 -68.50 -52.70 -64.10 -38.00 -81.80 -11.10 62.60 59.00 IARTDSTV *** HHHHTTSS AAAa

1ccwA 27 32 5.591 -65.10 -44.80 -58.70 -30.70 -78.40 -7.20 105.00 6.10 AFTNAGFN *** HHHHTT E AAAa

1ccwA 78 83 5.099 -57.50 -51.10 -60.40 -30.20 -81.20 -3.70 86.80 18.20 KCDEAGLE *** HHHHTT T AAAa

1ccwA 108 113 5.806 -61.40 -42.00 -59.70 -30.10 -72.80 -8.60 109.50 7.80 RFKDMGYD *** HHHHTT S AAAa

1ccwB 162 167 5.508 -72.20 -41.00 -66.30 -22.80 -73.20 -2.20 82.40 13.70 IIHAGGWT *** HHHHTT AAAa

1ccwB 204 209 5.101 -63.30 -35.10 -66.20 -25.30 -91.00 2.80 90.70 14.80 FYEEQGVH *** HHHHTT AAAa

1ccwB 240 245 5.536 -58.90 -35.50 -63.90 -30.30 -86.70 1.50 107.90 4.90 LAAEQGVK *** HHHHTT AAAa

1ccwB 276 281 5.115 -64.00 -39.60 -60.40 -30.50 -100.30 11.30 89.50 11.10 YLKAYGYN *** HHHHTT AAAa

1ccwB 384 389 4.834 -65.00 -49.80 -60.50 -33.70 -90.60 7.50 47.00 45.00 MFELGKGD *** HHHHTTT AAAa

1cf3A 298 303 4.997 -61.00 -40.40 -60.70 -32.20 -85.50 0.70 87.20 21.80 ILEYSGIG *** HHHHTTBS AAAa

1cf3A 380 385 5.485 -66.70 -41.60 -62.70 -28.00 -84.10 -2.20 84.40 11.80 EAVARGGF *** HHHHTTS AAAa

1chd_ 294 299 5.408 -55.70 -44.40 -72.50 -17.80 -87.80 0.50 95.40 0.50 AMYQAGAW *** HHHHTT E AAAa

1chd_ 318 323 6.191 -61.60 -47.10 -65.00 -21.90 -81.10 -8.30 85.10 13.20 EAINMGGV *** HHHHTT AAAa

1chmA 35 40 4.905 -68.20 -36.30 -66.90 -19.60 -100.10 -1.80 49.20 41.10 HLAAENID *** HHHHTT S AAAa

1ci9A 29 34 5.420 -66.00 -46.70 -55.90 -44.60 -81.20 -0.50 59.10 38.40 QALRERRL *** HHHHTTS AAAa

1ci9A 334 339 5.286 -55.00 -54.20 -57.60 -29.90 -89.40 -5.10 60.20 39.90 DPAAAGTP *** HHHHT S AAAa

1cmcA 43 48 5.167 -58.30 -58.40 -58.10 -31.50 -86.30 -0.40 63.40 34.00 RRQVNNLR *** HHHHTTBS AAAa

1cmcA 92 97 5.065 -56.10 -51.40 -57.90 -39.60 -79.70 2.30 78.50 21.10 IMREMGIN *** HHHHTT AAAa

1cnv_ 72 77 5.346 -64.10 -39.70 -64.20 -28.10 -92.10 14.10 90.40 4.00 EaQRMGVK *** HHHHTT E AAAa

1cnzA 329 334 5.418 -66.70 -35.40 -59.30 -47.90 -63.60 -16.10 91.60 18.40 RALEEGVR *** HHHHTT AAAa

1cozA 81 86 4.822 -76.40 -56.00 -54.80 -35.90 -106.40 8.40 49.00 54.40 DIIDHNID *** HHHHTT S AAAa

1cpo_ 35 40 5.163 -64.60 -37.50 -64.30 -28.20 -97.00 0.60 76.50 12.10 ALANHGYI *** HHHHTTSS AAAa

1cruA 186 191 4.856 -57.30 -48.00 -60.50 -28.80 -95.90 9.30 59.40 36.90 QELNGKDY *** HHHHTT AAAa

1crzA 41 46 6.570 -65.20 -42.70 -54.80 -30.30 -77.60 -10.70 72.50 -8.10 DLRNSGKF *** HHHHTTSE AAAa

1cs0A 37 42 5.702 -58.90 -49.00 -54.80 -32.40 -82.50 2.40 75.50 38.10 ALREEGYR *** HHHHTT E AAAa

1cs0A 102 107 5.172 -73.30 -52.70 -51.60 -55.80 -60.10 -5.80 70.80 11.40 ELERQGVL *** HHHHTTHH AAAa

1cs0A 136 141 5.073 -59.40 -36.80 -63.00 -28.70 -102.90 13.70 65.50 30.60 AMKKIGLE *** HHHHTT B AAAa

1cs0A 325 330 5.609 -74.10 -35.00 -54.70 -30.40 -101.60 40.40 84.90 -19.10 AKLAVGYT *** HHHHTT AADa

1cs0A 442 447 5.267 -66.40 -42.90 -65.00 -27.20 -84.90 -1.70 104.80 -0.90 DAFRAGLS *** HHHHTTB AAAa

1cs0A 492 497 5.781 -60.40 -43.80 -57.90 -23.70 -92.30 5.40 84.30 12.00 QLKRKGFA *** HHHHTT AAAa

1cs0A 589 594 5.408 -72.40 -39.60 -65.70 -36.70 -69.70 -8.50 100.20 -0.60 ALREDGYE *** HHHHTT E AAAa

1cs0A 654 659 5.170 -63.60 -44.40 -58.00 -34.30 -86.60 10.60 83.00 19.20 ALEAAGVP *** HHHHTT AAAa

1cs0A 682 687 5.158 -55.40 -45.80 -54.20 -49.50 -81.50 -1.50 57.50 58.40 AVERLKLK *** HHHHTT AAAa

1cs0A 817 822 5.235 -67.40 -50.10 -51.10 -56.30 -74.70 1.70 64.90 45.20 LAFELQVR *** HHHHTT AAAa

1cs0A 867 872 5.235 -62.40 -49.50 -54.90 -37.60 -80.70 -3.90 126.40 -22.80 ARVMAGKS *** HHHHTT AAAa

1cs0A 874 879 5.309 -56.40 -51.90 -56.40 -31.00 -91.70 -16.00 79.60 37.20 SLAEQGVT *** HHHHT AAAa

1cs0A 932 937 5.759 -67.30 -41.60 -60.50 -30.20 -78.70 4.30 74.70 28.30 AQLGSNST *** HHHHTT AAAa

1cs0A 981 986 4.761 -54.00 -47.40 -62.70 -19.60 -91.10 -9.50 95.00 8.60 VLGEAGIN *** HHHHTT AAAa

1cs0A 1004 1009 5.311 -62.80 -44.60 -61.10 -28.80 -93.70 16.10 73.40 16.80 DRIKNGEY *** HHHHTT AAAa

1cs0A 1033 1038 5.046 -71.20 -48.40 -58.60 -38.20 -93.80 7.30 66.10 32.70 SALQYKVH *** HHHHTT E AAAa

1cs1A 163 168 5.109 -58.50 -34.40 -58.50 -38.50 -98.50 15.00 72.00 17.50 LAREVGAV *** HHHHTT E AAAa

1ct5A 230 235 5.417 -61.20 -42.40 -64.70 -29.90 -97.30 19.60 94.20 24.00 EAIRQGTA *** HHHHTT S AAAa

1cuj_ 194 199 5.718 -68.60 -26.60 -82.30 -24.90 -129.80 -80.80 -66.10 -33.60 PDARGPAP *** HHHHTHHH AAAA

1cv8_ 74 79 5.216 -68.10 -37.50 -66.10 -22.80 -103.00 26.00 80.70 21.60 FGQTQGRS *** HHHHTT AAAa

1cv8_ 96 101 5.178 -65.50 -40.10 -64.40 -31.90 -88.50 10.30 58.60 46.60 NLTKNNKG *** HHHHTT AAAa

1cvrA 31 36 5.286 -65.30 -37.60 -59.60 -36.60 -93.50 11.00 97.80 13.40 WKNQRGLR *** HHHHTT E AAAa

1cvrA 296 301 5.401 -68.40 -41.80 -59.00 -39.00 -88.20 21.80 48.40 44.10 NEILCEKH *** HHHHTT S AAAa

1cxlA 60 65 5.555 -76.20 -41.40 -75.00 -29.60 -88.80 6.40 86.40 -3.00 NKINDGYL *** HHHHTTTT AAAa

1cxlA 125 130 4.790 -57.10 -39.70 -59.00 -26.30 -106.40 19.70 63.00 31.10 AAHAKNIK *** HHHHTT E AAAa

1cxlA 218 223 6.300 -62.10 -39.30 -68.00 -23.40 -80.30 -6.80 97.40 16.60 MWLDLGID *** HHHHTT AAAa

1cxpC 371 376 4.657 -68.40 -47.20 -105.00 -7.80 -118.40 44.70 100.60 5.00 RVVLEGGI *** HHHHTT S AADa

1cxpC 400 405 6.441 -56.70 -33.90 -105.70 -17.10 -90.70 -26.20 -116.10 -30.70 VDEIRERL *** HHHHT B AAAA

1cxpC 425 430 5.384 -71.30 -42.70 -52.90 -37.90 -87.10 1.40 62.50 32.20 RSRDHGLP *** HHHHTT AAAa

1cxpC 437 442 4.903 -61.70 -46.10 -55.20 -42.60 -80.10 4.30 68.10 25.50 WRRFfGLP *** HHHHTT AAAa

1cxqA 171 176 5.026 -61.40 -36.50 -57.10 -36.90 -83.90 3.30 86.70 11.40 LAEGDGFM *** HHHHTT S AAAa

1cy5A 75 80 4.654 -59.40 -39.90 -91.10 -20.80 -117.10 22.90 105.80 19.80 ALLHEGYK *** HHHHTT H AAAa

1czpA 30 35 5.113 -62.00 -44.10 -58.90 -32.50 -91.90 7.90 101.20 11.20 AAEEQGYD *** HHHHTT AAAa

1czpA 70 75 5.634 -66.20 -42.10 -64.60 -29.00 -79.20 -4.70 106.70 4.20 DQIEAGYV *** HHHHTTEE AAAa

1d02A 18 23 6.354 -59.10 -46.10 -64.80 -27.50 -75.70 7.50 60.10 -2.80 GLKASGAE *** HHHHTT AAAa

1d1qA 31 36 5.047 -66.10 -43.40 -61.30 -33.80 -92.20 5.40 56.70 39.90 EVEKANLE *** HHHHTT G AAAa

1d1qA 65 70 4.867 -60.40 -40.70 -60.50 -32.50 -96.00 4.10 71.30 28.40 ICKQHGVK *** HHHHTT AAAa

1d2nA 614 619 5.248 -79.70 -37.90 -61.50 -35.40 -87.00 6.00 52.20 41.10 IERLLDYV *** HHHHTT B AAAa

1d2nA 660 665 5.238 -64.00 -47.60 -62.40 -27.20 -85.00 -1.60 67.40 24.80 VLQEMEML *** HHHHTT T AAAa

1d3gA 62 67 5.159 -67.60 -39.60 -64.50 -27.80 -89.10 -3.60 72.80 26.60 RFTSLGLL *** HHHHTT AAAa

1d3gA 171 176 5.117 -57.70 -47.00 -59.80 -32.80 -78.30 1.60 95.20 22.80 KLTEDGLP *** HHHHTT AAAa

1d3gA 321 326 4.990 -64.90 -43.30 -65.00 -25.40 -103.50 11.40 59.00 28.00 MYALTQGR *** HHHHTTT AAAa

1d3gA 378 383 5.093 -63.80 -44.30 -62.10 -31.20 -99.20 6.50 83.20 24.70 LLKEQGFG *** HHHHTT S AAAa

1d3vA 31 36 5.819 -65.90 -43.50 -61.70 -26.50 -79.60 4.20 86.10 21.40 ALRKAGLV *** HHHHTTHH AAAa

1d3vA 87 92 5.680 -67.10 -36.80 -62.40 -23.90 -98.60 17.40 67.40 38.30 ETQKNGTI *** HHHHTT E AAAa

1d3vA 301 306 5.834 -65.70 -38.60 -62.60 -21.30 -113.40 31.60 111.50 -22.00 TLSCFGTK *** HHHHTT AAAa

1d3yA 89 94 4.794 -69.50 -39.90 -68.60 -25.30 -105.80 13.90 51.00 49.00 QLLETDDF *** HHHHTT AAAa

1d3yA 205 210 5.201 -62.20 -51.20 -63.00 -33.20 -90.90 8.20 48.80 52.30 RLNAERFW *** HHHHTTHH AAAa

1d3yA 211 216 4.711 -76.30 -50.60 -54.70 -41.40 -113.00 13.70 43.20 60.00 FWDKHNCI *** HHHHTTEE AAAa

1d3yA 291 296 5.182 -71.70 -51.30 -61.60 -37.50 -98.70 5.40 51.00 49.10 DIIDYDLP *** HHHHTT AAAa

1d4oA 33 38 5.308 -80.90 -24.90 -54.70 -36.80 -93.30 -1.90 56.10 37.10 GLCAAKAQ *** HHHHTTTH AAAa

1d4oA 49 54 5.863 -63.70 -37.30 -67.70 -24.70 -82.80 10.20 81.90 3.80 MLSEQGKK *** HHHHTT E AAAa

1d5tA 23 28 5.380 -61.20 -48.50 -66.20 -27.70 -79.10 -0.50 100.60 8.10 IMSVNGKK *** HHHHTT AAAa

1d5tA 53 58 4.897 -63.00 -45.90 -56.70 -36.80 -92.80 14.50 64.10 34.60 LYKRFQLL *** HHHHTT T AAAa

1d5tA 172 177 5.507 -61.80 -36.90 -56.70 -32.20 -103.00 6.40 48.40 52.10 VYRKFDLG *** HHHHTT AAAa

1d7uA 272 277 6.994 -56.50 -42.70 -54.40 -29.40 -100.40 -8.80 51.90 55.70 SKTLGAGL *** HHHHTTS AAAa

1d7uA 293 298 5.049 -62.50 -54.70 -52.80 -34.90 -84.30 -2.20 86.70 27.40 RAHELGYL *** HHHHTT AAAa

1d7uA 321 326 4.944 -69.70 -56.10 -56.10 -50.00 -79.80 10.80 51.80 46.80 VVQRDGLV *** HHHHTTHH AAAa

1d7uA 386 391 4.881 -56.10 -46.30 -55.40 -44.90 -66.70 -12.10 97.40 21.40 ECMNLGLS *** HHHHTTEE AAAa

1d8wA 24 29 4.912 -60.90 -33.30 -61.00 -25.60 -100.10 21.40 77.80 21.00 RFAAVGID *** HHHHTT AAAa

1d8wA 130 135 4.786 -53.60 -44.00 -66.90 -32.70 -97.50 9.80 62.70 46.30 WAKANQLG *** HHHHTT E AAAa

1d8wA 320 325 5.135 -71.30 -39.40 -58.20 -36.80 -96.10 12.30 51.50 41.40 EIVRHDLF *** HHHHTTGG AAAa

1d8wA 400 405 4.956 -62.30 -42.80 -63.60 -20.80 -109.70 22.10 48.00 49.10 YCQRHDTP *** HHHHTT AAAa

1dbwA 23 28 5.561 -59.40 -45.00 -65.10 -21.90 -89.60 3.60 93.20 3.80 MLTMNGFA *** HHHHTT E AAAa

1dbwA 69 74 5.511 -55.40 -42.70 -65.50 -30.00 -76.90 -4.70 65.00 34.70 NLGDLKIN *** HHHHTT AAAa

1dbwA 93 98 5.609 -69.10 -39.40 -62.80 -34.20 -77.00 -5.80 105.40 7.30 EAMKAGAV *** HHHHTT S AAAa

1dc1A 73 78 6.077 -61.50 -48.60 -60.20 -24.80 -75.50 3.80 95.90 12.70 LLYASGIS *** HHHHTT AAAa

1dc1A 150 155 5.037 -62.20 -48.30 -63.50 -28.50 -89.50 3.70 56.90 46.30 ALDIANIP *** HHHHTT AAAa

1dc1A 268 273 4.969 -56.60 -36.80 -53.90 -35.10 -93.40 8.90 75.50 17.80 KFSEKGLS *** HHHHTT AAAa

1dciA 312 317 4.951 -59.80 -42.80 -63.10 -34.20 -92.30 2.60 62.60 40.80 QAAMEKKD *** HHHHTT AAAa

1dcs_ 12 17 5.455 -58.10 -42.90 -66.50 -21.80 -87.30 0.70 86.40 18.10 AELQQGLH *** HHHHTT S AAAa

1dcs_ 138 143 4.860 -66.50 -41.80 -64.50 -27.20 -101.30 5.80 65.70 30.40 VLRATGTE *** HHHHTT AAAa

1dcs_ 231 236 4.760 -64.30 -54.00 -64.00 -32.80 -89.10 4.10 66.90 35.20 ATLVTGGQ *** HHHHTTS AAAa

1dcs_ 278 283 5.501 -63.00 -30.60 -72.00 -21.70 -114.50 20.40 120.10 -5.30 LARECGFD *** HHHHTT AAAa

1dekA 59 64 5.557 -71.00 -42.10 -62.30 -23.40 -77.40 -17.90 87.20 15.80 RKEFEGID *** HHHHTTTT AAAa

1dekA 185 190 5.224 -66.80 -38.30 -59.70 -37.60 -84.70 4.80 77.30 24.30 AARAMGAT *** HHHHTT E AAAa

1dfmA 155 160 5.212 -64.40 -39.30 -58.50 -35.40 -100.50 16.20 53.80 36.30 SLAEYNVF *** HHHHTT AAAa

1din_ 51 56 5.605 -64.70 -39.60 -61.80 -30.60 -86.40 5.60 97.20 11.20 WLVDQGYA *** HHHHTT E AAAa

1dj0A 213 218 5.324 -63.30 -45.10 -65.50 -28.40 -85.30 1.20 71.50 21.80 MEVGAHNQ *** HHHHTTSS AAAa

1dk0A 138 143 4.781 -59.20 -38.00 -60.80 -28.30 -96.50 8.70 63.40 30.40 YGLMSGDT *** HHHHTT AAAa

1dljA 37 42 4.838 -56.30 -47.20 -62.50 -12.60 -112.10 18.40 74.80 24.00 DKINNGLS *** HHHHTT AAAa

1dljA 128 133 5.244 -79.30 -36.70 -65.10 -44.00 -97.70 0.40 49.10 57.70 MRQKFQTD *** HHHHTT S AAAa

1dljA 225 230 4.702 -66.60 -41.60 -63.80 -33.70 -99.70 10.20 56.10 45.50 YAESRKLN *** HHHHTT AAAa

1dlwA 3 8 5.715 -80.00 -42.90 -61.30 -27.90 -80.70 -14.70 115.40 -4.10 LFEQLGGQ *** HHHHTTSH AAAa

1dlwA 50 55 5.013 -62.80 -47.10 -63.80 -27.30 -90.60 6.30 95.20 19.40 LCAALGGP *** HHHHTT S AAAa

1dlwA 89 94 5.702 -63.20 -41.10 -65.40 -25.30 -82.30 -2.10 91.30 12.00 ALTGAGVA *** HHHHTT AAAa

1dmhA 16 21 5.274 -63.90 -50.10 -59.00 -33.20 -83.10 0.40 68.10 15.40 LRVASGLE *** HHHHTTTT AAAa

1dmhA 45 50 4.835 -71.80 -52.30 -57.70 -42.20 -91.00 0.80 50.30 52.80 AIEDLNIT *** HHHHTT AAAa

1dmhA 65 70 5.435 -71.90 -35.60 -60.80 -31.80 -96.40 9.90 49.80 42.00 QLGANQEA *** HHHHTT H AAAa

1dmhA 75 80 5.590 -63.50 -30.00 -65.50 -30.70 -94.10 13.70 81.10 -4.40 LSPGLGFD *** HHHHTTHH AAAa

1dmhA 92 97 5.080 -59.00 -40.80 -66.70 -29.20 -88.70 5.30 70.20 15.00 EDAALGIE *** HHHHTT AAAa

1dmhA 212 217 5.089 -60.60 -41.90 -64.60 -19.70 -98.50 2.70 69.20 27.90 LLNQLGRH *** HHHHTT AAAa

1dmhA 274 279 5.612 -64.80 -37.40 -65.20 -46.60 -84.10 8.70 38.30 63.90 AIKANDVE *** HHHHTT S AAAa

1dozA 27 32 5.141 -60.50 -46.80 -59.10 -40.90 -81.60 19.40 54.80 46.50 YTHIRRGR *** HHHHTTT AAAa

1dozA 47 52 5.413 -66.10 -41.40 -60.20 -36.70 -87.10 8.30 102.80 6.60 RYEAIGGI *** HHHHTT S AAAa

1dozA 100 105 5.193 -62.10 -44.50 -63.20 -39.60 -73.60 -4.60 96.40 7.30 EMHKDGIT *** HHHHTT AAAa

1dozA 305 310 4.986 -64.50 -38.10 -64.80 -27.80 -94.40 8.20 68.90 24.70 VLKKLGR# *** HHHHTT AAAa

1dp4A 226 231 5.046 -57.60 -48.60 -61.60 -29.50 -86.60 9.40 74.40 20.60 LALNAGLT *** HHHHTT AAAa

1dp4A 335 340 5.187 -58.10 -45.30 -55.40 -33.90 -96.30 20.30 92.00 10.80 ETLAQGGT *** HHHHTT AAAa

1dp7P 30 35 4.771 -62.20 -46.30 -63.80 -33.20 -95.60 10.30 55.60 46.30 HSQEQKLE *** HHHHTT AAAa

1dqaA 471 476 5.004 -54.70 -53.30 -52.30 -40.70 -106.20 38.30 17.60 52.30 LVNAKHIP *** HHHHTT AAAa

1dqaA 573 578 5.299 -64.10 -44.60 -68.60 -17.70 -92.20 9.80 90.50 5.20 AIGLGGGA *** HHHHTT B AAAa

1dqaA 740 745 5.377 -66.40 -43.20 -60.80 -38.70 -73.60 -10.90 72.80 45.00 GSAMAGSI *** HHHHTT S AAAa

1dqaA 761 766 4.650 -65.20 -37.20 -69.90 -35.30 -101.80 20.40 66.60 25.00 IYIACGQD *** HHHHTT AAAa

1dqsA 302 307 5.293 -63.30 -45.30 -55.50 -40.50 -79.00 -1.30 78.20 3.40 LARHLGIL *** HHHHTTSS AAAa

1dqsA 320 325 5.316 -62.10 -31.50 -68.20 -22.90 -96.00 1.60 90.60 9.50 CLAAYGLP *** HHHHTT AAAa

1dqzA 242 247 4.909 -64.70 -39.70 -61.10 -32.90 -86.60 8.60 92.20 8.20 TYAADGGR *** HHHHTT AAAa

1dusA 97 102 5.555 -73.50 -37.10 -64.60 -21.90 -109.10 15.20 52.70 46.40 NIKLNNLD *** HHHHTT T AAAa

1duvG 32 37 4.780 -66.00 -44.40 -64.90 -34.00 -91.20 2.00 68.70 22.90 ADKKSGKE *** HHHHTT AAAa

1duvG 66 71 4.892 -56.50 -48.60 -54.90 -32.40 -96.60 7.90 83.50 7.50 AAYDQGAR *** HHHHTT E AAAa

1duvG 205 210 4.953 -63.10 -42.00 -54.00 -30.40 -98.70 9.40 91.50 13.30 LAQQNGGN *** HHHHTT E AAAa

1duvG 289 294 4.527 -73.20 -40.70 -60.30 -38.80 -115.30 13.50 58.60 37.80 MAEEFGLH *** HHHHTT AAAa

1dvoA 108 113 4.825 -73.40 -40.50 -63.30 -38.10 -94.00 1.80 50.70 45.70 DVAQRNIP *** HHHHTT S AAAa

1dwkA 22 27 5.347 -69.40 -41.20 -64.40 -34.40 -87.50 -4.80 57.90 44.60 SKAKKDLS *** HHHHTT AAAa

1dwkA 45 50 5.014 -63.40 -46.00 -54.70 -39.10 -83.00 10.50 92.50 12.10 TAALLGQQ *** HHHHTTSS AAAa

1dxrC 177 182 4.383 -78.60 -8.10 -89.70 -15.60 -118.70 44.60 51.40 38.30 LAKYTAYS *** HHHHTTT AADa

1dxrH 107 112 5.457 -70.60 -44.80 -78.60 -39.10 -73.00 -11.70 55.00 60.80 NPLVDAVG *** HHHHT G AAAa

1dxrM 74 79 4.982 -63.90 -30.50 -66.10 -34.40 -100.80 16.50 57.70 40.90 MAAEVHFD *** HHHHTTT AAAa

1dxrM 135 140 4.781 -65.40 -40.10 -61.90 -33.80 -95.90 5.70 74.40 35.50 RARALGLG *** HHHHTT AAAa

1dxrM 188 193 4.930 -61.20 -50.50 -56.00 -35.60 -107.30 24.70 71.10 38.00 FSIRYGNF *** HHHHTT G AAAa

1dxrM 296 301 5.089 -61.30 -40.70 -60.00 -36.80 -98.90 12.40 77.80 7.80 WCVKHGAA *** HHHHTT AAAa

1dy5A 30 35 5.163 -70.50 -53.40 -65.10 -30.00 -89.60 14.80 65.90 37.00 MMKSRNLT *** HHHHTTSS AAAa

1e19A 40 45 6.175 -64.60 -31.60 -65.90 -29.30 -80.70 2.80 90.20 8.20 EIIARGYE *** HHHHTT E AAAa

1e19A 101 106 4.959 -57.70 -44.50 -70.00 -23.30 -90.90 9.10 72.80 28.80 ELRKRGME *** HHHHTT AAAa

1e19A 182 187 5.396 -61.50 -44.50 -64.40 -20.60 -92.40 6.70 78.30 22.00 KLVERGVI *** HHHHTT E AAAa

1e19A 223 228 5.113 -62.70 -43.90 -69.30 -26.90 -100.10 9.70 55.20 49.90 LAEEVNAD *** HHHHTT S AAAa

1e19A 263 268 6.451 -58.00 -44.10 -63.90 -29.80 -70.30 -8.60 82.80 17.50 KYYEEGHF *** HHHHTT S AAAa

1e19A 302 307 5.327 -70.10 -41.90 -65.40 -31.30 -78.10 0.70 89.30 10.80 VEALEGKT *** HHHHTTSS AAAa

1e1hA 229 234 5.152 -73.60 -27.50 -77.60 -28.80 -90.60 -0.20 67.30 31.90 EHRLYGIA *** HHHHTT AAAa

1e25A 83 88 5.239 -72.90 -44.00 -58.20 -35.50 -83.20 0.90 75.90 6.30 HQVDQGKL *** HHHHTTSS AAAa

1e25A 152 157 5.827 -60.80 -39.70 -59.90 -22.80 -88.20 5.00 84.10 11.10 YIQSMGIK *** HHHHTT AAAa

1e25A 192 197 6.757 -66.00 -49.20 -59.40 -33.20 -73.20 -6.50 49.90 32.70 KKFEQKTQ *** HHHHTT S AAAa

1e4cP 17 22 5.490 -70.90 -40.40 -64.20 -33.40 -78.20 -4.00 80.00 16.70 EMTRLGLN *** HHHHTTS AAAa

1e58A 15 20 5.134 -59.50 -50.00 -58.30 -45.20 -84.10 0.70 61.90 50.50 QWNKENRF *** HHHHTTB AAAa

1e58A 44 49 5.301 -60.50 -50.20 -57.40 -34.60 -87.10 4.00 87.50 17.90 LLKEEGYS *** HHHHTT AAAa

1e58A 171 176 6.124 -57.60 -42.10 -67.30 -25.90 -74.60 -3.00 103.20 11.40 PRMKSGER *** HHHHTT AAAa

1e58A 192 197 4.920 -69.40 -46.30 -69.10 -36.00 -89.70 0.40 55.60 41.80 VKYLDNMS *** HHHHTT AAAa

1e5kA 149 154 5.791 -74.80 -36.10 -65.80 -24.20 -77.60 -3.80 93.60 10.10 EYLQAGER *** HHHHTT AAAa

1e5kA 161 166 5.468 -75.70 -44.40 -56.30 -36.30 -76.80 -5.90 83.90 23.80 FMRLAGGH *** HHHHTT E AAAa

1e5pA 130 135 4.961 -60.90 -49.30 -58.20 -37.50 -91.00 -0.40 62.10 44.80 FAHEKKIP *** HHHHTT AAAa

1e6bA 27 32 5.269 -64.90 -44.00 -66.10 -28.80 -85.00 -7.20 70.70 30.90 ALALKGLD *** HHHHTT AAAa

1e6cA 165 170 4.627 -71.50 -61.40 -42.10 -49.30 -93.70 -3.30 67.70 66.30 LMQTMRL# *** HHHHTT AAAa

1e6iA 380 385 4.985 -58.90 -49.70 -66.20 -26.70 -92.20 13.40 64.40 31.70 IKLESNKY *** HHHHTT AAAa

1e6uA 94 99 4.847 -60.90 -44.10 -66.50 -19.90 -110.30 18.80 59.80 34.70 AAHQNDVN *** HHHHTT AAAa

1e6uA 290 295 5.944 -65.30 -40.30 -61.60 -27.50 -79.10 -2.20 87.50 22.00 RLHQLGWY *** HHHHTT AAAa

1e7lA 16 21 4.974 -67.40 -43.80 -62.60 -36.20 -85.40 5.30 53.00 36.20 FYDAQNGK *** HHHHTTTB AAAa

1e85A 28 33 4.751 -68.00 -42.90 -72.90 -19.20 -90.40 0.50 77.00 11.60 TPVVKGQA *** HHHHTTSS AAAa

1ec7A 329 334 4.402 -62.10 -50.20 -61.20 -31.30 -102.80 0.50 81.50 18.10 MCHEFGLT *** HHHHTT AAAa

1ec7A 414 419 5.244 -58.40 -51.70 -47.00 -46.40 -89.60 179.70 -104.70 65.00 LYQKHGLG *** HHHHTT AAPD

1ed8A 136 141 5.296 -58.40 -43.80 -66.60 -22.00 -84.00 -10.80 102.20 2.20 MAKAAGLA *** HHHHTT E AAAa

1ed8A 229 234 5.453 -62.40 -44.90 -58.90 -31.30 -85.10 0.70 91.30 12.10 QAQARGYQ *** HHHHTT E AAAa

1ed8A 329 334 5.380 -64.10 -42.00 -62.50 -35.70 -78.80 1.50 48.00 47.50 KQDHAANP *** HHHHTT H AAAa

1edg_ 330 335 4.925 -59.50 -40.30 -61.40 -21.80 -109.00 15.60 69.60 20.10 QAKARGIL *** HHHHTT E AAAa

1eexA 211 216 6.249 -67.10 -36.60 -63.70 -27.20 -81.60 -8.10 94.20 12.50 KLGMLGHT *** HHHHTT AAAa

1eexA 232 237 6.055 -62.50 -45.80 -67.10 -18.50 -78.90 3.20 59.70 42.40 VFTDGDDT *** HHHHTT AAAa

1eexA 249 254 5.264 -61.50 -31.20 -71.30 -16.40 -98.60 8.80 72.20 15.40 SYASRGLK *** HHHHTT E AAAa

1eexA 265 270 5.445 -62.00 -53.90 -54.10 -36.40 -100.30 15.70 88.50 3.90 SEVQMGYA *** HHHHTT AAAa

1eexA 323 328 5.770 -64.60 -38.50 -64.40 -26.70 -87.10 16.30 63.30 42.30 ICSSLDLE *** HHHHTT E AAAa

1eexA 423 428 4.670 -61.80 -49.90 -63.20 -37.30 -87.80 4.30 67.70 28.20 VFAGMGLP *** HHHHTT S AAAa

1eexA 463 468 4.839 -66.50 -54.00 -69.00 -23.00 -106.70 10.30 52.10 50.50 EIINKNRN *** HHHHTT AAAa

1eexA 477 482 4.854 -61.40 -41.90 -78.30 -12.70 -110.10 15.00 94.10 13.70 ALAQGGFT *** HHHHTT H AAAa

1eexB 95 100 5.689 -65.30 -34.00 -54.60 -30.70 -94.60 13.80 69.20 34.30 GIEEEGIK *** HHHHTT E AAAa

1eexB 174 179 5.442 -63.00 -44.90 -51.10 -44.50 -84.70 12.50 65.20 25.50 ARYAKRES *** HHHHTT AAAa

1eexG 68 73 4.779 -66.50 -46.00 -69.80 -35.90 -85.50 1.90 61.40 20.30 ENVLSNKV *** HHHHTTSS AAAa

1eexG 93 98 5.210 -63.30 -40.40 -64.30 -30.10 -80.20 2.90 84.30 24.30 IAKDAGRD *** HHHHTT H AAAa

1eexG 163 168 4.864 -65.30 -56.60 -57.30 -43.90 -94.90 4.90 59.40 62.30 LYVERKKL *** HHHHTT S AAAa

1ef8A 133 138 5.135 -69.50 -17.30 -71.50 -32.60 -105.80 -8.10 76.60 29.50 TPVNLGVP *** HHHHT AAAa

1ef8A 172 177 5.161 -68.80 -46.50 -59.10 -39.50 -81.90 -4.20 101.10 1.90 RALAVGIL *** HHHHTTS AAAa

1ef8A 249 254 4.969 -65.30 -53.60 -61.10 -40.20 -75.50 -3.70 48.60 50.50 NAFLEKRK *** HHHHTTS AAAa

1ejdA 57 62 5.335 -63.30 -39.80 -64.70 -26.70 -83.50 -7.10 106.40 9.00 LLTQLGTK *** HHHHTT E AAAa

1ejdA 129 134 5.521 -61.70 -39.50 -64.70 -27.80 -81.70 3.30 103.10 1.80 GLEKLGAE *** HHHHTT E AAAa

1ejdA 198 203 5.399 -65.80 -35.70 -62.30 -28.90 -83.70 -8.30 117.50 3.30 FLVALGAK *** HHHHTT E AAAa

1ejdA 266 271 5.598 -64.20 -36.60 -65.90 -28.70 -79.20 -3.50 104.00 2.70 KLREAGAD *** HHHHTT E AAAa

1ejdA 338 343 5.515 -64.00 -35.80 -70.30 -17.40 -95.60 9.50 98.40 5.70 ELIRMGAH *** HHHHTT E AAAa

1el5A 22 27 5.359 -62.60 -38.10 -56.40 -27.60 -98.30 12.00 76.40 12.60 QLAKQGVK *** HHHHTT AAAa

1el5A 108 113 4.739 -64.60 -46.20 -61.90 -30.80 -110.60 15.20 48.00 48.30 AAKEHSLT *** HHHHTT AAAa

1el5A 160 165 5.332 -59.70 -39.40 -59.90 -32.30 -90.30 13.70 103.80 -7.70 LAEARGAK *** HHHHTT E AAAa

1epxA 106 111 4.885 -61.10 -39.30 -69.50 -19.40 -93.70 6.70 77.10 13.60 YLTARGVV *** HHHHTT E AAAa

1epxA 147 152 5.394 -62.60 -43.80 -62.70 -29.10 -83.70 -2.30 111.10 0.70 AYYKKGCR *** HHHHTT AAAa

1epxA 226 231 5.655 -69.40 -40.40 -60.60 -43.50 -80.30 -1.50 71.50 39.40 ALQRHGVI *** HHHHTT AAAa

1epxA 346 351 5.021 -59.00 -39.20 -63.20 -32.10 -89.30 -2.00 76.60 18.10 SMAQLGKY *** HHHHTT AAAa

1eqcA 83 88 5.627 -61.70 -43.60 -60.00 -28.80 -82.80 -0.60 91.00 13.30 QISNLGLN *** HHHHTT AAAa

1eqcA 123 128 4.677 -59.10 -47.90 -61.80 -28.40 -105.10 12.60 60.70 38.80 WARKNNIR *** HHHHTT E AAAa

1eqcA 214 219 5.995 -66.40 -33.60 -62.60 -23.40 -79.00 -6.90 92.60 8.40 SLRQTGSV *** HHHHTT AAAa

1eqcA 378 383 5.212 -67.30 -44.90 -64.50 -28.70 -92.50 6.90 76.60 15.10 TLTYNGLF *** HHHHTT S AAAa

1es9A 58 63 6.156 -66.00 -37.10 -69.70 -25.40 -117.80 -50.90 -104.80 -26.70 IWRELFSP *** HHHHTGG AAAA

1esgA 130 135 5.167 -66.90 -48.80 -60.10 -34.30 -87.60 3.10 71.00 14.60 LGLKHGEI *** HHHHTTS AAAa

1eswA 36 41 5.064 -65.10 -44.40 -65.20 -28.60 -79.00 -6.40 86.60 8.10 FLKEAGGR *** HHHHTT AAAa

1eswA 77 82 5.438 -63.90 -34.00 -68.00 -25.20 -94.20 4.40 82.10 5.00 PLAERGYV *** HHHHTTS AAAa

1eswA 148 153 4.802 -67.70 -29.60 -81.70 -16.70 -114.20 41.90 54.30 32.20 LKGAHGGL *** HHHHTTT AADa

1eswA 162 167 5.264 -80.90 -41.70 -59.10 -42.80 -90.90 5.90 54.60 47.50 LPLRKREE *** HHHHTT H AAAa

1eswA 203 208 4.806 -61.90 -39.40 -64.00 -27.80 -93.40 6.70 73.80 19.40 EAEALGIR *** HHHHTT E AAAa

1eswA 268 273 4.644 -61.10 -44.90 -57.70 -32.50 -93.00 19.40 63.10 29.00 VLEREGFS *** HHHHTTTH AAAa

1eswA 353 358 4.860 -62.10 -39.50 -53.90 -37.00 -104.10 13.50 64.80 30.20 LRDRFGLP *** HHHHTT AAAa

1eswA 418 423 5.002 -60.50 -35.90 -62.30 -29.00 -97.40 15.10 73.30 24.30 YLADWGIT *** HHHHTT AAAa

1eswA 452 457 5.435 -81.20 -31.20 -63.60 -37.00 -85.90 -3.90 50.70 46.20 VQDVLALG *** HHHHTT AAAa

1eswA 494 499 4.781 -64.80 -44.50 -65.80 -27.10 -103.20 12.50 57.60 52.40 MAEATERL *** HHHHTT AAAa

1eu1A 254 259 4.861 -64.70 -48.20 -66.60 -26.30 -97.80 6.00 57.50 42.70 TLYSEDLH *** HHHHTT S AAAa

1eu1A 277 282 5.037 -75.10 -44.10 -58.30 -41.30 -84.40 -10.80 86.00 6.00 AAYLTGES *** HHHHTTTT AAAa

1eu1A 519 524 5.209 -61.30 -42.30 -64.20 -26.80 -89.00 -0.30 79.30 24.60 LAERLGKG *** HHHHTT H AAAa

1eu1A 549 554 4.975 -58.10 -41.20 -67.30 -21.40 -110.00 18.20 58.90 40.10 QAEFKNVA *** HHHHTT AAAa

1euvA 553 558 4.675 -72.10 -45.10 -63.90 -32.40 -86.50 3.40 59.30 47.60 VMEESKHT *** HHHHTTSS AAAa

1euvA 590 595 5.026 -63.00 -40.40 -63.00 -31.00 -91.90 3.50 49.40 48.20 LYGSADAP *** HHHHTT AAAa

1euvA 614 619 5.069 -64.70 -49.30 -67.40 -26.30 -96.10 13.00 50.80 56.00 HLILTDAL *** HHHHTTTT AAAa

1evfA 72 77 4.852 -61.00 -43.30 -58.10 -27.00 -106.20 16.70 57.20 38.50 YLHENNVT *** HHHHTT AAAa

1evfA 189 194 4.770 -61.10 -42.00 -67.60 -26.20 -101.90 7.70 56.80 37.70 MAQQXDLE *** HHHHTT E AAAa

1ewfA 270 275 5.925 -66.40 -42.80 -65.30 -24.70 -79.00 -8.50 77.20 17.90 VYQEAGVL *** HHHHTT S AAAa

1ex0A 242 247 5.408 -57.10 -40.00 -69.30 -22.80 -78.10 -7.80 56.60 44.10 VMDRAQMD *** HHHHTT AAAa

1ey4A 103 108 5.197 -69.70 -41.20 -62.40 -28.40 -87.40 0.00 70.30 29.10 ALVRQGLA *** HHHHTTS AAAa

1ey4A 132 137 5.540 -71.50 -37.70 -64.70 -38.00 -81.80 -2.60 51.20 47.30 QAKKEKLN *** HHHHTT G AAAa

1eyeA 38 43 5.536 -62.40 -41.80 -58.20 -39.90 -70.80 -5.50 116.30 -2.70 AMAAAGAG *** HHHHTT S AAAa

1eyeA 76 81 5.449 -60.20 -36.40 -68.10 -22.50 -90.60 14.50 77.20 8.90 ELAAQGIT *** HHHHTT AAAa

1eyeA 96 101 5.442 -61.80 -43.10 -58.30 -25.80 -100.80 18.20 109.80 -6.90 AALQNGAQ *** HHHHTT AAAa

1eyeA 164 169 5.544 -59.50 -46.50 -57.10 -24.80 -84.20 -1.40 100.20 5.90 DAVAAGVD *** HHHHTT AAAa

1eyeA 244 249 5.228 -60.70 -45.10 -61.40 -28.40 -104.00 18.60 87.40 8.20 LAALHGAW *** HHHHTT S AAAa

1ezwA 26 31 5.324 -57.50 -45.60 -62.90 -24.30 -97.80 12.00 93.90 2.70 VAEDNGFE *** HHHHTT AAAa

1ezwA 84 89 4.760 -64.70 -51.40 -59.50 -35.10 -84.30 0.70 73.30 30.20 LDWISGGR *** HHHHTTS AAAa

1ezwA 140 145 5.391 -64.60 -46.90 -66.10 -24.30 -79.70 -5.40 104.80 -2.40 YQYLEGGP *** HHHHTT AAAa

1ezwA 214 219 4.756 -59.90 -40.60 -65.10 -18.90 -103.30 8.90 71.50 24.70 GAKEAGRS *** HHHHTT AAAa

1ezwA 261 266 5.054 -63.50 -46.40 -52.00 -38.40 -100.60 11.70 54.30 41.70 VLERHGID *** HHHHTT AAAa

1ezwA 314 319 5.540 -61.30 -38.50 -63.50 -29.50 -83.30 2.20 106.80 -2.70 ELLKAGVT *** HHHHTT AAAa

1f0lA 103 108 4.755 -61.40 -50.10 -75.50 -25.90 -92.20 4.20 61.30 45.80 IKKELGLS *** HHHHTT AAAa

1f1eA 92 97 5.281 -59.80 -45.00 -62.60 -28.10 -80.00 -1.30 102.30 6.20 ILKRAGIE *** HHHHTT AAAa

1f1eA 127 132 5.046 -52.90 -40.40 -64.30 -29.00 -98.50 15.00 54.80 44.60 YADEDGRK *** HHHHTT S AAAa

1f20A 1299 1304 5.396 -62.60 -45.60 -59.70 -26.40 -93.80 10.10 86.80 7.20 QAKNKGVF *** HHHHTTSE AAAa

1f20A 1384 1389 4.983 -63.60 -51.40 -54.70 -37.80 -86.50 7.50 57.80 44.40 RLRDDNRY *** HHHHTT E AAAa

1f46A 32 37 5.822 -68.30 -38.20 -62.10 -35.70 -73.60 -3.60 97.70 3.20 SIQQAGFI *** HHHHTTEE AAAa

1f60A 66 71 6.373 -61.80 -43.50 -57.80 -32.10 -72.10 -16.80 84.20 19.70 AERERGIT *** HHHHTT AAAa

1f60A 140 145 5.081 -64.50 -45.70 -60.20 -38.80 -81.10 2.70 89.00 16.70 LAFTLGVR *** HHHHTT AAAa

1f74A 196 201 6.027 -67.80 -28.70 -65.40 -25.10 -94.70 8.40 87.40 21.90 PAASLGVD *** HHHHTT AAAa

1f74A 222 227 5.029 -65.00 -41.90 -60.70 -31.80 -81.10 -4.10 78.20 16.60 ELTKAGKL *** HHHHTT H AAAa

1f74A 258 263 5.198 -60.90 -34.10 -72.10 -19.10 -96.50 6.70 86.00 17.80 LLKLEGVD *** HHHHTT AAAa

1f86A 79 84 5.026 -67.90 -37.60 -64.90 -14.00 -97.10 11.00 75.90 21.70 YWKALGIS *** HHHHTT AAAa

1f8aB 99 104 4.912 -72.60 -44.40 -65.80 -34.00 -79.70 -2.80 72.60 14.80 QKIKSGEE *** HHHHTTSS AAAa

1f9yA 77 82 5.206 -62.00 -46.80 -63.30 -15.70 -103.40 20.40 79.40 16.10 IELQQGRV *** HHHHTT AAAa

1fc6A 188 193 5.582 -62.30 -43.80 -59.90 -24.50 -94.50 16.50 92.40 -0.30 PAEKAGAR *** HHHHTT AAAa

1fc6A 300 305 6.251 -69.30 -40.00 -61.70 -33.50 -73.50 -10.50 67.10 39.20 ELSKQGVA *** HHHHTT S AAAa

1fc6A 381 386 5.096 -67.00 -54.10 -62.00 -38.90 -73.80 -7.90 67.10 8.90 ALKDSKRG *** HHHHTTSE AAAa

1fcjA 103 108 5.998 -70.20 -38.70 -60.30 -30.20 -81.00 1.90 99.30 1.30 LLKALGAN *** HHHHTT E AAAa

1fcjA 161 166 4.916 -67.80 -40.20 -68.70 -26.90 -103.30 6.70 49.70 42.70 IWEDTDGQ *** HHHHTTT AAAa

1fcjA 212 217 4.949 -59.00 -42.60 -73.00 -19.00 -92.40 -0.40 78.20 11.80 AQALAGEE *** HHHHTT AAAa

1fcqA 213 218 7.783 -59.30 -33.00 -89.00 -54.30 -68.40 -29.20 -74.90 -23.00 MSWLFESE *** HHHHTT AAAA

1fcqA 290 295 5.947 -71.80 -43.30 -60.20 -33.60 -76.70 -14.70 98.80 21.40 KITDLGAD *** HHHHTT S AAAa

1fd3A 8 13 5.722 -66.10 -35.20 -57.50 -37.80 -75.30 -2.20 89.30 11.10 TaLKSGAI *** HHHHTT E AAAa

1fdr_ 159 164 6.739 -61.90 -46.40 -57.90 -35.00 -87.50 -1.10 46.60 46.60 LEKRYEGK *** HHHHTTTS AAAa

1fipA 67 72 4.601 -66.90 -40.20 -67.60 -37.00 -88.50 -3.60 52.00 39.90 VMQYTRGN *** HHHHTTT AAAa

1fipA 92 97 4.771 -72.00 -39.70 -50.40 -37.90 -98.80 9.60 57.70 42.40 KLKKYGMN *** HHHHTT AAAa

1fiuA 16 21 4.969 -72.80 -50.60 -59.10 -32.10 -87.10 2.80 63.00 23.10 KLLDGNIL *** HHHHTTSS AAAa

1fiuA 48 53 5.349 -65.10 -43.50 -65.90 -18.40 -96.30 1.60 63.70 34.40 IADLLHSE *** HHHHTT AAAa

1fiuA 268 273 4.953 -73.10 -48.10 -68.30 -26.70 -85.40 11.50 56.10 38.10 IMVNGKRL *** HHHHTTSE AAAa

1fj2A 98 103 5.620 -61.10 -45.90 -63.70 -21.40 -84.90 5.70 108.70 -5.20 QEVKNGIP *** HHHHTT AAAa

1fk5A 16 21 5.401 -65.30 -40.50 -62.20 -23.00 -88.10 -5.70 103.00 5.70 ISYARGQG *** HHHHTT S AAAa

1fk5A 72 77 4.920 -64.60 -38.80 -69.60 -22.40 -93.20 -0.90 74.40 26.20 IPSKcGVS *** HHHHTT AAAa

1fkmA 291 296 5.164 -71.90 -45.20 -66.10 -34.50 -81.40 -4.80 85.90 24.50 WKLLIGYL *** HHHHTT S AAAa

1fkmA 469 474 4.731 -59.90 -47.30 -62.80 -32.60 -96.00 8.30 51.40 43.20 HFQNEHVE *** HHHHTT AAAa

1fkmA 483 488 4.328 -75.70 -47.20 -59.00 -37.80 -113.00 18.20 48.10 49.70 RWXNCLLX *** HHHHTTTG AAAa

1fn9A 174 179 4.858 -65.40 -44.60 -65.30 -27.20 -110.30 14.60 49.60 46.90 MIDSSDLI *** HHHHTT E AAAa

1fn9A 190 195 5.652 -62.80 -38.20 -87.70 -30.00 -152.10 102.30 149.40 -58.30 SHAFNGVK *** HHHHTTS AABx

1fo8A 374 379 5.069 -71.10 -43.30 -58.90 -41.00 -86.90 2.30 66.30 46.00 EKVRTNDR *** HHHHTT AAAa

1fo8A 400 405 4.790 -62.80 -42.30 -60.70 -31.60 -102.20 9.50 69.40 26.50 FAKALGVM *** HHHHTT AAAa

1fp3A 260 265 6.077 -45.60 -51.90 -63.60 -37.60 -69.20 9.00 31.40 35.70 HSSRSGDA *** HHHHTT H AAAa

1fs7A 190 195 4.884 -60.30 -48.40 -53.30 -32.30 -93.30 4.30 84.20 10.90 GLSAAGFK *** HHHHTT AAAa

1fs7A 255 260 5.187 -69.00 -36.30 -58.90 -41.70 -95.20 13.10 50.00 40.70 YYDEINFA *** HHHHTT AAAa

1fs7A 288 293 5.250 -63.60 -36.10 -70.40 -31.80 -82.10 -1.90 86.80 11.50 IHGQKGVS *** HHHHTT AAAa

1fs7A 432 437 5.042 -63.90 -43.10 -55.80 -36.70 -95.40 17.90 72.80 25.50 VLAKYGAI *** HHHHTT T AAAa

1fs7A 452 457 5.175 -72.20 -42.70 -64.80 -35.40 -84.30 0.20 54.80 49.00 AQKLAKVD *** HHHHTT AAAa

1fsjB 78 83 5.050 -69.20 -26.70 -63.30 -26.10 -95.70 4.30 89.50 7.30 SSVSKGYS *** HHHHTT AAAa

1fsjB 128 133 5.115 -62.70 -46.90 -54.60 -44.20 -95.80 21.70 50.60 51.40 HIDIHRGK *** HHHHTTT AAAa

1ft5A 43 48 4.941 -61.10 -43.30 -59.30 -34.10 -90.80 7.50 62.80 30.70 AKQKAKLD *** HHHHTT AAAa

1ft5A 121 126 5.479 -63.80 -34.80 -64.40 -26.90 -96.20 13.00 102.20 -2.30 DLAKKGQD *** HHHHTT AAAa

1fviA 155 160 5.517 -66.40 -34.10 -69.10 -23.70 -88.90 -0.60 97.00 7.80 DVLSKGFE *** HHHHTT AAAa

1fvkA 112 117 5.471 -61.70 -42.40 -66.50 -27.70 -77.60 -7.50 110.40 -1.10 VFINAGIK *** HHHHTT AAAa

1fvkA 142 147 5.284 -63.40 -43.80 -55.30 -32.80 -105.70 9.50 50.80 49.10 AAADVQLR *** HHHHTT AAAa

1fx2A 936 941 5.058 -72.70 -40.60 -58.50 -43.30 -93.70 10.10 57.10 32.90 LIGRYKCY *** HHHHTT E AAAa

1fxoA 43 48 5.374 -62.20 -43.90 -65.00 -23.20 -85.90 5.70 81.80 16.00 TLMLAGIR *** HHHHTT AAAa

1fxoA 206 211 5.004 -60.60 -42.40 -60.40 -31.10 -89.00 4.90 65.60 29.70 AYLERGQL *** HHHHTT E AAAa

1fxoA 257 262 5.132 -63.00 -45.30 -65.20 -34.30 -81.70 -3.00 67.50 25.00 IAYRQKWI *** HHHHTTSS AAAa

1fyeA 97 102 5.147 -66.60 -51.10 -63.20 -31.70 -84.00 -0.20 71.70 26.70 ESRERGLL *** HHHHTT H AAAa

1fyeA 109 114 5.886 -66.80 -41.50 -58.90 -32.90 -86.20 10.60 121.00 -8.40 DRVKRGAL *** HHHHTT E AAAa

1g0sA 179 184 4.852 -70.90 -43.40 -61.90 -38.80 -85.00 -1.90 81.90 7.10 QWVEEGKI *** HHHHTTSS AAAa

1g2rA 91 96 5.048 -66.00 -38.00 -68.20 -29.50 -86.70 1.90 70.60 24.20 KRRELGLE *** HHHHTT AAAa

1g3mA 59 64 4.479 -60.70 -39.00 -65.70 -33.80 -96.60 19.50 53.80 30.00 YMIYKEGD *** HHHHTTT AAAa

1g3mA 117 122 5.640 -72.10 -43.70 -59.50 -37.30 -89.70 0.80 57.20 39.30 SFWEKDCK *** HHHHTT E AAAa

1g3mA 160 165 4.998 -60.60 -47.50 -59.10 -31.70 -88.60 -1.50 71.20 20.50 EKFMQGQV *** HHHHTT S AAAa

1g3mA 207 212 5.316 -66.40 -40.10 -61.00 -35.90 -89.80 11.30 54.90 46.70 LIHFLERK *** HHHHTT AAAa

1g3p_ 179 184 5.241 -61.30 -51.50 -61.50 -31.70 -81.50 10.70 73.70 15.70 DAYWNGKF *** HHHHTTTT AAAa

1g5aA 172 177 4.961 -60.30 -40.10 -62.80 -31.70 -89.90 8.50 74.20 31.60 ALHEAGIS *** HHHHTT E AAAa

1g5aA 197 202 5.339 -65.90 -49.50 -62.50 -30.20 -77.70 -10.30 93.40 14.40 QRCAAGDP *** HHHHTT G AAAa

1g5aA 404 409 4.974 -58.10 -35.30 -63.70 -24.90 -98.20 7.70 76.00 24.90 DAAYLGIS *** HHHHTT AAAa

1g5aA 420 425 4.771 -60.10 -50.00 -74.70 -8.50 -110.80 19.60 59.10 47.80 NRFFVNRF *** HHHHTT S AAAa

1g5hA 47 52 4.831 -70.20 -43.30 -62.20 -38.30 -96.50 11.50 69.00 30.50 LCRRRHFL *** HHHHTTSB AAAa

1g5hA 171 176 4.663 -64.40 -41.40 -59.30 -37.10 -100.70 13.40 48.50 41.80 CLDLVNRK *** HHHHTTTB AAAa

1g5hA 380 385 5.108 -60.30 -47.00 -54.30 -28.90 -95.80 15.40 63.10 19.90 ELLENGIS *** HHHHTT AAAa

1g5hA 406 411 5.220 -63.20 -38.10 -73.30 -20.10 -99.60 4.30 67.20 19.20 KYDEXSVL *** HHHHTT S AAAa

1g5tA 52 57 5.302 -64.70 -43.50 -58.70 -25.10 -92.40 11.60 79.60 6.60 RAVGHGKN *** HHHHTT AAAa

1g5tA 132 137 5.205 -77.30 -39.40 -62.50 -28.40 -104.30 11.60 57.00 38.30 YMVAYDYL *** HHHHTTSS AAAa

1g61A 2088 2093 4.839 -61.80 -46.20 -57.30 -29.20 -101.30 10.70 54.30 39.80 FLKENNLD *** HHHHTT AAAa

1g6sA 57 62 5.263 -56.30 -49.00 -61.20 -29.90 -82.90 -2.90 94.60 11.50 ALTALGVS *** HHHHTT E AAAa

1g6sA 133 138 5.400 -65.80 -38.80 -58.90 -28.50 -88.70 4.50 112.00 -12.30 ALRLGGAK *** HHHHTT AAAa

1g6sA 208 213 5.272 -64.20 -40.20 -57.10 -36.40 -91.80 19.30 88.00 12.70 LMKTFGVE *** HHHHTT AAAa

1g6sA 352 357 5.720 -64.70 -29.60 -64.20 -26.60 -98.30 5.10 115.60 -6.50 ELRKVGAE *** HHHHTT E AAAa

1g8kA 207 212 5.316 -66.50 -43.40 -64.80 -28.60 -77.00 -9.40 104.10 6.70 ATREMGIG *** HHHHTT AAAa

1g8kA 404 409 5.135 -66.20 -43.50 -62.20 -28.00 -103.40 12.90 54.90 42.20 LVIATHNV *** HHHHTT S AAAa

1g8kA 443 448 5.431 -64.00 -43.00 -61.00 -35.80 -78.70 -0.30 98.90 5.60 QELIKGKG *** HHHHTT AAAa

1g8kA 578 583 5.070 -57.00 -53.10 -62.10 -19.70 -94.90 8.60 64.20 30.60 MYQKDGKA *** HHHHTT H AAAa

1g8kA 602 607 6.263 -64.20 -36.90 -79.80 -22.30 -113.70 -80.20 -50.00 -49.20 AFNDGFRR *** HHHHTGGG AAAA

1g8kA 735 740 5.170 -57.80 -44.10 -61.50 -42.70 -83.10 1.90 56.80 43.00 DCKQLDVT *** HHHHTT AAAa

1g8mA 25 30 5.809 -65.90 -37.50 -66.00 -23.60 -79.40 -6.90 97.20 7.50 SLNALGLG *** HHHHTT E AAAa

1g8mA 42 47 5.612 -68.40 -39.10 -57.70 -27.00 -87.30 7.30 78.50 11.00 ALRDAGLP *** HHHHTT AAAa

1g8mA 89 94 4.910 -70.60 -46.70 -57.70 -40.10 -88.20 -5.60 60.20 41.70 DXNKQDFS *** HHHHTT AAAa

1g8mA 285 290 5.166 -67.40 -47.20 -58.70 -39.30 -87.30 7.90 57.70 38.20 EAQVCXVH *** HHHHTT T AAAa

1g8qA 169 174 5.825 -64.80 -45.00 -63.00 -32.20 -81.40 16.30 55.50 39.10 SVLKNNLb *** HHHHTT S AAAa

1g9gA 596 601 5.126 -61.00 -38.00 -66.00 -23.70 -97.70 10.40 65.60 33.40 AALQAGQV *** HHHHTT AAAa

1ga6A 14 19 4.465 -67.30 -41.30 -60.20 -43.90 -102.80 21.20 59.10 44.90 FPTIYDAS *** HHHHTT T AAAa

1ga6A 49 54 4.926 -65.20 -45.10 -61.60 -37.30 -86.70 3.50 68.10 23.50 FTSANGLA *** HHHHTT AAAa

1ga6A 89 94 5.105 -61.10 -48.70 -64.30 -32.60 -70.70 -1.90 84.70 19.30 IVGSAGGA *** HHHHTTS AAAa

1ga6A 141 146 4.942 -69.40 -42.20 -61.80 -34.20 -103.60 7.00 85.70 -3.00 DANADGTL *** HHHHTTHH AAAa

1ga6A 157 162 5.109 -59.70 -41.90 -65.00 -30.10 -88.90 2.00 87.60 14.10 TAAAQGQT *** HHHHTT E AAAa

1ga6A 301 306 4.370 -61.30 -35.70 -69.00 -16.30 -124.90 27.60 53.80 33.60 LQSANSNS *** HHHHTTT AAAa

1ga8A 140 145 5.486 -66.60 -27.70 -62.70 -30.10 -101.50 24.40 81.40 12.00 YKQKIGXA *** HHHHTT AAAa

1gai_ 66 71 5.564 -59.80 -50.10 -62.00 -24.30 -89.10 9.40 115.00 -6.70 DLFRNGDT *** HHHHTT G AAAa

1gai_ 142 147 4.883 -59.50 -48.80 -62.00 -31.70 -101.30 19.40 79.50 15.70 WLLDNGYT *** HHHHTT H AAAa

1gai_ 203 208 4.973 -63.90 -42.50 -59.20 -32.60 -92.60 8.30 83.40 19.10 FATAVGSS *** HHHHTT AAAa

1gai_ 427 432 4.867 -62.30 -38.80 -59.20 -33.60 -93.20 13.60 64.40 26.80 NNRRNSVV *** HHHHTT AAAa

1garA 20 25 5.058 -76.00 -31.70 -63.10 -48.10 -82.90 16.00 53.40 28.10 DACKTNKI *** HHHHTTSS AAAa

1garA 183 188 5.386 -59.50 -47.60 -64.00 -32.30 -82.20 11.00 69.20 28.80 SWFADGRL *** HHHHTT E AAAa

1gca_ 55 60 6.141 -66.60 -38.50 -62.80 -29.20 -84.30 2.70 79.70 27.90 VLLAKGVK *** HHHHTT S AAAa

1gca_ 80 85 5.081 -51.30 -45.20 -52.00 -39.60 -104.70 13.20 50.40 39.10 KARGQNVP *** HHHHTT AAAa

1gca_ 166 171 5.302 -64.40 -36.00 -70.30 -28.60 -79.00 7.10 85.20 17.00 ELNDKGIQ *** HHHHTT AAAa

1gca_ 222 227 4.954 -61.70 -41.50 -60.50 -35.90 -91.50 13.30 45.70 43.00 ALKAHNKS *** HHHHTT T AAAa

1gca_ 271 276 5.128 -64.80 -35.80 -62.00 -28.80 -92.80 4.20 75.10 14.10 KNLAEGKG *** HHHHTTS AAAa

1gci_ 8 13 5.840 -67.30 -38.80 -60.80 -38.60 -83.00 0.60 68.30 31.40 GISRVQAP *** HHHHTTHH AAAa

1gci_ 16 21 5.270 -57.50 -38.30 -67.80 -21.50 -90.50 10.80 85.10 14.80 AAHNRGLT *** HHHHTT S AAAa

1gci_ 114 119 5.178 -72.30 -21.50 -93.90 -3.70 -126.40 17.00 81.80 18.20 WAGNNGMH *** HHHHTT S AAAa

1gci_ 142 147 5.339 -61.10 -41.60 -62.20 -29.00 -87.80 11.20 89.60 7.20 SATSRGVL *** HHHHTT E AAAa

1gk9B 355 360 5.716 -59.30 -38.20 -81.00 -19.90 -120.50 -90.20 -59.10 -48.00 MLKRTVVA *** HHHHTHHH AAAA

1gk9B 527 532 4.687 -57.90 -52.30 -61.00 -34.90 -90.60 12.80 62.90 27.40 KMYENFGR *** HHHHTT AAAa

1gkmA 45 50 5.271 -68.80 -44.00 -62.80 -30.40 -91.90 1.00 55.80 37.50 QIIAEDRT *** HHHHTT AAAa

1gkmA 175 180 4.815 -64.50 -38.90 -55.20 -39.70 -86.70 3.60 85.00 17.70 ALAVAGLE *** HHHHTT AAAa

1gkmA 226 231 4.776 -64.90 -41.80 -58.70 -36.80 -102.70 15.10 65.70 38.00 SVEAVLGS *** HHHHTT AAAa

1gkmA 491 496 5.416 -64.30 -41.50 -64.00 -29.20 -82.30 2.80 79.30 25.80 ELLAKGSL *** HHHHTTTT AAAa

1gkpA 82 87 4.900 -64.70 -40.80 -59.20 -28.10 -94.40 2.80 92.30 8.20 AALMGGTT *** HHHHTTEE AAAa

1gkpA 141 146 6.051 -63.50 -44.90 -64.00 -19.90 -78.80 -1.10 113.10 3.50 EIVADGIS *** HHHHTT AAAa

1gkpA 197 202 5.198 -62.90 -39.20 -65.10 -18.50 -97.00 5.50 78.40 16.20 KLLSEGKT *** HHHHTT AAAa

1gkpA 252 257 5.270 -55.80 -49.80 -61.40 -32.90 -87.60 7.70 96.60 7.30 AAKARGVP *** HHHHTT AAAa

1gkpA 303 308 4.962 -65.50 -47.00 -57.60 -32.40 -91.80 0.00 80.00 10.20 DALAQGFI *** HHHHTTSS AAAa

1gkpA 372 377 4.544 -58.00 -51.30 -63.40 -31.50 -108.70 13.70 78.20 19.10 AAKLFGLF *** HHHHTT T AAAa

1gmxA 15 20 5.108 -66.60 -47.20 -60.30 -36.00 -79.30 -1.90 62.10 35.00 QKLQEKEA *** HHHHTT AAAa

1gnlA 51 56 5.141 -60.20 -41.80 -64.70 -29.10 -82.60 -3.10 83.10 19.60 RLRAEGKA *** HHHHTT AAAa

1gnlA 160 165 5.219 -62.10 -40.90 -56.10 -33.10 -93.80 10.80 92.10 9.30 HADVLGKH *** HHHHTT AAAa

1gnlA 384 389 4.954 -68.40 -46.30 -61.30 -35.90 -81.60 -9.60 84.80 0.60 DAVKSGAI *** HHHHTTS AAAa

1gnlA 466 471 5.235 -66.60 -48.10 -63.40 -39.50 -79.30 -5.20 64.00 36.40 LKEVFGLE *** HHHHTT S AAAa

1gnlA 497 502 5.525 -62.00 -41.10 -64.20 -28.90 -82.40 -0.10 89.40 14.70 ALLSLGVK *** HHHHTT AAAa

1gp6A 10 15 7.350 -64.10 -27.50 -101.70 -0.60 -73.60 -9.40 94.90 13.30 SLAKSGII *** HHHHTT S AAAa

1gp6A 188 193 5.038 -60.10 -37.50 -61.80 -31.40 -91.30 3.20 80.00 17.60 LSVGLGLE *** HHHHTTS AAAa

1gp6A 199 204 5.252 -63.10 -43.40 -65.20 -23.10 -102.50 14.50 117.80 -20.80 LEKEVGGL *** HHHHTTHH AAAa

1gp6A 276 281 5.020 -65.30 -49.50 -60.20 -35.30 -89.70 13.10 51.80 43.50 LEILSNGK *** HHHHTTTS AAAa

1gpr_ 33 38 5.324 -93.60 -50.40 -64.00 -24.40 -85.40 4.60 55.50 34.90 QVFSGKMM *** HHHHTTSS AAAa

1gpuA 78 83 5.255 -65.50 -51.40 -60.60 -32.80 -91.70 3.40 109.00 4.50 MLHLTGYD *** HHHHTT S AAAa

1gpuA 172 177 6.037 -66.90 -44.90 -60.30 -38.90 -77.60 -2.50 48.90 47.00 LAGHLKLG *** HHHHTT T AAAa

1gpuA 276 281 4.725 -56.30 -44.60 -54.20 -40.70 -90.00 8.40 80.10 21.40 LKSKFGFN *** HHHHTT AAAa

1gpuA 332 337 6.097 -57.60 -43.40 -65.60 -24.40 -80.60 -6.90 72.80 28.40 ARRLSGQL *** HHHHTT AAAa

1gpuA 384 389 5.873 -69.50 -13.60 -98.00 -37.70 -82.30 -24.70 60.90 46.50 LTPSNLTR *** HHHHT S AAAa

1gpuA 646 651 5.243 -65.50 -38.50 -55.70 -40.10 -87.60 4.30 77.70 14.10 VFKFFGFT *** HHHHTT S AAAa

1gqiA 134 139 5.117 -67.80 -46.90 -58.10 -31.60 -99.30 10.60 62.30 40.00 RLIQTQHA *** HHHHTT AAAa

1gqiA 248 253 5.616 -73.80 -39.40 -62.40 -36.20 -79.90 0.00 65.30 23.10 SPRAFGDV *** HHHHTTS AAAa

1gqzA 84 89 4.916 -70.30 -53.60 -61.20 -37.10 -86.10 -3.10 72.00 22.40 FVTLKGLT *** HHHHTTS AAAa

1gsa_ 28 33 5.516 -71.00 -42.20 -56.90 -31.60 -86.20 -8.80 98.60 17.90 EAQRRGYE *** HHHHTT E AAAa

1gsa_ 105 110 5.746 -60.70 -34.00 -68.80 -22.40 -88.20 8.90 97.70 8.10 RAEEKGTL *** HHHHTT E AAAa

1gsa_ 262 267 4.855 -67.20 -45.70 -59.70 -41.50 -89.40 8.70 70.70 23.10 TLKEKGLI *** HHHHTT AAAa

1gsjA 52 57 4.999 -60.10 -55.20 -59.20 -25.20 -91.00 7.80 59.50 21.30 LXKGLNLP *** HHHHTT AAAa

1gsjA 92 97 4.920 -61.90 -42.50 -57.10 -30.10 -102.30 21.80 56.40 32.00 WAKKHQIA *** HHHHTT AAAa

1gsjA 136 141 5.372 -65.30 -41.90 -65.70 -27.90 -87.00 2.70 89.30 14.10 SLLENGYL *** HHHHTT E AAAa

1gsjA 204 209 5.189 -68.40 -42.90 -62.10 -32.40 -87.10 -1.90 73.80 17.50 QLIEQGII *** HHHHTTSS AAAa

1gtkA 69 74 5.046 -63.50 -42.80 -67.80 -26.90 -109.10 21.40 63.90 31.50 VALLENRA *** HHHHTTS AAAa

1gtkA 158 163 5.431 -60.90 -51.30 -60.60 -34.70 -75.40 -3.90 96.00 5.00 SKLDNGEY *** HHHHTTS AAAa

1gtkA 174 179 4.838 -57.40 -47.00 -59.40 -37.60 -83.30 1.50 77.20 17.10 GLKRLGLE *** HHHHTT G AAAa

1gtvA 105 110 4.770 -61.60 -39.30 -65.00 -29.80 -94.00 2.70 60.20 37.20 SAARLHEN *** HHHHEEEE AAAa

1gtvA 182 187 5.118 -63.90 -46.30 -60.80 -32.50 -92.10 -8.80 73.00 37.60 ELAAQGWG *** HHHHEEEE AAAa

1gu7A 152 157 5.347 -66.60 -42.60 -64.20 -31.60 -84.10 9.90 84.40 4.90 QSKANGKP *** HHHHTT S AAAa

1gveA 31 36 6.061 -64.80 -36.10 -65.30 -26.30 -77.70 -3.70 96.50 -2.40 AFLQRGHT *** HHHHTT AAAa

1gveA 95 100 5.267 -67.20 -51.70 -56.60 -42.80 -77.70 -6.30 50.10 52.90 SLKRLQCP *** HHHHTT S AAAa

1gveA 127 132 5.678 -67.70 -42.20 -64.60 -30.60 -74.90 -8.00 85.90 20.80 QLHQEGKF *** HHHHTTSE AAAa

1gvfA 70 75 5.105 -69.60 -43.90 -58.40 -39.60 -102.80 9.50 49.70 51.60 YSTTYNMP *** HHHHTTS AAAa

1gvfA 93 98 6.055 -70.80 -40.60 -60.50 -32.60 -74.00 -6.80 122.20 -7.20 RKVHAGVR *** HHHHTT AAAa

1gvfA 124 129 4.755 -64.20 -31.30 -66.70 -15.50 -110.30 8.20 57.90 43.20 FCHSQDCS *** HHHHTT E AAAa

1gvfA 221 226 5.579 -69.70 -38.70 -58.40 -29.70 -97.20 6.60 125.10 -0.80 RTIELGVT *** HHHHTTEE AAAa

1gvnA 29 34 5.267 -69.30 -45.50 -60.50 -35.50 -91.20 7.60 50.30 38.60 YVLNHELN *** HHHHTT AAAa

1gweA 253 258 5.116 -66.90 -40.10 -60.70 -31.90 -82.40 -6.60 91.50 11.80 ESIAKGDH *** HHHHTT AAAa

1gweA 483 488 5.622 -61.00 -42.00 -67.00 -20.60 -86.60 -3.10 59.70 44.00 DEVKRHEG *** HHHHTT AAAa

1gwuA 109 114 5.141 -64.60 -44.10 -60.80 -29.30 -84.10 6.50 102.30 3.90 SVTLAGGP *** HHHHTT AAAa

1gwuA 236 241 4.624 -59.50 -39.10 -62.40 -24.70 -100.80 12.60 63.50 36.50 VNLEEQKG *** HHHHTT AAAa

1gx5A 107 112 5.210 -65.00 -52.10 -59.00 -33.00 -90.80 9.90 51.40 52.10 KDVRNLSS *** HHHHTT H AAAa

1gx5A 303 308 5.438 -71.40 -44.20 -60.40 -36.10 -86.50 -1.90 53.90 37.60 ACRAAKLQ *** HHHHTT E AAAa

1gx5A 343 348 5.111 -64.00 -43.20 -60.70 -37.70 -79.50 1.90 74.70 55.90 AXTRYSAP *** HHHHTT AAAa

1gx5A 433 438 4.889 -67.80 -44.20 -65.50 -28.90 -103.00 9.30 54.00 37.80 ILLAQEQL *** HHHHTT T AAAa

1gxmA 463 468 4.891 -63.20 -46.30 -60.90 -31.20 -101.40 11.80 56.20 35.40 DFAANKRA *** HHHHTT T AAAa

1h05A 40 45 4.795 -65.90 -39.80 -60.30 -31.50 -86.00 -6.50 82.00 17.10 EAAELGLK *** HHHHTT E AAAa

1h16A 122 127 4.806 -65.60 -45.00 -62.70 -36.60 -105.90 15.40 55.80 55.20 SCKAYNRE *** HHHHTT AAAa

1h16A 237 242 5.378 -64.50 -28.10 -63.00 -26.60 -94.40 7.40 84.90 15.70 MAAKYGYD *** HHHHTT AAAa

1h16A 290 295 4.956 -65.90 -45.80 -65.80 -27.90 -84.10 -7.70 75.20 17.60 RDLKAGKI *** HHHHTTSS AAAa

1h16A 444 449 4.452 -65.80 -41.10 -72.70 -34.80 -99.30 20.10 60.20 33.20 LYAINGGV *** HHHHTTTB AAAa

1h1dA 154 159 5.257 -68.50 -44.60 -57.90 -45.40 -77.20 8.10 60.50 34.90 LLEKCGLL *** HHHHTT AAAa

1h1nA 40 45 5.711 -63.60 -39.00 -66.50 -23.00 -82.40 -6.30 114.00 -3.20 TLISKGMN *** HHHHTT AAAa

1h1nA 81 86 5.628 -70.80 -28.50 -64.10 -25.30 -88.20 3.40 87.80 2.60 AITQKGAY *** HHHHTT E AAAa

1h1nA 153 158 5.725 -67.00 -32.70 -62.80 -23.80 -81.30 -1.40 81.60 11.70 GIRSAGAT *** HHHHTT AAAa

1h1nA 229 234 4.793 -66.50 -46.20 -60.90 -31.60 -99.10 5.80 62.20 33.60 WLRANGKK *** HHHHTT AAAa

1h4gA 166 171 5.077 -63.10 -35.80 -60.90 -36.50 -80.50 5.80 81.10 15.80 AWENLGMN *** HHHHTT AAAa

1h4rA 125 130 5.062 -70.40 -47.20 -67.00 -35.40 -82.40 3.10 68.50 19.80 KQILDEKI *** HHHHTTSS AAAa

1h4rA 308 313 5.163 -60.00 -38.00 -61.80 -25.80 -101.70 19.90 62.30 32.90 FMRRRKA# *** HHHHTT AAAa

1h4xA 90 95 5.449 -63.80 -33.30 -58.80 -35.70 -90.20 16.60 72.10 27.30 VFQFSGLG *** HHHHTT G AAAa

1h65A 32 37 4.924 -58.20 -26.30 -65.20 -36.40 -92.00 19.50 64.50 23.50 NLKQEDVN *** HHHHTT AAAa

1h65A 190 195 5.161 -72.20 -46.30 -59.30 -39.30 -84.70 0.60 54.90 41.40 VRSGASLK *** HHHHTT AAAa

1h70A 145 150 4.836 -63.80 -38.40 -57.60 -31.30 -97.90 13.90 78.50 9.10 ILEKHGLS *** HHHHTT E AAAa

1h72C 72 77 4.932 -66.20 -43.10 -62.80 -27.80 -107.40 15.50 49.60 40.20 MIDDFNIG *** HHHHTT AAAa

1h72C 110 115 4.864 -69.00 -49.10 -56.90 -42.60 -102.80 13.90 46.50 52.00 INELFKLN *** HHHHTT AAAa

1h72C 212 217 4.884 -65.40 -43.50 -65.10 -24.30 -101.20 9.20 49.10 41.30 YALYNKDK *** HHHHTT H AAAa

1h7eA 59 64 5.301 -60.80 -36.30 -55.70 -33.40 -97.90 7.40 86.60 12.00 AVQAFGGK *** HHHHTT E AAAa

1h7eA 205 210 5.357 -64.10 -33.50 -59.60 -37.50 -86.50 3.70 79.20 19.50 RLMNAGIN *** HHHHTT AAAa

1hbnA 531 536 5.208 -64.70 -37.40 -62.90 -34.80 -81.50 3.50 62.60 36.70 AKGALREF *** HHHHTT AAAa

1hbnB 201 206 5.241 -66.70 -46.70 -64.00 -31.30 -97.30 4.90 62.00 32.30 VVAATLKN *** HHHHTTT AAAa

1hbnB 223 228 5.102 -70.30 -46.20 -60.90 -38.20 -71.70 -12.70 84.20 -1.90 AMFEMGDA *** HHHHTTTT AAAa

1hbnB 276 281 5.049 -68.60 -47.90 -55.80 -37.80 -87.60 -4.10 81.30 16.00 RALEDGVI *** HHHHTTS AAAa

1hd2A 85 90 5.144 -66.30 -29.00 -70.60 -14.80 -124.10 19.60 54.90 43.50 WGRAHKAE *** HHHHTT T AAAa

1hdhA 64 69 5.211 -73.30 -20.20 -85.40 -41.40 -83.50 -5.50 121.30 -7.20 DHHIAGIG *** HHHHT S AAAa

1hdhA 301 306 5.198 -60.60 -51.60 -63.90 -30.40 -69.20 -10.50 69.30 26.00 YLRRQGEL *** HHHHTT G AAAa

1hdhA 442 447 5.178 -64.20 -51.90 -75.70 -20.20 -83.30 -0.40 86.80 -1.70 LGWLSGET *** HHHHTTSS AAAa

1hdoA 23 28 5.493 -64.60 -44.10 -61.60 -33.40 -76.50 -10.30 108.50 0.90 QAVQAGYE *** HHHHTT E AAAa

1hfeS 102 107 5.140 -70.60 -49.00 -65.80 -28.00 -77.20 -11.90 90.10 4.40 ELTTAGKL *** HHHHTT AAAa

1hfuA 357 362 5.897 -74.00 -40.60 -66.00 -21.90 -83.50 -15.60 140.50 -3.70 LQIMSGAQ *** HHHHTT AAAx

1hfuA 457 462 5.571 -56.70 -47.20 -65.80 -22.50 -100.10 17.80 109.70 -4.20 FHLMNGLA *** HHHHTT E AAAa

1hjsA 15 20 5.501 -62.50 -39.50 -69.10 -21.30 -87.70 4.10 97.90 2.80 VEERAGVS *** HHHHTT AAAa

1hjsA 36 41 5.314 -60.40 -41.40 -64.80 -25.30 -96.60 16.70 99.20 -11.50 ILAANGVN *** HHHHTT AAAa

1hjsA 69 74 5.127 -56.40 -43.30 -65.70 -21.40 -94.50 3.90 79.50 20.40 RAKAAGLG *** HHHHTT E AAAa

1hjsA 120 125 5.034 -56.60 -46.30 -64.20 -20.10 -95.70 5.70 77.40 19.60 KLQNAGIQ *** HHHHTT AAAa

1hm9A 118 123 5.405 -67.10 -52.70 -60.70 -35.60 -88.20 -5.40 54.90 47.50 FHINHKNV *** HHHHTT S AAAa

1hm9A 247 252 5.479 -65.10 -39.90 -62.10 -33.50 -81.60 8.80 95.70 19.00 KHMVNGVS *** HHHHTT E AAAa

1hn0A 373 378 5.961 -61.00 -39.70 -70.40 -34.80 -59.20 -6.00 98.50 7.80 HLLDQGFV *** HHHHTT S AAAa

1hn0A 408 413 4.933 -56.20 -57.50 -59.60 -38.10 -78.60 -1.00 64.20 30.90 ALKEANLQ *** HHHHTT H AAAa

1hnjA 125 130 5.304 -75.10 -40.90 -63.40 -32.70 -77.60 -8.10 85.90 2.60 QYVKSGAV *** HHHHTTS AAAa

1hnjA 228 233 4.956 -59.90 -37.90 -63.30 -21.00 -117.30 10.90 52.90 54.30 TLAANNLD *** HHHHTT AAAa

1hnjA 255 260 4.876 -64.40 -35.70 -64.30 -50.40 -71.10 3.00 61.20 31.60 TAKKLGMS *** HHHHTT AAAa

1hnjA 287 292 5.225 -73.70 -48.10 -65.40 -27.90 -96.50 9.30 81.80 5.80 EAVRDGRI *** HHHHTT S AAAa

1hp1A 498 503 4.997 -75.60 -40.80 -60.10 -29.20 -92.70 11.40 104.70 2.30 NFNATGGD *** HHHHTTGG AAAa

1hq0A 731 736 5.255 -67.70 -45.20 -62.10 -13.90 -103.10 12.00 80.50 12.90 QKLSRGNI *** HHHHTT G AAAa

1hq0A 905 910 5.123 -61.70 -35.40 -81.20 -11.00 -113.10 42.80 61.10 30.40 LELLTKEP *** HHHHTT S AADa

1hs6A 394 399 5.120 -66.60 -38.90 -72.00 -37.20 -94.40 9.70 126.10 -15.80 LEQLLGGP *** HHHHTT H AAAa

1hs6A 542 547 5.356 -64.20 -47.90 -65.10 -24.60 -88.70 6.80 69.60 31.00 LCIQSKWE *** HHHHTT T AAAa

1ht6A 30 35 5.960 -65.70 -38.50 -66.10 -25.40 -79.10 1.40 89.80 15.70 DIAAAGVT *** HHHHTT AAAa

1ht6A 78 83 5.033 -60.70 -34.70 -62.00 -25.90 -100.60 12.00 77.00 19.60 ALHGKGVQ *** HHHHTT E AAAa

1ht6A 343 348 5.120 -66.10 -45.50 -68.70 -19.50 -98.20 7.80 63.90 28.00 IRKRNGIT *** HHHHTT AAAa

1htrP 17 22 5.061 -61.40 -46.50 -63.30 -33.40 -91.50 5.40 66.10 31.60 TMKEKGLL *** HHHHTT H AAAa

1huw_ 181 186 6.670 -69.80 -26.80 -91.40 -14.90 -88.40 -150.40 -60.00 -13.40 VQbRSVEG *** HHHHTTSS AAPA

1hw1A 19 24 4.725 -76.20 -44.60 -63.00 -38.80 -91.00 10.90 65.10 24.30 ESIWNNRF *** HHHHTTSS AAAa

1hw1A 55 60 5.360 -65.30 -42.50 -66.10 -26.20 -86.60 -3.90 94.60 -2.70 RLARDGWL *** HHHHTTSE AAAa

1hw1A 153 158 7.328 -71.60 -31.40 -65.40 -15.40 -67.30 -21.50 95.10 -8.40 LAFASGNP *** HHHHTT H AAAa

1hx0A 86 91 4.986 -59.80 -34.00 -68.60 -16.90 -111.80 19.90 79.80 20.60 RaNNVGVR *** HHHHTT E AAAa

1hx0A 264 269 5.225 -63.00 -49.90 -66.70 -32.10 -76.20 -12.20 59.60 40.90 GTVVRKWS *** HHHHTT T AAAa

1hxiA 359 364 5.061 -67.60 -44.50 -59.50 -32.70 -86.50 2.30 68.00 26.80 SXLKLANL *** HHHHTT H AAAa

1hxiA 393 398 5.149 -63.80 -41.20 -66.60 -29.10 -92.60 20.80 65.30 24.00 TQAENEKD *** HHHHTT H AAAa

1hxn_ 427 432 5.504 -65.90 -38.50 -62.50 -36.80 -80.30 6.10 79.10 44.30 VSRLLGaT *** HHHHTT AAAa

1hyoA 87 92 5.161 -70.30 -41.20 -79.80 -15.10 -93.30 -7.50 65.40 24.60 QNLLSASQ *** HHHHTTSS AAAa

1hz4A 22 27 5.266 -69.70 -34.20 -63.80 -33.70 -84.90 8.40 73.60 34.50 VAINDGNP *** HHHHTT H AAAa

1hz4A 81 86 4.772 -63.00 -41.80 -57.60 -32.10 -110.90 17.90 55.70 33.20 MARQHDVW *** HHHHTT H AAAa

1hz4A 101 106 5.216 -63.20 -44.40 -60.50 -35.00 -77.00 -3.60 82.90 13.40 ILFAQGFL *** HHHHTT H AAAa

1hz4A 121 126 4.627 -67.60 -47.10 -61.90 -42.40 -98.50 10.70 53.90 54.90 LINEQHLE *** HHHHTT T AAAa

1hz4A 143 148 5.085 -63.90 -45.80 -54.40 -42.40 -78.80 2.00 66.30 28.60 LLWAWARL *** HHHHTT H AAAa

1hz4A 223 228 4.632 -61.90 -46.20 -65.00 -24.20 -108.00 9.00 58.50 33.60 YWQMTGDK *** HHHHTT H AAAa

1hz4A 261 266 4.856 -60.80 -45.10 -58.40 -36.90 -86.20 7.10 75.10 11.50 AQILLGEF *** HHHHTT H AAAa

1hz4A 281 286 4.809 -66.80 -37.90 -63.90 -31.10 -107.00 8.00 57.10 38.20 NARSLRLM *** HHHHTT H AAAa

1hz4A 344 349 7.399 -65.20 -42.40 -64.90 -28.70 -70.30 -17.50 60.90 27.40 QLIQLNTL *** HHHHTT S AAAa

1i0dA 90 95 5.472 -63.10 -41.10 -60.40 -33.30 -80.60 1.10 106.00 -2.80 RARAAGVR *** HHHHTT AAAa

1i0dA 216 221 5.485 -66.30 -29.20 -69.00 -15.50 -101.70 -0.70 85.30 11.40 IFESEGLS *** HHHHTT AAAa

1i0dA 243 248 5.693 -55.50 -40.50 -61.10 -30.40 -81.90 -4.90 109.40 -6.20 ALAARGYL *** HHHHTT E AAAa

1i0dA 287 292 5.460 -63.50 -48.80 -55.80 -34.40 -77.90 -1.20 96.60 10.30 ALIDQGYM *** HHHHTT G AAAa

1i0dA 336 341 5.417 -63.00 -40.60 -61.40 -28.90 -85.40 8.10 101.10 -4.10 FLREKGVP *** HHHHTT AAAa

1i0sA 152 157 5.564 -67.10 -35.70 -63.70 -29.30 -85.10 6.30 96.00 0.50 HLMKKGKT *** HHHHTT E AAAa

1i19A 107 112 4.523 -58.20 -49.00 -66.70 -15.90 -122.10 16.00 55.20 40.40 WAHEHDYK *** HHHHTT E AAAa

1i19A 175 180 4.784 -57.80 -35.40 -63.50 -18.60 -113.30 18.20 58.70 39.10 ELQKHDLG *** HHHHTTEE AAAa

1i19A 196 201 4.874 -64.80 -35.80 -54.00 -28.10 -123.10 22.40 50.70 49.60 GALAVNAH *** HHHHTT AAAa

1i19A 380 385 5.678 -62.30 -46.40 -63.40 -25.40 -83.00 4.00 104.40 26.70 GAINAGNP *** HHHHTT G AAAa

1i19A 404 409 4.802 -70.30 -52.40 -57.50 -36.80 -96.60 5.20 51.60 49.30 GLAATNAN *** HHHHTT S AAAa

1i1wA 194 199 5.290 -64.20 -37.80 -64.40 -25.70 -84.40 -3.10 95.90 10.60 KWRAAGVP *** HHHHTT AAAa

1i24A 21 26 5.577 -62.60 -43.70 -59.70 -38.20 -78.60 12.20 75.60 21.70 HLSKKNYE *** HHHHTT E AAAa

1i24A 342 347 5.666 -62.10 -39.70 -64.40 -24.80 -91.00 6.40 106.80 7.30 KLMELGLE *** HHHHTT AAAa

1i2kA 50 55 4.768 -60.00 -46.60 -70.30 -22.50 -94.00 6.20 63.20 30.20 ACQRLMIS *** HHHHTT AAAa

1i2kA 264 269 4.554 -86.80 -23.60 -75.40 -31.80 -56.40 -54.90 -90.10 118.70 PLCERPN# *** HHHHTT AAAB

1i3cA 48 53 5.267 -66.50 -47.40 -64.90 -32.20 -89.90 1.90 55.20 54.10 XAFLQQQG *** HHHHTT G AAAa

1i3cA 109 114 5.675 -67.40 -30.80 -67.60 -27.50 -88.70 9.00 73.60 35.50 ASYELHVN *** HHHHTT S AAAa

1i4uA 154 159 5.162 -60.60 -35.40 -63.70 -21.50 -112.70 24.50 60.80 33.10 AFKNINVD *** HHHHTT AAAa

1i7qA 511 516 4.823 -62.70 -51.10 -54.70 -26.20 -106.10 14.30 50.80 46.50 IATAHHAK *** HHHHTT S AAAa

1i7qB 21 26 5.587 -65.30 -37.50 -60.30 -24.80 -101.20 23.30 98.30 -17.20 QLRASGHQ *** HHHHTT E AAAa

1i7qB 92 97 5.016 -69.50 -44.90 -55.90 -28.30 -87.90 -5.50 105.10 4.70 IVEAYGGQ *** HHHHTT E AAAa

1i88A 41 46 4.814 -73.20 -49.40 -68.10 -18.90 -100.30 8.60 57.00 43.00 YFKITNSE *** HHHHTT T AAAa

1i88A 353 358 5.187 -68.20 -57.40 -46.90 -48.70 -83.10 11.20 63.40 31.40 KSTQNGLK *** HHHHTT S AAAa

1ib2A 835 840 5.234 -58.60 -45.90 -63.90 -28.00 -92.70 17.60 62.20 33.70 EDFRNNRY *** HHHHTT AAAa

1icxA 20 25 6.116 -59.90 -36.10 -78.00 -15.70 -120.10 -86.50 -50.20 -41.40 YKALTKDS *** HHHHTTTH AAAA

1ijbA 641 646 5.065 -59.00 -40.40 -69.20 -15.90 -105.80 13.90 58.30 34.90 GLKKKKVI *** HHHHTTEE AAAa

1inlA 290 295 4.771 -65.40 -40.20 -66.10 -33.00 -89.70 -2.90 74.60 38.20 VKKELGLM *** HHHHTT AAAa

1iomA 137 142 4.930 -64.00 -36.10 -64.20 -23.00 -96.50 2.10 76.10 22.60 KRLKEGKE *** HHHHTT AAAa

1iomA 246 251 5.175 -65.20 -37.30 -63.40 -34.50 -88.40 6.60 57.30 32.60 EKLAKKER *** HHHHTT AAAa

1iomA 319 324 5.390 -63.20 -45.80 -63.90 -25.90 -90.20 6.10 104.60 0.80 VYSDLGFS *** HHHHTT AAAa

1iow_ 30 35 5.302 -68.60 -37.60 -62.80 -28.00 -94.20 14.20 89.50 4.40 GLREGGID *** HHHHTT E AAAa

1iow_ 104 109 5.187 -63.90 -38.40 -64.00 -30.00 -82.80 -1.10 90.60 -0.50 LWQGAGLP *** HHHHTT AAAa

1iow_ 287 292 5.429 -56.10 -49.30 -61.40 -32.30 -81.20 11.60 80.90 2.90 AARQAGMS *** HHHHTT AAAa

1ispA 26 31 5.438 -61.70 -40.70 -59.30 -27.60 -99.70 9.50 108.30 -3.30 YLVSQGWS *** HHHHTT AAAa

1isuA 7 12 4.965 -64.30 -42.70 -55.90 -40.90 -103.50 14.10 51.30 46.10 MRKAFNYQ *** HHHHTT B AAAa

1ituA 55 60 5.430 -68.60 -38.60 -68.20 -33.50 -84.20 3.50 57.20 36.90 KLRAGFVG *** HHHHTTEE AAAa

1ituA 140 145 5.361 -59.50 -47.00 -62.80 -29.60 -84.00 0.40 115.00 -2.70 ALYQLGMR *** HHHHTTEE AAAa

1ituA 253 258 6.608 -65.70 -11.90 -104.80 -13.70 -94.70 -63.10 -94.70 -8.20 YNNYISbT *** HHHHTSS AAAA

1ituA 312 317 5.436 -63.30 -42.90 -57.60 -39.90 -78.70 10.20 75.00 28.30 ELLRRNWT *** HHHHTT AAAa

1itwA 100 105 5.312 -63.50 -40.20 -65.50 -25.90 -86.40 2.00 99.30 1.40 ELQQQGYK *** HHHHTT AAAa

1itwA 286 291 5.285 -70.20 -39.50 -58.70 -42.90 -85.40 6.40 68.60 30.70 VLKQIGFD *** HHHHTT AAAa

1itwA 356 361 4.838 -60.30 -42.50 -65.40 -40.10 -86.70 57.10 59.50 17.20 AMIRDSGK *** HHHHTTTE AADa

1itwA 531 536 5.007 -60.40 -39.90 -71.20 -21.30 -95.00 13.50 79.90 15.80 ARIREGKD *** HHHHTT AAAa

1itwA 618 623 4.473 -75.60 -38.00 -69.70 -42.20 -99.20 6.40 51.60 51.10 LGNAYKNP *** HHHHTT H AAAa

1itwA 640 645 4.856 -73.60 -53.40 -64.10 -31.70 -99.00 10.90 49.20 56.80 KILDNNKS *** HHHHTT S AAAa

1itxA 301 306 5.421 -64.40 -40.60 -64.30 -28.50 -80.80 -10.30 77.30 23.30 AASAAGVP *** HHHHTT T AAAa

1iu8A 121 126 5.128 -61.20 -39.20 -61.30 -22.90 -100.30 15.40 74.80 19.30 EMKKNGIP *** HHHHTT AAAa

1iuaA 14 19 4.802 -67.30 -49.70 -62.00 -38.40 -93.20 0.00 53.20 46.80 TAIALKYN *** HHHHTT B AAAa

1iulA 113 118 5.316 -63.10 -38.60 -67.80 -28.40 -80.50 -9.70 88.60 14.70 ALKEAGIP *** HHHHTT AAAa

1iv2A 49 54 4.948 -57.30 -37.90 -61.80 -24.20 -115.10 10.50 71.30 22.00 LLSAYGLG *** HHHHTT AAAa

1iv2A 84 89 5.349 -57.40 -41.90 -65.10 -22.90 -96.40 13.40 98.80 -3.80 LVEARGAK *** HHHHTT AAAa

1iv8A 75 80 4.831 -62.80 -35.20 -62.90 -41.50 -88.50 6.30 87.60 18.40 TAHTIGLG *** HHHHTT E AAAa

1iv8A 137 142 5.536 -73.70 -40.00 -68.50 -29.60 -80.70 -12.30 71.70 35.50 TVISKGLL *** HHHHTT E AAAa

1ixh_ 44 49 5.382 -68.10 -36.70 -62.80 -39.50 -72.90 -12.50 67.40 31.00 KQIIANTV *** HHHHTT S AAAa

1ixh_ 102 107 5.251 -64.50 -48.80 -69.10 -19.40 -80.60 -13.10 86.30 9.20 GDIYLGKI *** HHHHTT AAAa

1ixh_ 288 293 5.116 -73.70 -27.80 -62.40 -30.60 -98.60 13.40 64.70 32.30 QANDLDYA *** HHHHTT E AAAa

1ixkA 11 16 5.622 -64.40 -38.10 -67.70 -33.60 -69.30 -10.50 110.50 4.40 KLLRLGYS *** HHHHTT AAAa

1ixkA 63 68 5.580 -59.60 -40.50 -60.40 -29.00 -87.20 -2.50 108.00 -6.40 RLNKKGFQ *** HHHHTT E AAAa

1ixkA 94 99 4.964 -72.10 -42.70 -68.00 -23.80 -102.90 13.20 73.30 28.20 PEFLTGLI *** HHHHTTSE AAAa

1ixkA 137 142 5.682 -72.80 -30.90 -68.00 -29.20 -89.00 -0.30 51.40 45.20 LAQLXRND *** HHHHTTT AAAa

1izcA 89 94 4.941 -76.50 -42.30 -74.80 -8.20 -108.10 3.30 49.80 42.50 AQHHSEGR *** HHHHTTT AAAa

1izcA 213 218 4.953 -64.20 -45.30 -65.90 -30.80 -86.70 -2.60 70.60 28.10 YMIDAGLD *** HHHHTT AAAa

1izcA 243 248 4.794 -58.60 -45.60 -64.90 -28.30 -95.60 2.40 69.50 37.90 AAQRNGVP *** HHHHTT AAAa

1izcA 263 268 5.404 -63.80 -39.60 -64.20 -29.30 -81.60 -5.90 107.00 4.60 SLIEQGYR *** HHHHTTEE AAAa

1izmA 12 17 5.382 -65.80 -44.10 -66.30 -22.20 -91.70 5.60 63.80 26.00 QLKSAGIG *** HHHHTT S AAAa

1izmA 31 36 5.634 -66.20 -38.40 -72.40 -17.20 -90.50 3.50 87.20 13.90 GLLCGGLK *** HHHHTT AAAa

1izmA 72 77 6.402 -65.10 -34.50 -73.50 -22.20 -81.90 6.70 83.10 52.70 SQTLSDVE *** HHHHTTTT AAAa

1j1nA 83 88 5.479 -66.60 -48.60 -63.90 -29.50 -82.30 -3.90 102.20 16.10 QYGQEGAF *** HHHHTTSB AAAa

1j1nA 146 151 4.830 -66.20 -46.50 -59.10 -38.80 -91.90 4.00 56.00 42.10 WLKKLNLK *** HHHHTT AAAa

1j1nA 237 242 5.309 -68.60 -40.80 -61.40 -37.20 -82.00 0.70 76.00 15.20 QWYKEGLI *** HHHHTT B AAAa

1j1nA 470 475 5.356 -69.10 -49.20 -72.70 -11.10 -96.00 -2.10 88.00 10.70 QLKLRGLY *** HHHHTTHH AAAa

1j1tA 10 15 7.441 -56.50 -30.70 -89.00 -59.60 -57.80 -37.40 -88.40 -18.50 PSSITSGS *** HHHHT S AAAA

1j1tA 211 216 4.950 -64.30 -49.40 -67.70 -28.20 -83.10 -6.20 74.90 20.70 aFEDLGIT *** HHHHTT AAAa

1j1yA 6 11 5.076 -60.40 -46.60 -59.30 -29.70 -92.30 -1.80 76.60 21.80 FMEALGLK *** HHHHTT E AAAa

1j2rA 55 60 5.187 -60.50 -39.80 -68.80 -24.70 -89.00 -3.50 69.60 29.60 KFRASGQP *** HHHHTT AAAa

1j2rA 127 132 5.046 -66.90 -44.20 -71.50 -20.90 -91.50 2.80 72.40 30.10 QLRRRGID *** HHHHTT AAAa

1j2rA 153 158 5.605 -63.60 -46.10 -64.00 -30.00 -76.50 -8.00 100.00 2.60 NAWELGFN *** HHHHTT E AAAa

1j30A 64 69 5.016 -64.90 -48.40 -65.40 -16.10 -99.50 0.10 76.60 16.40 FIRQGGLT *** HHHHTT B AAAa

1j30A 107 112 5.100 -63.80 -43.90 -62.30 -28.60 -91.60 0.60 83.90 31.60 VAREEGFP *** HHHHTT H AAAa

1j31A 30 35 5.587 -59.80 -45.60 -60.10 -31.20 -81.30 -1.90 96.60 4.30 EASKEGAK *** HHHHTT S AAAa

1j31A 158 163 5.474 -63.40 -41.10 -66.90 -25.20 -85.30 5.70 104.90 -0.10 TLALKGAE *** HHHHTT S AAAa

1j53A 149 154 4.510 -67.70 -42.10 -60.60 -39.00 -105.40 14.30 53.50 46.90 LCARYEID *** HHHHTT AAAa

1j5wA 14 19 5.580 -61.90 -42.70 -64.30 -18.80 -96.70 6.40 94.70 16.10 FWASKGCL *** HHHHTT E AAAa

1j5wA 96 101 5.308 -60.70 -29.30 -69.70 -28.10 -89.00 5.10 88.10 26.20 SLEYLGIN *** HHHHTT AAAa

1j5wA 223 228 5.553 -64.90 -27.80 -70.60 -23.30 -89.80 17.00 64.80 19.00 RLVEKNLY *** HHHHTT H AAAa

1j5wA 246 251 5.504 -57.70 -46.30 -66.50 -22.10 -92.00 6.20 59.70 31.00 LLDARGAI *** HHHHTT AAAa

1j6pA 254 259 4.894 -72.80 -47.30 -58.80 -34.90 -97.80 13.60 69.90 26.20 SNLKLGNG *** HHHHTT AAAa

1j6pA 267 272 5.386 -65.00 -37.60 -63.10 -25.90 -91.30 8.20 84.40 8.60 RXIEHGXK *** HHHHTT E AAAa

1j79A 92 97 4.759 -66.80 -54.60 -53.40 -37.30 -85.60 11.40 66.00 17.30 RGFNEGVF *** HHHHTTSE AAAa

1j79A 282 287 4.863 -65.30 -55.80 -55.90 -30.60 -86.80 2.50 59.70 36.60 VFEEMNAL *** HHHHTT G AAAa

1j9lA 116 121 5.085 -59.10 -40.50 -62.30 -18.40 -107.60 10.60 57.70 39.80 EGAMMNIP *** HHHHTT AAAa

1j9lA 218 223 5.167 -58.40 -42.50 -57.80 -36.50 -86.40 10.70 82.50 21.00 KAVREGYV *** HHHHTTEE AAAa

1jakA 234 239 4.935 -61.10 -37.90 -69.30 -16.40 -106.60 16.10 53.70 45.80 YAASRHLE *** HHHHTT E AAAa

1jakA 334 339 5.509 -66.20 -33.50 -56.40 -35.30 -99.80 24.20 67.30 28.30 IVAKYGKT *** HHHHTT E AAAa

1jakA 377 382 5.417 -62.10 -43.30 -62.60 -31.80 -81.60 5.10 102.80 -2.30 EAARNGTG *** HHHHTT E AAAa

1jb9A 264 269 5.531 -67.70 -35.70 -66.10 -25.40 -87.20 5.00 105.50 8.20 KLLDGGAH *** HHHHTT E AAAa

1jb9A 305 310 5.201 -68.30 -43.10 -60.30 -35.00 -88.10 3.10 64.10 39.00 QLKKNKQW *** HHHHTT E AAAa

1jbeA 25 30 5.093 -60.80 -39.40 -58.00 -31.20 -90.60 -4.10 95.00 13.20 LLKELGFN *** HHHHTT AAAa

1jbeA 98 103 5.423 -61.60 -47.30 -64.80 -28.50 -80.90 -5.50 112.00 12.50 AAAQAGAS *** HHHHTT S AAAa

1jboA 12 17 5.025 -62.20 -41.10 -62.30 -31.70 -93.60 8.10 61.40 31.10 AADTQGRF *** HHHHTT AAAa

1jdrA 116 121 5.085 -64.80 -43.40 -65.10 -29.20 -87.00 23.90 69.80 21.30 AVQEMQGP *** HHHHTT AAAa

1jdrA 289 294 4.941 -57.70 -44.30 -67.00 -28.70 -87.60 -6.60 71.10 23.50 TLEEQGL# *** HHHHT AAAa

1jdw_ 134 139 5.332 -63.50 -38.00 -64.90 -35.30 -81.20 13.10 78.20 21.40 ILKTEGVT *** HHHHTT E AAAa

1jdw_ 203 208 5.446 -63.20 -46.20 -60.00 -30.40 -88.60 10.20 109.00 -9.60 DYFHRGAK *** HHHHTT E AAAa

1jdw_ 330 335 5.502 -61.50 -45.20 -60.10 -30.70 -80.20 -3.00 77.60 29.50 LFKKAGWT *** HHHHTT E AAAa

1jdw_ 383 388 5.143 -61.30 -38.90 -65.40 -24.80 -87.80 4.10 76.50 24.60 MFEKLGIT *** HHHHTT E AAAa

1jfxA 22 27 6.004 -65.60 -45.70 -68.80 -19.90 -70.80 -12.40 106.60 4.60 SVKSAGMS *** HHHHTT AAAa

1jfxA 52 57 5.293 -61.00 -48.90 -62.10 -28.60 -80.50 -6.70 90.80 14.70 NAYNAGII *** HHHHTT E AAAa

1jfxA 79 84 5.192 -61.70 -27.20 -91.10 -8.30 -133.20 48.10 123.60 -23.80 YFASNGGG *** HHHHTT AADa

1jg1A 28 33 5.110 -70.10 -43.50 -62.00 -37.90 -82.60 0.10 84.50 8.80 MLKAEGII *** HHHHTTS AAAa

1jg1A 134 139 5.146 -63.30 -45.40 -59.90 -30.20 -86.80 -0.20 87.60 4.60 NLERAGVK *** HHHHTT AAAa

1jh6A 105 110 5.016 -63.60 -56.00 -56.30 -40.90 -91.80 17.90 46.20 49.50 CKNHFNCS *** HHHHTT AAAa

1jhgA 29 34 4.768 -57.10 -40.60 -65.40 -24.90 -105.70 9.60 58.00 35.20 NAYQNDLH *** HHHHTT H AAAa

1ji1A 477 482 5.339 -81.70 -42.60 -62.50 -29.90 -83.90 2.00 66.60 23.50 FATRSGGD *** HHHHTTT AAAa

1ji7A 42 47 4.722 -77.30 -41.40 -63.30 -36.00 -107.70 7.90 53.30 46.80 AENEFSLR *** HHHHTT S AAAa

1jidA 53 58 5.811 -61.20 -37.70 -62.80 -31.60 -85.80 6.30 97.10 1.90 VCSAVGLN *** HHHHTT AAAa

1jixA 346 351 5.037 -63.90 -38.50 -61.60 -35.10 -98.00 25.90 58.80 40.90 FKKAIDL# *** HHHHTT AAAa

1jndA 119 124 4.790 -69.20 -48.90 -59.00 -37.80 -100.30 11.20 85.10 17.40 LVKTYGFD *** HHHHTT S AAAa

1jndA 262 267 5.612 -64.10 -45.00 -70.30 -24.70 -82.30 -2.90 105.90 9.40 YWLSQGFP *** HHHHTT AAAa

1jndA 383 388 5.187 -62.20 -43.10 -59.40 -39.00 -88.50 8.20 53.10 39.60 YARVKNLG *** HHHHTT S AAAa

1jnrA 97 102 5.108 -69.00 -35.10 -66.30 -31.00 -82.80 10.40 57.70 41.80 VTLDMMGL *** HHHHTTT AAAa

1jnrA 123 128 5.648 -68.00 -37.60 -60.50 -39.20 -77.10 -1.70 119.80 -8.50 LFEKWGLP *** HHHHTT AAAa

1jnrA 324 329 5.292 -65.30 -43.30 -68.30 -29.10 -79.30 -8.00 77.40 36.60 LEIMDGNQ *** HHHHTT AAAa

1jnrA 373 378 4.641 -63.00 -45.50 -68.40 -30.70 -100.90 8.70 53.70 46.10 LWACQNID *** HHHHTT AAAa

1jnrA 526 531 4.007 -82.90 -26.20 -119.40 -16.50 -110.60 38.50 62.60 50.60 MDEYAAGI *** HHHHTT G AAAa

1jq5A 82 87 5.243 -59.20 -51.90 -53.60 -44.70 -74.80 -4.70 61.70 34.20 IARKAEAA *** HHHHTT S AAAa

1jq5A 222 227 5.509 -66.30 -36.10 -59.60 -37.40 -91.90 2.10 54.80 47.50 ESVKAKVV *** HHHHTT AAAa

1jq5A 303 308 4.913 -63.80 -40.20 -61.50 -29.80 -96.80 9.40 61.70 35.60 LYLCLDLP *** HHHHTT AAAa

1jr7A 72 77 4.986 -61.00 -34.70 -69.00 -21.10 -102.90 28.30 52.10 36.60 LDDLCANQ *** HHHHTTT AAAa

1jraA 90 95 4.701 -57.70 -43.00 -59.10 -43.00 -88.10 8.30 61.30 40.40 VNEYLKKD *** HHHHTT AAAa

1jraA 104 109 5.243 -63.40 -43.30 -54.60 -27.50 -119.80 40.40 42.30 60.30 ILEDYDcG *** HHHHTT S AADa

1jrlA 95 100 5.532 -63.70 -41.50 -63.70 -19.60 -93.60 13.40 59.10 37.00 DVKAANAE *** HHHHTT E AAAa

1jrlA 129 134 4.867 -74.60 -43.30 -60.80 -39.00 -103.00 3.30 51.90 51.50 LAKEFDVP *** HHHHTT AAAa

1ju2A 162 167 5.442 -68.00 -35.20 -70.60 -31.40 -85.10 10.80 80.80 15.80 AFLEAGVH *** HHHHTT AAAa

1ju2A 266 271 5.084 -61.40 -46.90 -61.90 -24.50 -91.10 2.80 89.40 15.20 LLLLSGVG *** HHHHTTEE AAAa

1ju2A 277 282 5.100 -64.40 -33.80 -63.20 -24.80 -103.40 5.00 65.40 29.20 YLSSLNIP *** HHHHTT AAAa

1ju3A 62 67 5.332 -71.00 -36.60 -71.20 -23.40 -94.50 4.30 108.00 7.60 EFVRDGYA *** HHHHTT E AAAa

1juhA 41 46 3.913 -57.70 -42.70 -71.30 -22.90 -110.60 8.40 66.00 32.10 TGPSSGYA *** HHHHTTS AAAa

1jv1A 10 15 4.889 -61.10 -41.10 -65.90 -21.20 -97.60 6.20 72.50 25.90 TLSKAGQE *** HHHHTT G AAAa

1jv1A 97 102 5.497 -60.50 -44.50 -63.50 -24.40 -92.60 11.30 58.20 35.30 FQISQNKV *** HHHHTT E AAAa

1jv1A 178 183 4.950 -53.60 -49.30 -57.10 -33.00 -96.90 16.00 51.70 40.50 FFTKHKYF *** HHHHTGGG AAAa

1jv1A 230 235 4.851 -69.40 -41.00 -62.80 -23.20 -121.90 22.90 46.60 39.00 ALAAQNIV *** HHHHTTHH AAAa

1jv1A 239 244 5.471 -62.90 -43.60 -63.40 -31.40 -82.30 -6.10 76.80 28.50 DMEQRGIW *** HHHHTT AAAa

1jv1A 268 273 5.288 -60.30 -49.30 -61.60 -32.10 -83.20 -4.50 70.60 29.50 FCIQKGAD *** HHHHTT S AAAa

1jv1A 433 438 5.315 -62.70 -41.20 -70.10 -21.90 -90.60 6.40 99.60 0.50 WVLNAGGH *** HHHHTT E AAAa

1jyhA 37 42 5.069 -66.60 -46.20 -59.30 -31.30 -100.10 0.80 56.30 40.80 WVDSKNIV *** HHHHTT AAAa

1jztA 81 86 5.354 -66.20 -45.10 -64.80 -34.60 -73.10 -6.60 106.10 2.70 HLKLFGYN *** HHHHTT AAAa

1jztA 109 114 5.149 -62.10 -42.20 -53.30 -38.00 -95.00 10.00 59.30 38.90 QLNFFKVP *** HHHHTT AAAa

1jztA 227 232 5.186 -53.10 -38.60 -59.10 -31.50 -99.40 16.80 85.70 6.90 FANKFGFE *** HHHHTT AAAa

1k07A 99 104 5.501 -70.70 -33.60 -61.60 -33.80 -75.80 -6.50 94.20 7.90 SIKKLGFK *** HHHHTT AAAa

1k07A 145 150 4.967 -68.20 -44.60 -63.80 -32.70 -93.30 2.40 59.70 29.50 SVILSGGK *** HHHHTTTT AAAa

1k07A 275 280 5.477 -64.70 -44.40 -63.70 -31.30 -73.90 -7.20 95.70 8.30 VLLSKGQN *** HHHHTT S AAAa

1k1eA 47 52 4.974 -58.60 -40.60 -62.60 -30.60 -94.10 20.70 67.00 30.40 MLMDADIQ *** HHHHTT E AAAa

1k1eA 156 161 5.064 -65.90 -40.10 -59.50 -34.40 -84.50 5.40 71.60 23.20 ILQAQGKS *** HHHHTT T AAAa

1k2xA 42 47 5.374 -62.90 -46.90 -56.80 -37.20 -78.60 -2.30 91.80 13.90 KMLEAGES *** HHHHTT AAAa

1k2xB 236 241 5.189 -69.70 -52.20 -58.10 -36.10 -94.10 1.10 38.10 57.70 VFIRALAA *** HHHHTTHH AAAa

1k2xB 268 273 4.847 -56.00 -40.60 -58.40 -27.30 -97.90 5.40 81.00 25.60 KLPALGGS *** HHHHTT AAAa

1k30A 24 29 5.375 -67.10 -46.60 -58.00 -33.10 -79.60 -4.80 77.70 17.30 KETEAGKL *** HHHHTTSS AAAa

1k30A 126 131 5.487 -61.90 -41.20 -63.90 -28.00 -78.50 0.00 88.40 3.10 EKLQQGHN *** HHHHTT E AAAa

1k3yA 44 49 5.531 -61.90 -49.90 -68.00 -25.70 -83.30 -4.80 88.60 12.90 KLRNDGYL *** HHHHTT AAAa

1k3yA 76 81 4.632 -61.90 -46.10 -58.30 -40.10 -103.00 20.40 60.00 43.40 IASKYNLY *** HHHHTT S AAAa

1k4iA 21 26 5.644 -65.40 -40.80 -61.80 -30.20 -79.80 6.40 79.10 18.60 QAFKNGEF *** HHHHTT AAAa

1k4iA 74 79 4.877 -75.90 -46.70 -58.90 -37.30 -96.90 1.20 58.30 59.30 RTTALDLP *** HHHHTT AAAa

1k4iA 161 166 4.742 -61.10 -48.70 -63.20 -31.10 -93.30 1.00 69.80 29.30 LCRLAGKR *** HHHHTT AAAa

1k4iA 203 208 4.802 -63.70 -40.10 -60.40 -32.20 -103.90 11.40 68.60 32.00 FARRWGLK *** HHHHTT E AAAa

1k55A 77 82 4.946 -68.20 -44.00 -61.10 -41.10 -82.40 -7.30 84.40 -0.90 IGLETGVI *** HHHHTTSS AAAa

1k55A 111 116 4.993 -66.60 -48.00 -68.20 -33.10 -93.60 2.60 56.50 51.40 GAIQVSAV *** HHHHTT H AAAa

1k55A 136 141 5.186 -69.10 -45.50 -58.70 -41.10 -80.60 -3.30 50.70 51.90 YLKKFSYG *** HHHHTT T AAAa

1k55A 172 177 4.797 -61.40 -47.00 -52.40 -41.60 -87.50 1.20 61.60 29.10 ESLYLNKL *** HHHHTT S AAAa

1k55A 258 263 5.144 -64.30 -35.90 -66.20 -20.40 -95.40 5.70 84.20 9.90 IMESEGII *** HHHHTT AAAa

1k5nA 82 87 4.837 -61.60 -51.10 -57.40 -34.00 -101.30 6.30 55.60 45.10 LLRYYNQS *** HHHHTT AAAa

1k5nA 147 152 5.154 -65.80 -43.20 -62.50 -37.50 -90.10 3.30 53.60 45.20 KWEAARVA *** HHHHTTHH AAAa

1k5nA 159 164 5.743 -68.10 -37.90 -81.00 -20.10 -111.10 -94.20 -53.70 -43.10 AYLEGEaV *** HHHHTHHH AAAA

1k75A 173 178 5.101 -64.20 -43.60 -54.20 -40.70 -94.80 15.20 74.30 16.70 AAQLCGVQ *** HHHHTT AAAa

1k75A 411 416 5.331 -65.20 -37.90 -67.70 -31.20 -87.10 -1.30 58.40 33.90 LAAAERLT *** HHHHTT H AAAa

1k77A 49 54 4.874 -68.20 -44.30 -57.50 -37.70 -103.90 11.10 49.70 50.70 QLEQNHLT *** HHHHTT E AAAa

1k77A 94 99 4.700 -68.20 -48.90 -66.50 -32.50 -98.30 7.30 54.60 47.50 YALALNCE *** HHHHTT S AAAa

1k77A 228 233 5.290 -66.80 -48.30 -52.40 -47.00 -79.80 -1.80 70.30 33.30 LFDEVGYQ *** HHHHTT AAAa

1k7cA 48 53 5.339 -66.00 -51.30 -59.60 -38.90 -76.50 1.20 79.50 14.50 SYTREGRF *** HHHHTTHH AAAa

1k7cA 121 126 5.368 -63.90 -38.40 -63.80 -27.00 -83.00 -4.60 96.80 7.60 LFTAKGAK *** HHHHTT E AAAa

1k7jA 23 28 5.334 -63.80 -44.20 -62.20 -28.30 -84.60 5.50 93.00 17.30 EIVRKGGV *** HHHHTT AAAa

1k8wA 33 38 5.278 -72.60 -41.60 -57.20 -38.20 -91.70 1.50 48.40 43.80 VKRIYNAN *** HHHHTT S AAAa

1k8wA 139 144 4.975 -53.40 -48.70 -65.00 -17.30 -96.40 4.40 71.00 35.00 EYARQGIE *** HHHHTT AAAa

1k8wA 226 231 5.022 -65.80 -42.40 -60.90 -39.30 -92.40 4.10 53.80 54.20 QAEQQDIP *** HHHHTTS AAAa

1k92A 30 35 5.349 -69.50 -46.20 -58.30 -34.60 -87.80 5.10 106.90 -3.80 WMRQKGAV *** HHHHTT E AAAa

1k92A 117 122 4.877 -59.50 -39.90 -59.80 -30.30 -96.50 10.90 72.10 25.10 AMKEDGVN *** HHHHTT AAAa

1k92A 175 180 5.316 -64.90 -41.50 -65.40 -25.00 -90.10 5.70 95.00 15.10 FMIACGFD *** HHHHTT AAAa

1k92A 329 334 5.010 -59.30 -47.80 -59.00 -35.00 -76.50 -7.60 86.80 20.10 RLLYQGRW *** HHHHTT T AAAa

1k92A 421 426 5.163 -70.60 -39.60 -64.30 -35.80 -92.10 11.80 83.00 1.70 GYAKTGLL *** HHHHTTSB AAAa

1ka1A 149 154 4.968 -50.90 -52.30 -67.10 -28.90 -76.20 -2.30 70.90 22.50 KGFLRGEQ *** HHHHTTS AAAa

1ka1A 251 256 4.831 -63.10 -44.60 -60.30 -34.90 -94.60 7.00 57.50 33.00 IKNKLNIS *** HHHHTT AAAa

1ka1A 302 307 5.297 -57.30 -50.60 -69.50 -20.50 -83.70 -2.10 96.50 13.40 IVHEAGGI *** HHHHTT E AAAa

1kgdA 794 799 5.201 -62.10 -45.10 -63.00 -25.80 -93.60 16.70 54.30 42.40 QDISNNEY *** HHHHTT E AAAa

1kgdA 821 826 5.593 -62.10 -39.10 -63.90 -21.60 -91.60 3.80 76.30 20.30 KIHEQGLI *** HHHHTT E AAAa

1khcA 342 347 5.167 -63.50 -52.50 -63.20 -27.00 -78.20 -2.50 99.30 18.80 EWAHGGFK *** HHHHTTST AAAa

1kjqA 94 99 5.620 -63.00 -43.80 -57.00 -35.20 -80.00 1.00 111.30 -8.10 QLEEEGLN *** HHHHTT E AAAa

1kjqA 305 310 5.349 -65.30 -49.50 -67.00 -26.60 -75.20 -2.60 83.70 18.50 VRAFLGLP *** HHHHTT AAAa

1kkoA 22 27 5.914 -68.70 -44.00 -60.90 -36.10 -63.40 -14.90 111.10 16.40 QAIKNGAG *** HHHHTT AAAa

1kkoA 155 160 4.437 -66.90 -48.70 -75.60 -22.90 -103.30 2.70 54.40 59.00 VaDEWQLP *** HHHHTT AAAa

1kkoA 184 189 5.301 -67.00 -44.70 -57.70 -33.70 -101.60 17.30 55.20 39.70 KXILKGVD *** HHHHTT S AAAa

1kkoA 244 249 4.469 -64.80 -35.90 -87.20 -11.80 -116.70 31.20 57.90 28.20 IGLIFDXD *** HHHHTTT AAAa

1kkoA 319 324 5.766 -65.40 -49.60 -61.90 -29.60 -79.20 -8.00 59.30 40.00 DFTDAGSC *** HHHHTT AAAa

1kl9A 115 120 5.144 -68.30 -44.40 -60.10 -43.50 -82.70 2.50 54.40 52.10 VAEVLEYT *** HHHHTT AAAa

1kl9A 130 135 5.759 -53.20 -37.80 -92.70 -3.80 -127.90 -88.40 -49.60 -46.00 LFQRTAWV *** HHHHTHHH AAAA

1kllA 22 27 5.573 -56.20 -45.90 -65.20 -28.70 -75.30 -9.00 107.60 -3.90 FYRKXGVE *** HHHHTT AAAa

1kllA 92 97 5.576 -63.30 -38.30 -58.40 -41.00 -72.30 -8.30 103.70 5.70 ELVDAGYE *** HHHHTT E AAAa

1km4A 87 92 5.978 -70.90 -34.30 -59.80 -34.50 -73.80 -6.60 103.90 0.20 ATFKAGAD *** HHHHTT S AAAa

1kngA 148 153 5.341 -65.10 -37.40 -63.70 -33.00 -75.70 -8.00 81.30 24.30 ASIEWGVY *** HHHHTT AAAa

1koe_ 168 173 5.290 -62.10 -39.10 -61.50 -29.10 -105.10 14.80 74.20 18.70 QARAVGLS *** HHHHTT AAAa

1kolA 51 56 5.477 -65.80 -47.60 -63.70 -29.60 -75.70 -6.20 79.70 28.60 QHMVRGRT *** HHHHTT S AAAa

1kolA 102 107 4.704 -61.70 -43.60 -62.90 -33.00 -88.30 11.40 71.80 19.20 RSCKEMHT *** HHHHTT G AAAa

1kolA 178 183 5.427 -63.70 -46.20 -70.80 -17.60 -86.10 4.30 77.90 23.20 GAVTAGVG *** HHHHTT AAAa

1kolA 205 210 5.406 -63.90 -43.50 -62.30 -34.70 -70.80 -11.40 105.60 8.00 SARLLGAA *** HHHHTT S AAAa

1kolA 226 231 5.704 -63.10 -40.50 -66.90 -25.50 -82.80 2.70 95.20 8.50 HAKAQGFE *** HHHHTT E AAAa

1kolA 313 318 4.905 -70.40 -23.10 -67.00 -18.70 -113.60 24.50 76.80 11.50 AAAKIGSL *** HHHHTT AAAa

1kolA 326 331 5.235 -63.50 -46.30 -62.40 -22.20 -100.10 6.10 55.90 45.70 LGWAKSHS *** HHHHTT E AAAa

1kolA 350 355 5.054 -66.80 -47.70 -65.40 -28.60 -95.30 15.70 58.60 39.20 QAIMWDRI *** HHHHTTS AAAa

1kp6A 65 70 5.072 -62.60 -38.90 -60.10 -26.60 -94.20 5.70 66.40 41.50 HcSSLNNN *** HHHHTT E AAAa

1kpeA 21 26 4.993 -70.50 -42.90 -64.70 -35.70 -82.70 2.90 59.50 23.80 GKIIRKEI *** HHHHTTSS AAAa

1kpgA 272 277 5.590 -79.60 -38.20 -58.70 -38.40 -90.40 -4.80 77.00 24.80 EXFRIGYI *** HHHHTTSE AAAa

1kqfA 181 186 5.372 -70.90 -33.70 -66.50 -22.90 -90.10 -5.10 85.80 12.80 FARSLGML *** HHHHTTB AAAa

1kqfA 291 296 4.763 -64.10 -45.80 -63.00 -34.50 -97.50 6.10 55.10 40.60 YLIENNKI *** HHHHTT S AAAa

1kqfA 454 459 5.652 -74.10 -44.00 -57.00 -42.50 -79.60 -3.00 75.90 34.00 GLTDLGLL *** HHHHTT S AAAa

1kqfA 543 548 5.330 -61.80 -39.40 -67.70 -22.10 -94.70 4.60 81.20 19.20 NMMDEGKV *** HHHHTT AAAa

1kqfC 94 99 5.562 -70.10 -39.50 -67.40 -18.70 -88.70 5.30 87.00 14.50 VEVLKGNE *** HHHHTT H AAAa

1kqpA 65 70 5.239 -65.50 -39.20 -59.80 -29.60 -82.40 3.30 91.10 8.60 SIREEGGD *** HHHHTT AAAa

1kqpA 193 198 5.356 -61.40 -44.20 -60.20 -34.80 -80.40 -6.30 84.70 21.60 LLKELGAP *** HHHHTT AAAa

1kqpA 233 238 5.285 -59.10 -54.50 -65.20 -28.30 -86.00 3.90 81.80 15.60 DDYLEGKE *** HHHHTT AAAa

1ks8A 83 88 4.904 -62.40 -43.00 -63.20 -28.40 -91.00 2.10 61.70 33.90 GYSSAGAL *** HHHHTT H AAAa

1ks8A 239 244 5.478 -73.10 -44.40 -56.80 -47.10 -81.00 -10.00 60.60 43.60 LYDEFGLQ *** HHHHTTGG AAAa

1ks8A 317 322 5.780 -59.90 -34.80 -65.80 -23.20 -79.70 -8.10 94.60 11.40 EAAELGLS *** HHHHTTSS AAAa

1ks8A 428 433 5.394 -67.20 -42.00 -61.80 -31.20 -83.80 -2.90 78.80 19.00 ALVALGY# *** HHHHTT AAAa

1kt6A 156 161 5.224 -62.70 -41.70 -64.80 -28.30 -84.80 1.30 58.70 35.80 RQEELaLA *** HHHHTT T AAAa

1kugA 21 26 5.028 -66.30 -39.00 -58.70 -38.10 -96.00 39.60 55.10 31.00 MYTKYSSN *** HHHHTTT AAAa

1kugA 147 152 5.418 -56.20 -43.60 -65.90 -21.50 -90.10 16.70 88.30 11.70 LGHNLGME *** HHHHTT AAAa

1kv7A 151 156 6.313 -68.50 -44.00 -60.40 -34.60 -71.00 -15.60 137.00 -9.30 RQVAMGLA *** HHHHTT AAAa

1kv7A 505 510 5.434 -55.00 -47.20 -70.00 -15.40 -114.40 20.00 127.00 -13.30 EHEDTGMM *** HHHHTT E AAAa

1kw3B 227 232 5.406 -71.40 -40.30 -60.80 -31.50 -82.70 -2.80 71.10 26.30 RLDAAGRI *** HHHHTT B AAAa

1kwfA 104 109 5.054 -69.50 -39.00 -64.50 -29.50 -96.10 16.00 56.30 48.80 LAVCFNEQ *** HHHHTT H AAAa

1kwfA 382 387 4.700 -64.50 -50.70 -74.80 -11.00 -97.00 4.00 85.30 21.70 LLYITGNF *** HHHHTT AAAa

1kwgA 301 306 4.847 -72.30 -42.10 -60.30 -34.90 -90.10 17.60 55.40 37.80 YRGVGRGR *** HHHHTTT AAAa

1kwgA 339 344 5.489 -61.70 -42.20 -60.50 -36.40 -82.10 5.50 102.10 3.20 EALAHGAE *** HHHHTT S AAAa

1kwgA 584 589 4.810 -59.30 -44.70 -59.80 -36.50 -82.90 1.40 78.30 17.10 LAAEAGLK *** HHHHTT AAAa

1l3sA 465 470 4.877 -60.90 -48.40 -58.40 -33.80 -83.60 -2.60 64.70 34.70 ELRRNEQD *** HHHHTT H AAAa

1l3sA 674 679 5.576 -74.50 -27.40 -67.70 -26.40 -93.80 0.30 50.40 46.00 EAFRRDLD *** HHHHTT AAAa

1l3sA 687 692 4.905 -70.60 -50.70 -72.70 -28.70 -100.10 3.30 50.10 58.70 AMDIFQVS *** HHHHTT AAAa

1l3sA 815 820 5.460 -71.20 -41.20 -60.00 -38.30 -87.20 -1.60 50.10 40.60 RLKEERLQ *** HHHHTT S AAAa

1l5oA 253 258 5.026 -62.50 -43.50 -58.70 -38.30 -77.90 -5.90 67.50 31.70 GAARaGLP *** HHHHTT AAAa

1l5oA 300 305 5.324 -64.70 -39.90 -72.70 -20.50 -93.70 -3.60 53.30 49.50 ALAHLSME *** HHHHTT AAAa

1l6rA 31 36 5.502 -63.40 -39.10 -62.20 -30.00 -84.20 2.10 111.90 -4.60 SAEKKGLT *** HHHHTT E AAAa

1l6rA 159 164 5.092 -66.60 -48.50 -54.60 -39.10 -100.60 5.80 48.80 54.30 LKEMYSLE *** HHHHTT AAAa

1l6rA 218 223 4.777 -62.50 -43.70 -56.00 -41.90 -90.20 14.40 56.00 42.10 IFKHFELM *** HHHHTT AAAa

1l7aA 105 110 5.750 -66.10 -37.20 -66.50 -26.00 -77.80 -5.10 102.30 11.90 NWALHGYA *** HHHHTT E AAAa

1l8aA 151 156 5.092 -64.50 -47.70 -58.80 -37.70 -74.00 -3.50 77.80 19.50 RAFLEGRL *** HHHHTTSS AAAa

1l8aA 211 216 5.102 -69.00 -42.60 -67.40 -35.50 -78.00 -7.80 76.10 20.00 YLEHRGLK *** HHHHTTS AAAa

1l8aA 245 250 4.916 -81.70 -49.40 -51.40 -45.40 -95.50 14.70 40.40 58.00 IATREKLD *** HHHHTT T AAAa

1l8aA 282 287 5.399 -66.70 -45.50 -59.90 -30.20 -83.30 2.70 85.80 11.00 IFEGAGWN *** HHHHTT E AAAa

1l8aA 421 426 4.926 -68.60 -50.00 -47.20 -45.30 -107.70 16.10 47.20 50.90 IRDRFNVP *** HHHHTT S AAAa

1l8aA 614 619 5.433 -62.40 -42.50 -61.00 -39.80 -81.20 7.40 55.80 43.60 AAGDQQAR *** HHHHTT AAAa

1l8aA 838 843 5.490 -86.10 -43.30 -56.70 -48.70 -80.00 4.50 52.10 52.30 LRHHFEVD *** HHHHTT S AAAa

1l8aA 856 861 5.178 -73.20 -41.10 -63.10 -28.20 -87.50 0.00 74.50 8.30 ELAKRGEI *** HHHHTTSS AAAa

1l8aA 871 876 5.209 -72.00 -45.10 -54.30 -46.70 -84.60 14.10 50.60 52.20 AIAKFNID *** HHHHTT AAAa

1l9lA 59 64 5.326 -62.10 -43.90 -61.70 -33.70 -77.40 -6.90 69.30 29.40 QGLVAGET *** HHHHTT AAAa

1l9xA 40 45 5.791 -62.90 -33.40 -61.10 -23.30 -96.30 9.50 87.50 30.60 YLESAGAR *** HHHHTT E AAAa

1l9xA 97 102 4.763 -59.10 -45.60 -60.90 -21.90 -99.50 10.30 84.30 20.10 QSFDDGDY *** HHHHTT AAAa

1lam_ 28 33 4.953 -60.90 -44.10 -58.10 -33.80 -103.30 29.80 51.60 33.70 FNKLVSGK *** HHHHTTTH AAAa

1lam_ 100 105 5.883 -66.00 -37.20 -64.90 -30.50 -79.70 1.50 62.20 34.80 QIQDLEIP *** HHHHTT S AAAa

1lam_ 208 213 5.813 -67.20 -40.00 -63.80 -36.20 -79.20 3.30 53.20 39.80 WIEEQEMG *** HHHHTT H AAAa

1lam_ 287 292 5.695 -67.60 -40.70 -61.50 -41.50 -74.90 -11.60 54.80 47.70 SAAKLDLP *** HHHHTT S AAAa

1latA 500 505 5.731 -66.20 -53.00 -50.80 -47.20 -60.90 -17.10 81.20 47.60 KCLQAGMN *** HHHHTT AAAa

1lb3A 121 126 4.874 -64.70 -45.90 -62.30 -34.40 -94.80 7.10 51.40 56.60 LGSARADP *** HHHHTT H AAAa

1lbu_ 54 59 4.879 -59.00 -39.70 -65.10 -25.10 -113.90 18.40 62.00 41.40 FQSAYGLA *** HHHHTTS AAAa

1lbu_ 123 128 5.504 -67.70 -41.30 -59.80 -31.90 -83.40 -0.30 105.20 21.80 MRHAMGDK *** HHHHTTS AAAa

1lbwA 208 213 5.541 -63.20 -40.60 -67.70 -22.10 -84.00 5.20 100.40 -0.30 IAEKAGGK *** HHHHTT E AAAa

1lc5A 115 120 5.438 -64.50 -40.50 -63.10 -27.50 -91.40 3.70 80.10 15.50 ALAQSGCE *** HHHHTT E AAAa

1lf2A 143 148 5.147 -67.20 -45.40 -59.80 -32.60 -89.20 4.90 67.00 21.80 ELKNQNKI *** HHHHTTSS AAAa

1lfwA 56 61 5.331 -65.80 -45.60 -55.50 -41.10 -81.00 1.00 97.30 13.20 FAKRDGFD *** HHHHTT E AAAa

1lj8A 38 43 6.044 -70.10 -35.30 -82.40 -12.40 -123.10 -79.70 -62.30 -43.80 FHRAHQAY *** HHHHTHHH AAAA

1lj8A 176 181 4.667 -57.10 -42.60 -68.30 -19.80 -100.20 2.00 71.50 24.90 QRRAAGIP *** HHHHTT AAAa

1lk9A 380 385 5.292 -65.40 -37.50 -64.70 -30.00 -87.70 5.50 76.60 31.30 TFQNGRIN *** HHHHTTEE AAAa

1ll2A 26 31 5.870 -70.60 -41.60 -56.50 -33.00 -99.40 25.70 47.60 61.20 SLKQHRTS *** HHHHTT AAAa

1llfA 142 147 4.966 -64.60 -44.80 -55.20 -36.30 -79.70 -9.60 74.00 17.50 KSVLMGKP *** HHHHTT AAAa

1llfA 263 268 5.158 -64.50 -44.60 -65.90 -26.90 -84.70 2.60 81.30 16.40 FVSSAGbG *** HHHHTT T AAAa

1llfA 322 327 5.045 -71.50 -44.00 -53.70 -40.60 -90.30 3.00 84.90 16.40 KLVRDGKY *** HHHHTT S AAAa

1lm4A 157 162 4.945 -56.60 -32.00 -70.90 -17.90 -106.60 11.70 71.70 29.60 IDHLNGVM *** HHHHTT AAAa

1lml_ 267 272 5.616 -60.60 -49.40 -61.70 -31.10 -78.30 -4.80 122.80 0.60 MAHALGFS *** HHHHTT S AAAa

1lml_ 277 282 5.641 -67.60 -38.50 -65.10 -38.60 -79.90 -8.60 60.80 41.30 FFEDARIV *** HHHHTT E AAAa

1lml_ 362 367 6.742 -71.20 -37.90 -67.90 -18.80 -74.70 -21.40 87.60 -2.60 IFQDLGFY *** HHHHTTS AAAa

1lniA 21 26 4.697 -59.70 -41.70 -61.20 -31.70 -94.00 5.10 61.00 35.40 NLIASDGP *** HHHHTT AAAa

1lo7A 39 44 5.711 -61.70 -43.10 -68.00 -29.20 -76.50 -8.10 106.20 2.90 YFIKCGLP *** HHHHTT AAAa

1louA 30 35 5.206 -61.00 -36.80 -67.80 -26.30 -96.80 13.00 87.20 11.00 AAENYGAR *** HHHHTT E AAAa

1lqtA 130 135 4.896 -64.10 -48.70 -72.70 -18.70 -99.60 13.20 62.70 32.30 VGWYNAHP *** HHHHTT G AAAa

1lqtA 389 394 4.739 -63.50 -40.60 -70.10 -30.00 -89.00 7.30 55.10 43.90 NAKEGAEC *** HHHHTT S AAAa

1ls1A 38 43 5.418 -53.20 -48.70 -67.80 -20.50 -88.70 11.70 67.00 26.50 ALMDADVN *** HHHHTT AAAa

1ls1A 59 64 5.357 -66.00 -30.90 -66.50 -30.70 -89.60 13.10 58.30 43.60 EALGKQVL *** HHHHTTTT AAAa

1ls1A 83 88 5.267 -67.70 -42.20 -61.10 -38.00 -78.70 -9.60 135.80 -8.80 LKEALGGE *** HHHHTTSS AAAa

1ls1A 122 127 5.492 -61.10 -40.60 -63.40 -21.70 -95.70 7.80 95.40 4.40 YYKGKGRR *** HHHHTT AAAa

1ls1A 289 294 5.124 -66.50 -38.60 -59.90 -35.80 -91.10 4.10 85.40 24.80 AGRILGMG *** HHHHTT AAAa

1lshA 380 385 5.552 -58.00 -53.30 -58.70 -30.80 -91.50 9.40 62.80 34.80 RTLASEQL *** HHHHTT S AAAa

1lshA 464 469 5.575 -75.60 -38.70 -58.20 -37.50 -87.80 -1.80 49.60 43.40 QSSDRAKE *** HHHHTT H AAAa

1lst_ 58 63 5.086 -62.00 -48.70 -63.80 -33.60 -82.80 2.20 63.70 26.30 PSLKAKKI *** HHHHTTS AAAa

1lst_ 149 154 5.162 -65.90 -49.80 -68.30 -26.40 -73.60 -8.80 80.80 11.40 SDLTAGRL *** HHHHTTS AAAa

1lst_ 217 222 5.099 -72.50 -43.30 -57.10 -48.20 -84.40 2.90 89.80 1.00 ELRQDGTY *** HHHHTTHH AAAa

1ltzA 110 115 4.839 -62.00 -40.30 -59.00 -22.00 -112.90 21.60 61.60 39.50 EHLANRRF *** HHHHTTEE AAAa

1lu4A 1092 1097 5.465 -74.30 -43.70 -62.40 -34.00 -97.90 12.50 46.20 41.20 IWARYNVP *** HHHHTT AAAa

1lucA 183 188 5.158 -61.10 -40.40 -61.50 -23.50 -108.60 3.80 63.40 29.80 WAAERGLP *** HHHHTT AAAa

1lucA 212 217 5.357 -62.20 -41.80 -60.00 -30.60 -85.20 -7.20 97.80 4.40 VATEHGYD *** HHHHTT AAAa

1lv7A 375 380 5.002 -63.30 -42.20 -62.80 -31.40 -94.70 3.00 59.40 43.50 FAARGNKR *** HHHHTT S AAAa

1lwdA 50 55 4.337 -65.10 -41.10 -65.60 -31.90 -102.60 14.50 54.40 37.70 NRDQTNDQ *** HHHHTTTH AAAa

1lwdA 239 244 5.103 -62.70 -40.70 -60.40 -42.50 -90.90 12.30 57.40 32.60 DFDKYKIW *** HHHHTT AAAa

1lwdA 318 323 5.399 -61.10 -40.10 -64.00 -21.70 -90.70 13.40 73.30 17.10 REHQKGRP *** HHHHTT AAAa

1lwdA 366 371 5.702 -65.10 -45.30 -63.50 -22.60 -79.70 -10.60 110.70 -6.60 ETVESGAM *** HHHHTT AAAa

1lyvA 274 279 5.464 -70.20 -48.80 -62.80 -32.80 -74.00 -8.80 52.70 43.90 MLAENRTP *** HHHHTT S AAAa

1lyvA 383 388 5.204 -66.70 -37.90 -59.80 -33.00 -88.00 -2.10 72.40 31.40 MYESKGSS *** HHHHTT G AAAa

1lzlA 273 278 5.335 -54.70 -46.50 -67.40 -20.20 -87.90 5.20 87.70 9.00 RLLQAGVS *** HHHHTT AAAa

1m0kA 59 64 5.105 -71.80 -42.80 -56.60 -44.00 -81.90 -3.10 96.90 -7.60 LSMLLGYG *** HHHHTTTT AAAa

1m0kA 98 103 5.239 -75.90 -33.80 -58.50 -46.30 -91.80 5.70 59.50 46.00 LALLVDAD *** HHHHTT AAAa

1m0wA 168 173 4.885 -73.30 -56.20 -65.80 -26.00 -94.60 15.10 55.70 43.60 YLNRANKY *** HHHHTTSS AAAa

1m15A 87 92 4.696 -68.10 -53.20 -61.00 -47.30 -85.10 9.10 73.90 31.50 IDDYHGGF *** HHHHTT AAAa

1m15A 181 186 5.314 -75.30 -44.10 -60.20 -34.60 -83.90 5.10 76.50 19.30 QLIDDHFL *** HHHHTT S AAAa

1m15A 195 200 5.224 -58.10 -49.80 -71.20 -14.10 -85.10 6.30 53.70 42.10 FLQTANAC *** HHHHTT S AAAa

1m15A 301 306 4.809 -62.20 -33.30 -71.40 -17.40 -98.10 12.10 59.90 35.40 IASKFNLQ *** HHHHTTEE AAAa

1m1fA 82 87 5.316 -65.10 -49.40 -60.90 -36.60 -78.90 -9.00 73.20 33.70 DMKARGGK *** HHHHT E AAAa

1m1nA 242 247 5.616 -74.20 -32.70 -62.70 -27.30 -79.70 -5.50 95.90 7.00 LLEEMGLR *** HHHHTT E AAAa

1m1nA 431 436 5.487 -60.40 -38.60 -63.80 -30.40 -77.10 -5.70 81.00 22.40 IFQKMGIP *** HHHHTT AAAa

1m1nB 169 174 5.345 -73.30 -40.90 -58.80 -38.80 -91.50 5.50 88.20 9.50 NSKKEGFI *** HHHHTTSS AAAa

1m1nB 383 388 5.080 -61.00 -39.90 -61.30 -29.20 -85.80 -2.00 75.10 22.40 FLLELGCE *** HHHHTT E AAAa

1m22A 56 61 5.316 -67.80 -38.30 -68.10 -25.50 -89.40 -1.80 77.00 17.10 ARMTAGEL *** HHHHTTS AAAa

1m22A 105 110 5.041 -61.60 -46.90 -63.90 -23.40 -90.40 -3.50 77.10 30.90 RERRDGRL *** HHHHTT AAAa

1m22A 154 159 5.537 -68.30 -37.40 -65.10 -31.20 -76.50 -5.30 95.40 3.90 RLRDAGAV *** HHHHTT E AAAa

1m22A 209 214 4.854 -67.60 -41.80 -64.70 -33.30 -93.40 8.40 56.10 44.10 VAVAANLA *** HHHHTTSS AAAa

1m22A 231 236 4.944 -63.70 -44.10 -61.10 -29.50 -107.60 18.50 61.60 36.80 PAAINGVV *** HHHHTTSE AAAa

1m22A 337 342 5.662 -65.50 -44.90 -63.60 -24.30 -80.80 -5.60 109.70 2.60 ELRRAGAV *** HHHHTT E AAAa

1m22A 375 380 4.924 -71.40 -47.20 -57.80 -41.50 -101.80 9.80 48.30 58.20 YFNTHRAP *** HHHHTT S AAAa

1m22A 442 447 4.602 -70.90 -36.70 -57.50 -31.00 -128.80 18.20 46.40 61.20 ALAAHQLD *** HHHHTT S AAAa

1m2jA 86 91 5.612 -79.50 -40.10 -59.70 -39.60 -69.70 -7.80 85.70 6.60 ELERLGVL *** HHHHTT E AAAa

1m2jA 104 109 5.479 -60.50 -45.20 -69.30 -13.40 -89.90 -10.40 113.60 -1.00 LHERAGSR *** HHHHTT AAAa

1m2jA 200 205 4.991 -65.00 -44.60 -59.30 -34.30 -83.80 -2.30 108.80 2.30 IVKQRGGA *** HHHHTT E AAAa

1m2rA 147 152 5.221 -64.30 -42.00 -64.50 -18.20 -101.70 19.30 69.00 29.10 YCHSQGIM *** HHHHTTEE AAAa

1m2rA 201 206 4.787 -76.90 -26.50 -87.50 -17.80 -112.60 5.40 70.50 35.30 PELLVDLQ *** HHHHTT AAAa

1m2rA 258 263 4.802 -67.00 -41.00 -58.40 -46.00 -93.30 11.20 49.70 44.40 YLNKYRIE *** HHHHTT AAAa

1m2xA 128 134 5.434 -69.00 -41.40 -61.20 -36.00 -87.20 11.90 97.60 0.30 YFGKIGAK *** HHHHTT E AAAa

1m2xA 146 167 5.123 -61.00 -45.30 -61.90 -36.20 -89.00 5.40 57.40 43.00 ILAKENKP *** HHHHTT AAAa

1m3uA 103 108 5.856 -64.20 -43.90 -62.30 -31.90 -75.00 -11.00 136.10 -7.30 TVMRAGAN *** HHHHTT S AAAa

1m3uA 126 131 5.187 -65.90 -41.00 -52.70 -37.10 -99.60 25.70 81.30 14.20 MLTERAVP *** HHHHTT AAAa

1m3uA 216 221 5.023 -73.50 -40.90 -59.00 -30.90 -108.50 18.00 64.80 22.40 MHDAFGIT *** HHHHTT S AAAa

1m40A 83 88 4.856 -57.30 -48.20 -60.20 -33.20 -84.00 1.40 73.20 24.80 SRVDAGQE *** HHHHTTS AAAa

1m40A 152 157 5.149 -57.10 -45.40 -64.10 -19.80 -98.00 6.60 97.90 10.00 FLHNMGDH *** HHHHTT S AAAa

1m4iA 29 34 7.248 -71.40 -25.20 -70.90 -20.30 -94.80 9.70 43.40 59.00 VTGAFAGD *** HHHHTTT AAAa

1m4iA 126 131 5.278 -59.60 -36.30 -68.10 -19.30 -95.00 5.80 102.20 -3.40 LYASRGWL *** HHHHTT E AAAa

1m4lA 258 263 5.540 -62.10 -38.10 -74.30 -12.00 -86.20 1.90 94.50 13.20 WSYNQGIK *** HHHHTT AAAa

1m65A 209 214 5.034 -71.40 -36.80 -58.50 -22.00 -113.60 13.20 47.60 47.80 ILDAVDFP *** HHHHTT AAAa

1m65A 231 236 5.525 -64.70 -41.00 -61.00 -27.10 -87.60 -3.20 122.90 -1.60 FLESRGMA *** HHHHTT AAAa

1m6sA 44 49 4.893 -56.90 -49.10 -57.70 -36.40 -100.10 8.10 61.60 33.90 AAETFGKE *** HHHHTT S AAAa

1m7gA 94 99 5.584 -61.20 -41.90 -62.30 -34.90 -73.80 -0.60 78.40 41.20 LFADSNSI *** HHHHTT E AAAa

1m7gA 200 205 5.732 -69.20 -31.80 -62.20 -28.70 -90.10 -6.50 69.40 24.60 YLDTKGYL *** HHHHTT S AAAa

1m93B 104 109 4.673 -67.90 -43.70 -59.30 -37.50 -94.40 17.90 52.50 39.50 VDIFTEGK *** HHHHTTTS AAAa

1m93B 250 255 5.383 -59.40 -45.60 -64.80 -23.00 -84.90 -0.70 113.30 -2.50 ALVKLGLT *** HHHHTT T AAAa

1m9xC 102 107 5.354 -73.50 -41.20 -60.10 -45.10 -72.10 -4.70 78.30 -5.70 GSDIAGTT *** HHHHTTSS AAAa

1mdl_ 116 121 5.085 -70.00 -36.70 -59.80 -40.20 -94.20 13.30 41.10 46.80 LGKVHETP *** HHHHTT B AAAa

1mdl_ 124 129 5.738 -75.40 -28.70 -69.50 -27.50 -79.20 -9.10 98.90 14.70 LVKLLGAN *** HHHHTT AAAa

1mdl_ 155 160 5.449 -61.70 -38.70 -59.60 -43.80 -72.10 2.20 97.70 -9.10 TAAELGFR *** HHHHTT S AAAa

1mdl_ 258 263 5.496 -67.50 -33.50 -60.20 -27.10 -98.40 -2.80 77.90 33.80 KALSIGAC *** HHHHTT AAAa

1mdl_ 287 292 5.108 -65.70 -45.30 -70.40 -37.80 -76.60 -1.60 76.70 35.20 LAQQFGIP *** HHHHTT AAAa

1me4A 38 43 6.024 -60.80 -46.20 -71.70 -15.10 -79.60 2.10 94.00 20.90 QWFLAGHP *** HHHHTTS AAAa

1mf7A 259 264 5.537 -63.10 -42.00 -62.20 -34.80 -84.70 -1.60 78.20 24.10 EADREGVI *** HHHHTTEE AAAa

1mgtA 47 52 5.446 -63.40 -37.80 -67.80 -15.60 -100.50 13.40 83.10 14.20 HLGKRGVS *** HHHHTT AAAa

1mgtA 69 74 4.997 -69.60 -43.40 -54.40 -44.80 -88.70 3.30 83.20 17.60 FKVLIGEL *** HHHHTTSS AAAa

1mgtA 116 121 4.943 -62.80 -37.10 -66.10 -28.00 -107.40 11.30 57.00 39.50 LAKALNTS *** HHHHTTS AAAa

1mgtA 164 169 5.282 -60.30 -44.50 -59.70 -34.50 -85.40 -0.90 84.60 22.80 LLEIEGV# *** HHHHTT AAAa

1mixA 222 227 4.691 -69.20 -49.50 -61.80 -37.80 -87.30 -0.40 89.30 -3.10 DDILNGSH *** HHHHTTSS AAAa

1mj5A 93 98 7.051 -64.20 -46.50 -60.60 -28.70 -77.20 -10.40 57.00 61.70 LWEALDLG *** HHHHTT T AAAa

1mk0A 79 84 5.127 -75.10 -43.50 -58.20 -41.70 -95.60 5.20 47.40 45.50 WIKELNSK *** HHHHTTTT AAAa

1mkaA 14 19 5.031 -56.70 -44.50 -59.20 -22.60 -101.90 4.10 72.20 28.20 LASGRGEL *** HHHHTT S AAAa

1mkaA 94 99 5.305 -70.70 -34.80 -63.10 -35.40 -75.80 -2.30 99.30 8.50 YLGWLGGE *** HHHHTT AAAa

1mla_ 77 82 5.053 -61.80 -46.00 -59.10 -40.70 -82.00 9.70 78.80 22.90 VWQQQGGK *** HHHHTT AAAa

1mla_ 99 104 5.282 -66.90 -50.60 -61.80 -31.60 -81.30 2.70 82.50 10.50 ALVCAGVI *** HHHHTTSS AAAa

1mla_ 183 188 5.308 -62.50 -46.40 -60.10 -35.00 -80.10 2.60 115.90 -1.10 ACKAAGAK *** HHHHTT S AAAa

1mla_ 264 269 5.460 -62.00 -39.40 -67.20 -22.10 -85.20 -5.80 106.50 0.60 YMAAQGVE *** HHHHTT AAAa

1mml_ 81 86 5.072 -59.70 -46.60 -57.10 -44.20 -77.50 0.00 78.80 13.10 RLLDQGIL *** HHHHTTSE AAAa

1mn8A 23 28 5.216 -58.20 -38.30 -66.20 -17.80 -95.70 11.00 61.30 34.90 IAHNQSVD *** HHHHTT AAAa

1moq_ 420 425 5.296 -74.30 -43.40 -61.40 -27.10 -89.00 2.30 63.30 36.70 LSRLKGLD *** HHHHTT AAAa

1msk_ 951 956 5.133 -62.40 -29.90 -68.00 -21.80 -107.60 28.60 45.50 39.90 FFMTWSLA *** HHHHTT AAAa

1msk_ 1076 1081 5.086 -72.00 -41.90 -51.60 -35.90 -100.50 23.00 59.30 25.70 AFEAQHDD *** HHHHTT H AAAa

1msk_ 1124 1129 5.499 -69.20 -47.40 -57.30 -35.90 -80.50 0.50 57.40 41.80 EELIRENY *** HHHHTT S AAAa

1msk_ 1152 1157 4.978 -75.50 -49.10 -57.80 -44.10 -94.30 -2.10 51.70 50.70 IWELLEVE *** HHHHTTHH AAAa

1mtpA 134 139 4.750 -63.10 -47.40 -63.50 -29.80 -98.80 19.30 53.00 37.30 IADATRGM *** HHHHTTTS AAAa

1mtpA 271 276 5.535 -68.40 -42.10 -65.30 -28.00 -79.40 -9.10 123.40 -20.20 VLAEAGVR *** HHHHTT AAAa

1mtyB 127 132 5.791 -64.20 -42.90 -67.30 -30.50 -81.10 -7.80 65.30 26.10 GYSADGQI *** HHHHTTGG AAAa

1mtyD 224 229 4.809 -62.70 -39.90 -64.80 -33.40 -94.30 19.60 82.60 1.60 WAAANGDE *** HHHHTT AAAa

1mtyD 390 395 5.529 -63.00 -40.80 -61.90 -35.20 -76.00 -3.70 84.60 27.30 EWRARGCE *** HHHHTTTT AAAa

1mtyD 408 413 4.998 -65.10 -44.50 -64.70 -35.50 -97.50 16.20 52.50 54.20 WFIENNHP *** HHHHTT AAAa

1mtyD 482 487 5.010 -63.20 -51.30 -57.10 -38.30 -91.30 17.20 57.00 46.10 VIAELHGL *** HHHHTT B AAAa

1mugA 41 46 5.330 -73.60 -55.90 -64.10 -25.90 -82.30 -7.40 103.20 -6.20 VIYQAGFT *** HHHHTTSS AAAa

1mun_ 146 151 4.519 -71.50 -48.80 -56.90 -42.00 -106.70 12.70 52.30 50.80 LARCYAVS *** HHHHTT AAAa

1musA 47 52 7.422 -59.30 -39.40 -62.80 -22.70 -76.00 -8.00 46.90 45.80 ITISSEGS *** HHHHTTT AAAa

1musA 200 205 4.912 -60.50 -45.50 -53.10 -38.60 -88.00 6.70 58.40 34.90 DKLAHNER *** HHHHTT E AAAa

1musA 430 435 5.323 -56.30 -40.70 -59.80 -28.00 -89.10 2.10 101.20 -5.40 IARLGGFM *** HHHHTT AAAa

1musA 469 474 5.221 -60.20 -52.20 -46.00 -24.40 -108.00 25.50 68.00 4.50 DLMAQGIK *** HHHHTT AAAa

1muwA 234 239 5.241 -63.10 -40.30 -60.30 -37.00 -80.20 -8.20 73.80 30.00 QALWAGKL *** HHHHTT B AAAa

1muwA 329 334 5.475 -64.10 -41.80 -59.50 -32.80 -90.80 3.10 66.40 29.30 ALRASRLD *** HHHHTTHH AAAa

1mwqA 31 36 5.249 -62.90 -41.60 -61.50 -31.60 -78.60 -2.30 57.80 35.60 QLQAENRL *** HHHHTT E AAAa

1mwqA 80 85 4.911 -72.30 -60.20 -59.00 -32.50 -78.40 -8.30 80.80 25.40 PYVEAGVY *** HHHHTT E AAAa

1mxiA 42 47 7.050 -59.10 12.80 -134.30 -19.70 -99.10 -59.40 -66.10 -15.00 DKRLRRSG *** HHHHTTS AAAA

1mxiA 145 150 5.066 -59.60 -48.20 -61.10 -33.00 -90.40 12.10 75.80 18.30 AWRQLGYK *** HHHHTTTT AAAa

1mxrA 218 223 5.561 -63.60 -34.50 -63.60 -26.40 -86.50 4.30 68.80 28.60 AFAERELM *** HHHHTT AAAa

1mxrA 316 321 5.109 -60.50 -35.50 -65.50 -20.80 -113.60 20.40 82.60 13.70 RMQAVGLD *** HHHHTT AAAa

1n0qA 21 26 5.441 -63.00 -41.10 -65.20 -30.30 -77.30 -3.10 95.20 21.80 LLLEAGAD *** HHHHTT AAAa

1n0qA 42 47 5.239 -68.00 -44.80 -55.60 -39.00 -96.80 5.30 75.30 25.60 LAARNGHL *** HHHHTT H AAAa

1n0qA 54 59 5.461 -62.10 -41.80 -65.00 -27.10 -84.60 7.00 81.60 23.10 LLLEAGAD *** HHHHTT AAAa

1n0qA 75 80 5.198 -65.80 -44.50 -57.20 -41.10 -92.40 1.10 77.90 27.90 LAARNGHL *** HHHHTT H AAAa

1n0qA 87 92 5.357 -60.20 -39.70 -66.40 -27.50 -84.30 5.30 93.90 14.70 LLLEAGAY *** HHHHTT AAAa

1n1jA 103 108 5.354 -62.20 -42.70 -64.90 -27.50 -88.90 13.60 61.50 34.30 RCHQEKRK *** HHHHTT S AAAa

1n1jA 120 125 4.790 -68.40 -39.80 -70.00 -34.40 -89.80 5.90 102.60 7.40 AMSTLGFD *** HHHHTT G AAAa

1n2eA 27 32 5.572 -60.60 -40.00 -64.30 -21.30 -94.60 2.70 106.00 -15.90 ALRLTGRR *** HHHHTT E AAAa

1n2eA 93 98 5.394 -63.50 -44.10 -62.50 -25.20 -90.20 -1.30 75.20 21.10 QLRAEGVE *** HHHHTT AAAa

1n2eA 172 177 4.870 -68.10 -46.50 -65.20 -31.90 -104.30 12.20 56.50 36.70 LVADFNLD *** HHHHTT AAAa

1n2zA 37 42 5.079 -60.10 -40.20 -65.50 -22.60 -89.50 -5.80 99.80 6.70 LAFAAGIT *** HHHHTT AAAa

1n2zA 180 185 5.058 -65.20 -40.80 -63.30 -27.20 -89.00 9.00 87.60 20.40 VLEVaGGE *** HHHHTTEE AAAa

1n3lA 62 67 5.327 -64.70 -39.10 -67.20 -30.00 -75.90 -10.90 100.40 7.20 DFLKAGCE *** HHHHTT E AAAa

1n3lA 174 179 5.861 -73.60 -42.70 -75.10 -25.90 -82.30 -4.80 57.10 50.00 DEEYLKVD *** HHHHTT S AAAa

1n3lA 298 303 5.582 -65.50 -42.10 -64.40 -32.70 -78.50 -7.60 66.40 34.10 KDFAAEVV *** HHHHTT S AAAa

1n40A 89 94 5.290 -64.00 -44.00 -62.50 -30.50 -78.90 -5.50 79.50 18.80 NIADAGLR *** HHHHTT H AAAa

1n4wA 29 34 5.338 -62.40 -40.50 -66.50 -26.00 -82.60 -1.10 84.70 16.30 RLGEAGVQ *** HHHHTT AAAa

1n4wA 181 186 5.234 -61.40 -32.50 -65.90 -22.60 -93.70 8.40 82.60 5.60 QAGKAGLG *** HHHHTT AAAa

1n4wA 202 207 5.154 -59.20 -46.40 -64.80 -31.50 -81.40 -0.40 74.00 20.80 QREAAGEV *** HHHHTTSS AAAa

1n4wA 236 241 7.654 -58.30 -36.90 -64.50 -17.90 -70.10 -21.80 84.50 -1.80 AALGTGKV *** HHHHTTSE AAAa

1n4wA 301 306 4.925 -62.40 -52.30 -83.70 -12.20 -101.70 2.80 84.00 12.40 RARDTGTL *** HHHHTTSS AAAa

1n55A 116 121 5.421 -63.40 -42.40 -59.10 -33.80 -87.60 10.30 83.90 18.30 EACKQGFM *** HHHHTT E AAAa

1n55A 133 138 5.063 -62.30 -45.10 -61.70 -30.20 -84.10 6.00 61.40 36.90 QQREANQT *** HHHHTT H AAAa

1n62A 94 99 4.496 -71.50 -48.50 -59.80 -38.50 -99.60 5.70 57.80 52.50 GFRMMHGL *** HHHHTT AAAa

1n62B 469 474 5.143 -63.90 -47.60 -67.70 -24.00 -82.10 -10.70 85.70 27.70 EAFKRGET *** HHHHTTS AAAa

1n62B 802 807 5.151 -54.70 -50.50 -66.60 -27.90 -78.40 -9.80 85.70 19.40 VGEQLGLH *** HHHHTTTT AAAa

1n62C 40 45 5.281 -70.60 -39.70 -65.30 -26.60 -91.20 -3.50 56.70 30.30 PIMKTRLA *** HHHHTTS AAAa

1n7hA 48 53 5.523 -62.30 -38.20 -65.40 -22.10 -90.70 3.20 87.90 12.50 FLLGKGYE *** HHHHTT E AAAa

1n7hA 361 366 4.841 -55.60 -34.60 -77.40 -47.60 -64.70 3.00 90.80 6.90 VLVDAGY# *** HHHHTT AAAa

1n8kA 211 216 5.569 -68.10 -41.10 -63.20 -31.80 -82.10 -3.90 89.90 18.60 GCKAAGAA *** HHHHTT S AAAa

1n8kA 255 260 4.989 -64.60 -43.90 -64.70 -34.20 -96.10 14.80 53.90 42.40 LTEMSNGG *** HHHHTTS AAAa

1n8kA 307 312 6.595 -85.00 -39.40 -60.90 -28.80 -67.40 -21.60 137.20 -5.60 MLLLSGRT *** HHHHTT E AAAa

1n8kA 334 339 5.372 -60.40 -44.50 -59.90 -33.50 -87.20 11.70 67.30 29.50 ADFMAKKF *** HHHHTTSS AAAa

1n8kA 361 366 5.420 -66.40 -49.40 -66.00 -29.40 -75.60 -11.10 90.20 -3.20 DLLRSGES *** HHHHTT AAAa

1n8vA 28 33 5.327 -64.20 -52.10 -67.50 -28.50 -81.70 3.20 60.40 41.50 VNaVMERG *** HHHHTTSS AAAa

1n93X 90 95 5.372 -54.70 -44.40 -62.10 -37.00 -72.40 -5.30 97.20 10.20 AFVHGGVP *** HHHHTT AAAa

1n93X 170 175 5.064 -61.20 -43.80 -61.50 -25.00 -96.20 14.00 57.80 32.50 MMAALNRP *** HHHHTT T AAAa

1n93X 361 366 4.874 -56.60 -32.30 -60.70 -27.40 -112.50 12.90 83.30 18.90 IMKMIGVT *** HHHHTT AAAa

1na3A 14 19 5.238 -55.00 -45.60 -68.70 -21.50 -78.60 -9.10 76.90 23.10 AYYKQGDY *** HHHHTT H AAAa

1na3A 48 53 5.096 -52.20 -48.30 -63.20 -29.20 -84.70 -1.30 78.60 16.60 AYYKQGDY *** HHHHTT H AAAa

1nar_ 249 254 5.022 -65.30 -35.10 -65.80 -25.30 -109.70 18.20 53.20 44.70 RLVQTFSL *** HHHHTT AAAa

1ndbA 152 157 4.802 -71.70 -44.60 -61.80 -41.50 -88.60 5.10 49.30 35.60 SXIDNETL *** HHHHTT S AAAa

1ndbA 422 427 4.893 -59.60 -39.10 -65.40 -19.80 -99.90 3.80 65.90 35.60 FPKSEKLS *** HHHHTT AAAa

1ndbA 509 514 4.920 -59.50 -44.00 -66.50 -23.80 -95.30 13.20 75.30 23.20 DRAIRGEA *** HHHHTT AAAa

1ndbA 527 532 5.723 -62.70 -44.60 -68.20 -33.00 -75.90 -6.10 63.20 34.30 QAIEDLVS *** HHHHTT AAAa

1ne9A 121 126 5.567 -64.10 -39.30 -60.20 -30.00 -86.00 5.90 94.60 -3.00 TLQDHGYV *** HHHHTT E AAAa

1ne9A 176 181 5.334 -64.60 -40.90 -68.00 -28.10 -84.20 10.50 96.70 -2.80 RPFRDGVE *** HHHHTTEE AAAa

1ne9A 203 208 5.042 -65.70 -35.80 -67.30 -38.20 -93.30 12.50 52.80 31.60 MAERHGIT *** HHHHTT AAAa

1ne9A 279 284 5.021 -66.40 -48.90 -58.80 -27.20 -101.30 9.60 53.40 42.20 WALDTNTD *** HHHHTT S AAAa

1nf9A 137 142 5.116 -57.90 -46.80 -68.60 -21.30 -94.60 -0.10 66.50 29.20 RMRAAGRD *** HHHHTT AAAa

1nf9A 163 168 5.133 -52.20 -46.40 -59.00 -35.30 -84.30 13.50 60.20 33.00 DAYSNDIQ *** HHHHTT E AAAa

1nfp_ 85 90 5.133 -62.60 -33.80 -53.80 -36.60 -106.40 7.50 64.50 28.20 KAAKYGMP *** HHHHTT AAAa

1nfp_ 185 190 4.975 -61.00 -39.40 -59.40 -29.60 -106.40 32.60 57.30 23.50 MAQGLNNK *** HHHHTTT AAAa

1nfp_ 222 227 4.541 -64.80 -45.30 -61.50 -38.80 -97.30 4.90 58.50 44.20 YRKEHNLN *** HHHHTT AAAa

1nh8A 20 25 5.519 -64.50 -40.90 -62.00 -33.60 -79.90 2.00 95.30 9.50 ILAEAGYR *** HHHHTT AAAa

1nh8A 143 148 5.120 -75.50 -48.50 -54.80 -43.90 -84.30 6.50 81.60 10.50 ISVQLGVA *** HHHHTTS AAAa

1nh8A 162 167 4.863 -60.00 -37.10 -65.00 -23.70 -113.90 21.10 55.00 45.60 TLSQHDLV *** HHHHTTEE AAAa

1nh8A 266 271 5.282 -60.10 -35.80 -67.50 -21.60 -101.40 7.90 103.10 7.40 ELAAIGAK *** HHHHTT E AAAa

1ni9A 23 28 5.278 -73.70 -32.20 -66.00 -31.30 -82.60 7.00 67.50 4.10 KWLGRGDK *** HHHHTT H AAAa

1ni9A 147 152 5.315 -60.20 -39.90 -61.10 -37.10 -83.20 3.90 73.30 30.30 VAAALGKP *** HHHHTT AAAa

1ni9A 174 179 5.669 -59.80 -55.10 -51.60 -35.90 -78.90 -9.10 73.10 29.10 EMQQLGVR *** HHHHTT E AAAa

1ni9A 221 226 5.041 -60.20 -43.60 -56.40 -34.80 -99.20 4.70 63.30 30.70 VIRALDGD *** HHHHTT E AAAa

1nkiA 81 86 5.460 -57.70 -43.40 -68.70 -26.00 -79.60 -4.60 90.60 12.20 QLRAHGVR *** HHHHTT AAAa

1nlnA 12 17 5.644 -68.40 -40.80 -61.40 -34.90 -79.60 6.30 88.50 9.90 IVKDLGCG *** HHHHTT G AAAa

1nm1G 85 90 4.984 -56.90 -45.60 -60.00 -37.50 -86.40 28.30 57.50 26.70 LDDYLNGR *** HHHHTTT AAAa

1nnfA 65 70 5.221 -62.10 -41.40 -67.60 -26.30 -88.80 3.50 70.10 28.80 DLSEAGLL *** HHHHTT B AAAa

1nnfA 181 186 4.966 -70.10 -47.40 -57.20 -37.60 -89.60 0.10 85.90 9.50 QAVENGEV *** HHHHTTS AAAa

1nnhA 29 34 5.541 -65.00 -45.20 -67.30 -26.80 -75.40 -4.20 90.90 18.80 FFVKEGFK *** HHHHTT E AAAa

1nnhA 83 88 6.091 -66.50 -36.30 -63.60 -24.60 -81.10 3.10 77.70 21.30 LAIAMGLK *** HHHHTT AAAa

1nnhA 233 238 5.536 -63.80 -41.20 -63.10 -33.60 -84.20 -7.50 76.10 21.90 KIRKAGLN *** HHHHTT AAAa

1nnhA 249 254 5.845 -65.50 -43.40 -63.00 -34.80 -73.10 -8.30 84.30 17.20 EIAKAGKL *** HHHHTT AAAa

1nofA 101 106 5.228 -64.80 -38.10 -60.20 -33.90 -88.10 11.80 93.60 4.50 QAVSLGAK *** HHHHTT E AAAa

1nofA 150 155 5.014 -64.10 -45.50 -60.90 -30.50 -93.30 9.30 86.40 18.20 YMQTNGAP *** HHHHTT AAAa

1nofA 242 247 5.561 -62.80 -43.90 -71.80 -21.20 -81.10 -5.90 77.30 26.10 LAQNAGKQ *** HHHHTT E AAAa

1nofA 278 283 5.696 -67.20 -40.20 -64.60 -26.70 -80.60 5.40 72.40 25.00 ASMVSNYS *** HHHHTTEE AAAa

1nogA 68 73 4.624 -63.10 -46.50 -67.70 -42.70 -87.10 10.20 59.00 24.50 EDVSTGGK *** HHHHTTTS AAAa

1nogA 161 166 4.916 -59.00 -45.30 -71.30 -17.40 -100.00 9.50 62.10 36.70 SNKRLNIP *** HHHHTT AAAa

1nox_ 67 72 5.152 -64.20 -38.70 -66.70 -24.30 -98.90 34.10 56.00 32.50 LREAAFGQ *** HHHHTTT AAAa

1nox_ 147 152 5.217 -60.70 -42.50 -64.40 -26.30 -94.10 2.40 85.50 19.50 LLEAYGLG *** HHHHTT E AAAa

1npk_ 19 24 5.394 -70.50 -43.00 -65.70 -31.60 -86.20 6.70 69.20 39.40 DGVARGLV *** HHHHTT H AAAa

1npsA 28 33 5.078 -60.10 -37.30 -68.50 -20.60 -99.10 10.50 81.60 18.90 QLAALGIE *** HHHHTT AAAa

1npyA 111 116 5.475 -64.40 -49.10 -54.50 -42.90 -87.40 6.10 49.20 47.50 LIEKYHLN *** HHHHTT AAAa

1npyA 138 143 5.503 -66.20 -46.10 -78.40 -7.00 -91.70 2.50 93.70 16.20 AFKNSGFE *** HHHHTT AAAa

1npyA 225 230 5.221 -65.20 -37.00 -68.30 -18.30 -105.20 6.80 69.60 31.50 YAQARGKQ *** HHHHTT E AAAa

1nquA 38 43 5.238 -63.10 -44.70 -79.00 -10.30 -88.70 1.50 105.90 0.00 CIVRHGGR *** HHHHTT AAAa

1nrjA 113 118 6.123 -77.80 -21.30 -74.00 -23.50 -102.50 10.00 50.70 40.00 LWNSLHLS *** HHHHTTHH AAAa

1ns5A 145 150 4.752 -64.50 -45.70 -65.90 -24.40 -110.70 6.50 55.30 38.30 WSITTNHP *** HHHHTT T AAAa

1nthA 40 45 5.249 -73.30 -43.80 -58.60 -37.70 -96.20 0.50 55.40 42.80 LKQKYGLD *** HHHHTT AAAa

1nthA 191 196 5.128 -62.50 -40.20 -66.10 -23.00 -90.10 6.90 79.10 21.30 ACAMAGRP *** HHHHTT T AAAa

1nthA 249 254 5.136 -61.70 -43.30 -66.10 -14.80 -107.70 3.60 60.50 36.50 HYKGNSDI *** HHHHTT E AAAa

1nu0A 133 138 5.637 -72.80 -38.90 -65.00 -37.00 -75.40 -1.10 82.90 27.40 SYXEQGY# *** HHHHTT AAAa

1nuuA 208 213 5.080 -58.50 -41.30 -64.00 -26.50 -93.40 5.90 77.70 10.30 RALGQGQS *** HHHHTT AAAa

1nuuA 227 232 4.969 -64.60 -48.90 -62.00 -31.60 -105.40 9.00 55.80 42.90 YIKDHGLY *** HHHHTT S AAAa

1nuyA 1085 1090 7.356 -58.00 -44.50 -74.60 -9.20 -73.40 -19.00 62.10 19.10 VLKSSFAT *** HHHHTT E AAAa

1nuyA 1288 1293 4.820 -64.50 -34.80 -69.80 -19.80 -93.70 4.40 89.00 17.20 VMEKAGGL *** HHHHTT E AAAa

1nvmA 20 25 4.599 -58.50 -41.50 -65.30 -28.00 -105.80 15.50 51.70 41.80 GSHAIRHQ *** HHHHTTT AAAa

1nvmA 215 220 5.483 -64.70 -41.40 -62.80 -28.80 -87.90 10.90 102.00 3.30 VAVEEGCD *** HHHHTT AAAa

1nvmB 48 53 5.115 -63.20 -42.20 -65.80 -27.70 -94.50 10.90 80.50 7.40 RAQRMGVT *** HHHHTT AAAa

1nxuA 16 21 5.349 -62.80 -39.60 -64.40 -19.40 -93.60 7.50 87.40 21.40 VLISRGVD *** HHHHTT AAAa

1nxuA 36 41 5.402 -64.10 -46.00 -66.60 -24.80 -78.60 -9.20 107.20 8.80 RTTESGVY *** HHHHTT AAAa

1nxuA 55 60 5.234 -72.10 -45.10 -66.00 -25.10 -82.50 -5.90 90.80 -6.00 QQLENGDI *** HHHHTTSS AAAa

1nxuA 127 132 5.244 -60.80 -34.30 -62.50 -32.00 -85.50 1.90 91.70 2.70 QAAEKGYI *** HHHHTT E AAAa

1nxuA 186 191 5.374 -60.90 -44.00 -63.50 -25.60 -91.10 0.10 64.80 37.20 VNRLAGRQ *** HHHHTT AAAa

1ny1A 52 57 4.738 -65.70 -31.10 -64.90 -21.10 -120.60 18.80 63.10 9.70 LIEKYDAF *** HHHHTT B AAAa

1ny1A 88 93 5.061 -68.80 -44.80 -55.40 -41.40 -91.90 0.30 50.30 47.60 VLKKHRVT *** HHHHTT AAAa

1ny1A 114 119 4.984 -65.60 -49.70 -66.80 -28.40 -92.10 17.70 78.00 12.30 RXSDEGHI *** HHHHTT E AAAa

1ny1A 177 182 5.281 -66.40 -36.30 -64.70 -30.40 -83.00 4.00 85.70 12.70 ETKRLGYQ *** HHHHTT E AAAa

1nytA 50 55 5.338 -68.80 -39.50 -59.30 -33.90 -76.70 -7.90 119.00 0.90 AFFSAGGK *** HHHHTT AAAa

1nytA 138 143 6.546 -69.30 -23.40 -67.40 -19.70 -92.00 15.80 49.50 54.90 PLLSLDCA *** HHHHTT E AAAa

1nytA 226 231 5.435 -65.00 -44.00 -54.60 -33.40 -93.10 2.00 116.70 -3.60 WCEQRGSK *** HHHHTT AAAa

1nzjA 33 38 4.969 -66.90 -35.40 -65.10 -32.30 -87.70 11.20 56.10 38.70 QARARQGR *** HHHHTT E AAAa

1nzjA 64 69 5.078 -64.80 -37.80 -63.10 -35.10 -91.00 7.00 83.10 11.50 QLEHYGLH *** HHHHTT AAAa

1nzjA 92 97 4.977 -67.00 -44.30 -62.40 -32.40 -88.80 5.70 80.20 22.20 WLHEQGLS *** HHHHTT E AAAa

1nzjA 108 113 5.203 -60.10 -39.20 -67.90 -24.50 -100.10 8.60 109.00 5.10 RIQSIGGI *** HHHHTTSS AAAa

1nzjA 180 185 5.236 -58.70 -48.80 -56.10 -42.50 -83.00 -1.10 75.10 21.60 DDHFQGVT *** HHHHTT AAAa

1nzjA 251 256 5.255 -59.30 -42.70 -57.60 -39.00 -83.70 11.10 73.60 16.30 ALQFLGQQ *** HHHHTT AAAa

1nzyA 137 142 5.115 -53.90 -43.50 -61.70 -32.50 -86.60 -4.10 84.20 17.80 AWHTIGIG *** HHHHT AAAa

1nzyA 251 256 5.357 -65.20 -41.00 -68.30 -22.20 -92.80 26.80 83.30 -2.20 TRFLDGHR *** HHHHTT AAAa

1o08A 1028 1033 4.889 -63.20 -34.60 -58.70 -34.80 -97.80 18.90 74.60 20.60 LAEEIGIN *** HHHHTT AAAa

1o08A 1103 1108 5.046 -64.00 -40.20 -60.60 -36.60 -91.60 13.20 55.50 36.30 DLRSNKIK *** HHHHTT E AAAa

1o08A 1123 1128 4.945 -65.10 -42.90 -52.90 -42.10 -90.40 6.60 54.10 44.00 LLERMNLT *** HHHHTT G AAAa

1o08A 1155 1160 5.233 -56.20 -39.40 -64.40 -20.30 -112.10 13.90 68.70 29.30 AAHAVGVA *** HHHHTT AAAa

1o1yA 21 26 4.802 -63.70 -42.90 -55.80 -37.50 -97.50 7.80 49.00 35.00 IFREKNWS *** HHHHTT E AAAa

1o1yA 95 100 5.394 -59.90 -46.40 -60.20 -33.60 -86.70 9.70 97.40 4.60 LAKVLGAS *** HHHHTT AAAa

1o1yA 192 197 5.144 -62.40 -46.10 -56.80 -42.40 -88.40 -0.80 55.20 47.50 ELEKKKID *** HHHHTT AAAa

1o1zA 24 29 5.562 -67.30 -39.90 -62.30 -32.80 -79.00 -4.10 100.70 11.10 KAIEAGAN *** HHHHTT S AAAa

1o1zA 71 76 4.398 -62.70 -47.30 -63.00 -33.10 -102.70 15.00 37.20 48.70 LKELTDGK *** HHHHTTT AAAa

1o1zA 182 187 5.636 -64.90 -36.20 -61.70 -30.70 -79.60 -6.60 104.00 3.50 SFRKKGIV *** HHHHTT E AAAa

1o20A 55 60 5.486 -69.00 -46.30 -45.60 -43.40 -80.70 6.90 62.40 31.50 KARERGVK *** HHHHTT AAAa

1o20A 135 140 4.787 -59.60 -42.90 -61.10 -38.60 -86.00 10.30 77.80 17.50 LALKSGNT *** HHHHTT AAAa

1o20A 279 284 5.182 -64.20 -30.90 -64.10 -26.60 -98.10 16.80 75.30 22.30 ELRKHGVE *** HHHHTT E AAAa

1o2dA 53 58 5.058 -64.80 -44.80 -58.40 -43.60 -84.60 -3.70 57.80 46.50 LLDETEIS *** HHHHTT E AAAa

1o2dA 224 229 5.427 -62.80 -42.50 -58.00 -39.90 -75.20 -5.20 104.70 5.80 PKAIEGNR *** HHHHTT H AAAa

1o2dA 297 302 4.988 -64.10 -43.00 -60.60 -38.90 -87.70 29.90 88.00 6.50 VNHIFGGS *** HHHHTTT AAAa

1o2dA 308 313 5.566 -63.10 -40.30 -63.80 -26.10 -84.20 -1.60 76.00 29.90 FLKELGLY *** HHHHTT AAAa

1o3uA 14 19 4.928 -57.20 -45.30 -61.90 -32.30 -96.50 6.50 83.90 13.30 HDLEHGFY *** HHHHTT H AAAa

1o4sA 24 29 5.278 -60.90 -48.80 -48.00 -35.50 -86.90 12.30 70.90 24.50 ALIKKGED *** HHHHTT AAAa

1o4sA 130 135 5.204 -62.90 -48.20 -55.90 -33.40 -82.40 -0.10 93.80 4.70 QIILAGGT *** HHHHTT E AAAa

1o4sA 301 306 5.361 -57.00 -46.80 -61.30 -35.00 -86.00 4.00 84.70 19.60 RLKKMGVK *** HHHHTT AAAa

1o4vA 24 29 5.121 -59.40 -41.00 -61.00 -36.50 -86.50 0.60 62.70 31.00 ILEEFGID *** HHHHTT E AAAa

1o4wA 35 40 5.524 -57.10 -38.50 -59.60 -35.40 -80.60 -1.70 103.50 8.30 QLREFGFS *** HHHHTTEE AAAa

1o4wA 113 118 4.860 -58.80 -45.60 -61.80 -24.80 -104.10 9.40 69.00 22.00 KAKQRGIP *** HHHHTT AAAa

1o54A 93 98 5.773 -72.00 -41.30 -62.10 -36.30 -74.10 -4.00 56.90 33.50 IAMMLDVK *** HHHHTT AAAa

1o54A 145 150 5.155 -65.60 -49.40 -57.50 -34.30 -92.00 4.20 74.80 25.40 NLTKWGLI *** HHHHTT G AAAa

1o69A 64 69 5.500 -65.90 -46.50 -70.40 -25.90 -68.20 -10.80 94.80 14.30 ALRVAGVK *** HHHHTT AAAa

1o69A 89 94 5.208 -62.70 -35.40 -58.00 -39.80 -89.20 -0.60 60.20 34.80 PICYLKAK *** HHHHTT E AAAa

1o69A 145 150 4.631 -57.00 -48.20 -66.30 -27.10 -101.90 7.50 55.80 48.30 ICKENDIV *** HHHHTT E AAAa

1o69A 321 326 4.957 -57.00 -32.70 -75.90 -20.80 -100.50 21.40 63.80 25.50 DLKNKQIE *** HHHHTT AAAa

1o6vA 58 63 5.071 -64.90 -48.00 -56.20 -39.70 -91.50 -0.20 73.80 30.70 MKTVLGKT *** HHHHTT S AAAa

1o7jA 237 242 5.820 -54.40 -51.50 -62.90 -25.40 -93.60 15.10 83.60 19.30 AAIQHGVK *** HHHHTT S AAAa

1o7jA 266 271 5.204 -59.50 -39.30 -64.70 -24.50 -92.80 5.60 82.90 9.70 KALEKGVV *** HHHHTT E AAAa

1o7nB 544 549 5.163 -66.50 -40.90 -63.30 -34.80 -88.20 7.00 63.10 36.90 HLLDIQAY *** HHHHTT H AAAa

1o7qA 305 310 5.187 -56.60 -40.90 -70.30 -23.30 -101.20 15.00 56.60 44.60 KDKKNDIE *** HHHHTT AAAa

1o83A 18 23 4.945 -55.80 -50.50 -65.20 -30.60 -82.60 -1.40 75.50 29.70 NVVEAGGW *** HHHHTT AAAa

1o8xA 101 106 4.984 -66.20 -45.20 -55.40 -42.20 -91.80 8.10 50.20 48.00 LSKHFNVE *** HHHHTT AAAa

1o98A 85 90 4.802 -70.60 -45.20 -59.10 -39.70 -90.70 1.80 75.40 23.70 IAIREGEF *** HHHHTT G AAAa

1o98A 136 141 5.228 -64.00 -43.50 -60.80 -40.80 -82.60 0.50 73.70 27.40 LAAKEGVK *** HHHHTT AAAa

1o98A 223 228 5.108 -59.60 -38.80 -61.90 -25.60 -95.20 8.10 76.20 12.30 DSYKHGIY *** HHHHTT AAAa

1o98A 321 326 5.335 -64.20 -46.60 -57.50 -32.70 -92.00 14.20 70.90 19.30 VLSQHGLR *** HHHHTT AAAa

1o98A 386 391 6.617 -64.80 -41.70 -62.80 -28.00 -79.80 -5.80 52.10 52.40 KEIEADKY *** HHHHTT AAAa

1o98A 432 437 5.001 -64.60 -36.80 -61.10 -29.50 -88.10 3.80 79.80 16.10 AILAKGGI *** HHHHTT E AAAa

1o9gA 138 143 5.438 -59.30 -46.00 -73.70 -25.10 -98.70 9.10 144.50 -11.20 RLTAEGGA *** HHHHTTSS AAAx

1o9rA 73 78 5.210 -69.30 -34.90 -56.40 -36.30 -84.50 6.30 94.70 2.50 RVVQLGGT *** HHHHTT AAAa

1o9rA 128 133 5.655 -62.60 -42.00 -62.30 -31.20 -76.50 -5.00 86.80 25.90 DSDEAGDP *** HHHHTT H AAAa

1oaa_ 226 231 4.950 -65.00 -52.40 -62.70 -33.70 -81.40 0.10 64.90 27.60 KLKSDGAL *** HHHHTT S AAAa

1oaiA 589 594 4.717 -63.10 -39.50 -63.40 -20.70 -119.10 30.20 63.30 27.70 CLQDNNWD *** HHHHTTT AAAa

1oaiA 606 611 5.555 -61.20 -46.40 -60.00 -36.10 -72.70 -9.70 75.10 18.60 HLKAKGEI *** HHHHTT S AAAa

1ob8A 17 22 5.218 -65.00 -39.90 -59.60 -31.50 -85.90 -0.50 95.30 9.60 ILRGEGFN *** HHHHTT E AAAa

1obdA 206 211 5.267 -61.10 -40.40 -64.60 -27.50 -83.50 -2.20 91.20 9.90 YAKEKGII *** HHHHTTEE AAAa

1obdA 267 272 4.734 -62.00 -43.80 -59.30 -28.80 -107.90 10.30 57.40 37.30 WLTANKLN *** HHHHTT T AAAa

1obfO 20 25 5.210 -74.10 -42.10 -65.30 -30.60 -84.30 7.50 79.10 24.90 AHYEGGKS *** HHHHTTS AAAa

1obfO 266 271 5.612 -69.00 -19.70 -99.00 -5.50 -125.20 -115.80 -55.20 -33.50 AASEGELK *** HHHHTTTT AAAA

1oboA 1109 1114 5.515 -65.50 -41.20 -62.70 -30.90 -84.00 7.30 109.80 -11.30 KISQRGGK *** HHHHTT E AAAa

1od6A 78 83 5.431 -66.50 -40.60 -57.90 -44.10 -89.90 0.60 64.70 40.60 FVRRVGAQ *** HHHHTT S AAAa

1od6A 107 112 5.140 -92.70 -42.60 -62.80 -42.80 -114.40 85.00 -35.90 115.70 NRQLYPGL *** HHHHTTT AADP

1od6A 135 140 4.889 -64.60 -35.10 -59.10 -33.90 -98.40 17.20 80.10 12.70 EIARYGGD *** HHHHTT AAAa

1od6A 155 160 4.527 -71.80 -44.60 -64.50 -48.20 -85.30 -3.00 61.10 49.60 LKAKLGQ# *** HHHHTT AAAa

1odmA 35 40 5.543 -69.80 -27.80 -86.70 -16.10 -95.80 -114.40 -75.20 -20.00 AASRDTGF *** HHHHTTSE AAAA

1odmA 116 121 5.394 -70.70 -44.00 -58.10 -42.10 -75.90 -9.80 59.60 37.30 PRIQAKTP *** HHHHTT T AAAa

1odmA 161 166 5.144 -60.80 -38.80 -60.30 -34.00 -90.20 8.90 81.40 17.10 YALALGKE *** HHHHTTS AAAa

1odmA 258 263 4.384 -61.50 -50.50 -60.80 -34.90 -99.70 9.90 54.90 38.30 MAHLTNNY *** HHHHTTTS AAAa

1odzA 131 136 5.152 -62.20 -37.90 -68.20 -26.50 -79.60 -1.10 78.80 16.90 KAYARGGI *** HHHHTT E AAAa

1odzA 327 332 5.502 -62.90 -38.20 -69.00 -23.70 -83.90 1.90 86.50 8.60 PDIEAGLY *** HHHHTT AAAa

1oe4A 236 241 5.545 -71.00 -32.10 -62.10 -36.70 -91.70 -2.10 67.70 36.10 ALMAEGID *** HHHHTT S AAAa

1oejA 16 21 5.042 -59.60 -39.20 -65.30 -23.90 -100.70 20.80 78.70 22.70 QKAANGVF *** HHHHTT AAAa

1oejA 45 50 5.108 -66.70 -48.90 -58.40 -35.30 -83.00 -8.80 93.10 -1.30 PLLQSGKL *** HHHHTTTT AAAa

1of1A 106 111 5.155 -66.60 -50.70 -55.60 -36.20 -82.60 -6.50 78.90 20.60 HRLDQGEI *** HHHHTTSS AAAa

1of1A 176 181 5.224 -59.60 -47.20 -63.40 -21.60 -91.10 6.40 72.10 9.20 ARYLMGSM *** HHHHTTSS AAAa

1of1A 248 253 5.286 -67.70 -42.70 -62.80 -30.20 -88.00 11.80 92.20 8.70 RYLQCGGS *** HHHHTT AAAa

1of8A 60 65 4.830 -75.10 -51.10 -68.30 -23.10 -96.10 9.50 79.50 8.90 IDIITGKD *** HHHHTTS AAAa

1of8A 302 307 5.386 -57.00 -51.20 -59.60 -31.10 -91.70 5.90 115.80 11.40 EQIANGEN *** HHHHTT AAAa

1ogqA 15 20 5.133 -62.70 -40.60 -66.20 -13.10 -107.80 12.50 85.60 25.40 IKKDLGNP *** HHHHTT AAAa

1ogsA 220 225 5.380 -65.30 -34.70 -63.80 -23.60 -97.60 20.50 61.20 40.70 AYAEHKLQ *** HHHHTT AAAa

1ogsA 370 375 5.194 -60.20 -35.50 -73.50 -13.40 -118.30 43.70 72.20 -1.60 TNLLYHVV *** HHHHTTEE AADa

1ohlA 78 83 5.525 -61.10 -34.50 -69.30 -27.60 -83.70 -2.70 113.20 4.10 PLVAKGLR *** HHHHTT AAAa

1ohlA 193 198 4.975 -63.60 -46.90 -65.60 -26.70 -91.60 2.60 60.00 43.90 GLINANLA *** HHHHTT T AAAa

1ohlA 252 257 5.780 -61.00 -49.20 -63.10 -27.00 -83.20 0.10 113.20 1.30 RDMSEGAD *** HHHHTT S AAAa

1ohlA 299 304 5.100 -63.90 -48.40 -59.40 -37.70 -73.30 -7.80 77.10 13.60 AAAEKGVV *** HHHHTTSS AAAa

1ohlA 318 323 5.517 -63.00 -38.80 -64.60 -39.60 -72.60 -6.00 116.10 -9.50 GFLRAGAR *** HHHHTT S AAAa

1oi2A 130 135 5.286 -57.00 -42.20 -67.70 -22.80 -91.50 5.60 90.60 6.70 LLHDSGVK *** HHHHTT AAAa

1oi7A 84 89 5.729 -64.30 -41.20 -66.70 -22.10 -84.90 -6.50 91.40 6.10 EAAHAGIP *** HHHHTT S AAAa

1oi7A 164 169 5.255 -67.00 -45.20 -64.20 -29.10 -83.00 -6.10 85.60 3.30 ALSQAGLG *** HHHHTT AAAa

1ojjA 315 320 5.284 -65.40 -44.10 -66.20 -28.20 -94.90 11.80 85.20 19.20 FiEATGSR *** HHHHTT H AAAa

1ojjA 323 328 5.544 -70.70 -43.90 -64.50 -30.20 -101.30 10.40 149.00 -18.70 KYMELGAT *** HHHHTTHH AAAx

1ojrA 129 134 4.273 -66.80 -45.00 -70.00 -24.10 -120.10 16.80 50.10 45.60 RIKATNGK *** HHHHTTT AAAa

1ojrA 241 246 5.170 -63.60 -36.90 -67.20 -20.20 -98.10 12.00 104.60 -0.10 KVYSMGGM *** HHHHTT AAAa

1ojxA 25 30 6.294 -54.40 -39.10 -89.30 -44.10 -64.50 -41.60 -120.70 -20.70 DHGIEHGP *** HHHHT G AAAA

1ojxA 101 106 5.530 -58.80 -47.50 -58.90 -31.10 -85.10 -2.10 95.90 17.50 EAVSLGAS *** HHHHTT S AAAa

1ojxA 220 225 5.809 -70.30 -40.20 -57.80 -33.40 -80.30 -8.30 94.60 16.90 GVLEAGAL *** HHHHTT AAAa

1on3A 39 44 5.533 -59.00 -40.00 -64.60 -21.00 -91.30 9.60 73.40 27.60 KQHSQGKQ *** HHHHTT AAAa

1on3A 358 363 4.771 -62.30 -43.20 -59.10 -34.50 -110.00 26.40 59.20 35.70 FCDSFNIP *** HHHHTT AAAa

1on3A 380 385 5.620 -72.10 -44.00 -68.90 -22.30 -84.00 -5.80 85.20 46.40 QQEYGGII *** HHHHTTHH AAAa

1on3A 481 486 5.189 -66.10 -39.20 -57.10 -40.20 -86.50 -4.20 83.40 8.70 VAAARGQV *** HHHHTTSS AAAa

1oneA 362 367 5.189 -59.90 -38.90 -59.30 -31.00 -86.70 7.40 76.10 14.00 DSFAAGWG *** HHHHTT E AAAa

1oneA 387 392 5.866 -75.40 -46.80 -53.30 -49.00 -69.60 -12.60 59.20 29.00 LVVGLRTG *** HHHHTT S AAAa

1onwA 92 97 5.120 -62.90 -42.10 -63.10 -21.50 -88.80 -4.40 88.90 11.80 RLTEAGVT *** HHHHTTEE AAAa

1onwA 244 249 5.561 -66.00 -40.60 -61.30 -31.30 -76.20 -7.10 122.70 -8.60 EFARKGGT *** HHHHTT AAAa

1onwA 270 275 5.427 -64.30 -42.00 -57.00 -34.90 -78.60 0.80 82.70 15.60 RAVQAGIP *** HHHHTT AAAa

1onwA 337 342 4.598 -73.00 -42.20 -63.50 -39.20 -95.90 6.90 50.20 43.40 VAGFLNLT *** HHHHTT T AAAa

1oohA 30 35 5.031 -62.70 -34.50 -59.80 -30.00 -101.90 14.20 77.90 19.60 DRLRVGDF *** HHHHTT AAAa

1ooyA 63 68 5.235 -76.10 -43.30 -57.40 -40.60 -83.70 3.10 59.10 38.70 LLLQSKQI *** HHHHTT E AAAa

1ooyA 108 113 4.739 -59.80 -22.00 -108.00 -2.10 -109.10 9.20 72.70 19.10 RAGGAGVP *** HHHHTT AAAa

1ooyA 349 354 5.524 -75.50 -49.10 -64.70 -29.50 -75.90 1.00 77.70 16.90 AMIRGGHV *** HHHHTT S AAAa

1oqvA 73 78 5.540 -59.50 -42.70 -68.00 -20.90 -88.10 -2.80 73.60 16.00 GLVSLGKI *** HHHHTTSS AAAa

1or7A 13 18 5.320 -59.70 -38.50 -65.30 -22.70 -89.70 -4.40 112.50 4.40 ERVQKGDQ *** HHHHTT H AAAa

1or7A 160 165 5.065 -68.80 -39.80 -65.60 -41.00 -95.20 5.80 44.30 61.50 IAAIMDCP *** HHHHTTS AAAa

1or7C 8 13 5.218 -58.20 -46.50 -67.20 -13.30 -98.40 1.90 75.20 14.60 SALMDGET *** HHHHTTS AAAa

1orc_ 33 38 5.766 -66.40 -47.70 -51.60 -46.40 -72.10 -6.20 61.70 40.00 KAIHAGRK *** HHHHTT E AAAa

1otkA 180 185 5.384 -73.10 -32.30 -67.50 -28.30 -85.60 1.90 73.50 13.90 ALSEEGIA *** HHHHTTSS AAAa

1otkA 204 209 5.246 -73.80 -35.90 -64.40 -30.50 -83.80 10.60 58.10 38.40 GINEATLN *** HHHHTT AAAa

1ow4A 29 34 5.224 -70.10 -45.80 -59.70 -37.50 -81.40 -2.90 58.80 38.10 KTVLNRNP *** HHHHTT AAAa

1owlA 88 93 5.080 -63.80 -44.60 -64.00 -22.70 -99.60 -2.90 51.50 40.60 LAQQLQAE *** HHHHTT S AAAa

1owlA 117 122 5.004 -59.30 -44.90 -58.90 -30.60 -88.10 2.60 71.00 27.20 ALKTAGIR *** HHHHTT E AAAa

1owlA 220 225 5.594 -70.30 -14.90 -86.40 -23.90 -139.10 -79.10 -47.00 -38.30 EFCDRAIA *** HHHHTGGG AAAA

1owlA 246 251 4.614 -79.90 -36.80 -65.60 -40.00 -99.80 4.50 63.70 37.20 PALKFGAI *** HHHHTTSS AAAa

1owlA 323 328 4.810 -60.80 -45.80 -65.60 -27.80 -98.40 12.70 54.10 46.80 TAWTQAQT *** HHHHTT S AAAa

1ox0A 86 91 5.335 -59.00 -49.40 -57.30 -41.70 -76.50 -8.80 50.10 42.80 AVNHANLD *** HHHHTT AAAa

1ox0A 177 182 5.218 -72.00 -43.30 -54.40 -40.50 -95.00 11.60 88.00 8.90 RSIKFGFQ *** HHHHTTS AAAa

1ox0A 203 208 5.480 -63.20 -41.10 -66.30 -29.70 -76.60 -5.90 58.10 37.50 GFQALTAL *** HHHHTT B AAAa

1ox0A 246 251 5.197 -72.90 -35.00 -63.60 -34.90 -92.10 -2.10 76.60 29.50 HAEKRGAT *** HHHHTT AAAa

1oxxK 125 130 5.049 -64.90 -44.30 -60.10 -37.10 -95.10 10.00 53.10 46.40 VAKILDIH *** HHHHTT G AAAa

1oygA 148 153 7.348 -82.40 -43.50 -63.20 -23.40 -67.00 -12.40 52.40 55.70 DKFDANDS *** HHHHTT T AAAa

1p0hA 100 105 4.477 -65.70 -39.90 -64.90 -40.70 -96.60 17.10 57.70 30.90 ALAKTAGR *** HHHHTTT AAAa

1p0hA 122 127 5.016 -63.00 -39.80 -67.20 -30.40 -89.00 2.50 73.90 22.40 TASALGLV *** HHHHTT E AAAa

1p0hA 294 299 5.482 -61.80 -34.10 -68.10 -15.60 -107.40 15.00 86.00 5.40 TYQSLGFT *** HHHHTT E AAAa

1p0zA 32 37 5.259 -71.40 -41.40 -59.30 -39.30 -84.50 -8.80 55.90 41.50 EAVQKRDL *** HHHHTT H AAAa

1p1jA 87 92 4.691 -73.00 -43.20 -59.20 -41.80 -102.90 16.20 44.90 54.50 LANKHNVE *** HHHHTT AAAa

1p1jA 231 236 5.111 -68.40 -33.00 -67.40 -17.10 -118.10 12.00 47.40 47.00 FKEENALD *** HHHHTT S AAAa

1p1jA 267 272 5.178 -65.70 -46.10 -62.70 -27.00 -90.10 4.90 56.60 34.60 QSIKNDHE *** HHHHTT T AAAa

1p1jA 285 290 4.954 -59.10 -45.00 -66.90 -25.30 -90.20 -3.50 74.50 24.00 ASILEGVP *** HHHHTT AAAa

1p1jA 336 341 5.102 -67.50 -38.30 -63.60 -26.20 -87.90 -3.30 86.00 14.30 FLVDAGIK *** HHHHTT E AAAa

1p1jA 515 520 4.844 -64.40 -30.50 -65.70 -27.10 -104.20 20.20 81.60 16.40 LRLLIGLP *** HHHHTT AAAa

1p1xA 60 65 5.292 -66.40 -43.70 -67.50 -27.80 -89.20 9.80 78.00 24.50 TLKEQGTP *** HHHHTT T AAAa

1p1xA 109 114 5.394 -60.10 -44.00 -60.70 -29.70 -88.80 10.40 96.80 4.30 RALMAGNE *** HHHHTT AAAa

1p1xA 129 134 4.743 -61.70 -34.80 -66.50 -25.80 -100.70 6.50 57.20 43.10 ACAAANVL *** HHHHTT E AAAa

1p1xA 158 163 5.450 -63.30 -45.10 -60.10 -35.70 -80.80 -6.40 111.70 12.00 ISIKAGAD *** HHHHTT S AAAa

1p3dA 59 64 5.341 -61.90 -42.20 -63.90 -30.10 -83.00 -6.10 113.80 0.70 RLAQAGAK *** HHHHTT E AAAa

1p3dA 97 102 5.234 -68.60 -38.60 -62.60 -38.70 -87.80 1.70 59.60 31.70 TSKQKRIP *** HHHHTT AAAa

1p3dA 140 145 5.434 -63.30 -47.20 -61.30 -36.30 -72.40 1.00 63.50 33.60 IYTQAKLD *** HHHHTT AAAa

1p3dA 303 308 5.233 -63.20 -40.90 -68.80 -22.00 -85.60 -12.00 91.50 13.40 VAKEEGIA *** HHHHTT AAAa

1p42A 84 89 5.124 -68.50 -46.90 -61.10 -33.50 -94.50 0.70 54.70 41.70 VLHLLEIT *** HHHHTT AAAa

1p42A 201 206 5.250 -68.20 -36.50 -64.80 -34.60 -88.30 16.90 78.70 18.20 HIKKVGLG *** HHHHTT AAAa

1p42A 275 280 5.904 -66.60 -41.80 -57.50 -49.00 -66.80 9.90 37.20 35.20 ELAKKQK# *** HHHHTT AAAa

1p4cA 145 150 5.334 -62.10 -48.90 -70.00 -20.20 -98.00 6.90 98.50 5.90 KALHTGYT *** HHHHTT AAAa

1p4cA 242 247 5.375 -66.50 -42.10 -63.00 -30.20 -85.30 0.10 117.10 -11.80 RCIAEGAD *** HHHHTT S AAAa

1p4cA 296 301 5.518 -62.70 -39.60 -63.80 -33.20 -79.50 -1.80 114.20 -4.80 KALALGAE *** HHHHTT S AAAa

1p57A 45 50 5.103 -53.30 -52.30 -48.50 -44.60 -80.70 -5.80 97.10 14.10 SbEEMGFL *** HHHHTT S AAAa

1p5dX 43 48 5.148 -63.20 -35.60 -66.60 -25.00 -96.00 10.80 81.20 20.00 ESLARGEP *** HHHHTT AAAa

1p5dX 230 235 4.953 -65.40 -46.70 -62.00 -36.90 -98.00 6.50 44.10 50.90 KVKAENAD *** HHHHTT S AAAa

1p5dX 294 299 5.062 -61.50 -39.30 -62.30 -35.40 -85.60 1.90 86.20 16.40 LISGYGGR *** HHHHTT E AAAa

1p5fA 26 31 5.383 -66.40 -36.00 -58.30 -33.60 -80.90 1.80 81.90 12.70 VMRRAGIK *** HHHHTT E AAAa

1p5fA 95 100 5.018 -63.20 -44.30 -70.20 -24.60 -95.50 6.00 63.80 42.00 EQENRKGL *** HHHHTT E AAAa

1p5fA 112 117 4.870 -59.80 -47.90 -56.90 -39.30 -98.70 8.90 57.60 46.30 ALLAHEIG *** HHHHTT S AAAa

1p5xA 100 105 5.193 -58.90 -44.20 -66.20 -26.50 -93.20 12.40 63.80 16.40 ESYKNKDM *** HHHHTT H AAAa

1p5zB 141 146 5.094 -57.80 -48.20 -62.30 -30.00 -86.10 7.20 65.60 26.80 NLYESECM *** HHHHTTSS AAAa

1p7tA 107 112 6.641 -60.80 -23.20 -105.20 -36.50 -79.10 -28.80 -133.10 17.90 DSEITSQA *** HHHHT AAAA

1p7tA 410 415 4.650 -59.20 -52.80 -56.00 -33.10 -100.90 11.20 62.80 28.60 IETMLGMA *** HHHHTT AAAa

1p7tA 493 498 5.548 -63.80 -46.30 -57.20 -41.60 -81.10 8.30 83.50 21.80 SGLFCGLR *** HHHHTT T AAAa

1p7tA 525 530 5.890 -71.20 -40.30 -64.30 -26.70 -76.00 -2.60 102.30 9.90 DQLRAGAN *** HHHHTT S AAAa

1p7tA 645 650 5.063 -63.10 -59.10 -43.20 -36.80 -96.00 11.20 73.30 9.40 NWLRHGIL *** HHHHTTSS AAAa

1p90A 182 187 5.608 -63.40 -42.60 -57.00 -26.40 -82.10 -2.90 83.00 17.60 KVVRAGIH *** HHHHTT E AAAa

1p99A 81 86 4.975 -71.90 -41.80 -65.70 -30.90 -90.00 1.20 79.30 7.20 KALNDGDI *** HHHHTTSS AAAa

1p99A 156 161 5.377 -67.70 -42.60 -62.90 -29.60 -82.00 -2.90 77.70 18.80 LLEAAGLI *** HHHHTTS AAAa

1p99A 211 216 5.221 -68.40 -28.20 -63.20 -34.90 -85.60 10.10 75.40 22.00 VATKAGKD *** HHHHTT AAAa

1pa1A 698 703 6.410 -56.50 -48.70 -64.60 -25.90 -67.80 -15.50 84.90 2.30 KVRESGSL *** HHHHTTTT AAAa

1pa1A 779 784 5.580 -71.70 -47.70 -52.20 -43.80 -85.40 15.90 108.80 1.90 AKFIMGDS *** HHHHTT T AAAa

1pb7A 212 217 4.933 -66.30 -43.40 -61.30 -36.00 -89.50 8.00 64.50 27.30 QAVRDNKL *** HHHHTTS AAAa

1pbe_ 21 26 5.152 -61.90 -53.90 -56.00 -28.00 -89.00 -2.10 87.80 14.20 LLHKAGID *** HHHHTT AAAa

1pbe_ 56 61 5.338 -60.10 -49.70 -60.50 -33.10 -82.80 -0.30 65.70 30.60 LLREAGVD *** HHHHTT AAAa

1pbe_ 89 94 4.374 -74.50 -35.20 -76.80 -29.50 -91.20 3.80 80.10 34.00 LKRLSGGK *** HHHHTTS AAAa

1pfvA 38 43 6.015 -65.90 -37.60 -63.50 -27.00 -89.70 10.40 82.10 13.00 YQRMRGHE *** HHHHTT E AAAa

1pfvA 84 89 5.540 -65.20 -34.90 -65.70 -24.20 -101.00 7.70 57.20 44.30 DFAGFNIS *** HHHHTT AAAa

1pfvA 113 118 5.194 -68.60 -43.00 -63.50 -31.80 -87.10 5.70 76.20 23.80 RLKENGFI *** HHHHTT E AAAa

1pfvA 309 314 4.859 -56.20 -47.60 -69.80 -15.20 -113.20 17.80 64.70 36.90 MLEGSNFR *** HHHHTTB AAAa

1pfvA 431 436 4.911 -63.30 -47.40 -55.20 -45.50 -83.00 3.00 68.90 18.30 EAWESREF *** HHHHTT H AAAa

1pg4A 290 295 4.920 -76.00 -36.90 -75.30 -34.50 -96.40 0.90 51.30 47.70 FKYVFDYH *** HHHHTT AAAa

1pg4A 320 325 5.212 -64.80 -36.70 -58.60 -35.20 -87.30 -0.20 75.60 21.80 GPLACGAT *** HHHHTT E AAAa

1pjcA 186 191 5.579 -66.50 -40.10 -61.30 -35.10 -79.70 -7.10 97.60 7.10 MAVGLGAQ *** HHHHTT E AAAa

1pkhA 11 16 4.929 -70.80 -41.00 -57.80 -42.80 -88.90 3.90 65.80 23.40 DYVTSKRI *** HHHHTTSS AAAa

1pmi_ 77 82 6.310 -68.10 -28.30 -83.00 -40.50 -91.80 -33.60 -104.10 -49.20 KPQEYLGE *** HHHHT H AAAA

1pmmA 105 110 5.135 -62.70 -45.20 -57.50 -33.80 -95.60 15.90 60.80 31.40 VADLWHAP *** HHHHTT AAAa

1pmmA 170 175 4.584 -66.90 -46.80 -70.50 -29.30 -111.10 20.00 46.20 55.50 FARYWDVE *** HHHHTT E AAAa

1pn2A 18 23 5.441 -60.10 -43.60 -58.50 -27.50 -90.80 10.60 99.10 7.50 YNIALGAT *** HHHHTT AAAa

1pn2A 193 198 5.335 -68.10 -35.20 -55.90 -38.60 -87.10 13.70 70.80 31.30 FAKGAKFP *** HHHHTT S AAAa

1pot_ 177 182 5.227 -75.70 -44.30 -49.90 -33.10 -87.90 -7.30 90.90 15.60 ALRKLGYS *** HHHHTT AAAa

1pot_ 217 222 5.661 -67.70 -43.30 -67.40 -39.40 -72.80 -16.20 59.60 78.00 NPYMEGEV *** HHHHTTS AAAa

1prxA 87 92 5.225 -63.60 -35.00 -62.10 -35.20 -86.70 5.30 60.50 33.10 INAYNSEE *** HHHHTTS AAAa

1prxA 111 116 5.431 -70.50 -43.90 -60.40 -39.40 -83.20 -1.20 72.10 32.50 LAILLGML *** HHHHTTSS AAAa

1psrA 46 51 5.062 -56.40 -39.10 -57.80 -31.50 -94.50 4.60 88.50 10.80 AaDKKGTN *** HHHHTT AAAa

1pszA 104 109 5.338 -61.00 -52.80 -59.20 -34.50 -86.30 0.80 55.50 51.30 LVENAKKT *** HHHHTT AAAa

1pu6A 55 60 5.224 -73.10 -38.10 -69.50 -23.50 -92.50 -0.60 60.60 28.40 NLKNAFIL *** HHHHTTSS AAAa

1pu6A 157 162 5.433 -58.70 -39.70 -65.30 -30.30 -83.30 -2.20 96.10 18.10 FLKKLGIE *** HHHHTT AAAa

1puc_ 71 76 5.977 -68.20 -34.70 -64.60 -20.90 -81.90 -8.40 104.80 3.90 EWRGLGIT *** HHHHTT AAAa

1pwgA 18 23 5.515 -61.40 -43.00 -63.70 -20.70 -97.30 -3.00 123.00 1.20 TALSQGAP *** HHHHTT S AAAa

1pwgA 75 80 5.264 -62.40 -46.10 -57.30 -35.30 -82.50 -2.00 82.40 12.20 QLVDEGKL *** HHHHTTS AAAa

1pwgA 104 109 4.810 -59.00 -49.20 -71.10 -17.00 -107.90 11.60 58.70 40.40 RQVMSHRS *** HHHHTT S AAAa

1pwgA 253 258 5.233 -59.80 -56.30 -69.00 -28.40 -84.70 1.50 93.10 21.90 SALMSGQL *** HHHHTT S AAAa

1px0A 21 26 5.753 -72.10 -41.00 -61.70 -31.20 -72.90 -7.50 100.70 0.40 RLSEAGHT *** HHHHTT E AAAa

1px0A 223 228 6.495 -67.00 -33.40 -77.80 -17.50 -76.70 -11.40 77.50 14.70 AFLASGSC *** HHHHTTS AAAa

1pz4A 73 78 5.196 -72.60 -43.50 -63.40 -22.90 -95.50 -0.60 81.60 11.30 FAIGTGAL *** HHHHTTSS AAAa

1pz4A 84 89 5.014 -67.60 -41.40 -65.30 -33.90 -89.70 12.30 59.80 34.80 EAMAQDKM *** HHHHTTSE AAAa

1pztA 206 211 6.302 -68.00 -42.90 -64.00 -29.30 -80.90 4.10 51.20 50.60 ILQRQQLD *** HHHHTT E AAAa

1pztA 301 306 5.261 -63.20 -33.80 -59.30 -33.90 -99.50 13.30 59.60 37.40 QFLSINGF *** HHHHTTS AAAa

1pztA 325 330 5.239 -63.90 -38.00 -68.70 -22.10 -99.80 19.90 71.70 26.60 RLAFRGMS *** HHHHTT AAAa

1pzxA 18 23 4.989 -63.50 -43.90 -56.70 -47.40 -92.80 15.90 45.50 58.40 YIREHRIA *** HHHHTT E AAAa

1pzxA 75 80 5.064 -63.00 -41.00 -67.90 -48.90 -78.20 -0.60 52.30 51.20 PYAKENRP *** HHHHTT AAAa

1pzxA 136 141 5.438 -66.50 -38.60 -74.70 -14.90 -91.00 1.90 56.70 49.70 ELAKQNTP *** HHHHTT AAAa

1q08A 54 59 5.944 -68.00 -30.50 -67.50 -25.80 -88.90 -0.20 81.30 12.80 HARQLGFS *** HHHHTT AAAa

1q0rA 88 93 4.924 -53.70 -44.50 -68.30 -19.10 -107.60 8.50 67.00 27.80 VLDGWGVD *** HHHHTT S AAAa

1q0rA 206 211 5.009 -56.30 -43.40 -61.60 -32.80 -86.40 23.60 69.00 24.90 AIDHAGGV *** HHHHTTT AAAa

1q2oA 137 142 5.016 -68.70 -40.60 -63.10 -31.00 -98.30 8.80 61.00 40.80 YYSSIKRS *** HHHHTT T AAAa

1q2oA 276 281 5.370 -66.40 -40.80 -65.60 -23.00 -99.00 19.00 101.90 0.70 LCIQHGWT *** HHHHTT AAAa

1q2oA 382 387 5.401 -64.90 -41.50 -65.10 -28.00 -85.90 -3.90 59.60 41.50 VAVCMDLD *** HHHHTT AAAa

1q2oA 412 417 5.025 -64.00 -42.10 -64.90 -36.30 -83.20 -1.50 59.30 45.00 SFQLAKVT *** HHHHTT AAAa

1q35A 66 71 5.548 -67.30 -43.00 -64.40 -28.40 -83.20 7.60 60.50 37.20 EIVNADLA *** HHHHTT B AAAa

1q6oA 76 81 5.702 -74.00 -30.00 -67.50 -24.80 -86.00 7.40 81.00 23.40 MCFEANAD *** HHHHTT S AAAa

1q6oA 102 107 4.605 -64.40 -33.80 -59.60 -29.90 -103.70 10.50 56.40 35.50 VAKEFNGD *** HHHHTT E AAAa

1q6oA 125 130 5.362 -61.30 -46.00 -61.50 -30.00 -79.30 -7.50 94.20 11.00 QWRDAGIG *** HHHHTT AAAa

1q6oA 141 146 5.438 -51.00 -46.00 -67.60 -26.40 -85.30 7.50 98.90 8.50 DAQAAGVA *** HHHHTT AAAa

1q6oA 159 164 5.874 -60.30 -42.70 -65.40 -25.60 -74.30 -11.30 105.50 4.40 RLSDMGFK *** HHHHTT E AAAa

1q6zA 12 17 5.080 -61.80 -41.50 -67.10 -23.80 -96.40 13.10 86.80 8.60 LLRRQGID *** HHHHTT AAAa

1q6zA 85 90 4.974 -60.50 -47.90 -70.00 -26.70 -92.20 3.30 55.80 41.80 NAWNSHSP *** HHHHTT AAAa

1q6zA 211 216 5.045 -64.00 -38.90 -57.80 -39.80 -80.60 -5.00 53.70 41.00 DVDAANAN *** HHHHTT H AAAa

1q74A 28 33 5.309 -65.40 -38.90 -64.50 -22.80 -94.20 8.80 83.50 13.30 HYTSRGAQ *** HHHHTT E AAAa

1q74A 76 81 4.954 -61.70 -38.70 -62.50 -30.00 -90.30 -0.70 89.10 11.90 ALRALGVS *** HHHHTT AAAa

1q7eA 28 33 5.596 -65.70 -42.20 -65.80 -24.20 -86.40 0.50 111.70 -3.40 XLAWFGAD *** HHHHTT E AAAa

1q7eA 146 151 5.175 -70.70 -45.40 -73.90 -17.50 -101.60 -1.50 108.20 -17.40 AQAAGGAA *** HHHHTTHH AAAa

1q7eA 283 288 4.838 -64.00 -44.20 -53.80 -44.90 -98.50 10.00 69.70 34.80 TCKAIGKP *** HHHHTT G AAAa

1q7eA 352 357 4.993 -69.30 -44.00 -63.80 -37.00 -78.50 -10.80 79.80 16.60 SLRQSGSV *** HHHHTTSE AAAa

1q7eA 399 404 5.604 -60.70 -44.90 -62.10 -28.10 -81.10 -5.20 98.10 7.60 VLQELGYS *** HHHHTT AAAa

1q7lA 128 133 5.444 -71.30 -45.90 -70.60 -24.00 -84.70 1.30 92.20 18.80 RLKVEGHR *** HHHHTT AAAa

1q7lB 331 336 5.070 -66.40 -43.60 -55.60 -39.70 -89.00 -0.40 64.70 37.00 VCKDMNLT *** HHHHTT AAAa

1q7lB 352 357 5.680 -66.90 -41.30 -62.80 -25.20 -95.00 12.70 98.00 8.40 YIRAVGVP *** HHHHTT AAAa

1q8fA 54 59 4.839 -62.00 -48.30 -62.40 -41.40 -89.60 5.10 52.90 43.00 VCQKLEIN *** HHHHTT AAAa

1q8iA 96 101 5.078 -59.50 -44.50 -59.40 -34.50 -83.40 3.60 88.20 14.80 RLREGGVT *** HHHHTT AAAa

1q8iA 115 120 4.888 -48.80 -53.50 -61.60 -16.10 -105.80 11.50 62.30 29.10 YLMERFIT *** HHHHTT S AAAa

1q8iA 237 242 4.941 -74.70 -33.80 -66.40 -40.70 -90.50 2.40 58.70 34.20 HAERYRLP *** HHHHTT AAAa

1q8iA 381 386 5.636 -64.50 -44.80 -59.10 -31.10 -78.10 -10.40 106.40 13.90 RMHRAGYV *** HHHHTTB AAAa

1q8iA 480 485 5.007 -58.80 -48.70 -57.10 -38.50 -85.30 0.30 76.20 19.90 EAKRQGNK *** HHHHTT H AAAa

1q8iA 534 539 6.172 -63.30 -36.60 -62.60 -26.00 -83.70 -1.40 101.00 7.60 LIEAQGYD *** HHHHTT E AAAa

1q8iA 655 660 4.937 -62.70 -39.30 -60.40 -36.30 -90.80 7.20 59.50 29.70 LRIFRNEP *** HHHHTT AAAa

1q8iA 672 677 5.377 -59.90 -53.50 -67.60 -18.80 -87.30 -6.80 81.80 20.90 DKLMAGEL *** HHHHTTTT AAAa

1q9uA 23 28 4.928 -57.40 -40.90 -62.40 -22.00 -103.10 10.50 82.50 12.60 SLKQEGFG *** HHHHTT E AAAa

1q9uA 40 45 5.170 -67.60 -31.40 -63.40 -36.00 -82.20 -0.80 81.90 17.40 KLQEKGLD *** HHHHTT AAAa

1qazA 120 125 5.334 -71.10 -44.80 -63.40 -26.70 -93.00 7.60 52.60 38.50 KWAKADAL *** HHHHTTTT AAAa

1qazA 204 209 4.790 -69.00 -52.20 -70.10 -22.00 -104.10 3.00 53.80 52.80 IGVISKDD *** HHHHTT H AAAa

1qb7A 162 167 5.311 -62.20 -46.60 -60.90 -26.70 -81.60 1.20 67.10 33.10 LVEASDAV *** HHHHTT E AAAa

1qd1A 257 262 4.900 -63.00 -33.70 -61.40 -23.50 -111.10 13.00 56.80 40.50 EAQELSLP *** HHHHTT AAAa

1qddA 66 71 7.115 -59.50 -41.80 -65.40 -20.20 -69.30 -22.90 81.30 6.60 LIKESGTD *** HHHHTT AAAa

1qf5A 139 144 5.289 -65.90 -57.50 -60.30 -34.90 -75.60 -7.40 62.30 28.20 EDKVARRG *** HHHHTT AAAa

1qf8A 51 56 4.742 -62.00 -45.20 -61.50 -36.00 -94.90 11.40 58.00 34.90 LDXILDLE *** HHHHTT AAAa

1qf8A 100 105 5.394 -62.50 -43.50 -67.10 -23.80 -92.30 -5.90 74.50 23.70 EKYQQGDF *** HHHHTTTT AAAa

1qfmA 537 542 5.147 -75.30 -49.40 -63.90 -34.90 -81.80 -1.00 84.50 13.30 YLIKEGYT *** HHHHTTS AAAa

1qgiA 86 91 5.633 -66.40 -45.70 -60.70 -28.40 -84.70 1.40 99.40 7.20 YDAAKGAS *** HHHHTT S AAAa

1qgiA 100 105 5.061 -62.70 -48.40 -55.70 -38.50 -83.90 -5.10 66.50 32.70 ALKRLGIN *** HHHHTT AAAa

1qgiA 156 161 4.838 -62.20 -36.60 -57.00 -34.80 -93.00 3.70 78.40 20.20 QARQRGFT *** HHHHTT AAAa

1qgiA 235 240 5.220 -65.00 -48.70 -59.70 -39.40 -78.20 -4.90 69.90 20.90 TLVDMGKM *** HHHHTT T AAAa

1qhdA 141 146 5.139 -55.30 -47.10 -59.30 -33.00 -91.10 15.80 56.20 46.40 NLQNRRQR *** HHHHTT AAAa

1qhdA 350 355 4.968 -66.60 -46.10 -60.10 -40.80 -94.50 18.20 48.50 44.30 VRQEYAIP *** HHHHTT AAAa

1qj4A 26 31 5.679 -63.30 -34.80 -70.10 -12.80 -90.90 -7.00 104.90 1.40 LLEALGHK *** HHHHTT E AAAa

1qlmA 54 59 5.435 -60.10 -46.30 -55.90 -34.40 -93.30 17.20 72.80 21.60 SEVCMGGL *** HHHHTTT AAAa

1qlmA 269 274 5.013 -63.90 -41.20 -71.80 -15.60 -97.00 21.10 56.60 35.30 IFEEADYD *** HHHHTTT AAAa

1qlmA 311 316 4.738 -59.80 -45.60 -60.80 -42.30 -94.50 4.10 55.80 52.90 LKESFSL# *** HHHHTT AAAa

1qlwA 92 97 5.480 -59.70 -44.20 -66.10 -25.90 -85.30 0.40 96.80 8.70 YFLRKGYS *** HHHHTT AAAa

1qlwA 119 124 5.232 -68.50 -43.20 -59.30 -36.20 -78.00 -4.50 76.30 18.50 NAVKLGKA *** HHHHTTSS AAAa

1qlwA 142 147 5.405 -79.40 -41.10 -57.70 -38.40 -86.10 -6.70 63.70 44.10 AWAIFRFG *** HHHHTTSS AAAa

1qlwA 276 281 5.108 -64.50 -39.10 -65.60 -27.10 -87.20 3.70 96.80 5.80 ALNAAGGK *** HHHHTT AAAa

1qmgA 148 153 5.081 -65.30 -45.50 -62.30 -35.40 -77.30 -6.90 62.70 36.80 SLTEAKSD *** HHHHTT AAAa

1qmgA 171 176 5.353 -64.00 -38.60 -62.10 -33.00 -80.50 -3.30 101.10 10.20 EARAAGFS *** HHHHTT AAAa

1qmgA 338 343 5.442 -68.60 -31.80 -64.60 -35.90 -83.50 9.90 102.70 -12.60 RYTESGMS *** HHHHTT AAAa

1qmgA 543 548 5.178 -59.20 -41.80 -76.50 -4.70 -110.10 13.80 61.10 37.00 VAVDNGAP *** HHHHTT AAAa

1qnaA 101 106 6.084 -59.90 -49.20 -61.00 -15.10 -90.10 3.80 99.70 11.60 IVQKLGFP *** HHHHTT AAAa

1qnrA 45 50 5.704 -67.00 -44.70 -68.30 -20.40 -74.40 -12.50 96.90 4.80 HISSSGLK *** HHHHTT AAAa

1qnrA 244 249 4.795 -63.10 -29.50 -91.90 -19.40 -103.80 -1.60 81.60 19.70 YPDSWGTN *** HHHHT AAAa

1qnrA 265 270 5.261 -65.90 -34.80 -63.20 -32.50 -85.00 2.10 80.20 17.40 AcLAAGKP *** HHHHTTS AAAa

1qo2A 39 44 5.650 -62.10 -41.30 -66.30 -36.10 -75.80 3.20 97.40 -1.50 KLIEEGFT *** HHHHTT AAAa

1qo2A 91 96 5.517 -64.60 -35.20 -62.60 -31.00 -80.10 -2.30 101.80 -3.00 KLRKLGYR *** HHHHTT AAAa

1qo2A 210 215 5.186 -76.90 -44.70 -61.20 -25.10 -99.90 8.80 51.60 40.60 VHTETNGL *** HHHHTTTS AAAa

1qo2A 225 230 4.728 -74.10 -54.30 -66.00 -28.70 -88.70 -3.50 72.90 9.90 RAFLEGIL *** HHHHTTSS AAAa

1qopA 11 16 4.928 -65.30 -35.20 -65.70 -25.10 -102.90 7.50 50.50 47.50 QLNDRREG *** HHHHTT AAAa

1qopA 40 45 5.667 -64.10 -49.10 -60.60 -29.80 -80.80 -8.70 124.80 4.10 TLIDAGAD *** HHHHTT S AAAa

1qopA 71 76 5.453 -63.30 -37.00 -62.40 -25.60 -93.30 6.30 76.00 19.70 RAFAAGVT *** HHHHTT AAAa

1qopA 143 148 4.679 -60.90 -38.00 -64.20 -25.80 -108.80 21.70 59.90 34.40 AALRHNIA *** HHHHTT E AAAa

1qopA 200 205 5.151 -64.80 -39.10 -58.20 -39.60 -90.10 12.00 57.00 40.70 KLKEYHAA *** HHHHTT AAAa

1qopA 223 228 5.609 -62.90 -44.50 -60.50 -32.00 -73.60 -9.00 110.60 -2.80 AAVRAGAA *** HHHHTT S AAAa

1qopB 98 103 5.149 -68.30 -35.70 -60.40 -36.60 -83.60 -4.80 85.10 14.00 LAKRMGKS *** HHHHTT AAAa

1qopB 149 154 5.460 -68.70 -36.90 -59.10 -35.60 -83.70 8.90 85.90 4.50 RMRLMGAE *** HHHHTT E AAAa

1qopB 316 321 5.234 -66.00 -47.30 -56.60 -37.90 -89.20 -1.70 79.60 10.00 YLNSIGRA *** HHHHTTSS AAAa

1qoyA 197 202 5.085 -73.00 -45.60 -63.20 -32.10 -89.10 -4.80 78.40 24.50 YSIAAGVV *** HHHHTTSS AAAa

1qoyA 289 294 5.435 -64.80 -40.70 -61.70 -39.40 -89.80 31.90 83.20 6.10 YQKRHGKK *** HHHHTT AAAa

1qqfA 1196 1201 4.802 -62.00 -38.00 -55.90 -29.60 -105.50 16.40 54.90 40.20 ALALMNKL *** HHHHTT AAAa

1qqfA 1240 1245 5.477 -69.60 -44.90 -57.90 -39.60 -66.50 -12.40 59.80 29.70 ALLLLKDF *** HHHHTT T AAAa

1qsgA 28 33 5.675 -63.70 -45.60 -56.10 -34.50 -94.10 19.30 84.00 6.70 AMHREGAE *** HHHHTT E AAAa

1qsgA 52 57 5.031 -57.80 -42.50 -62.60 -20.30 -105.10 15.90 74.40 17.80 FAAQLGSD *** HHHHTT AAAa

1qtnA 274 279 5.505 -71.00 -39.10 -59.00 -31.80 -90.10 7.80 82.60 22.80 TFEELHFE *** HHHHTT E AAAa

1qtwA 21 26 5.205 -64.60 -37.00 -66.30 -22.10 -102.60 12.70 61.30 32.10 RAAEIDAT *** HHHHTT S AAAa

1qtwA 56 61 4.928 -69.90 -37.50 -60.60 -39.10 -105.50 13.60 64.80 44.40 ACEKYHYT *** HHHHTT AAAa

1qtwA 98 103 4.920 -58.50 -45.80 -62.10 -30.60 -86.40 -2.40 81.60 15.80 RCEQLGLS *** HHHHTT AAAa

1qu9A 61 66 5.399 -68.80 -32.50 -65.00 -22.30 -92.60 4.90 88.20 13.90 IVEAAGLK *** HHHHTT AAAa

1qu9A 95 100 5.099 -60.30 -40.60 -59.80 -27.30 -101.30 14.30 47.40 44.40 FFTEHNAT *** HHHHTT AAAa

1qvzA 38 43 5.190 -59.90 -40.60 -58.00 -36.60 -87.60 11.90 85.20 11.70 TFRKEGFE *** HHHHTT E AAAa

1qvzA 127 132 5.251 -69.60 -42.80 -62.20 -26.10 -85.50 5.50 92.40 6.20 EIYANGGV *** HHHHTT E AAAa

1qvzA 170 175 5.360 -67.80 -41.80 -62.50 -37.90 -80.50 -6.80 70.20 15.00 GETIXGVD *** HHHHTT H AAAa

1qvzA 179 184 4.930 -65.70 -43.70 -62.10 -33.90 -96.30 8.00 55.30 49.60 ILKAKNLA *** HHHHTT AAAa

1qvzA 191 196 4.836 -65.20 -43.40 -60.00 -38.50 -102.40 20.50 69.30 26.20 VAKKYGAK *** HHHHTT E AAAa

1qw9A 156 161 5.665 -61.20 -45.70 -61.80 -32.00 -75.80 -2.40 106.70 13.90 LRIAHGYK *** HHHHTT AAAa

1qwgA 81 86 5.016 -58.10 -43.00 -68.50 -23.20 -92.40 6.10 62.10 33.60 YAYSKGKF *** HHHHTT H AAAa

1qwgA 123 128 5.801 -59.40 -45.10 -59.20 -26.10 -92.30 6.80 94.10 6.90 RAKDNGFM *** HHHHTT E AAAa

1qwnA 152 157 5.348 -81.40 -45.50 -62.70 -34.60 -85.10 1.30 85.30 7.70 SIVKNGQL *** HHHHTTSE AAAa

1qwnA 237 242 4.714 -62.10 -41.50 -59.80 -30.40 -100.70 6.60 50.40 48.10 ELAQQRQL *** HHHHTT S AAAa

1qwnA 389 394 5.435 -59.60 -50.10 -65.10 -24.90 -80.30 0.00 80.40 20.10 QAERAGQA *** HHHHTTS AAAa

1qwoA 346 351 5.665 -66.60 -38.60 -62.60 -33.20 -76.50 -5.90 79.10 10.40 IFFALGLY *** HHHHTTTT AAAa

1qwyA 71 76 5.416 -69.00 -42.40 -59.10 -35.80 -89.40 3.10 53.80 49.10 AMFDNKEY *** HHHHTT AAAa

1qxoA 150 155 5.187 -62.80 -38.50 -64.90 -24.10 -100.60 22.50 59.50 37.10 LLAELDXE *** HHHHTT E AAAa

1qxoA 207 212 5.312 -60.70 -42.70 -74.40 -23.20 -75.60 -8.50 75.60 15.70 QIKRDGDT *** HHHHTT AAAa

1qxyA 154 159 4.719 -60.30 -40.90 -57.60 -22.20 -114.00 16.90 60.50 36.10 TARQNDLK *** HHHHTT E AAAa

1qz5A 142 147 5.610 -59.50 -37.30 -66.90 -19.50 -88.80 -0.20 84.90 17.10 SLYASGRT *** HHHHTT S AAAa

1qz5A 193 198 5.711 -64.20 -38.10 -69.80 -30.80 -77.80 -4.10 111.80 13.10 ILTERGYS *** HHHHTT AAAa

1qz9A 213 218 5.457 -68.90 -47.20 -61.80 -38.10 -72.50 -18.10 75.00 16.50 DLHQAGAD *** HHHHT S AAAa

1qzmA 511 516 5.401 -66.40 -48.00 -56.40 -29.10 -103.80 19.70 60.20 34.50 QIERNALK *** HHHHTT AAAa

1r0mA 159 164 5.984 -59.90 -43.80 -67.20 -27.10 -74.80 -13.70 99.20 8.80 RHVEQGYR *** HHHHTT S AAAa

1r0mA 286 291 5.021 -58.50 -37.10 -66.30 -22.90 -90.60 -2.40 64.10 38.20 VAQSFGAP *** HHHHTT AAAa

1r0rI 41 46 5.116 -62.20 -42.60 -59.90 -31.40 -86.40 3.30 58.50 34.80 AVVESNGT *** HHHHTTT AAAa

1r0vA 73 78 6.024 -60.00 -47.60 -73.80 -16.90 -73.90 -5.70 97.50 21.10 DLRDRGNK *** HHHHTT AAAa

1r0vA 226 231 5.951 -56.80 -44.20 -64.70 -25.10 -77.70 1.80 90.00 14.50 NLKERGFV *** HHHHTT E AAAa

1r0vA 282 287 4.959 -59.20 -43.90 -67.20 -31.70 -94.80 15.20 54.60 53.60 LAQNVRKR *** HHHHTT E AAAa

1r26A 64 69 5.266 -72.70 -40.50 -59.10 -39.00 -90.80 1.60 52.20 46.40 IVSKCRVL *** HHHHTT AAAa

1r29A 25 30 5.010 -60.30 -42.80 -63.50 -20.30 -107.70 5.70 55.60 36.90 RLRSRDIL *** HHHHTT S AAAa

1r29A 109 114 5.210 -61.90 -48.60 -60.20 -43.00 -77.90 -2.50 55.50 57.30 TAMYLQME *** HHHHTT H AAAa

1r2mA 43 48 4.879 -61.10 -34.80 -66.00 -20.40 -103.40 20.50 60.50 29.80 HbASKGSK *** HHHHTT E AAAa

1r2qA 152 157 4.714 -61.80 -42.00 -67.90 -22.80 -110.90 11.50 56.80 46.90 YADDNSLL *** HHHHTT E AAAa

1r45A 90 95 4.966 -63.40 -42.80 -54.70 -43.20 -95.30 10.70 50.80 43.80 PLRANQGN *** HHHHTTT AAAa

1r4vA 71 76 5.163 -61.40 -39.90 -65.50 -24.30 -88.10 1.20 82.60 9.00 RARWNGRD *** HHHHTT S AAAa

1r5lA 56 61 4.473 -65.10 -32.80 -66.50 -21.60 -120.50 30.50 47.90 38.80 FLRARDFD *** HHHHTTT AAAa

1r5lA 95 100 6.159 -65.20 -43.70 -65.60 -25.80 -70.90 -13.30 96.40 16.80 GLLKAGYH *** HHHHTT E AAAa

1r5rA 33 38 5.009 -59.60 -41.70 -63.70 -25.80 -87.40 5.80 81.80 16.70 DDVDKGNL *** HHHHTT AAAa

1r5rA 53 58 5.026 -62.80 -40.20 -60.10 -30.10 -106.20 22.20 59.50 28.90 LLEAFSLV *** HHHHTTSB AAAa

1r5yA 59 64 6.074 -67.00 -41.10 -61.90 -25.40 -72.50 -16.10 113.10 -1.60 TVRATGAD *** HHHHTT S AAAa

1r5yA 74 79 5.591 -68.40 -24.50 -86.80 -43.70 -154.70 115.50 -86.80 -3.80 HLMLRPGA *** HHHHTT H AABA

1r5yA 83 88 5.271 -60.70 -43.20 -59.80 -33.90 -84.20 1.70 108.10 3.70 RIAKLGGL *** HHHHTT H AAAa

1r5yA 334 339 5.117 -67.70 -41.30 -59.60 -37.80 -81.10 -6.10 76.80 18.40 HLIRAGEI *** HHHHTT H AAAa

1r5yA 364 369 5.008 -65.60 -45.60 -55.70 -33.40 -96.40 4.90 68.90 23.50 DSISEGRF *** HHHHTT H AAAa

1r5zA 232 237 5.390 -66.30 -40.40 -63.60 -25.00 -75.20 -11.90 108.20 5.60 ARLMEGDY *** HHHHTT G AAAa

1r69_ 10 15 4.998 -64.60 -42.50 -62.60 -28.80 -92.20 -8.40 74.30 39.50 KRIQLGLN *** HHHHTT AAAa

1r69_ 33 38 4.908 -74.60 -38.40 -74.90 -18.00 -102.40 11.30 64.20 24.20 EQLENGKT *** HHHHTTS AAAa

1r69_ 49 54 4.924 -66.30 -43.90 -67.60 -27.80 -93.90 -6.60 63.70 39.70 LASALGVS *** HHHHTT AAAa

1r6dA 114 119 5.156 -65.30 -44.80 -64.70 -24.40 -99.10 1.10 67.00 29.10 CAVDAGVG *** HHHHTT AAAa

1r6dA 198 203 4.818 -62.80 -42.40 -60.00 -32.80 -87.50 1.50 75.10 18.30 TNLLDGGT *** HHHHTT AAAa

1r6wA 92 97 4.961 -60.80 -38.50 -59.50 -40.40 -93.00 3.50 60.70 34.30 LAELTDTL *** HHHHTT S AAAa

1r6wA 252 257 4.855 -68.10 -37.60 -60.80 -29.30 -93.20 6.20 71.20 22.30 AAHALGLT *** HHHHTT E AAAa

1r6xA 47 52 4.617 -74.80 -50.50 -61.50 -32.10 -94.60 5.20 86.70 0.50 ELILNGGF *** HHHHBTTT AAAa

1r6xA 183 188 4.828 -61.50 -40.00 -63.10 -22.20 -107.50 4.90 62.20 33.70 EFQSRQWD *** HHHHTT AAAa

1r6xA 212 217 4.675 -62.40 -41.80 -67.40 -29.70 -97.70 7.10 50.60 51.50 AAREANAK *** HHHHTT E AAAa

1r6xA 278 283 5.515 -65.40 -41.70 -63.80 -30.10 -83.00 0.00 107.30 0.70 IRKNYGAS *** HHHHTT S AAAa

1r7aA 181 186 5.486 -63.80 -25.70 -93.90 -6.20 -110.60 26.40 63.50 26.80 QMAASHVS *** HHHHTT AAAa

1r7aA 222 227 4.786 -54.60 -48.30 -54.10 -31.30 -100.60 4.60 73.70 26.00 EGVKRGLE *** HHHHTT E AAAa

1r7aA 323 328 5.182 -69.90 -46.50 -61.90 -30.10 -96.80 8.60 54.30 39.60 IHANTHGE *** HHHHTTTH AAAa

1r7aA 351 356 5.018 -69.20 -33.40 -63.00 -24.90 -95.60 10.40 69.10 25.70 YYSALGXN *** HHHHTTT AAAa

1r7aA 380 385 5.108 -69.90 -30.20 -62.10 -27.00 -109.70 19.30 42.10 54.20 YVGALAGK *** HHHHTT AAAa

1r89A 155 160 5.017 -65.40 -43.20 -62.90 -24.80 -106.30 11.70 78.50 18.70 FLKANGIY *** HHHHTT B AAAa

1r89A 308 313 5.338 -65.00 -48.30 -57.30 -37.40 -90.40 14.40 65.40 31.40 FLERENFM *** HHHHTT AAAa

1r8sA 140 145 5.398 -65.10 -51.10 -75.20 -21.80 -91.90 -1.40 69.20 29.00 ITDKLGLH *** HHHHTTGG AAAa

1r8sE 83 88 4.985 -68.00 -47.70 -61.50 -33.70 -92.10 12.60 59.20 32.80 FLVENELL *** HHHHTTSS AAAa

1r9lA 31 36 5.587 -61.10 -33.70 -74.40 -17.20 -88.60 1.10 98.30 -3.90 ALEKLGYT *** HHHHTT E AAAa

1r9lA 73 78 5.266 -67.50 -42.30 -65.50 -29.00 -82.80 -3.00 134.10 -17.80 MYEAAGGD *** HHHHTTGG AAAa

1r9lA 150 155 4.900 -66.20 -50.40 -59.30 -36.00 -92.90 8.90 58.00 42.40 QLAAYELT *** HHHHTT T AAAa

1r9lA 176 181 5.833 -54.50 -43.00 -69.40 -20.60 -76.90 -7.30 83.20 17.00 SRYKEGKP *** HHHHTT AAAa

1ra0A 111 116 5.302 -59.90 -44.50 -60.30 -29.90 -94.10 19.10 68.20 21.50 WQIANGIQ *** HHHHTTEE AAAa

1ra0A 172 177 5.845 -68.10 -41.00 -63.60 -27.20 -81.50 -9.30 119.00 3.80 EALRLGAD *** HHHHTT S AAAa

1ra0A 301 306 4.856 -60.70 -42.60 -64.90 -28.10 -94.50 1.10 74.60 22.30 EMLESGIN *** HHHHTT AAAa

1ra0A 335 340 4.759 -70.20 -53.00 -66.00 -36.10 -84.90 -0.90 56.30 42.20 GLHVCQLM *** HHHHTT AAAa

1ra0A 358 363 5.375 -64.60 -41.20 -59.00 -36.60 -86.60 3.30 56.80 36.80 SARTLNLQ *** HHHHTT S AAAa

1ra4A 40 45 5.382 -71.70 -45.00 -56.80 -38.50 -76.70 -14.80 77.90 18.90 KAVERGIA *** HHHHTT AAAa

1ra4A 85 90 5.274 -61.60 -43.90 -58.90 -34.80 -80.70 5.40 82.70 17.70 LGKAAGLE *** HHHHTT S AAAa

1rcqA 35 40 4.888 50.60 52.90 50.10 44.00 83.10 -5.80 -86.60 -16.30 ADAYGHGA *** HHHHTT H rrgA

1rcqA 66 71 5.446 -61.40 -43.20 -59.20 -33.60 -80.40 -4.30 100.60 10.10 ELREAGIR *** HHHHTT AAAa

1rcqA 90 95 5.144 -66.50 -46.70 -65.80 -30.90 -95.00 6.40 52.90 47.50 LIVAHDFW *** HHHHTTEE AAAa

1rcqA 144 149 7.219 -61.00 -39.40 -78.80 -15.60 -70.00 -27.20 63.20 21.10 RLRASGKV *** HHHHTT E AAAa

1rcqA 333 338 4.704 -69.90 -17.50 -53.30 -40.00 -121.10 27.80 85.40 11.50 LAAQFGSI *** HHHHTT AAAa

1rdqE 79 84 5.018 -62.60 -46.80 -58.50 -38.20 -90.80 8.20 63.10 40.70 KVVKLKQI *** HHHHTT H AAAa

1rdqE 157 162 5.187 -64.70 -30.20 -65.20 -21.40 -94.40 8.80 67.10 25.70 YLHSLDLI *** HHHHTTEE AAAa

1rdqE 208 213 5.567 -68.60 -48.00 -62.90 -33.20 -91.90 8.80 61.60 36.80 PEIILSKG *** HHHHTT AAAa

1rdqE 304 309 5.088 -61.90 -48.50 -55.30 -40.20 -84.70 2.60 57.90 37.40 IAIYQRKV *** HHHHTT S AAAa

1re9A 154 159 4.781 -67.90 -44.50 -70.70 -28.80 -92.20 0.60 60.90 42.50 FMLLAGLP *** HHHHTT AAAa

1rec_ 156 161 5.414 -64.90 -41.30 -63.80 -36.10 -75.60 -2.50 64.80 42.30 IWGFFGKK *** HHHHTT AAAa

1regX 54 59 5.342 -56.70 -57.60 -57.50 -6.80 -111.10 2.80 108.00 -4.70 MLRMDGRQ *** HHHHTT AAAa

1regX 78 83 5.247 -62.50 -48.70 -55.60 -37.50 -78.20 -7.00 78.10 18.90 LLEDWGLI *** HHHHTTS AAAa

1rgzA 20 25 4.615 -65.70 -48.10 -65.00 -35.20 -104.60 8.80 54.60 49.80 LMKAQSVP *** HHHHTT S AAAa

1rh6A 24 29 5.324 -62.50 -46.60 -62.40 -28.80 -88.90 0.50 61.70 45.60 RWVRESRI *** HHHHTT E AAAa

1rhs_ 18 23 5.326 -64.70 -37.00 -60.90 -40.40 -68.60 -10.50 84.70 15.90 ESVRAGKV *** HHHHTT AAAa

1rhs_ 84 89 5.464 -61.40 -35.60 -69.70 -19.30 -95.70 10.40 79.30 25.20 YVGSLGIS *** HHHHTT AAAa

1rhs_ 115 120 5.158 -62.40 -43.30 -65.60 -21.70 -98.10 18.30 88.30 8.40 MFRVFGHR *** HHHHTT AAAa

1rhs_ 133 138 5.679 -69.30 -50.60 -56.90 -35.70 -85.80 -0.90 82.00 15.10 NWLKEGHP *** HHHHTT AAAa

1rhs_ 232 237 4.981 -61.30 -39.90 -70.60 -15.00 -95.80 1.30 63.20 34.20 MFEAKKVD *** HHHHTT AAAa

1rhs_ 260 265 5.146 -61.90 -43.20 -72.40 -12.00 -96.20 16.00 86.70 15.60 AAYLCGKP *** HHHHTT AAAa

1rjoA 27 32 5.128 -62.10 -48.50 -60.90 -33.90 -83.00 -3.70 83.40 17.10 ILRTAGLL *** HHHHTT AAAa

1rjoA 122 127 5.108 -65.90 -34.40 -63.90 -25.30 -105.20 6.40 57.70 40.30 ALAARNLD *** HHHHTT AAAa

1rk6A 323 328 5.004 -59.10 -40.30 -68.70 -21.90 -93.70 4.70 73.20 24.30 IAAERGKS *** HHHHTTS AAAa

1rkqA 33 38 5.386 -72.40 -29.50 -65.50 -26.20 -84.00 -4.10 94.00 6.20 AARARGVN *** HHHHTT E AAAa

1rkqA 55 60 5.010 -68.10 -37.90 -60.10 -37.10 -89.30 6.20 55.40 42.90 YLKELHME *** HHHHTT AAAa

1rkuA 50 55 5.132 -61.50 -45.10 -59.70 -32.00 -93.20 5.10 68.50 25.70 ILDEHGLK *** HHHHTT AAAa

1rkuA 101 106 4.920 -57.20 -37.50 -74.90 -12.70 -103.70 8.30 81.10 25.10 LMRQLGFP *** HHHHTT AAAa

1rkuA 140 145 5.704 -65.80 -32.10 -68.40 -16.00 -102.00 14.20 70.10 26.20 AFKSLYYR *** HHHHTT E AAAa

1rmwA 79 84 6.176 -66.10 -33.90 -97.10 -1.50 -131.40 25.10 159.50 -25.40 EKMNNGWD *** HHHHTTGG AAAx

1rmwA 127 132 4.679 -70.60 -49.40 -70.10 -32.70 -99.50 3.50 55.50 51.30 LADNFHIP *** HHHHTT AAAa

1rmwA 156 161 4.946 -67.60 -45.60 -57.90 -39.90 -97.00 9.40 47.40 44.40 WLQDKNIR *** HHHHTTEE AAAa

1rmwA 175 180 4.833 -62.90 -35.00 -64.20 -24.60 -106.80 5.80 78.60 17.00 AARDVGAR *** HHHHTT E AAAa

1rq2A 27 32 5.419 -65.20 -46.50 -57.20 -36.90 -88.30 -2.60 83.40 29.30 RMIEQGLK *** HHHHTT AAAa

1rqbA 45 50 7.825 -62.40 -27.30 -107.00 8.00 -76.40 -25.90 103.90 18.30 DIDAAGYW *** HHHHTT S AAAa

1rqbA 115 120 5.629 -64.00 -43.20 -61.30 -35.70 -85.70 0.80 122.50 -6.70 KSAENGXD *** HHHHTT AAAa

1rqbA 142 147 5.508 -66.00 -38.10 -59.60 -39.90 -76.80 -3.70 74.40 21.00 AVKKAGKH *** HHHHTT E AAAa

1rqbA 173 178 5.644 -63.60 -33.60 -68.30 -27.00 -97.00 27.70 85.50 9.80 QLLDXGAD *** HHHHTT S AAAa

1rqbA 230 235 5.493 -67.60 -39.70 -64.50 -30.20 -82.20 -8.20 107.00 14.80 KAIEAGVD *** HHHHTT S AAAa

1rqbA 339 344 5.154 -59.60 -44.60 -60.10 -34.70 -83.50 6.20 87.40 22.60 VRKAAGFP *** HHHHTT AAAa

1rqbA 377 382 5.022 -61.90 -48.90 -61.90 -31.30 -91.60 2.00 84.90 12.70 ADIXLGYY *** HHHHTTTT AAAa

1rqpA 168 173 5.507 -66.00 -41.50 -61.80 -34.10 -77.40 -5.90 108.30 8.20 AHLAAGFP *** HHHHTT AAAa

1rqwA 155 160 4.991 -67.60 -43.20 -77.50 -36.50 -90.80 -10.90 59.40 38.00 TSEYghTT *** HHHHTTT AAAa

1rtqA 132 137 5.267 -67.20 -39.10 -69.70 -20.40 -113.10 14.90 56.50 46.20 VLSENNFQ *** HHHHTT AAAa

1rtqA 165 170 5.224 -64.80 -38.50 -71.60 -30.40 -77.00 -2.30 68.90 27.20 QYKSEGKN *** HHHHTT E AAAa

1rtqA 233 238 5.419 -57.20 -46.40 -66.00 -29.60 -79.20 -1.10 98.10 9.90 SWHNAGYP *** HHHHTT AAAa

1rw1A 21 26 5.415 -63.20 -41.80 -69.50 -29.40 -86.00 5.30 62.00 35.10 WLDEHKVA *** HHHHTT AAAa

1rwjA 53 58 5.341 -63.30 -35.40 -62.60 -26.20 -92.80 10.10 90.30 10.80 ADMDKGKS *** HHHHTT G AAAa

1rx0A 59 64 5.517 -64.50 -31.80 -65.40 -24.70 -86.70 -7.60 105.10 12.70 KAAQLGFG *** HHHHTT S AAAa

1rx0A 126 131 5.069 -75.10 -38.60 -65.40 -28.80 -96.30 -8.10 62.70 30.80 PPLCTMEK *** HHHHTTSS AAAa

1rx0A 317 322 5.338 -69.70 -45.40 -67.50 -24.70 -88.40 0.90 60.50 31.50 VALQEERK *** HHHHTT T AAAa

1rxeA 52 57 5.127 -64.00 -31.00 -63.50 -29.30 -106.20 24.60 61.30 37.20 AMKEVDID *** HHHHTT AAAa

1ryiA 23 28 5.494 -61.10 -40.20 -63.80 -29.80 -83.20 5.40 64.40 25.00 YLAKENKN *** HHHHTT AAAa

1ryiA 161 166 5.295 -60.80 -46.00 -64.20 -23.90 -90.20 4.50 103.40 3.30 AAKMLGAE *** HHHHTT E AAAa

1ryiA 210 215 4.892 -65.50 -42.40 -57.10 -30.30 -103.70 24.30 75.80 24.50 FFKQLGLN *** HHHHTT AAAa

1ryiA 344 349 5.249 -63.50 -48.70 -58.10 -36.30 -81.70 0.80 55.20 35.50 SDLIMNKE *** HHHHTT AAAa

1s0aA 383 388 5.409 -56.90 -39.70 -66.80 -26.80 -86.70 2.40 76.30 28.80 FFVEQGVW *** HHHHTTEE AAAa

1s1dA 200 205 5.604 -61.90 -42.80 -62.30 -26.60 -81.10 1.00 75.60 29.90 LRAAAGIQ *** HHHHTT AAAa

1s2oA 171 176 5.465 -71.10 -34.20 -60.30 -46.10 -81.60 -1.30 56.90 39.80 LQQHLAME *** HHHHTT AAAa

1s2oA 237 242 5.151 -69.00 -46.10 -56.60 -44.50 -87.80 5.10 58.60 43.10 AIAHFDFL *** HHHHTT AAAa

1s3cA 22 27 6.210 -64.70 -35.50 -70.50 -8.40 -93.20 -1.90 100.10 9.40 MIRNSGTE *** HHHHTT AAAa

1s3cA 66 71 5.272 -70.80 -52.60 -76.20 -31.10 -83.50 -4.70 74.70 30.10 PYEQLGLA *** HHHHTTTT AAAa

1s3eA 23 28 5.515 -61.00 -44.00 -65.90 -21.70 -87.30 -5.20 101.60 4.10 LLHDSGLN *** HHHHTT AAAa

1s3eA 72 77 5.305 -61.30 -44.80 -59.80 -33.10 -82.10 -1.60 91.80 18.30 LAKELGLE *** HHHHTT AAAa

1s3eA 451 456 5.334 -66.20 -42.80 -59.40 -31.40 -84.60 -2.70 75.50 21.10 ILHAMGKI *** HHHHTTSS AAAa

1s3zA 104 109 5.434 -63.60 -45.20 -66.60 -19.30 -90.60 6.90 80.70 19.20 WGTNKGCR *** HHHHTT S AAAa

1s3zA 127 132 5.434 -70.00 -39.90 -58.80 -32.80 -90.70 14.70 95.50 6.80 VHQALGFE *** HHHHTT E AAAa

1s4kA 11 16 5.274 -72.90 -38.10 -58.20 -38.10 -95.60 6.20 48.80 55.10 LRRIFDXT *** HHHHTT AAAa

1s4kA 110 115 5.220 -68.90 -38.90 -65.30 -27.00 -92.40 5.10 65.70 28.10 ELFAHDLE *** HHHHTTS AAAa

1s7zA 22 27 5.064 -66.80 -45.00 -61.80 -34.30 -110.30 11.60 45.90 58.60 NIRYDDIR *** HHHHTT AAAa

1s95A 197 202 5.424 -67.50 -41.40 -55.30 -39.50 -84.60 11.90 66.10 30.10 QWYKDQKK *** HHHHTT AAAa

1s99A 140 145 5.146 -71.70 -45.80 -54.70 -35.40 -93.80 -1.50 78.40 17.50 IAKAQGTF *** HHHHTT E AAAa

1s9rA 345 350 5.215 -65.40 -44.00 -54.10 -43.50 -85.00 39.00 52.20 42.70 RETHFDGT *** HHHHTTTT AAAa

1s9rA 374 379 5.221 -58.50 -37.80 -68.60 -23.80 -89.90 2.30 87.40 24.00 ALEAAGIK *** HHHHTT E AAAa

1s9uA 110 115 4.814 -61.60 -44.60 -68.60 -22.40 -100.60 9.80 68.60 29.50 WXRENGIQ *** HHHHTT AAAa

1s9uA 138 143 4.894 -65.50 -38.10 -68.60 -24.90 -107.80 23.90 60.90 27.60 WLAENDRH *** HHHHTT H AAAa

1sacA 171 176 5.285 -59.00 -48.70 -58.60 -32.20 -90.20 1.10 97.70 18.00 LSAYQGTP *** HHHHTT AAAa

1sbp_ 51 56 5.897 -67.70 -41.50 -57.00 -40.20 -93.30 24.40 119.60 -18.10 TSVINGIE *** HHHHTT AAAa

1sbp_ 72 77 5.784 -66.00 -40.10 -59.40 -28.90 -88.90 1.90 86.50 11.10 AIAERGRI *** HHHHTTSS AAAa

1sbp_ 145 150 4.409 -66.40 -20.70 -106.90 1.10 -129.90 30.00 54.80 39.50 ALHHNNND *** HHHHTTT AAAa

1sbp_ 255 260 5.198 -54.90 -45.60 -60.90 -30.40 -94.70 15.80 74.00 29.00 IAAKNFYR *** HHHHTT E AAAa

1sbxA 143 148 5.072 -61.10 -45.20 -62.80 -32.70 -89.80 5.60 53.70 43.70 VCDELHIY *** HHHHTT AAAa

1sbxA 161 166 5.376 -70.40 -43.80 -61.90 -31.20 -79.80 -8.70 78.00 15.30 ILKVXGIL *** HHHHTTSS AAAa

1sdiA 52 57 5.023 -58.00 -34.20 -51.10 -35.00 -104.60 26.80 84.30 6.20 TLAVFGGS *** HHHHTTS AAAa

1sdiA 181 186 5.025 -73.00 -33.30 -65.60 -29.90 -97.10 5.90 113.90 2.30 LWHQVGGG *** HHHHTT AAAa

1sfsA 54 59 5.683 -65.10 -29.40 -60.90 -27.20 -85.90 1.80 74.80 22.20 RIRNYGVK *** HHHHTT E AAAa

1sfsA 88 93 5.326 -68.60 -35.80 -64.40 -35.00 -85.30 -6.30 78.00 23.60 HARRLGIP *** HHHHTT AAAa

1sfxA 57 62 5.197 -65.80 -49.60 -65.20 -32.40 -78.00 -10.60 84.40 20.80 VLLKRGFV *** HHHHTTSE AAAa

1sgwA 45 50 5.239 -73.90 -45.80 -56.50 -35.90 -82.30 2.00 76.30 0.10 LKTISTYL *** HHHHTTSS AAAa

1sgwA 95 100 5.158 -64.70 -28.30 -60.70 -25.10 -99.80 11.30 69.30 19.50 VASLYGVK *** HHHHTT AAAa

1sgwA 111 116 5.142 -57.50 -36.50 -65.40 -21.00 -116.10 13.80 56.40 43.90 ALESVEVL *** HHHHTT AAAa

1sjwA 129 134 5.099 -55.40 -47.80 -67.40 -23.50 -84.90 5.40 78.30 28.80 TLRQLGDP *** HHHHTTS AAAa

1smbA 21 26 5.517 -67.80 -32.70 -66.00 -22.30 -104.00 2.40 67.70 27.80 YRQKHGVP *** HHHHTT AAAa

1smbA 57 62 5.170 -69.30 -46.90 -61.80 -32.60 -89.10 4.80 66.90 19.50 PESSRGQX *** HHHHTTSS AAAa

1snrA 145 150 5.609 -63.50 -46.00 -81.70 0.80 -90.80 8.20 96.70 8.90 WHVVSGMN *** HHHHTT E AAAa

1snrA 209 214 7.228 -56.40 -41.20 -69.90 -14.90 -73.40 -13.50 59.50 23.30 KVMRTLTP *** HHHHTT AAAa

1sqsA 141 146 5.552 -75.10 -39.80 -61.60 -34.80 -73.20 -4.90 110.30 -14.90 IFSYXGGQ *** HHHHTT E AAAa

1sqsA 175 180 5.079 -71.00 -45.70 -58.70 -31.80 -91.20 4.60 69.00 22.60 EDVLEGKI *** HHHHTT AAAa

1sr9A 128 133 4.957 -79.10 -45.50 -73.50 -39.20 -70.80 -11.90 69.90 38.80 EIIEQGAI *** HHHHTT S AAAa

1sr9A 300 305 6.232 -70.60 -41.30 -63.30 -25.20 -76.60 -9.20 121.10 6.30 LGFAAGAD *** HHHHTT AAAa

1sr9A 394 399 5.047 -68.20 -42.20 -64.60 -23.60 -108.10 10.60 58.20 23.20 DADAADCD *** HHHHTT AAAa

1sra_ 270 275 5.224 -61.30 -39.40 -66.30 -29.00 -94.30 14.60 74.40 17.60 WAGbFGIK *** HHHHTT AAAa

1srvA 265 270 5.143 -68.40 -46.80 -66.30 -35.60 -76.90 -16.10 82.80 16.20 VNKLRGTL *** HHHHTTS AAAa

1su8A 106 111 4.848 -59.90 -48.10 -84.20 -10.20 -86.20 3.40 81.10 9.90 KKAVQGKA *** HHHHTTS AAAa

1su8A 176 181 5.025 -62.30 -36.60 -61.90 -29.60 -97.20 6.60 71.70 28.40 VLSAHGLI *** HHHHTT S AAAa

1su8A 280 285 5.435 -63.80 -37.40 -57.60 -27.90 -89.70 0.00 102.40 0.70 EARAAGAT *** HHHHTT S AAAa

1su8A 464 469 5.258 -64.40 -36.80 -66.30 -25.60 -95.00 18.80 69.40 28.60 KLLKQNVL *** HHHHTT E AAAa

1su8A 481 486 5.113 -63.50 -40.30 -72.30 -11.80 -110.00 15.30 91.10 8.20 ALMRHGFM *** HHHHTTTT AAAa

1su8A 508 513 5.316 -62.30 -36.70 -72.10 -19.00 -95.10 7.60 80.30 14.20 IGEANGLG *** HHHHTT S AAAa

1su8A 629 634 5.101 -62.20 -40.20 -66.40 -20.20 -91.70 3.00 82.60 16.40 RRAGLGLP *** HHHHTT AAAa

1sur_ 93 98 4.685 -72.10 -44.60 -65.60 -47.60 -90.80 -0.10 56.20 54.80 LTDKLKLN *** HHHHTT E AAAa

1sur_ 143 148 5.061 -65.50 -53.00 -61.00 -38.50 -82.70 -3.00 59.60 38.50 ALKELNAQ *** HHHHTTEE AAAa

1suzA 56 61 4.974 -60.10 -40.10 -61.80 -26.00 -102.40 5.50 80.60 14.60 IAEKHGYI *** HHHHTT E AAAa

1sw5A 29 34 5.330 -68.60 -35.80 -61.10 -29.10 -102.50 22.10 99.50 -3.50 LLEENGYK *** HHHHTT AAAa

1sw5A 178 183 5.140 -70.10 -38.60 -62.80 -38.60 -83.00 0.50 70.00 18.90 EAIKNKQV *** HHHHTTS AAAa

1sw5A 196 201 5.104 -75.80 -43.60 -55.30 -49.30 -94.30 10.40 57.20 52.70 RVDLFNLK *** HHHHTTEE AAAa

1swxA 110 115 5.536 -65.60 -44.30 -65.40 -31.20 -83.50 -0.10 79.10 18.40 QSICDGER *** HHHHTT AAAa

1swxA 198 203 4.883 -67.70 -46.60 -60.60 -42.80 -81.60 0.00 52.50 44.40 MYTQMNAE *** HHHHTT AAAa

1sx7A 120 125 5.267 -62.80 -43.80 -69.40 -25.40 -79.10 -7.60 63.00 36.80 RMLQQKRW *** HHHHTT H AAAa

1sxrA 17 22 4.730 -54.30 -38.30 -71.10 -24.30 -104.40 6.20 83.50 19.30 LKRQWGGK *** HHHHT AAAa

1sxrA 135 140 4.855 -66.20 -44.30 -62.50 -37.20 -85.90 -0.40 80.90 8.20 aGVQQGEL *** HHHHTTSE AAAa

1syyA 207 212 5.014 -54.10 -43.10 -58.70 -26.20 -99.80 19.30 59.40 33.00 SFHRQNKM *** HHHHTTSS AAAa

1syyA 300 305 4.929 -59.70 -36.20 -69.70 -22.30 -106.80 22.60 78.30 23.70 RLERIGLK *** HHHHTT AAAa

1szoA 172 177 4.999 -77.90 -27.30 -93.80 -18.90 -116.60 5.10 74.30 20.40 YFLLTGQE *** HHHHTT E AAAa

1szwA 63 68 5.216 -58.40 -43.90 -63.80 -36.70 -84.70 -2.50 53.30 40.70 LAKFLKIH *** HHHHTT AAAa

1szwA 247 252 5.198 -61.80 -46.00 -59.40 -37.60 -80.60 3.20 63.70 23.80 RRVNDKEL *** HHHHTTSE AAAa

1szwA 289 294 5.515 -63.80 -49.40 -53.40 -40.80 -88.60 10.50 54.70 41.90 LLVREKVE *** HHHHTT B AAAa

1t06A 220 225 5.413 -66.60 -39.60 -60.20 -29.40 -85.40 -0.70 71.70 29.90 KELDRGRL *** HHHHTT T AAAa

1t0bA 38 43 5.504 -60.20 -46.80 -59.90 -28.20 -86.70 3.10 103.10 3.30 YLAEAGFD *** HHHHTT E AAAa

1t0bA 90 95 5.402 -59.10 -43.80 -63.50 -27.90 -80.50 -6.40 127.50 -12.30 RRVLEGMG *** HHHHTT E AAAa

1t0tV 50 55 5.401 -62.10 -43.90 -64.90 -26.80 -85.20 1.80 64.40 32.60 TTESEKQG *** HHHHTTS AAAa

1t2dA 22 27 5.247 -66.50 -42.70 -53.90 -40.40 -95.60 1.20 52.10 43.10 LIVQKNLG *** HHHHTT AAAa

1t2dA 202 207 5.376 -66.20 -45.10 -58.00 -36.80 -82.30 3.90 61.10 31.50 EFINNKLI *** HHHHTTSS AAAa

1t2dA 248 253 4.687 -64.40 -54.30 -63.60 -35.90 -102.40 4.90 53.10 47.90 ESYLKDLK *** HHHHTT AAAa

1t3iA 82 87 4.821 -67.50 -33.40 -60.10 -37.40 -108.40 10.50 58.10 39.50 VAKFINAR *** HHHHTT S AAAa

1t3iA 195 200 5.111 -63.10 -33.00 -67.10 -33.60 -81.50 -8.00 88.80 18.70 LAHQAGAK *** HHHHTT E AAAa

1t3iA 217 222 6.198 -74.20 -32.50 -72.90 -47.50 -75.90 -20.90 58.40 33.90 DVQLIDCD *** HHHHT S AAAa

1t3iA 377 382 4.888 -61.70 -40.40 -54.90 -36.40 -108.60 25.10 47.80 45.10 LHRLFDAS *** HHHHTT AAAa

1t3tA 95 100 5.175 -63.40 -43.50 -72.10 -7.80 -103.40 14.40 78.80 12.20 IAHNCGLQ *** HHHHTT T AAAa

1t3tA 175 180 4.855 -77.30 -44.80 -62.30 -49.10 -88.40 -4.70 63.80 50.60 ANLRLGLA *** HHHHTT AAAa

1t3tA 516 521 4.993 -62.90 -44.40 -63.30 -23.70 -94.50 -0.70 73.90 20.50 LVSDGGRG *** HHHHTT E AAAa

1t3tA 844 849 4.953 -57.10 -42.50 -63.60 -19.50 -113.00 23.10 55.90 36.80 LAQVYRQL *** HHHHTT AAAa

1t3tA 873 878 5.135 -65.30 -47.60 -65.30 -33.20 -84.20 -4.10 61.40 36.20 ALVAARKL *** HHHHTT AAAa

1t3tA 943 948 5.475 -65.60 -31.30 -67.20 -18.20 -103.20 13.70 74.00 20.40 LLAQYGLA *** HHHHTT G AAAa

1t3tA 1060 1065 5.460 -62.60 -44.80 -59.40 -31.60 -86.80 2.60 99.60 10.80 AFHRAGFD *** HHHHTT E AAAa

1t3tA 1212 1217 6.172 -63.10 -46.00 -65.00 -27.40 -77.60 -14.50 74.80 31.60 ALESKGLV *** HHHHTT E AAAa

1t4bA 21 26 4.848 -71.70 -39.70 -61.60 -38.00 -93.90 3.50 55.50 41.40 RMVEERDF *** HHHHTTGG AAAa

1t4bA 85 90 5.548 -63.30 -38.80 -67.80 -18.80 -85.60 -4.80 94.30 7.00 KLRESGWQ *** HHHHTT AAAa

1t4bA 122 127 5.075 -56.80 -39.30 -74.70 -20.60 -104.40 25.30 81.00 5.40 DGLNNGIR *** HHHHTT AAAa

1t4bA 147 152 5.320 -74.10 -41.60 -57.70 -34.60 -93.90 13.70 48.80 50.00 GLFANDLV *** HHHHTT E AAAa

1t4fM 61 66 5.341 -62.70 -52.50 -58.40 -33.10 -92.40 2.40 48.40 48.50 YIMTKRLY *** HHHHTT E AAAa

1t5hX 46 51 5.777 -65.90 -44.50 -58.60 -35.10 -74.10 -8.90 123.60 -13.70 RLHADGLR *** HHHHTT AAAa

1t5hX 97 102 5.947 -62.80 -53.80 -54.60 -36.50 -78.00 -8.70 53.20 53.00 LIKRGEXT *** HHHHTT S AAAa

1t5hX 218 223 5.274 -74.80 -39.90 -60.70 -27.10 -95.00 9.40 73.10 28.90 AALALDGT *** HHHHTT E AAAa

1t6cA 74 79 5.282 -65.70 -49.70 -65.10 -35.80 -84.40 -0.40 55.30 46.30 LIDEFKVE *** HHHHTT S AAAa

1t6cA 218 223 4.545 -63.50 -49.00 -59.90 -40.60 -101.40 11.20 57.50 43.80 AALEYNVY *** HHHHTT AAAa

1t6t1 86 91 5.404 -67.50 -37.50 -64.50 -25.50 -93.40 4.00 96.40 5.20 LLSSQGFL *** HHHHTT E AAAa

1t6t1 101 106 4.983 -56.10 -45.90 -60.40 -39.00 -92.20 9.10 58.70 39.00 FLKKWNII *** HHHHTT AAAa

1t7rA 775 780 6.502 -69.50 -45.70 -61.50 -29.10 -73.20 -11.80 67.90 15.30 RMHKSRMY *** HHHHTT H AAAa

1t7rA 794 799 5.210 -83.20 -45.50 -60.70 -39.60 -86.80 -2.00 53.00 48.30 EFGWLQIT *** HHHHTT AAAa

1t7rA 905 910 5.174 -74.30 -47.50 -64.30 -28.20 -90.10 3.20 82.60 8.60 PKILSGKV *** HHHHTTSE AAAa

1t82A 19 24 5.612 -74.10 -42.80 -64.10 -42.80 -73.70 -7.50 55.30 45.70 VSEFXQIA *** HHHHTT E AAAa

1t8uA 327 332 4.903 -64.90 -48.70 -54.70 -38.30 -85.50 -3.50 75.00 27.90 VQDFLGLK *** HHHHTT AAAa

1t9iA 133 138 5.791 -66.80 -37.20 -71.20 -33.50 -74.20 172.40 -88.90 60.90 IAALNDSK *** HHHHTT AAPD

1tafA 29 34 5.147 -68.40 -43.90 -61.60 -31.70 -92.70 18.80 62.50 32.30 ILKELNVQ *** HHHHTT AAAa

1tafA 64 69 5.080 -61.90 -50.30 -65.90 -15.60 -97.50 -5.10 63.90 50.70 YANHARKK *** HHHHTT S AAAa

1tafB 16 21 5.512 -69.30 -38.00 -64.20 -21.60 -109.90 2.50 72.60 21.00 IAESIGVG *** HHHHTT AAAa

1tafB 51 56 5.349 -62.40 -39.70 -58.10 -30.30 -91.40 7.40 62.50 27.60 FMNHAKRQ *** HHHHTT S AAAa

1tag_ 84 89 4.899 -72.00 -56.30 -51.80 -44.10 -90.60 -7.20 55.70 61.30 AMTTLNIQ *** HHHHTT AAAa

1tbfA 551 556 5.791 -63.80 -47.60 -59.20 -39.10 -83.30 -9.90 67.40 15.30 SAQTLKIT *** HHHHTTT AAAa

1tbfA 579 584 5.342 -72.50 -48.80 -75.90 -16.10 -86.20 8.10 65.90 18.90 MFTDLNLV *** HHHHTTHH AAAa

1tbfA 585 590 4.950 -69.10 -57.00 -63.10 -26.80 -111.70 13.70 45.00 53.70 LVQNFQMK *** HHHHTT AAAa

1tbfA 665 670 5.138 -66.40 -47.70 -66.70 -27.50 -95.70 6.40 56.10 43.30 FLINTNSE *** HHHHTT H AAAa

1tbfA 673 677 4.742 -63.40 -47.10 -58.80 -40.20 -104.60 13.40 55.20 39.60 LALMYNDE *** HHHHTTTS AAAa

1tbfA 738 743 5.031 -66.80 -44.90 -58.20 -37.80 -85.00 1.40 62.30 37.30 ELIRKNQF *** HHHHTT AAAa

1tca_ 91 96 4.928 -67.30 -42.70 -66.70 -15.90 -105.40 15.20 88.10 30.90 LYAGSGNN *** HHHHTTS AAAa

1tca_ 144 149 5.494 -61.10 -47.20 -62.50 -29.80 -85.90 -0.10 60.70 34.50 PLDALAVS *** HHHHTT B AAAa

1tca_ 167 172 5.002 -63.30 -47.90 -68.10 -22.90 -84.50 -5.90 96.60 15.00 ALRNAGGL *** HHHHTTTT AAAa

1tdzA 178 183 5.187 -65.10 -46.70 -59.30 -42.50 -84.10 4.20 56.10 45.00 VLWLAKIH *** HHHHTT AAAa

1tdzA 211 216 5.061 -60.10 -49.10 -54.10 -38.70 -88.60 6.50 75.60 24.50 KAIKLGGS *** HHHHTT AAAa

1tg5A 135 140 5.305 -68.20 -33.70 -62.30 -28.90 -102.50 26.20 99.60 6.80 ISVANGAI *** HHHHTT AAAa

1tg7A 224 229 5.483 -62.00 -40.60 -66.20 -21.80 -87.60 -0.10 110.80 -1.80 HARDAGIV *** HHHHTT AAAa

1tg7A 863 868 5.250 -74.50 -44.40 -58.50 -34.10 -79.90 -16.20 83.60 18.00 YAERQGFH *** HHHHTTTT AAAa

1thfD 39 44 5.274 -54.00 -41.10 -78.90 -20.00 -103.60 8.50 112.10 -0.80 FYSEIGID *** HHHHTT AAAa

1thfD 92 97 5.579 -66.20 -42.20 -58.70 -34.10 -83.10 -1.80 123.40 -5.60 ELILRGAD *** HHHHTT S AAAa

1thfD 160 165 5.931 -67.30 -41.40 -59.70 -34.10 -76.60 -7.40 100.50 14.80 EVEKRGAG *** HHHHTT S AAAa

1thfD 213 218 5.786 -68.50 -37.10 -66.30 -26.90 -78.80 -8.80 116.20 0.90 EAFLAGAD *** HHHHTT S AAAa

1thfD 226 231 4.863 -89.20 -40.90 -62.50 -42.50 -92.60 8.00 49.50 53.70 SVFHFREI *** HHHHTT S AAAa

1thfD 241 246 5.265 -65.60 -34.80 -64.20 -27.90 -98.60 21.90 78.90 7.70 YLKKHGVN *** HHHHTT AAAa

1ti6B 77 82 6.775 -57.40 -45.80 -111.90 21.40 -92.10 -2.80 55.00 34.40 CVAKGNGA *** HHHHTTTS AAAa

1tif_ 38 43 5.094 -62.40 -43.10 -65.70 -24.00 -95.70 1.90 56.70 41.60 IAARRNLD *** HHHHTT E AAAa

1tjoA 85 90 5.128 -64.20 -39.70 -63.30 -32.40 -86.90 6.40 87.10 17.60 RVQALGGV *** HHHHTT AAAa

1tjoA 140 145 5.126 -69.00 -35.00 -66.60 -32.10 -81.70 -4.90 83.40 14.00 LAENLGDH *** HHHHTT H AAAa

1tjvA 51 56 5.164 -66.10 -41.30 -63.10 -34.90 -78.00 -4.30 83.20 9.90 ALEKAGIL *** HHHHTTSS AAAa

1tjvA 379 384 5.652 -63.00 -37.90 -71.00 -18.40 -94.20 2.20 113.60 -25.70 YLVRKGVP *** HHHHTT AAAa

1tjvA 400 405 5.100 -63.10 -50.40 -58.60 -27.80 -90.10 -4.80 65.10 32.70 LAETKGIT *** HHHHTTS AAAa

1tjyA 80 85 5.467 -61.70 -47.90 -66.60 -27.30 -87.60 0.40 86.70 21.50 NFVNQGYD *** HHHHTT S AAAa

1tjyA 105 110 5.281 -68.60 -35.10 -59.30 -32.00 -91.60 4.60 82.10 12.90 RAMQRGVK *** HHHHTT E AAAa

1tjyA 232 237 4.942 -65.80 -45.20 -59.10 -37.20 -96.90 3.20 55.50 41.30 AAENLKRN *** HHHHTT AAAa

1tjyA 281 286 5.247 -64.30 -41.20 -68.20 -25.20 -94.30 5.60 58.40 33.60 NALLKNMP *** HHHHTT AAAa

1tkeA 148 153 5.095 -63.70 -41.40 -66.10 -20.20 -97.60 -0.50 69.70 20.00 TFANRGES *** HHHHTT H AAAa

1tn6A 141 146 5.078 -74.90 -51.50 -56.60 -39.10 -93.10 -0.80 52.20 49.30 LLKSLQKD *** HHHHTT AAAa

1tn6A 210 215 4.523 -72.50 -43.00 -67.90 -36.60 -109.50 11.80 50.90 52.40 VIQEFKLW *** HHHHTT AAAa

1tn6A 244 249 5.413 -60.50 -28.60 -94.70 -5.50 -112.20 -87.30 -78.90 -33.00 VISNTTGY *** HHHHTT S AAAA

1tn6A 322 327 5.264 -64.40 -29.10 -77.30 -13.90 -117.30 34.90 50.20 51.10 DMLENQCD *** HHHHTT S AAAa

1tn6B 611 616 5.244 -61.50 -45.10 -62.20 -37.10 -80.70 0.80 56.00 42.80 SLELLDEP *** HHHHTT AAAa

1tn6B 711 716 5.116 -71.40 -43.20 -62.30 -42.40 -90.00 5.30 54.90 56.40 VASLTNII *** HHHHTT AAAa

1tn6B 815 820 5.591 -64.20 -34.40 -67.30 -26.50 -86.50 -3.00 78.60 30.40 ALHAQGDP *** HHHHTT T AAAa

1tqgA 72 77 4.990 -69.20 -37.30 -70.40 -23.30 -96.10 14.10 58.10 32.20 DKARNSEI *** HHHHTTSS AAAa

1tqhA 39 44 5.221 -53.40 -43.30 -66.00 -20.80 -96.30 5.40 105.20 -1.60 FLESKGYT *** HHHHTT E AAAa

1tqjA 26 31 5.685 -61.00 -53.30 -64.50 -27.80 -74.10 -6.30 106.80 10.80 AVDEAGAD *** HHHHTT S AAAa

1tqjA 108 113 5.654 -71.20 -32.30 -65.60 -26.00 -80.60 4.50 76.20 17.50 QIRELGKK *** HHHHTT E AAAa

1tqmA 54 59 5.201 -72.90 -36.70 -65.70 -35.10 -84.10 6.20 64.70 31.20 YLSDLRVV *** HHHHTTSE AAAa

1tqmA 85 90 5.534 -68.60 -42.30 -71.20 -27.80 -82.60 -2.70 75.10 14.90 RLVRSGKV *** HHHHTTS AAAa

1tqmA 209 214 5.065 -56.80 -39.90 -67.90 -23.30 -88.60 3.60 82.90 22.20 KFYHRGIV *** HHHHTTEE AAAa

1tr9A 150 155 5.389 -62.40 -36.90 -56.90 -32.10 -100.00 4.80 89.80 23.30 GXKAVGXA *** HHHHTT AAAa

1tu9A 60 65 5.566 -57.40 -45.60 -67.60 -18.40 -90.90 -1.80 111.50 8.90 VMYARGMS *** HHHHTT AAAa

1tv9A 114 119 4.871 -58.70 -37.10 -58.40 -33.80 -91.50 7.60 89.90 6.10 KFVDEGIK *** HHHHTT AAAa

1tv9A 180 185 5.428 -57.40 -48.50 -66.00 -27.40 -81.10 7.70 77.80 16.60 GSFRRGAE *** HHHHTT S AAAa

1tv9A 218 223 5.295 -75.80 -35.70 -62.20 -38.60 -88.90 14.40 63.70 23.80 QLQKVHFI *** HHHHTT E AAAa

1tv9A 286 291 4.844 -57.70 -39.00 -62.40 -31.20 -95.90 12.00 81.80 31.70 HALEKGFT *** HHHHTTEE AAAa

1tv9A 320 325 5.108 -58.00 -53.10 -47.50 -41.50 -94.30 13.30 53.90 52.60 IFDYIQWK *** HHHHTT AAAa

1tvzA 23 28 5.295 -55.50 -44.70 -70.50 -30.20 -90.50 9.40 55.60 44.30 LAEARQVD *** HHHHTTS AAAa

1tw6A 108 113 5.367 -64.20 -41.90 -59.70 -34.40 -85.40 9.20 98.80 -1.90 LLAAAGFF *** HHHHTTEE AAAa

1twdA 17 22 5.212 -57.80 -50.60 -62.20 -19.40 -104.30 16.90 100.20 3.80 TAQQNGAD *** HHHHTT S AAAa

1twdA 82 87 5.836 -68.20 -45.40 -64.50 -31.40 -74.40 -10.10 90.70 15.30 TVRELGFP *** HHHHTT S AAAa

1twiA 94 99 5.548 -61.60 -46.00 -61.70 -26.50 -76.60 -7.30 98.60 6.30 LLAKLGCG *** HHHHTT E AAAa

1twiA 112 117 5.612 -67.50 -42.70 -67.70 -14.60 -92.90 0.00 71.40 25.40 IAKLSNVP *** HHHHTT AAAa

1twiA 137 142 5.631 -69.50 -44.30 -62.40 -34.90 -83.50 -5.30 51.80 51.50 MGIEANIR *** HHHHTT S AAAa

1twiA 249 254 5.070 -60.60 -37.70 -61.70 -27.70 -88.00 11.20 79.30 14.80 ELKEEGIE *** HHHHTT AAAa

1twyA 71 76 5.339 -65.80 -41.10 -71.00 -24.70 -87.90 -0.70 75.10 16.30 SLLKKGVA *** HHHHTTS AAAa

1twyA 227 232 5.021 -73.80 -39.60 -70.90 -28.70 -96.90 -1.00 60.30 25.20 DNIAKHTY *** HHHHTT S AAAa

1twyA 268 273 5.732 -60.90 -44.70 -64.70 -29.50 -81.70 6.60 92.50 14.20 LIVEYGYI *** HHHHTT B AAAa

1tx4A 99 104 5.263 -62.70 -46.90 -65.80 -29.80 -80.00 -5.90 71.20 24.30 QKYNMGLP *** HHHHTT AAAa

1txgA 40 45 5.009 -64.90 -40.60 -64.60 -33.40 -83.50 -2.10 77.10 15.80 KSISAGRE *** HHHHTT AAAa

1txgA 143 148 5.235 -57.80 -47.00 -61.20 -30.50 -90.70 7.70 65.90 29.50 REVAKRMP *** HHHHTT AAAa

1txgA 266 271 5.771 -65.40 -40.40 -62.90 -29.40 -73.70 -11.40 105.30 4.70 ELLGKGLS *** HHHHTT AAAa

1txgA 280 285 5.158 -61.70 -45.10 -58.60 -36.80 -80.20 -2.80 83.10 15.30 ELERRGVG *** HHHHTT AAAa

1txgA 301 306 5.095 -64.40 -40.70 -60.50 -35.10 -100.90 6.90 57.00 36.70 LSSKINAD *** HHHHTT AAAa

1txgA 330 335 4.887 -68.70 -48.30 -70.10 -15.70 -108.60 14.50 72.40 28.20 FELATFK# *** HHHHTT AAAa

1tyv_ 239 244 6.619 -83.80 -46.50 -62.00 -42.60 -120.50 83.10 -47.20 122.10 DYVKFPGI *** HHHHTTTH AADP

1tzpA 85 90 5.612 -60.20 -43.10 -71.60 -17.70 -85.00 5.30 77.90 18.40 QVSNLGMG *** HHHHTT AAAa

1tzpA 268 273 5.221 -71.30 -44.30 -63.60 -31.70 -94.10 5.30 69.90 36.30 ALLDEHVI *** HHHHTT AAAa

1tzyA 34 39 6.889 -71.10 -38.30 -65.50 -25.30 -81.50 -6.00 48.80 56.30 LLRKGNYA *** HHHHTTSS AAAa

1tzyA 70 75 4.881 -67.20 -44.80 -53.00 -21.90 -110.20 28.10 64.00 36.10 AARDNKKT *** HHHHTT S AAAa

1tzyB 81 86 4.834 -60.40 -47.00 -52.50 -40.40 -96.40 8.50 53.90 47.70 LAHYNKRS *** HHHHTT S AAAa

1tzyC 111 116 5.099 -60.90 -37.80 -59.60 -34.70 -90.10 14.00 62.60 36.60 CAIHAKRV *** HHHHTT S AAAa

1tzyD 38 43 5.327 -64.00 -41.60 -61.60 -30.20 -79.00 4.10 80.90 21.30 LARRGGVK *** HHHHTT AAAa

1tzyD 73 78 5.312 -68.20 -37.50 -60.50 -39.60 -80.50 0.20 58.40 41.10 YTEHAKRK *** HHHHTT S AAAa

1tzyD 90 95 5.163 -58.40 -34.40 -61.90 -35.00 -85.60 11.90 77.00 16.50 ALKRQGRT *** HHHHTT E AAAa

1u0fA 71 76 5.633 -62.40 -45.40 -70.00 -21.90 -90.20 0.50 72.30 35.30 LAKSRGVE *** HHHHTTHH AAAa

1u0fA 82 87 5.189 -57.40 -52.30 -65.90 -26.10 -91.70 -0.60 87.80 16.30 DNMFSGSK *** HHHHTT AAAa

1u0fA 134 139 5.189 -72.10 -49.90 -68.20 -29.40 -74.50 -16.00 92.10 -3.00 QRVRSGDW *** HHHHTT S AAAa

1u0fA 448 453 5.893 -60.00 -46.80 -68.90 -22.80 -80.20 -3.20 81.00 30.40 ELQAAGKS *** HHHHTT AAAa

1u14A 141 146 4.581 -66.10 -51.40 -77.40 -15.40 -99.80 10.10 53.90 36.40 IGVFTAGK *** HHHHTTTS AAAa

1u4gA 276 281 4.876 -60.90 -40.90 -59.30 -36.90 -92.00 4.90 65.00 34.30 SAQNRNYS *** HHHHTT AAAa

1u4gA 290 295 4.902 -62.30 -31.70 -63.60 -27.80 -103.70 19.50 76.40 20.20 AFSTVGVT *** HHHHTT AAAa

1u60A 104 109 4.696 -68.90 -29.80 -70.60 -13.10 -125.60 29.10 59.20 30.90 ALELHGGK *** HHHHTTT AAAa

1u60A 211 216 5.103 -68.60 -43.30 -60.50 -33.60 -82.30 10.60 69.70 30.30 ATLAAGGV *** HHHHTTSE AAAa

1u7pA 58 63 5.128 -59.30 -40.60 -67.40 -33.60 -82.40 -0.80 77.60 20.00 RLQSLGVP *** HHHHTT AAAa

1u7pA 81 86 5.178 -66.50 -57.10 -55.10 -35.40 -84.70 10.50 52.50 44.30 LLELFDLG *** HHHHTT G AAAa

1ua4A 256 261 5.206 -62.40 -39.70 -59.90 -39.90 -86.20 4.10 57.40 42.40 VLNEREIP *** HHHHTT AAAa

1ua4A 300 305 4.904 -54.60 -45.70 -82.40 -9.00 -96.40 2.90 85.30 2.10 IMEILGEK *** HHHHTT H AAAa

1ualA 25 30 5.085 -70.70 -48.20 -60.90 -31.20 -90.60 7.40 64.10 25.50 RAVKHNLL *** HHHHTTSE AAAa

1uasA 83 88 5.224 -62.40 -35.90 -65.90 -23.10 -89.20 1.10 82.20 19.00 YVHAKGLK *** HHHHTT E AAAa

1uasA 237 242 5.209 -63.60 -50.10 -60.80 -36.00 -81.50 8.40 54.70 41.50 IWALAKAP *** HHHHTT AAAa

1ubkL 177 182 7.137 -71.50 -34.50 -72.40 -14.30 -70.00 -15.70 78.00 -3.50 TFVETGQL *** HHHHTT AAAa

1ubkL 394 399 5.292 -52.50 -53.50 -68.70 -15.70 -87.80 -2.60 84.10 23.00 IAYSQGHP *** HHHHTT H AAAa

1ubkL 451 456 5.921 -60.60 -41.40 -67.90 -22.20 -72.70 -7.10 95.80 10.30 DNIAKGDN *** HHHHTT AAAa

1ubkS 210 215 5.382 -62.40 -43.20 -56.60 -38.80 -82.40 2.10 83.30 25.00 EEARKGWC *** HHHHTT B AAAa

1ucrA 46 51 5.396 -67.20 -44.40 -56.20 -41.30 -76.10 -12.00 71.30 22.70 ALVNDEVL *** HHHHTTSE AAAa

1uehA 32 37 4.918 -59.00 -37.70 -60.40 -27.10 -110.00 21.50 67.00 31.60 WAKKQGKI *** HHHHTT AAAa

1uehA 57 62 5.240 -60.20 -42.70 -75.20 -12.50 -111.90 23.90 62.10 21.10 FAANNGIE *** HHHHTT S AAAa

1uehA 100 105 5.251 -61.20 -43.30 -67.60 -15.30 -101.30 10.60 60.60 36.80 SLHRHNVR *** HHHHTT E AAAa

1uehA 161 166 5.250 -67.20 -40.30 -68.90 -34.10 -78.90 -0.90 80.90 5.60 EKVQQGNL *** HHHHTTS AAAa

1uekA 68 73 4.930 -61.30 -51.50 -62.50 -28.90 -90.40 1.50 80.40 31.20 YLEAAGQP *** HHHHTT AAAa

1uekA 220 225 5.843 -59.30 -41.50 -64.40 -25.50 -84.00 7.70 89.70 7.00 RMRALGLR *** HHHHTT E AAAa

1ufoA 122 127 5.281 -63.80 -37.90 -67.70 -21.30 -101.40 14.60 86.10 16.90 LLLAEGFR *** HHHHTT AAAa

1ufyA 62 67 5.354 -70.90 -22.00 -68.00 -23.70 -105.40 23.50 83.70 20.50 AARQIGMH *** HHHHTTGG AAAa

1ug6A 106 111 5.031 -59.50 -39.80 -64.30 -24.10 -99.60 8.80 74.40 25.40 RLLASGIT *** HHHHTT E AAAa

1ug6A 206 211 5.381 -62.20 -41.70 -66.40 -26.00 -93.80 10.90 94.90 10.80 ALRAAGAR *** HHHHTT S AAAa

1ug6A 244 249 4.949 -58.40 -43.00 -64.10 -29.60 -84.50 1.00 80.50 21.20 LDPILGKG *** HHHHTTS AAAa

1ugpA 28 33 5.845 -68.60 -43.70 -65.30 -28.50 -75.70 -11.40 78.90 11.60 MLIEQGIL *** HHHHTTS AAAa

1ugpB 40 45 5.308 -66.60 -48.10 -62.70 -36.50 -76.40 -3.00 79.90 20.40 ATFRAGFM *** HHHHTTS AAAa

1ugpB 80 85 4.818 -71.40 -42.90 -55.80 -46.10 -85.60 -5.70 76.30 16.30 HGVRTGKI *** HHHHTT S AAAa

1uhkA 66 71 5.686 -57.80 -36.60 -70.50 -22.00 -89.70 9.90 89.60 3.20 FFGGAGMK *** HHHHTT AAAa

1uhkA 96 101 4.797 -61.90 -41.70 -68.00 -28.70 -90.20 7.60 59.20 34.90 EKYAKNEP *** HHHHTT AAAa

1uiuA 208 213 5.071 -69.30 -40.60 -57.60 -45.50 -79.50 -9.00 88.70 1.60 VAFETGDI *** HHHHTTS AAAa

1uiuA 319 324 5.128 -53.70 -45.80 -67.30 -27.70 -85.40 4.10 87.30 9.60 LLEKAGWT *** HHHHTT B AAAa

1uiuA 458 463 5.501 -69.30 -54.10 -67.50 -30.00 -86.70 5.50 52.60 50.60 RLHDEAVY *** HHHHTT E AAAa

1uj2A 41 46 5.577 -66.10 -42.20 -68.40 -20.50 -86.90 2.00 70.60 19.10 IVQLLGQN *** HHHHTTGG AAAa

1uj2A 75 80 4.821 -59.60 -40.10 -60.50 -24.40 -100.10 8.80 66.00 28.30 AKALKGQF *** HHHHTT S AAAa

1uj2A 100 105 5.394 -61.90 -41.40 -65.20 -24.20 -85.90 1.30 81.10 12.30 KEITEGKT *** HHHHTT AAAa

1uj6A 44 49 4.625 -73.00 -46.30 -61.90 -38.20 -85.90 2.40 70.20 14.20 RRLREGEL *** HHHHTTSS AAAa

1uj6A 66 71 5.217 -64.70 -40.50 -62.00 -34.20 -85.00 -1.90 98.50 -1.40 LAKREGIP *** HHHHTT AAAa

1uk8A 89 94 4.731 -61.60 -45.30 -59.70 -31.00 -99.80 8.30 58.10 37.30 IMDALEIE *** HHHHTT AAAa

1ukkA 66 71 5.029 -62.20 -41.50 -62.00 -22.90 -92.40 -0.40 92.70 17.30 SLEREGFP *** HHHHTT AAAa

1ukuA 21 26 5.346 -64.70 -46.00 -59.20 -36.00 -83.50 -0.60 62.10 31.10 TLLKERLI *** HHHHTTS AAAa

1umgA 37 42 5.178 -68.70 -42.40 -62.90 -37.60 -77.90 -7.00 83.10 7.20 SAKEQGII *** HHHHTTS AAAa

1umgA 85 90 5.146 -65.10 -45.40 -64.50 -28.90 -87.40 -3.10 65.40 26.50 VAKDLGLY *** HHHHTT B AAAa

1unkA 76 81 4.903 -62.80 -47.50 -62.10 -27.80 -100.30 15.00 63.30 30.70 WRAANGKP *** HHHHTT AAAa

1uozA 150 155 5.166 -59.00 -40.70 -66.00 -22.40 -91.70 0.40 64.30 35.60 AAQAAGAM *** HHHHTT B AAAa

1uozA 260 265 5.192 -62.50 -37.80 -65.20 -31.40 -96.00 9.20 81.00 40.70 RLNDVGVG *** HHHHTTGG AAAa

1uozA 292 297 4.775 -61.90 -28.50 -91.20 -0.40 -129.80 37.00 58.00 38.50 ISGLTNGS *** HHHHTTT AAAa

1uozA 375 380 5.204 -56.80 -42.70 -70.90 -20.00 -85.40 6.20 86.20 7.70 LAHNAGQ# *** HHHHTT AAAa

1upgA 57 62 5.078 -78.10 -30.00 -65.40 -35.80 -91.00 -2.10 83.20 6.30 ETYKTGQA *** HHHHTTS AAAa

1uq5A 191 196 4.691 -72.60 -41.00 -59.00 -35.40 -110.20 9.70 49.20 41.60 TRIRYNRR *** HHHHTT AAAa

1urqC 78 83 5.206 -69.90 -34.90 -69.50 -19.40 -88.40 0.30 74.50 25.00 NLKDLGK# *** HHHHTT AAAa

1us0A 61 66 5.335 -67.00 -44.00 -64.20 -43.50 -59.30 -19.70 61.20 35.30 EKLREQVV *** HHHHTTSS AAAa

1us0A 147 152 5.390 -60.60 -47.80 -69.70 -13.60 -100.30 -2.20 82.70 7.30 ELVDEGLV *** HHHHTTSB AAAa

1us0A 199 204 5.141 -60.20 -38.90 -61.60 -24.50 -98.00 4.80 80.30 9.80 YCQSKGIV *** HHHHTT E AAAa

1us0A 252 257 5.263 -55.50 -41.40 -77.80 -4.60 -95.40 10.20 66.40 31.20 FPMQRNLV *** HHHHTT E AAAa

1us5A 66 71 5.054 -69.10 -41.10 -61.10 -37.90 -79.40 -6.60 81.20 13.90 NAINAGEF *** HHHHTTS AAAa

1us5A 151 156 4.877 -59.90 -38.90 -62.60 -22.40 -106.60 14.80 74.10 20.80 ILEAYGLT *** HHHHTT AAAa

1us5A 175 180 4.866 -64.00 -46.20 -60.40 -37.60 -87.80 7.90 61.10 27.20 QLMQDKRA *** HHHHTTS AAAa

1us5A 307 312 5.224 -63.50 -45.80 -62.00 -35.40 -79.50 -7.20 76.70 21.00 FYKEAGVL *** HHHHTT AAAa

1usgA 33 38 5.238 -61.40 -39.20 -68.10 -18.80 -96.80 6.60 116.40 6.70 DINAKGGI *** HHHHTTTB AAAa

1usgA 65 70 5.667 -59.90 -47.50 -64.60 -34.40 -75.70 -8.40 77.70 16.80 KIVNDGIK *** HHHHTT AAAa

1usgA 162 167 5.326 -62.70 -39.50 -63.20 -27.70 -92.60 12.50 61.90 31.60 GLKAANAN *** HHHHTT AAAa

1usgA 189 194 5.221 -68.60 -45.90 -65.20 -33.10 -92.60 6.40 50.00 51.20 RLKKENID *** HHHHTT AAAa

1usgA 213 218 5.267 -72.00 -27.60 -61.70 -27.10 -104.00 10.70 75.30 24.10 QARSVGLK *** HHHHTT AAAa

1usgA 265 270 4.941 -59.00 -41.50 -56.90 -33.80 -90.70 11.40 60.40 38.70 ALKADKKD *** HHHHTT AAAa

1uslA 25 30 5.126 -59.90 -48.30 -64.90 -23.80 -103.00 11.30 95.00 10.70 HLKQTGHE *** HHHHTT E AAAa

1uujA 19 24 4.785 -68.20 -45.60 -76.30 -21.70 -100.40 6.80 95.60 -0.30 YLRSNGYE *** HHHHTT H AAAa

1uujA 32 37 4.786 -63.00 -50.90 -68.30 -42.80 -79.30 -3.50 55.30 61.50 FKKEAELD *** HHHHTT AAAa

1uuyA 16 21 5.070 -68.10 -41.50 -62.20 -36.40 -80.90 -5.10 77.50 19.40 DTVSAGAG *** HHHHTTSS AAAa

1uwcA 173 178 4.851 -66.60 -49.40 -66.80 -22.60 -115.10 17.00 53.40 41.50 MNDAFQVS *** HHHHTTTT AAAa

1uwkA 121 126 5.046 -62.60 -41.70 -61.40 -25.10 -90.50 3.10 76.10 21.10 ELDAKGLA *** HHHHTT AAAa

1uwkA 186 191 5.182 -56.00 -46.00 -66.80 -29.30 -77.10 -7.50 101.10 3.80 AATLAGAC *** HHHHTT E AAAa

1uwkA 206 211 5.029 -67.90 -43.60 -62.80 -31.70 -96.20 2.10 61.70 37.40 FRLETRYV *** HHHHTTS AAAa

1uwkA 229 234 5.029 -66.10 -39.50 -61.30 -32.60 -88.10 -6.50 71.50 24.60 KYTAEGKA *** HHHHTT AAAa

1uwkA 313 318 5.444 -59.10 -40.20 -63.70 -25.70 -78.50 2.30 92.10 3.40 DFQKQGVP *** HHHHTT AAAa

1uwkA 331 336 5.435 -59.70 -40.70 -69.80 -23.40 -94.10 16.60 100.10 3.70 MAKEEGVA *** HHHHTT T AAAa

1uwkA 533 538 5.883 -68.00 -37.90 -61.40 -37.80 -71.40 -13.40 98.20 15.00 RHADAGYD *** HHHHTT H AAAa

1uwkA 545 550 5.198 -68.60 -42.70 -61.00 -38.80 -90.00 -1.00 70.50 30.30 CAKEQGLD *** HHHHTT B AAAa

1uylA 102 107 5.289 -55.50 -49.30 -63.70 -38.60 -84.20 57.40 55.50 44.00 ADLINNLG *** HHHHTGGG AADa

1uylA 121 126 5.308 -58.50 -27.20 -79.90 -31.90 -89.80 26.10 95.90 7.20 EALQAGAD *** HHHHTT AAAa

1uzbA 197 202 5.438 -64.30 -37.10 -54.20 -40.00 -93.30 10.30 113.00 0.40 GPVAVGNT *** HHHHTT E AAAa

1uzeA 208 213 5.190 -62.60 -38.30 -69.70 -9.50 -109.30 9.20 95.70 2.90 AARLNGYV *** HHHHTT S AAAa

1uzeA 305 310 5.052 -66.90 -43.70 -52.80 -45.70 -98.70 38.30 53.60 34.30 AMLKQGWT *** HHHHTT AAAa

1uzeA 323 328 5.296 -63.40 -29.70 -64.50 -26.70 -86.90 0.40 89.40 5.20 FFTSLGLL *** HHHHTT AAAa

1uzeA 427 432 5.281 -71.10 -33.10 -69.80 -19.10 -95.20 0.40 69.60 26.40 HLHSLNLL *** HHHHTTS AAAa

1uzeA 470 475 5.010 -78.00 -45.70 -63.20 -37.40 -86.50 8.40 82.00 0.60 WRVFDGSI *** HHHHTTSS AAAa

1uzeA 538 543 5.324 -67.20 -37.90 -71.60 -29.20 -81.50 -0.40 81.30 17.70 LcQAAGHT *** HHHHTT AAAa

1uzeA 607 612 5.312 -63.80 -44.80 -69.60 -21.30 -95.90 -0.20 64.60 36.90 ENELHGEK *** HHHHTT AAAa

1v0wA 109 114 5.896 -65.40 -36.90 -71.90 -22.60 -79.00 3.10 96.90 3.50 ESAAKGNK *** HHHHTT AAAa

1v0wA 190 195 5.870 -72.90 -32.00 -84.60 -30.90 -106.70 -30.20 -117.20 -69.30 WKDDYLDT *** HHHHTSS AAAA

1v0wA 365 370 5.166 -65.10 -37.00 -62.80 -25.00 -100.00 14.90 78.00 14.80 AKMAAGVK *** HHHHTT E AAAa

1v3hA 42 47 5.485 -60.20 -47.30 -65.80 -29.30 -77.20 -7.20 95.20 2.00 QLRAAGVD *** HHHHTT AAAa

1v3hA 79 84 5.639 -63.20 -43.50 -61.30 -33.40 -84.10 7.30 67.70 34.20 LVQECGLT *** HHHHTT E AAAa

1v3hA 221 226 5.229 -65.50 -43.00 -66.10 -23.20 -85.10 -1.80 90.00 12.30 AVARAGHP *** HHHHTT T AAAa

1v3hA 370 375 5.471 -66.90 -34.90 -63.10 -35.20 -88.30 5.20 58.10 47.00 GGWREDIR *** HHHHTT AAAa

1v3hA 439 444 5.025 -69.80 -36.60 -62.40 -33.60 -85.00 13.20 55.10 43.20 VLKMHADQ *** HHHHTTT AAAa

1v4bA 160 165 4.942 -57.90 -42.40 -51.60 -40.30 -92.00 1.60 119.90 -9.60 FLGFIGIT *** HHHHTT AAAa

1v58A 122 127 5.467 -69.10 -45.50 -67.30 -24.80 -81.80 -8.60 83.10 10.70 PWVDSGKV *** HHHHTTSE AAAa

1v58A 162 167 5.058 -66.50 -38.80 -64.40 -33.50 -86.80 1.40 69.70 22.60 QYEASGGK *** HHHHTTT AAAa

1v58A 225 230 5.062 -72.90 -41.30 -58.90 -43.70 -72.80 0.60 74.70 19.10 LNIIMGN# *** HHHHTT AAAa

1v6zA 92 97 5.274 -65.80 -49.50 -68.50 -20.20 -90.10 8.10 94.20 2.00 AATELGAT *** HHHHTT S AAAa

1v6zA 129 134 4.872 -63.10 -44.80 -70.20 -24.70 -91.20 1.90 86.40 2.20 AAKQSGRV *** HHHHTT S AAAa

1v73A 90 95 5.010 -71.10 -44.60 -60.60 -34.00 -95.20 9.90 56.00 34.40 LLKKQKII *** HHHHTTSB AAAa

1v73A 136 141 4.761 -60.30 -41.90 -59.70 -32.50 -87.30 -2.30 88.20 8.20 QARDAGGM *** HHHHTT E AAAa

1v73A 170 175 5.058 -74.10 -50.50 -66.90 -43.60 -83.00 -3.20 51.50 64.60 ATTLINRP *** HHHHTTS AAAa

1v73A 214 219 5.763 -72.00 -40.30 -58.30 -37.80 -86.40 0.40 52.10 37.70 EWISRELT *** HHHHTT AAAa

1v74B 12 17 5.277 -67.20 -45.60 -63.80 -28.80 -86.50 2.80 62.00 25.70 KLFLASKI *** HHHHTTSS AAAa

1v74B 82 87 4.982 -65.60 -40.60 -54.60 -44.60 -91.20 6.70 52.10 48.50 ILEKFKL# *** HHHHTT AAAa

1v7cA 72 77 5.308 -63.30 -38.80 -58.50 -36.00 -81.80 -2.60 94.20 4.20 KAVEGGAQ *** HHHHTT S AAAa

1v7cA 120 125 5.446 -62.80 -63.30 -59.60 -38.00 -70.00 -6.30 80.20 24.50 QSLVHGAR *** HHHHTT E AAAa

1v7cA 202 207 5.209 -71.40 -38.50 -67.70 -33.00 -78.50 -10.60 82.60 7.60 AYHALGKA *** HHHHTTS AAAa

1v7cA 224 229 5.180 -63.40 -32.40 -74.00 -44.90 -77.30 -24.40 96.10 5.20 APLVLGRP *** HHHHTS AAAa

1v7cA 300 305 4.907 -64.00 -54.00 -60.10 -25.60 -94.30 4.90 62.00 32.50 KLLREGRL *** HHHHTT S AAAa

1v7cA 345 350 5.418 -65.60 -48.40 -60.50 -31.40 -83.20 0.40 64.80 28.00 VAAAAGLL *** HHHHTT AAAa

1v7wA 583 588 5.682 -87.10 -45.10 -56.80 -40.50 -68.20 -23.40 92.70 3.60 LAVLSGLA *** HHHHTT S AAAa

1v7wA 653 658 5.292 -68.50 -34.60 -69.50 -25.90 -87.20 -6.50 78.00 22.90 AETKLGRG *** HHHHTT H AAAa

1v7zA 138 143 5.014 -62.10 -34.10 -57.10 -28.80 -100.50 15.60 70.90 20.30 ELRYAGIQ *** HHHHTT AAAa

1v82A 301 306 5.407 53.70 53.80 57.70 41.10 61.00 45.90 -120.30 -2.50 KAANCTKI *** HHHHTS rrrA

1v8aA 155 160 5.101 -74.10 -40.60 -58.60 -47.70 -97.00 8.50 45.50 54.40 AAREFNTT *** HHHHTTSE AAAa

1v93A 67 72 5.304 -66.60 -39.00 -69.80 -17.00 -92.10 3.50 80.60 21.00 RIQSLGLN *** HHHHTT AAAa

1v93A 95 100 5.290 -56.10 -41.70 -64.50 -22.60 -92.90 7.40 93.90 5.90 RFVESGVE *** HHHHTT AAAa

1v93A 195 200 5.308 -61.10 -36.80 -65.00 -33.00 -82.60 4.70 84.60 15.90 RARRAGIG *** HHHHTT AAAa

1v93A 261 266 5.929 -61.30 -45.10 -65.10 -27.80 -77.00 -4.90 84.70 24.60 ELLEAGVE *** HHHHTT S AAAa

1v93A 284 289 5.446 -66.30 -39.10 -69.60 -20.40 -82.70 -8.70 108.20 -5.30 VLERLGLR *** HHHHTT AAAa

1va0A 100 105 5.125 -61.30 -40.60 -63.10 -23.20 -101.20 17.10 67.40 22.70 FLLRHGVP *** HHHHTT AAAa

1va0A 172 177 5.507 -71.20 -39.90 -63.20 -34.20 -85.40 7.70 104.90 -7.20 ELLRLGRD *** HHHHTT AAAa

1va0A 203 208 5.001 -69.20 -48.90 -56.80 -34.10 -83.10 -2.10 76.00 15.80 EEVAEGKV *** HHHHTT AAAa

1vc4A 74 79 5.472 -58.30 -53.30 -58.40 -28.80 -82.80 -6.60 112.50 5.90 AYARGGAR *** HHHHTT S AAAa

1vc4A 124 129 5.433 -66.10 -41.80 -60.90 -28.40 -104.40 21.50 94.80 6.20 EARAFGAS *** HHHHTT S AAAa

1vc4A 202 207 5.302 -64.70 -40.50 -64.50 -34.80 -79.00 4.50 93.70 5.70 LARKRGFG *** HHHHTT AAAa

1vdkA 60 65 4.616 -66.20 -45.80 -61.70 -33.90 -91.00 2.30 78.30 14.00 ANLELGEL *** HHHHTTSS AAAa

1vdkA 80 85 5.480 -67.60 -44.20 -58.10 -35.90 -78.50 -0.10 79.10 13.30 EEVVQGKW *** HHHHTTTT AAAa

1vdkA 116 121 4.819 -62.00 -42.00 -61.10 -29.20 -92.30 18.40 68.70 21.00 ASEILGKP *** HHHHTT AAAa

1vfjA 20 25 5.215 -62.30 -40.70 -59.40 -24.50 -96.10 13.30 70.80 19.60 ALFQAEVR *** HHHHTT AAAa

1vfjA 44 49 6.022 -68.20 -48.30 -61.90 -28.70 -75.70 -4.50 69.30 -1.00 VETYRGTT *** HHHHTTS AAAa

1vh5A 21 26 5.305 -65.30 -45.60 -55.50 -41.30 -89.90 0.50 60.00 38.20 MVGFLDIR *** HHHHTT E AAAa

1vhh_ 166 171 5.926 -68.60 -45.20 -68.70 -19.20 -80.40 -12.40 103.60 7.20 LAVEAGFD *** HHHHTT S AAAa

1vhnA 41 46 5.302 -64.10 -44.90 -67.10 -25.90 -86.50 -0.30 58.70 42.80 KGFLXNSQ *** HHHHTT H AAAa

1vhnA 98 103 5.311 -64.00 -39.30 -60.60 -32.40 -78.40 -11.10 88.50 14.10 KVVKEGAG *** HHHHTT G AAAa

1vhnA 149 154 5.186 -61.30 -47.00 -63.10 -34.60 -85.60 5.30 84.30 19.80 ILVEEGVD *** HHHHTT AAAa

1vhwA 118 123 4.110 -62.10 -39.90 -76.00 -34.60 -112.20 43.70 50.10 37.40 NRIRFKDH *** HHHHTTTS AADa

1vhwA 142 147 5.102 -60.40 -38.60 -66.50 -23.40 -96.10 0.60 77.70 26.90 AAKARGID *** HHHHTT AAAa

1vhwA 171 176 5.308 -58.70 -51.50 -56.10 -37.70 -90.40 11.80 73.40 22.10 VMDKYGIV *** HHHHTT AAAa

1vi9A 26 31 5.850 -67.80 -32.40 -65.30 -27.70 -83.30 -8.70 101.50 10.90 PXRRLGAN *** HHHHTT E AAAa

1vi9A 67 72 5.869 -70.70 -34.60 -65.30 -28.20 -94.30 11.60 60.60 44.00 GIAAIDKL *** HHHHTT G AAAa

1vi9A 233 238 5.344 -64.70 -45.50 -61.30 -35.40 -75.20 2.30 87.80 14.80 VKLLQGAT *** HHHHTT AAAa

1vi9A 258 263 5.372 -63.20 -46.30 -61.80 -36.40 -86.10 6.40 51.00 46.80 TTKAXQEY *** HHHHTT S AAAa

1vimA 56 61 5.515 -62.50 -44.00 -57.30 -35.20 -77.00 -8.50 99.50 9.40 RLMHLGYT *** HHHHTT AAAa

1vioA 21 26 5.442 -65.70 -44.50 -65.10 -27.50 -89.60 19.90 61.30 27.60 KAIRQSAV *** HHHHTT E AAAa

1vioA 190 195 5.244 -64.70 -39.80 -63.90 -29.40 -83.00 -2.70 93.50 9.80 MFAALGNK *** HHHHTT AAAa

1vjoA 116 121 5.537 -61.70 -34.40 -68.80 -21.30 -96.60 14.80 83.60 15.00 XAGRYGAD *** HHHHTT E AAAa

1vjoA 299 304 5.311 -54.20 -44.90 -50.90 -35.20 -102.40 14.90 79.40 23.70 RLEDIGLS *** HHHHTT AAAa

1vjpA 22 27 4.934 -65.30 -49.90 -65.40 -24.10 -96.50 12.70 51.50 23.70 EKLRKGEI *** HHHHTTSS AAAa

1vjpA 148 153 5.016 -69.30 -48.80 -57.40 -38.90 -90.40 7.90 55.60 44.60 KAIENNDK *** HHHHTT T AAAa

1vjpA 197 202 4.588 -61.80 -37.10 -77.20 -15.20 -121.80 20.10 61.70 40.30 LAKENNLV *** HHHHTTEE AAAa

1vjpA 224 229 5.247 -59.10 -44.70 -54.00 -37.90 -93.60 7.40 55.10 41.50 HLAQRNRY *** HHHHTT E AAAa

1vjpA 334 339 5.329 -62.80 -49.20 -54.80 -36.40 -83.50 -0.80 54.40 40.40 IALDRKEF *** HHHHTT AAAa

1vjpA 372 377 5.272 -62.80 -43.40 -57.40 -37.70 -79.10 -4.30 79.80 26.20 MRIWAGLK *** HHHHTT AAAa

1vjuA 263 268 4.786 -71.70 -29.60 -79.80 -23.60 -122.70 19.70 99.00 4.00 FGLQSGGR *** HHHHTT AAAa

1vk1A 117 122 5.616 -64.90 -34.70 -63.50 -22.40 -91.10 6.50 90.80 11.30 RLKAEGLE *** HHHHTT AAAa

1vk1A 133 138 5.211 -61.60 -44.90 -67.40 -21.60 -89.10 -1.50 75.80 23.50 EXAEXGEI *** HHHHTTSS AAAa

1vk1A 168 173 5.149 -60.60 -52.30 -61.40 -36.10 -80.10 -4.90 61.70 25.70 EMDQAXEI *** HHHHTTSS AAAa

1vk1A 188 193 5.128 -65.50 -41.30 -58.60 -43.30 -73.90 -9.70 81.20 15.60 ADMEKGEI *** HHHHTTS AAAa

1vk1A 211 216 5.396 -66.10 -32.00 -69.10 -21.80 -101.10 9.00 80.20 17.40 ELVKRGEV *** HHHHTT AAAa

1vk4A 34 39 5.479 -63.60 -48.70 -65.10 -32.20 -72.70 -2.30 99.60 11.90 TSSLLGVK *** HHHHTT E AAAa

1vk4A 193 198 6.291 -58.50 -41.80 -61.30 -29.80 -73.60 -6.70 119.70 -4.90 IIRSFGAK *** HHHHTT S AAAa

1vkbA 79 84 5.272 -72.00 -44.60 -55.50 -44.60 -82.90 6.60 48.90 50.10 LDDFEDCP *** HHHHTTTT AAAa

1vkeA 54 59 5.526 -75.70 -46.70 -63.10 -30.50 -89.60 -0.40 56.60 51.10 ASTVLRaD *** HHHHTT H AAAa

1vkeA 70 75 5.461 -63.90 -45.80 -55.30 -34.60 -92.90 10.80 86.60 15.00 RCVQEGAS *** HHHHTT AAAa

1vkeA 112 117 4.957 -55.20 -43.50 -60.90 -34.20 -94.90 4.00 75.60 19.80 EMEKNGET *** HHHHTT AAAa

1vkfA 40 45 5.883 -68.20 -34.10 -62.30 -30.90 -78.70 2.50 81.10 17.00 ILKDRGKT *** HHHHTT E AAAa

1vkfA 86 91 5.001 -56.90 -46.00 -56.80 -30.90 -97.00 7.50 74.60 24.90 VAKKNGIP *** HHHHTT AAAa

1vkhA 226 231 5.270 -61.90 -43.80 -64.20 -32.70 -96.70 3.30 56.20 46.20 CLQDYQLS *** HHHHTT AAAa

1vkiA 155 160 5.163 -55.90 -43.50 -61.80 -31.90 -80.00 3.70 89.30 16.90 FLEATGHA *** HHHHTT AAAa

1vkkA 112 117 5.569 -65.40 -43.50 -56.10 -43.90 -81.50 -6.20 59.10 32.70 LVQTAELT *** HHHHTT AAAa

1vkmA 159 164 5.324 -65.80 -50.70 -50.90 -44.90 -75.70 -1.30 67.00 37.60 XLETLEIP *** HHHHTT AAAa

1vkmA 202 207 5.029 -61.80 -44.70 -63.50 -36.50 -94.40 12.30 56.20 44.10 SXKEXELE *** HHHHTT AAAa

1vkmA 253 258 4.730 -67.10 -44.30 -69.80 -27.60 -105.60 13.00 51.30 41.00 LVEXTNGR *** HHHHTTTH AAAa

1vknA 157 162 4.999 -76.20 -40.20 -52.90 -40.60 -104.80 18.50 39.10 54.50 PALKHNLV *** HHHHTT S AAAa

1vknA 321 326 4.841 -62.80 -45.80 -62.90 -30.90 -99.40 4.20 71.80 26.50 XNIXFGLD *** HHHHTT AAAa

1vkyA 318 323 5.233 -60.80 -51.90 -49.60 -42.80 -89.80 5.10 59.70 42.00 EAVKRRYR *** HHHHTT AAAa

1vl1A 196 201 5.201 -60.50 -41.40 -63.40 -22.70 -102.20 23.60 65.50 30.50 TEILKDTP *** HHHHTT AAAa

1vl5A 84 89 5.901 -73.70 -26.70 -55.40 -33.00 -92.10 8.60 81.40 19.80 FIEGNGHQ *** HHHHTT AAAa

1vl5A 205 210 5.579 -68.80 -24.80 -63.20 -30.80 -95.00 11.60 52.40 41.70 WCDRXNVT *** HHHHTT AAAa

1vl5A 230 235 4.662 -69.20 -45.00 -57.80 -49.30 -101.90 13.20 45.30 47.10 YYQKFKIV *** HHHHTT E AAAa

1vlaA 58 63 5.716 -60.60 -45.10 -60.60 -44.60 -75.70 2.40 65.00 36.40 ILRKXKVI *** HHHHTT G AAAa

1vlpA 167 172 5.088 -61.40 -40.20 -60.00 -27.40 -95.10 11.40 60.40 29.80 TLFDNGIR *** HHHHTT AAAa

1vlpA 342 347 4.853 -61.70 -44.90 -55.30 -36.80 -103.80 18.20 65.60 35.90 AAKENGXL *** HHHHTT E AAAa

1vlpA 409 414 5.147 -69.70 -51.30 -58.20 -34.70 -90.80 4.80 74.70 14.30 VKEELGYT *** HHHHTT AAAa

1vls_ 140 145 4.744 -68.90 -27.10 -70.30 -41.70 -97.60 14.10 67.30 16.30 QFLDNGNM *** HHHHTT H AAAa

1vm0A 68 73 5.140 -73.80 -57.70 -57.80 -29.90 -97.60 8.90 83.10 12.90 ILKNNGFA *** HHHHTTSE AAAa

1vmeA 278 283 5.349 -65.20 -41.80 -57.90 -29.70 -96.60 6.70 96.20 3.10 SLKEKGFT *** HHHHTT E AAAa

1vns_ 490 495 5.558 -73.40 -44.60 -63.40 -33.40 -78.50 -16.50 78.10 16.70 SRIFLGVH *** HHHHTTSS AAAa

1vns_ 563 568 5.494 -61.30 -49.80 -54.10 -35.70 -87.50 3.40 65.10 31.70 EIFNNGLK *** HHHHTTS AAAa

1vp4A 167 172 5.023 -62.90 -47.40 -63.90 -30.30 -93.00 14.30 53.70 38.30 EFDKNGKI *** HHHHTT G AAAa

1vp4A 205 210 4.597 -61.10 -50.00 -54.70 -44.30 -101.30 15.80 51.60 49.80 IAEKYDLF *** HHHHTT E AAAa

1vp4A 407 412 4.827 -75.80 -36.70 -61.20 -35.00 -90.80 0.10 60.80 35.90 YGKEKHLL *** HHHHTT AAAa

1vp8A 82 87 5.496 -66.00 -40.60 -58.90 -34.70 -85.20 2.80 97.70 10.70 ELRKRGAK *** HHHHTT E AAAa

1vp8A 138 143 5.178 -65.60 -48.00 -64.70 -32.30 -80.10 -4.50 80.00 19.40 XAADSGAI *** HHHHTTSS AAAa

1vpbA 17 22 5.673 -68.90 -31.80 -82.30 -14.30 -121.60 23.90 135.60 -14.60 YALKNGCQ *** HHHHTT S AAAa

1vpbA 259 264 5.109 -66.60 -42.00 -67.90 -36.20 -88.60 15.30 44.20 43.30 SALQQKNS *** HHHHTT AAAa

1vpdA 21 26 5.652 -63.00 -42.90 -63.70 -31.30 -79.50 -2.50 97.90 7.70 NLLKAGYS *** HHHHTT E AAAa

1vpdA 41 46 5.870 -65.20 -35.10 -62.90 -26.80 -84.80 -2.80 126.50 -5.60 DVIAAGAE *** HHHHTT E AAAa

1vpdA 193 198 5.375 -62.70 -46.80 -62.20 -32.20 -83.80 6.00 92.80 14.80 LATKAGVN *** HHHHTT AAAa

1vpdA 223 228 4.884 -57.00 -49.50 -69.60 -30.10 -86.40 -0.40 62.10 34.60 PXVXDRNF *** HHHHTT AAAa

1vpdA 269 274 5.054 -59.10 -44.40 -65.00 -25.90 -100.30 20.30 84.10 16.40 ALRADGHG *** HHHHTT T AAAa

1vprA 1187 1192 4.780 -61.80 -55.30 -58.10 -38.90 -97.00 9.00 47.80 43.50 DYTALAGR *** HHHHTTT AAAa

1vquA 21 26 5.431 -69.10 -43.90 -56.10 -39.20 -71.80 -0.40 93.70 -5.00 QQLIDGES *** HHHHTT AAAa

1vquA 39 44 4.876 -60.30 -47.10 -60.60 -33.80 -82.90 -3.80 55.60 35.00 QGWLSEAV *** HHHHTT S AAAa

1vquA 122 127 5.561 -67.00 -33.60 -63.00 -27.70 -80.70 -7.50 102.60 2.10 VAAAYGVP *** HHHHTT AAAa

1vquA 147 152 5.165 -59.40 -37.80 -72.80 -19.90 -97.00 -2.10 109.30 10.50 VLEALGVN *** HHHHTT AAAa

1vquA 230 235 5.601 -58.30 -51.20 -54.00 -36.30 -83.60 -0.60 94.90 13.80 ALDNLGKQ *** HHHHTT S AAAa

1vquA 300 305 4.946 -69.40 -42.40 -65.80 -25.60 -86.00 8.00 87.40 15.70 KAVLQGKG *** HHHHTT S AAAa

1vqzA 109 114 5.101 -64.70 -36.80 -55.70 -35.60 -86.40 5.20 84.00 21.60 TLAQLGVK *** HHHHTT AAAa

1vqzA 236 241 6.067 -62.50 -35.30 -81.70 -49.80 -72.30 -37.60 -109.10 -91.60 TWDWNYGK *** HHHHT AAAX

1vr5A 355 360 5.304 -62.30 -47.50 -59.80 -34.60 -76.60 -10.90 84.90 13.80 ILDELGFK *** HHHHTT B AAAa

1vsrA 32 37 5.876 -63.00 -43.80 -62.60 -29.40 -78.50 -4.00 86.30 18.80 LLTGQGLA *** HHHHTT AAAa

1vsrA 104 109 5.430 -62.70 -37.10 -64.60 -31.00 -79.70 -5.40 98.30 10.20 RLQELGWR *** HHHHTT E AAAa

1vybA 172 177 5.020 -65.60 -39.20 -61.70 -32.80 -89.70 8.50 59.20 39.90 ALHQADLI *** HHHHTTEE AAAa

1vyiA 250 255 5.021 -64.60 -39.30 -59.00 -41.60 -78.50 -6.00 75.00 22.60 RLAHDGSK *** HHHHTT AAAa

1vyrA 90 95 5.172 -65.50 -32.10 -63.40 -23.90 -98.30 12.60 66.90 29.70 GVHAEDGR *** HHHHTT AAAa

1vyrA 170 175 5.764 -60.60 -46.80 -60.70 -36.90 -74.10 -5.50 92.60 8.30 NAREAGFD *** HHHHTT S AAAa

1vyrA 260 265 7.143 -60.00 -34.40 -60.80 -27.20 -84.70 -9.50 66.10 37.00 ELAKRGIA *** HHHHTT S AAAa

1vyrA 311 316 5.591 -64.50 -42.40 -65.70 -31.10 -78.50 -6.90 78.10 20.80 DLIGKGLI *** HHHHTTS AAAa

1w0hA 281 286 4.888 -59.60 -42.10 -60.10 -34.30 -91.70 6.60 76.30 25.10 XLEKLGXD *** HHHHTT AAAa

1w0hA 309 314 5.360 -65.30 -45.30 -57.10 -37.00 -76.90 -4.20 97.60 10.00 RXLQDGCE *** HHHHTT AAAa

1w1oA 319 324 5.007 -64.50 -46.40 -66.50 -23.90 -102.10 -1.60 50.40 42.30 LAGERNAT *** HHHHTT S AAAa

1w1oA 385 390 4.941 -66.20 -31.90 -66.10 -31.70 -90.30 -0.10 71.00 29.30 ALNKLGLW *** HHHHTT S AAAa

1w1oA 481 486 5.270 -66.80 -43.30 -69.10 -32.20 -79.80 -9.10 70.80 29.00 FCDLAGIQ *** HHHHTT AAAa

1w23A 229 234 4.598 -61.60 -50.80 -64.70 -35.90 -92.60 6.40 52.90 42.40 THIKSDSL *** HHHHTTT AAAa

1w23A 254 259 5.302 -60.60 -46.10 -58.70 -34.90 -84.40 -1.20 114.30 13.00 WIKDLGGA *** HHHHTTHH AAAa

1w23A 316 321 5.198 -61.60 -42.60 -58.60 -37.60 -85.30 4.10 97.20 6.00 KAKEQGFV *** HHHHTTEE AAAa

1w2wA 44 49 4.804 -73.00 -49.90 -63.50 -39.30 -82.00 -7.40 68.10 27.50 SVIKSXQV *** HHHHTTSS AAAa

1w2wA 166 171 5.216 -63.60 -39.90 -60.80 -40.00 -77.90 -0.90 69.00 22.50 VLQKDGFK *** HHHHTT AAAa

1w2yA 158 163 5.380 -61.00 -46.50 -61.60 -33.10 -80.10 1.00 90.30 17.40 LAIKCGLN *** HHHHTT AAAa

1w4rA 43 48 5.418 -59.90 -44.80 -67.50 -21.70 -90.50 14.20 76.30 18.30 RFQIAQYK *** HHHHTT AAAa

1w4rA 112 117 5.453 -66.30 -35.40 -74.40 -16.70 -93.30 8.30 102.20 1.00 AMANAGKT *** HHHHTT E AAAa

1w53A 37 42 5.233 -67.30 -39.40 -67.80 -16.10 -106.10 17.70 58.50 35.20 KTIEHQIP *** HHHHTT AAAa

1w6sA 204 209 6.854 -58.10 -38.90 -74.70 -28.70 -70.10 -20.70 55.60 65.40 PDKDLLLA *** HHHHTB AAAa

1w78A 71 76 5.485 -67.40 -42.30 -60.20 -34.40 -79.40 2.30 82.80 9.20 ILMAAGYK *** HHHHTT AAAa

1w85I 139 144 4.780 -63.60 -50.70 -51.90 -22.20 -121.60 23.50 53.10 29.30 YAREKGVD *** HHHHTT AAAa

1w96A 50 55 5.002 -59.90 -53.10 -60.50 -32.50 -84.10 8.70 85.30 8.00 FVKSHGGH *** HHHHTT AAAa

1w96A 102 107 5.360 -61.70 -45.10 -66.20 -25.40 -91.90 -0.20 55.70 45.70 EDLEANAE *** HHHHTT H AAAa

1w96A 138 143 5.457 -71.30 -45.70 -60.70 -38.90 -86.10 -2.70 52.40 48.10 IAERADVD *** HHHHTT S AAAa

1w96A 193 198 5.346 -63.70 -30.90 -71.30 -21.90 -92.90 11.00 60.60 38.30 VAQSAKVP *** HHHHTT AAAa

1w96A 405 410 5.540 -63.40 -39.30 -66.80 -22.50 -86.10 -2.60 92.70 10.40 LQIAMGIP *** HHHHTT AAAa

1w96A 419 424 5.032 -61.30 -41.40 -60.20 -33.50 -94.40 14.40 64.20 28.40 IRTLYGMN *** HHHHTT AAAa

1w96A 551 556 5.004 -64.60 -46.40 -57.80 -43.20 -82.80 -3.00 60.60 42.70 EDFEDNTI *** HHHHTT AAAa

1w9aA 124 129 5.221 -62.70 -48.40 -59.40 -32.70 -92.00 4.50 49.50 46.50 AXVTDRRV *** HHHHTTEE AAAa

1w9hA 161 166 5.311 -64.00 -41.70 -65.90 -27.60 -82.00 -0.50 97.40 4.80 FVSKLGGK *** HHHHTT B AAAa

1w9hA 275 280 5.187 -60.50 -31.40 -78.00 -40.80 -69.80 13.80 43.90 36.20 ELKKQEMV *** HHHHTTSS AAAa

1wc1A 1048 1053 5.028 -59.00 -42.60 -65.30 -17.10 -107.20 18.50 61.30 25.60 AVFENQGT *** HHHHTT E AAAa

1wc1A 1100 1105 4.966 -64.30 -45.70 -63.60 -30.90 -90.00 10.00 52.50 39.90 GWQERGLV *** HHHHTTSS AAAa

1wd3A 24 29 5.357 -61.30 -43.80 -67.50 -21.70 -92.00 8.20 68.00 27.50 IYEAGDTP *** HHHHTT AAAa

1wdcC 37 42 6.107 -70.20 -30.50 -67.60 -22.20 -90.70 4.50 100.60 1.50 VCRCLGIN *** HHHHTT AAAa

1wdcC 49 54 5.287 -62.40 -24.20 -79.90 -27.90 -97.90 25.10 98.40 4.60 DVFAVGGT *** HHHHTT AAAa

1wddA 192 197 6.373 -68.40 -34.20 -71.30 -17.60 -83.60 -7.00 129.40 -4.50 ECLRGGLD *** HHHHTT S AAAa

1wddA 430 435 5.355 -56.30 -47.80 -64.60 -31.30 -79.30 -2.80 100.20 11.80 QARNEGRD *** HHHHTT AAAa

1wddS 33 38 5.450 -69.90 -39.30 -67.50 -29.20 -77.60 -4.40 73.20 30.20 YLLRSKWV *** HHHHTT E AAAa

1wdeA 107 112 5.092 -53.20 -46.00 -68.70 -27.40 -87.40 15.80 88.50 -20.60 EALEAGVA *** HHHHTT E AAAa

1wdeA 262 267 4.572 -64.70 -42.90 -63.30 -38.10 -93.30 3.60 63.00 44.40 LAAASGQR *** HHHHTT AAAa

1wdjA 134 139 5.453 -62.90 -42.90 -67.70 -18.50 -94.30 0.10 116.10 -5.50 IYLRNGVL *** HHHHTT S AAAa

1wehA 29 34 5.368 -67.00 -40.70 -63.70 -33.80 -78.70 3.50 90.60 11.10 VLAEEGFG *** HHHHTTEE AAAa

1wehA 52 57 4.950 -66.60 -31.40 -62.70 -27.50 -103.70 16.40 75.20 12.70 GVKAKGGL *** HHHHTT AAAa

1whi_ 106 111 5.604 -58.60 -40.00 -66.40 -26.50 -96.30 13.90 68.70 29.10 ELRDKDFM *** HHHHTT H AAAa

1whsB 285 290 4.696 -64.90 -54.20 -57.90 -20.80 -92.60 -14.60 72.30 28.90 VQMALHAN *** HHHHTT S AAAa

1whsB 320 328 5.598 -65.60 -41.10 -69.30 -36.70 -65.00 -11.20 83.50 15.10 ELIAAGLR *** HHHHTT E AAAa

1whzA 14 19 5.459 -61.60 -29.70 -70.00 -25.90 -81.00 -10.60 108.10 6.20 KLRRLGFV *** HHHHTT E AAAa

1whzA 57 62 5.045 -56.60 -52.40 -69.30 -30.00 -78.90 -5.90 68.30 34.10 ILRDAGLT *** HHHHTT AAAa

1wkqA 17 22 5.037 -60.80 -52.50 -69.60 -23.30 -91.20 5.50 73.90 13.30 EGVNAGIG *** HHHHTTSS AAAa

1wkqA 44 49 4.836 -80.30 -20.70 -88.50 -22.50 -103.20 1.20 51.00 51.30 NVTTSNDP *** HHHHT T AAAa

1wkqA 107 112 5.394 -63.40 -42.90 -63.50 -27.60 -83.60 2.30 101.80 6.70 DAAEAGFD *** HHHHTT S AAAa

1wojA 334 339 5.435 -67.20 -41.20 -64.80 -31.20 -78.10 -8.20 108.50 5.40 RQEKGGSR *** HHHHTT AAAa

1woqA 188 193 5.276 -71.20 -44.10 -57.70 -38.90 -92.00 8.80 58.50 37.80 ARERDGLS *** HHHHTT AAAa

1wp5A 25 30 5.162 -60.00 -42.30 -63.70 -20.30 -112.00 19.50 52.00 42.80 SYAASNGQ *** HHHHTTT AAAa

1wpcA 92 97 5.046 -61.00 -40.30 -69.80 -17.80 -103.30 12.40 68.10 33.70 SLKNNGIQ *** HHHHTT E AAAa

1wpcA 279 284 5.008 -65.00 -42.80 -67.60 -24.60 -98.20 12.60 59.90 30.70 YLQKTNWN *** HHHHTTT AAAa

1wpnA 27 32 5.362 -63.40 -41.40 -57.80 -32.10 -88.40 7.30 93.40 13.80 LKNKLGFN *** HHHHTT AAAa

1wpnA 50 55 4.886 -71.50 -39.30 -64.50 -42.20 -98.20 15.50 53.90 50.80 ALDYFKQE *** HHHHTT AAAa

1wpnA 127 132 5.172 -69.20 -39.10 -60.40 -31.70 -98.30 4.30 51.60 40.70 MYKENNVK *** HHHHTT AAAa

1wqjB 71 76 4.993 -66.20 -39.00 -56.00 -36.80 -95.00 12.50 72.80 25.40 HTLMHGQG *** HHHHTT E AAAa

1wteA 24 29 5.391 -83.00 -47.90 -67.00 -35.10 -87.40 0.20 57.50 48.50 SKLTKLMS *** HHHHTT AAAa

1wteA 44 49 5.170 -80.90 -36.20 -62.80 -34.70 -103.90 13.40 54.10 49.10 IFDYHSLN *** HHHHTT AAAa

1wteA 205 210 4.572 -53.50 -46.80 -55.00 -46.60 -107.00 46.50 53.90 39.20 WSWLNNGE *** HHHHTTT AADa

1wu4A 18 23 5.538 -63.70 -43.40 -64.50 -24.60 -93.80 13.00 105.30 -12.20 LFKEFGYS *** HHHHTT AAAa

1wu4A 79 84 5.073 -69.50 -34.30 -65.20 -31.90 -91.40 3.30 58.40 48.00 MAVQMDRK *** HHHHTT H AAAa

1wu4A 370 375 4.670 -64.00 -45.50 -69.70 -17.00 -99.90 1.30 79.50 25.50 MLALSGNF *** HHHHTT AAAa

1wvfA 141 146 5.089 -68.80 -46.00 -65.90 -29.40 -97.00 7.90 51.30 44.40 YIQENNLP *** HHHHTT S AAAa

1wvfA 164 169 5.093 -70.50 -32.20 -65.70 -17.00 -109.50 8.40 54.80 47.80 GNTMDRGV *** HHHHTT B AAAa

1wvfA 268 273 5.135 -75.80 -41.70 -62.50 -33.50 -89.00 -3.40 69.70 11.20 PLRMSNTI *** HHHHTTSS AAAa

1wvfA 287 292 5.304 -64.10 -36.70 -68.60 -23.20 -89.20 8.00 69.10 28.20 EAGSAHLT *** HHHHTT AAAa

1wvfA 370 375 4.743 -57.30 -42.50 -74.00 -6.70 -120.00 16.60 68.10 31.70 AQLMSGVP *** HHHHTT AAAa

1wvfA 417 422 4.913 -60.90 -37.40 -54.30 -36.20 -98.90 15.60 74.70 18.10 VLHKYGLD *** HHHHTT AAAa

1wvfA 465 470 4.986 -60.20 -35.50 -60.40 -25.00 -103.70 13.10 78.40 24.50 EFEKEGYA *** HHHHTT AAAa

1x6iA 56 61 4.884 -70.80 -42.90 -67.10 -25.70 -91.80 5.50 62.40 25.80 FNWLMNHG *** HHHHTTSS AAAa

1x6oA 137 142 5.771 -59.30 -44.10 -67.60 -25.10 -77.10 -4.50 83.10 32.70 EQFDSGKD *** HHHHTT AAAa

1x74A 26 31 5.308 -60.10 -40.40 -55.40 -43.60 -83.80 4.20 117.30 -5.30 ILGDLGAD *** HHHHTT E AAAa

1x74A 202 207 4.640 -54.20 -39.50 -66.00 -24.10 -111.80 15.30 79.80 8.10 AMRATGMW *** HHHHTTS AAAa

1x74A 313 318 5.630 -60.10 -49.80 -70.50 -27.80 -92.90 -2.00 51.90 48.40 HIIERNTF *** HHHHTT E AAAa

1x7dA 273 278 5.320 -69.70 -44.20 -64.50 -29.80 -82.00 -11.20 77.90 21.50 WRVLRGET *** HHHHTTSS AAAa

1x7yA 77 82 5.163 -66.60 -43.30 -57.90 -32.00 -86.30 -3.70 77.10 12.40 ESQRQGRI *** HHHHTTSS AAAa

1x7yA 119 124 5.304 -62.30 -42.60 -63.00 -37.80 -78.30 16.30 64.40 32.30 VLMYRDYP *** HHHHTT AAAa

1x7yA 208 213 5.007 -77.00 -51.40 -71.30 -26.40 -99.20 3.60 54.80 69.70 FAATLECP *** HHHHTT AAAa

1x7yB 86 91 5.883 -66.10 -29.90 -57.70 -28.60 -85.30 -4.00 97.30 -3.70 GIAVTGAT *** HHHHTT AAAa

1x7yB 118 123 4.834 -58.50 -52.40 -69.40 -17.40 -97.50 1.80 61.00 29.90 YRYRSGDL *** HHHHTTTS AAAa

1x8qA 153 158 5.449 -58.60 -41.20 -67.30 -23.10 -81.90 3.00 60.00 48.00 AVSAATLE *** HHHHTT AAAa

1x91A 21 26 4.928 -71.80 -42.30 -61.00 -34.20 -105.50 18.70 33.30 68.40 FLNTKFAS *** HHHHTT AAAa

1x91A 88 93 5.212 -54.60 -39.60 -63.70 -28.30 -88.40 4.20 74.10 12.70 EHLASGDG *** HHHHTT H AAAa

1x99A 108 113 7.760 -57.40 -44.60 -67.30 -20.40 -71.20 -18.70 54.40 50.80 YVREKQLA *** HHHHTT S AAAa

1x9dA 298 303 4.913 -59.30 -51.60 -62.70 -39.60 -75.50 -5.30 81.80 18.90 TMWILGLR *** HHHHTT H AAAa

1x9dA 473 478 5.085 -62.70 -49.40 -53.80 -43.50 -81.50 10.20 78.00 26.70 QWIQGGKQ *** HHHHTTS AAAa

1x9dA 536 541 5.157 -61.00 -42.60 -52.70 -37.40 -92.10 9.10 111.50 2.00 LGVYHGLP *** HHHHTT AAAa

1x9iA 59 64 4.802 -75.10 -46.40 -63.20 -44.80 -100.60 10.80 54.00 48.50 FSLTWNWE *** HHHHTT S AAAa

1xbbA 485 490 5.569 -64.40 -46.90 -57.30 -35.70 -80.10 -7.30 57.70 45.80 YLEESNFV *** HHHHTTEE AAAa

1xbbA 564 569 5.026 -67.60 -45.20 -74.70 -14.40 -94.40 19.90 46.20 47.30 WEAFSYGQ *** HHHHTTT AAAa

1xbbA 584 589 5.418 -64.60 -38.20 -63.20 -35.60 -75.60 4.10 80.20 8.20 AMLEKGER *** HHHHTT AAAa

1xd3A 20 25 5.514 -59.60 -46.40 -65.10 -25.00 -90.10 7.10 88.70 17.00 FLKQLGLH *** HHHHTTB AAAa

1xdnA 72 77 5.187 -69.30 -47.70 -62.90 -37.60 -71.60 -14.80 89.80 -7.80 SLHKSGLA *** HHHHTTGG AAAa

1xdnA 267 272 5.021 -66.20 -38.00 -64.50 -27.60 -102.50 24.00 94.10 6.50 LPALLGLG *** HHHHTT T AAAa

1xdwA 88 93 5.346 -61.90 -49.00 -50.40 -40.40 -82.20 -4.40 85.20 14.80 YAKELGFP *** HHHHTT AAAa

1xdwA 116 121 5.005 -87.30 -47.00 -58.40 -41.60 -103.10 11.30 53.20 33.20 MMLLRHTA *** HHHHTTHH AAAa

1xdwA 165 170 5.638 -59.50 -42.40 -65.70 -22.80 -84.40 -7.00 110.30 -2.10 IFHGMGAT *** HHHHTT E AAAa

1xffA 111 116 5.579 -56.90 -49.50 -56.00 -35.10 -78.90 -3.70 115.70 -0.90 ELKARGYT *** HHHHTT AAAa

1xfiA 111 116 5.531 -70.10 -39.10 -56.10 -37.70 -76.70 0.30 93.90 3.30 VLRELGFR *** HHHHTT S AAAa

1xfiA 237 242 5.319 -58.50 -47.60 -60.20 -31.00 -90.80 3.20 104.30 3.40 ELLRRGAQ *** HHHHTT E AAAa

1xfoA 151 156 5.750 -67.00 -43.30 -52.20 -33.60 -81.00 -10.80 113.80 2.20 EAEDMGVK *** HHHHTT AAAa

1xfoA 281 286 4.818 -60.80 -49.30 -57.90 -45.10 -93.60 8.10 54.00 41.20 LAKKHEIP *** HHHHTT AAAa

1xg4A 34 39 5.873 -66.70 -43.70 -65.10 -24.80 -80.80 -7.20 103.40 -2.10 LAQRAGYQ *** HHHHTT S AAAa

1xg4A 177 182 5.638 -71.90 -36.50 -66.20 -29.70 -77.20 -6.90 121.60 -7.80 AYVEAGAE *** HHHHTT S AAAa

1xg4A 225 230 5.465 -61.80 -36.70 -66.90 -21.00 -99.90 8.10 64.00 34.50 ELRSAHVA *** HHHHTT S AAAa

1xg4A 274 279 4.769 -61.50 -44.30 -58.60 -38.40 -103.00 11.00 60.20 38.90 LYESINYY *** HHHHTTHH AAAa

1xgkA 25 30 5.293 -61.90 -43.70 -59.50 -28.60 -97.20 10.00 106.30 -4.40 VAAAVGHH *** HHHHTT AAAa

1xgkA 343 348 5.380 -61.90 -37.40 -64.20 -27.30 -86.90 0.90 94.20 3.60 EEEANGLD *** HHHHTT AAAa

1xjuA 140 145 5.205 -60.40 -40.20 -65.80 -23.20 -92.30 1.00 69.00 19.80 KWAQKGEW *** HHHHTT H AAAa

1xkiA 134 139 4.888 -67.30 -36.30 -59.70 -22.60 -113.30 18.00 79.70 24.90 AAGARGLS *** HHHHTT T AAAa

1xm8A 199 204 5.187 -66.40 -29.80 -64.60 -25.60 -108.30 21.50 62.10 32.20 ELRSKKLP *** HHHHTT AAAa

1xmbA 160 165 5.446 -58.00 -46.30 -54.20 -38.20 -88.90 10.20 90.40 18.10 KMREEGAL *** HHHHTTTT AAAa

1xmbA 292 297 4.871 -58.50 -41.40 -80.50 -8.10 -103.60 22.60 44.30 55.30 QAAVHRCN *** HHHHTTEE AAAa

1xmtA 65 70 4.675 -63.10 -45.50 -61.60 -32.70 -98.30 7.30 59.20 35.20 HASSHSIS *** HHHHTT E AAAa

1xocA 339 344 5.192 -62.20 -48.00 -56.50 -34.80 -81.50 4.80 98.60 1.30 MLAEAGWK *** HHHHTT B AAAa

1xqoA 179 184 5.580 -66.70 -43.50 -61.80 -30.40 -82.80 -2.10 76.20 20.10 LTWcAGLI *** HHHHTTS AAAa

1xsvA 45 50 4.874 -75.80 -34.90 -60.90 -42.00 -105.20 6.10 52.30 57.90 IADTFNVS *** HHHHTT AAAa

1xtpA 65 70 4.623 -60.50 -45.40 -77.20 -22.40 -101.50 15.80 56.40 39.50 VSGVLGGX *** HHHHTTT AAAa

1xu9A 54 59 5.293 -65.30 -46.80 -59.00 -32.40 -81.80 -3.40 78.10 27.80 HLAKMGAH *** HHHHTT E AAAa

1xu9A 248 253 5.251 -63.20 -45.90 -61.50 -36.70 -87.30 1.00 61.40 33.50 KGGALRQE *** HHHHTT S AAAa

1xubA 222 227 5.099 -71.70 -45.70 -59.00 -38.60 -93.90 15.80 82.00 10.80 HLARHGQI *** HHHHTTSS AAAa

1xv2A 13 18 6.240 -66.30 -49.30 -56.10 -30.70 -69.60 -13.90 113.90 2.00 GTLXAGLL *** HHHHTT AAAa

1xvwA 101 106 5.248 -64.50 -43.00 -59.20 -35.40 -85.40 -3.00 71.20 26.00 VSQAYGVF *** HHHHTT E AAAa

1xx1A 149 154 4.936 -64.50 -52.10 -53.00 -41.80 -81.30 0.10 77.50 28.20 VLKKEGHE *** HHHHTT G AAAa

1xy7A 119 124 5.367 -67.70 -39.20 -60.60 -25.90 -91.50 -4.20 123.30 -4.00 KAVDAGAV *** HHHHTT E AAAa

1xy7A 133 138 5.135 -55.30 -41.30 -60.80 -27.60 -87.70 -0.10 88.30 9.80 AEVELGFK *** HHHHTTEE AAAa

1y08A 242 247 5.814 -64.60 -47.70 -60.70 -33.90 -77.70 5.60 83.20 20.10 KELTEGKA *** HHHHTT E AAAa

1y0bA 140 145 5.524 -62.30 -40.50 -58.50 -32.80 -78.80 -6.20 79.50 8.20 IVKQAGAS *** HHHHTT E AAAa

1y0bA 165 170 5.601 -62.90 -42.80 -53.90 -38.10 -79.60 2.30 92.30 10.50 ELVKLGYR *** HHHHTT AAAa

1y0eA 134 139 5.742 -63.10 -47.70 -54.60 -40.70 -74.70 -4.70 78.10 26.60 NAARLGFD *** HHHHTT S AAAa

1y0eA 192 197 6.103 -68.10 -37.50 -62.80 -34.90 -73.40 -7.10 100.70 15.30 RVXDLGVH *** HHHHTT S AAAa

1y0kA 38 43 5.128 -60.60 -40.60 -58.90 -37.30 -92.90 2.10 55.10 46.50 FLEALNVP *** HHHHTT AAAa

1y0kA 164 169 7.372 -55.20 -47.40 -62.60 -22.80 -73.10 -15.50 94.00 4.10 EYLRQGWR *** HHHHTT S AAAa

1y0uA 37 42 5.285 -60.40 -39.90 -68.30 -22.30 -92.30 1.70 77.20 18.90 RXLDKGRS *** HHHHTT AAAa

1y0uA 65 70 5.205 -66.20 -45.90 -62.30 -27.50 -96.80 18.20 69.90 17.50 VLEAGFCI *** HHHHTTSE AAAa

1y21A 68 73 5.446 -59.90 -41.10 -62.60 -28.00 -85.60 -2.80 93.60 14.50 LLTSKGYT *** HHHHTTEE AAAa

1y21A 186 191 5.261 -59.00 -49.30 -58.70 -28.50 -90.30 -1.80 57.10 34.70 SLVDQNNI *** HHHHTT H AAAa

1y21A 204 209 5.248 -64.80 -36.50 -61.00 -31.90 -87.00 14.80 62.80 29.00 KAKEQKLF *** HHHHTTSS AAAa

1y42X 58 63 5.108 -64.20 -50.50 -52.60 -41.00 -82.00 -4.40 81.00 11.40 EAIKKGKK *** HHHHTTSS AAAa

1y42X 181 186 5.630 -61.20 -40.30 -74.30 -25.00 -95.50 11.70 85.20 16.80 QMRARGYE *** HHHHTT AAAa

1y4jA 156 161 4.701 -53.50 -40.00 -66.90 -33.70 -81.00 0.70 71.80 24.70 YaAWRGKR *** HHHHTT B AAAa

1y5iC 165 170 4.874 -58.10 -52.80 -71.60 -14.20 -105.80 11.90 56.40 41.40 QSVVTFHG *** HHHHTT T AAAa

1y7rA 18 23 5.166 -61.10 -45.20 -79.70 -7.70 -89.20 0.30 79.30 25.40 LRINAGXS *** HHHHTT AAAa

1y7yA 22 27 5.731 -66.90 -31.40 -65.20 -23.70 -96.20 -2.50 66.20 41.30 LRTAKGLS *** HHHHTT AAAa

1y7yA 45 50 5.058 -68.10 -39.10 -63.30 -29.80 -87.00 -2.10 72.80 24.10 GGVERGQR *** HHHHTT S AAAa

1y7yA 62 67 5.251 -58.00 -43.80 -66.40 -36.50 -79.80 -6.20 57.70 42.50 LATALDIE *** HHHHTTS AAAa

1y80A 112 117 5.531 -62.60 -34.80 -62.70 -26.00 -94.90 4.30 106.20 -2.90 MLESGGFT *** HHHHTT E AAAa

1y80A 163 168 5.188 -60.90 -50.80 -59.30 -35.60 -77.70 -9.30 81.70 16.80 ALIAAGLR *** HHHHTT G AAAa

1y8aA 62 67 5.494 -60.90 -44.60 -58.10 -37.10 -76.70 -4.20 89.40 15.90 FLAAAGVK *** HHHHTT AAAa

1y8aA 114 119 5.135 -62.70 -29.40 -64.90 -24.60 -119.80 21.70 66.60 33.80 TASMIGVR *** HHHHTT AAAa

1y8aA 218 223 5.120 -60.00 -45.60 -61.50 -35.00 -81.80 -1.70 82.80 17.80 AARGLGGV *** HHHHTT E AAAa

1yacA 47 52 5.341 -67.60 -47.20 -56.60 -47.80 -81.50 -0.70 57.60 43.40 LAKYFNLP *** HHHHTT AAAa

1yacA 100 105 7.878 -65.30 -29.90 -67.80 -20.00 -69.00 -25.60 73.20 13.70 AVKATGKK *** HHHHTT S AAAa

1yacA 126 131 5.487 -63.30 -43.20 -61.40 -34.40 -76.50 -4.70 99.40 4.70 SAIEEGFD *** HHHHTT E AAAa

1yc9A 223 228 5.022 -57.30 -40.80 -59.70 -34.30 -94.10 14.80 72.40 13.60 KRYANGLE *** HHHHTTSS AAAa

1yc9A 263 268 5.159 -63.90 -38.80 -64.20 -26.80 -99.50 11.90 80.60 25.30 LAALVGQG *** HHHHTT AAAa

1ydgA 29 34 5.455 -59.10 -49.00 -55.10 -35.80 -81.70 3.40 88.90 15.80 AGRAAGAE *** HHHHTT E AAAa

1ydgA 105 110 5.162 -56.30 -51.90 -70.10 -19.50 -90.00 -2.20 73.20 16.30 GLWSSGKL *** HHHHTTTT AAAa

1ydgA 157 162 5.034 -56.00 -39.30 -71.80 -18.60 -105.80 14.50 109.70 -8.30 VIFKSGGN *** HHHHTT AAAa

1yge_ 273 278 5.278 -78.80 -43.30 -60.40 -34.90 -85.90 -1.50 56.70 44.90 SAFDLKST *** HHHHTT S AAAa

1yge_ 398 403 4.998 -64.70 -50.70 -63.20 -36.10 -82.40 3.90 59.60 46.90 EALGSRRL *** HHHHTT E AAAa

1yge_ 585 590 5.371 -68.40 -44.50 -58.50 -43.40 -78.60 1.20 68.20 28.00 DLIKRGVA *** HHHHTTSE AAAa

1yghA 221 226 5.278 -59.30 -40.90 -62.30 -28.00 -93.30 16.70 81.50 19.50 YFKKQGFT *** HHHHTT B AAAa

1yiiA 155 160 7.961 -60.50 -35.40 -62.30 -22.60 -71.20 -20.40 51.90 41.90 VVLLQANR *** HHHHTT AAAa

1yiiA 182 187 5.146 -65.10 -39.80 -64.00 -27.10 -93.80 22.90 52.30 32.50 RAGELKWG *** HHHHTTT AAAa

1ykdA 284 289 4.910 -68.70 -44.40 -66.20 -36.10 -94.30 1.80 55.60 45.20 AKELMNAD *** HHHHTTEE AAAa

1yleA 177 182 5.595 -59.10 -34.20 -85.70 -22.20 -121.20 -92.80 -51.90 -40.70 FWNAVGRN *** HHHHTGGG AAAA

1ym3A 59 64 5.178 -74.90 -49.10 -55.50 -39.60 -105.80 8.30 44.40 61.10 AEIIFDQG *** HHHHTT AAAa

1ym3A 141 146 5.152 -68.60 -39.10 -57.40 -34.80 -93.60 9.50 93.60 3.50 LGRRDGLS *** HHHHTT AAAa

1yn9A 73 78 5.375 -58.80 -39.30 -64.40 -31.20 -87.30 2.00 94.20 9.30 HFLRAGLL *** HHHHTT E AAAa

1yoyA 54 59 5.175 -52.40 -44.30 -68.70 -20.60 -94.80 16.30 80.60 19.90 LALKSGES *** HHHHTT AAAa

1ypqA 172 177 5.005 -56.40 -39.30 -63.60 -19.30 -99.50 15.20 61.70 37.90 KcLSLDAK *** HHHHTT E AAAa

1yqgA 97 102 4.928 -59.30 -48.50 -64.80 -27.80 -96.50 4.90 85.50 13.20 LSRYLGGT *** HHHHTTS AAAa

1yqgA 182 187 5.683 -53.20 -49.00 -56.80 -26.00 -91.80 -0.30 109.10 6.80 AAIRQGFD *** HHHHTT AAAa

1yqgA 235 240 5.314 -66.70 -43.80 -57.60 -42.90 -83.70 5.90 51.20 47.00 AFRRHRVA *** HHHHTTHH AAAa

1yqhA 65 70 5.472 -62.60 -44.30 -61.10 -31.00 -79.70 -3.90 117.50 -0.50 ACVDAGAE *** HHHHTT S AAAa

1yr0A 135 140 5.310 -62.20 -39.40 -64.80 -15.90 -108.40 14.60 96.30 -4.50 LHESLGFR *** HHHHTT E AAAa

1yviA 17 22 5.289 -73.50 -40.90 -72.60 -26.40 -83.90 0.30 79.90 12.30 SMFSQGLV *** HHHHTTS AAAa

1yviA 104 109 5.305 -69.70 -42.70 -63.40 -38.10 -74.20 -9.30 51.00 51.30 QFCQDKSR *** HHHHTT H AAAa

1ywmA 85 90 5.301 -59.00 -46.60 -64.50 -24.60 -85.00 5.00 60.20 27.80 YDVKLGKI *** HHHHTT AAAa

1zin_ 100 105 4.911 -59.00 -38.20 -61.00 -28.00 -105.10 11.60 74.80 35.00 MLADIGRK *** HHHHTT AAAa

1zpdA 13 18 5.738 -59.00 -44.60 -60.70 -28.90 -89.40 -0.90 114.60 4.10 RLVQIGLK *** HHHHTT S AAAa

1zpdA 87 92 5.115 -61.50 -47.30 -63.20 -32.90 -101.70 11.20 58.70 48.20 GAYAENLP *** HHHHTT AAAa

1zpdA 453 458 5.133 -59.20 -50.00 -56.20 -43.80 -95.00 9.40 56.60 45.70 QMVRLKLP *** HHHHTT AAAa

1zrn_ 107 112 6.279 -67.70 -36.90 -62.00 -21.90 -88.00 10.00 70.80 25.20 ELKRRGLK *** HHHHTT E AAAa

1zrn_ 129 134 5.135 -66.00 -41.40 -58.10 -34.70 -89.80 1.30 71.00 16.60 VVSHAGLR *** HHHHTT G AAAa

256bA 78 83 5.142 -62.40 -37.00 -72.30 -19.60 -93.80 8.50 78.60 9.80 KLANEGKV *** HHHHTT H AAAa

2a0b_ 701 706 5.148 -63.90 -45.10 -62.00 -42.90 -74.70 -4.30 54.30 40.90 SNLTAQDK *** HHHHTT H AAAa

2a0b_ 721 726 5.088 -67.90 -28.20 -67.10 -30.00 -109.30 13.00 83.20 19.90 AAGSVGLR *** HHHHTT H AAAa

2baa_ 170 175 4.663 -77.20 -39.70 -71.40 -26.20 -90.50 6.00 78.60 23.40 HAVIAGQW *** HHHHTT AAAa

2baa_ 182 187 4.957 -65.30 -43.00 -62.30 -34.30 -85.90 5.90 76.30 7.60 ADRAAGRV *** HHHHTT AAAa

2bbkH 259 264 5.485 -60.20 -42.40 -57.50 -27.90 -86.10 4.20 95.00 5.20 AERADGWR *** HHHHTTEE AAAa

2bemA 40 45 5.193 -61.70 -46.50 -60.90 -39.50 -73.80 -6.20 71.20 23.40 YQaKLQLN *** HHHHTTSS AAAa

2bepA 21 26 5.140 -71.90 -31.20 -64.80 -28.20 -88.70 6.00 57.90 38.90 EETSKNQI *** HHHHTTSE AAAa

2bgrA 760 765 4.704 -57.80 -51.10 -51.50 -42.00 -94.90 7.20 55.40 42.40 IKQeFSLP *** HHHHTT AAAa

2bj4A 490 495 5.697 -55.80 -44.30 -64.60 -28.90 -83.20 3.90 84.00 15.60 LMAKAGLT *** HHHHTT AAAa

2bjiA 1072 1077 5.446 -63.80 -42.10 -58.70 -30.50 -84.40 -2.10 123.00 -5.10 ESVAAGEK *** HHHHTT AAAa

2bjiA 1202 1207 5.124 -64.10 -41.80 -54.00 -41.40 -81.20 -5.70 84.40 17.30 CLVAAGAA *** HHHHTTSS AAAa

2bjiA 1228 1233 5.312 -70.20 -35.90 -73.10 -16.30 -84.80 1.50 97.40 7.40 IVTEAGGV *** HHHHTT E AAAa

2bkaA 119 124 5.443 -61.10 -44.80 -67.30 -26.10 -84.30 0.60 100.30 -0.60 LAKAGGCK *** HHHHTT AAAa

2bkxA 120 125 5.439 -61.70 -42.60 -66.40 -24.90 -75.60 -10.00 91.60 20.40 LVDSLGDT *** HHHHTTS AAAa

2cb5A 87 92 5.400 -63.10 -54.70 -58.30 -34.60 -86.50 -1.00 59.90 46.90 FMKKLNIE *** HHHHTT S AAAa

2cb5A 121 126 5.478 -68.30 -41.70 -66.10 -28.10 -81.90 2.40 58.00 39.50 DTAQRKEP *** HHHHTT AAAa

2cb5A 195 200 5.434 -61.60 -44.00 -64.40 -35.50 -80.00 -1.70 78.60 33.50 NLVHSGAT *** HHHHTT AAAa

2cb5A 315 320 5.297 -58.20 -47.20 -62.90 -19.00 -96.10 3.70 88.40 15.30 ASIKDGEA *** HHHHTT AAAa

2cb5A 362 367 4.737 -65.70 -48.80 -65.30 -42.20 -93.30 22.90 72.50 19.00 ERLTFGES *** HHHHTTS AAAa

2dri_ 78 83 5.264 -60.80 -46.50 -61.00 -36.50 -81.40 1.70 62.20 36.90 MANQANIP *** HHHHTT AAAa

2dri_ 223 228 5.270 -68.90 -43.70 -66.80 -31.70 -82.20 2.20 77.70 3.00 KAVNDGKL *** HHHHTTSS AAAa

2dri_ 250 255 4.954 -54.60 -46.10 -64.20 -34.20 -79.30 1.60 82.50 2.70 DKVLKGEK *** HHHHTT AAAa

2dtr_ 18 23 4.965 -50.60 -48.70 -62.40 -33.30 -81.50 2.90 79.00 20.00 ELEEEGVT *** HHHHTT AAAa

2dtr_ 48 53 5.349 -67.00 -48.50 -58.50 -30.00 -86.10 -1.00 72.10 29.30 RMERDGLV *** HHHHTTSE AAAa

2ebn_ 80 85 5.086 -60.00 -35.90 -62.00 -26.50 -93.00 5.50 84.70 8.50 PLQDKGIK *** HHHHTT E AAAa

2eng_ 169 174 6.367 -57.50 -37.00 -91.80 -31.70 -84.20 -77.80 -67.20 -43.50 YWRFDWFK *** HHHHTTST AAAA

2erl_ 6 11 5.722 -57.70 -49.00 -67.90 -26.40 -79.10 -6.60 70.60 37.10 QAAIQbVE *** HHHHTT H AAAa

2fdx_ 23 28 5.802 -64.20 -43.50 -60.60 -28.90 -78.00 -0.10 97.40 3.70 GIIESGKD *** HHHHTT AAAa

2fdx_ 103 108 5.594 -65.30 -40.70 -62.20 -26.00 -84.10 -1.80 93.60 10.70 RMNGYGCV *** HHHHTT E AAAa

2gdm_ 97 102 5.225 -59.20 -43.90 -57.20 -38.20 -90.10 8.20 72.60 35.60 VHVSKGVA *** HHHHTT AAAa

2hts_ 253 258 5.212 -59.40 -37.80 -65.30 -26.00 -99.80 16.50 90.90 0.10 QLNMYGWH *** HHHHTTEE AAAa

2igd_ 39 44 4.928 -61.20 -43.70 -60.30 -26.70 -104.10 9.90 75.60 22.00 YANDNGVD *** HHHHTT AAAa

2kinA 22 27 5.190 -69.00 -47.80 -61.60 -35.50 -75.20 -2.00 76.90 10.60 AEILRGDK *** HHHHTT AAAa

2kinA 73 78 5.636 -65.60 -39.50 -71.90 -8.20 -93.30 -4.40 97.30 20.50 KDVLEGYN *** HHHHTT E AAAa

2lisA 105 110 4.572 -70.50 -51.80 -68.90 -32.20 -95.30 6.70 58.70 49.60 FLKDKNMI *** HHHHTT AAAa

2mhr_ 62 67 5.122 -67.00 -47.50 -58.80 -40.70 -83.10 -0.30 48.40 46.00 MMDAAKYS *** HHHHTT T AAAa

2nacA 63 68 5.047 -69.50 -32.00 -72.10 -9.70 -109.90 14.40 74.20 14.40 YLESNGHT *** HHHHTT E AAAa

2nacA 132 137 4.687 -60.50 -53.00 -60.40 -30.20 -103.30 10.90 59.10 32.40 SAIDRNVT *** HHHHTT E AAAa

2nacA 160 165 4.974 -66.50 -46.50 -62.90 -41.40 -89.60 0.80 55.30 45.10 LSLVRNYL *** HHHHTTHH AAAa

2nacA 171 176 4.989 -64.30 -48.40 -59.80 -37.70 -84.40 -2.60 95.80 10.30 EWARKGGW *** HHHHTT AAAa

2nacA 296 301 5.251 -81.50 -43.20 -62.00 -35.40 -87.10 -0.10 73.30 16.40 RALESGRL *** HHHHTTSE AAAa

2nlrA 183 188 5.577 -56.70 -44.00 -63.90 -25.80 -90.10 3.00 85.50 13.70 ATVARGLA *** HHHHTTSS AAAa

2olbA 113 118 5.744 -75.50 -39.70 -60.20 -36.60 -85.80 -8.20 61.70 40.50 YLQYGHIA *** HHHHTTBT AAAa

2olbA 123 128 4.892 -68.00 -46.50 -61.40 -36.20 -75.10 -7.60 81.70 7.90 DDIIAGKK *** HHHHTTSS AAAa

2olbA 235 240 5.339 -72.70 -52.40 -67.80 -22.60 -78.10 -12.20 107.90 -8.20 NRYRSGEI *** HHHHTTS AAAa

2olbA 348 353 5.486 -65.80 -47.50 -60.40 -34.20 -74.10 -2.90 98.10 6.10 LLAEAGFT *** HHHHTT AAAa

2olbA 473 478 5.558 -65.20 -42.50 -60.60 -39.30 -84.10 -4.40 46.60 54.80 QLDKDSAI *** HHHHTT E AAAa

2pgd_ 21 26 5.604 -61.40 -48.60 -58.70 -21.50 -97.60 12.30 81.30 33.40 NMNDHGFV *** HHHHTT AAAa

2pgd_ 41 46 5.788 -60.40 -34.10 -79.30 -26.70 -109.40 -97.50 -58.10 -33.70 DFLANEAK *** HHHHTTTT AAAA

2pgd_ 115 120 5.093 -55.20 -41.50 -60.30 -30.80 -96.30 14.50 70.40 16.00 DLKDKGIL *** HHHHTT E AAAa

2por_ 212 217 5.032 -64.70 -28.70 -55.00 -41.60 -95.50 12.80 62.80 40.30 ARAVFDLT *** HHHHTT AAAa

2pspA 29 34 5.367 -64.70 -44.90 -57.00 -37.40 -72.90 -2.80 88.70 18.60 QdFTSGcb *** HHHHTT E AAAa

2pspA 78 83 5.581 -66.90 -30.50 -66.60 -46.30 -68.50 9.20 69.10 25.60 DgAARNfe *** HHHHTT E AAAa

2pth_ 120 125 4.969 -60.20 -53.60 -67.60 -18.10 -89.90 13.40 85.00 43.10 IISKLGNN *** HHHHTTS AAAa

2pviA 23 28 5.073 -59.60 -40.90 -58.60 -24.80 -111.30 22.40 88.40 6.90 LALKHGIN *** HHHHTT S AAAa

2pviA 131 136 5.078 -62.80 -55.90 -47.60 -46.00 -67.10 5.30 77.70 30.20 KWYSDGHK *** HHHHTTS AAAa

2rn2_ 85 90 4.599 -69.50 -22.70 -89.70 -27.90 -94.60 5.10 71.30 28.80 NWKKRGWK *** HHHHTTSB AAAa

2scpA 120 125 5.239 -61.30 -32.40 -71.90 -35.20 -74.30 3.70 82.50 3.30 FFGMLGLD *** HHHHTT AAAa

2sli_ 753 758 5.037 -65.20 -49.90 -58.80 -32.60 -91.60 15.10 75.70 -11.90 ISELTGQA *** HHHHTT AAAa

2tpsA 174 179 5.715 -69.10 -34.80 -64.30 -36.70 -78.50 9.60 77.20 16.30 AVRRQGIS *** HHHHTT AAAa

2tpsA 197 202 5.977 -68.30 -39.70 -65.90 -32.90 -71.20 -12.20 119.80 4.70 PVIQAGAD *** HHHHTT S AAAa

3c2c_ 55 60 5.381 -70.80 -46.10 -50.40 -38.00 -65.10 -22.60 90.50 28.70 EMKAKGLT *** HHHHTT B AAAa

3daaA 55 60 5.018 -56.10 -35.40 -63.90 -25.60 -116.50 24.80 48.90 44.60 SAEKIRIT *** HHHHTT AAAa

3daaA 159 164 4.768 -65.50 -35.20 -66.30 -23.30 -99.00 8.30 72.10 19.10 EAHEKGCY *** HHHHTT S AAAa

3daaA 213 218 4.907 -68.30 -36.30 -67.90 -33.30 -97.90 7.70 60.80 37.80 CANEINMP *** HHHHTT AAAa

3lzt_ 12 17 5.633 -66.80 -42.10 -58.70 -33.40 -87.50 11.50 83.20 20.80 AMKRHGLD *** HHHHTT T AAAa

3lzt_ 98 103 5.910 -74.60 -32.50 -65.30 -23.90 -90.00 16.80 91.30 6.90 KIVSDGNG *** HHHHTT AAAa

3nul_ 126 131 4.815 -59.30 -46.40 -65.90 -24.70 -87.50 6.40 70.60 23.60 YLIESEL# *** HHHHTT AAAa

3pcgA 23 28 5.240 -47.50 -54.00 -58.30 -34.40 -73.10 -9.00 68.80 45.10 ALEAAGNP *** HHHHT AAAa

4bcl_ 193 198 5.173 -62.10 -33.20 -63.20 -30.80 -83.40 -3.10 66.90 37.30 AINEGGQR *** HHHHTTEE AAAa

4bcl_ 351 356 5.072 -63.00 -30.40 -70.40 -20.30 -94.60 -2.70 118.20 7.80 EHVCKGGT *** HHHHTT AAAa

4eugA 210 215 5.002 -73.00 -41.20 -40.70 -41.00 -107.00 12.10 83.70 18.30 WLEQHGET *** HHHHTT AAAa

4ubpA 23 28 5.679 -64.10 -37.00 -69.40 -27.30 -92.70 13.70 100.60 7.30 RRKARGLK *** HHHHTT AAAa

4ubpA 46 51 5.650 -64.20 -42.80 -61.40 -34.70 -71.50 -16.60 96.00 11.80 EGARDGKT *** HHHHTT AAAa

5csmA 71 76 5.570 -64.20 -32.20 -69.00 -22.90 -105.70 25.00 78.90 16.30 AHSRIRRF *** HHHHTTGG AAAa

5csmA 179 184 5.171 -67.20 -51.50 -52.80 -43.00 -91.90 8.10 49.90 46.40 KLIKSKDV *** HHHHTT H AAAa

7a3hA 176 181 4.944 58.80 52.80 60.20 46.40 55.60 44.40 -110.50 -25.50 TGTWSQDV *** HHHHTBH rrrA

7a3hA 217 222 5.704 -58.90 -36.10 -68.60 -16.50 -90.40 4.70 84.40 5.80 YALDQGAA *** HHHHTT AAAa

7a3hA 252 257 4.845 -65.30 -42.10 -62.70 -35.10 -95.00 5.80 52.20 44.90 FMDERNLS *** HHHHTT AAAa

7ahlA 218 223 6.810 -68.90 -29.30 -61.70 -39.30 -102.20 -71.90 -58.00 -38.00 SSLLSSGF *** HHHHT B AAAA

7hbiA 122 127 4.950 -62.20 -40.20 -66.60 -20.40 -93.80 3.30 70.30 39.10 VLASKNFG *** HHHHTT AAAa

7odcA 98 103 5.925 -70.90 -29.70 -65.60 -20.40 -94.80 9.00 87.10 11.40 LVQGLGVP *** HHHHTT AAAa

7odcA 123 128 4.938 -56.60 -44.10 -65.30 -26.40 -105.80 13.60 76.10 14.90 YAASNGVQ *** HHHHTT AAAa

7odcA 184 189 5.112 -64.70 -41.90 -58.20 -35.90 -86.30 0.30 61.20 39.80 RAKELNID *** HHHHTT E AAAa

8abp_ 53 58 5.347 -57.90 -44.80 -61.80 -37.60 -76.50 -9.10 81.00 22.30 SLAASGAK *** HHHHTT AAAa

8abp_ 78 83 4.871 -52.80 -45.50 -55.30 -33.50 -102.00 10.90 58.80 37.60 KARGYDMK *** HHHHTT E AAAa

8abp_ 216 221 5.553 -59.70 -41.40 -65.90 -20.40 -100.70 17.30 78.20 22.70 ATEGQGFK *** HHHHTT AAAa

8abp_ 298 303 4.983 -69.00 -37.70 -69.30 -21.20 -99.20 -2.10 82.70 13.80 ELEKKGLG *** HHHHTT AAAa

8acn_ 280 285 4.852 -62.60 -43.50 -68.50 -21.30 -101.20 31.20 70.80 23.40 YLSKTGRA *** HHHHTT H AAAa

8acn_ 376 381 5.084 -59.90 -41.00 -60.30 -21.40 -105.50 17.60 65.40 29.20 QALAHGLK *** HHHHTT AAAa

8acn_ 400 405 5.118 -68.20 -52.90 -58.90 -45.00 -86.30 9.90 87.40 -3.80 TIERDGYA *** HHHHTTHH AAAa

8acn_ 409 414 5.405 -64.20 -48.80 -59.80 -30.40 -76.90 -4.40 115.50 12.90 VLRDVGGI *** HHHHTTEE AAAa

8acn_ 623 628 4.673 -58.40 -45.20 -56.70 -41.30 -94.40 11.90 70.80 24.90 YYKQHGIR *** HHHHTT AAAa

8acn_ 651 656 5.025 -63.60 -45.30 -64.60 -38.20 -90.80 18.10 97.40 10.00 EPRFLGGR *** HHHHTTEE AAAa

8acn_ 672 677 5.327 -61.70 -40.10 -59.00 -32.80 -98.60 13.60 81.10 14.80 NLKKQGLL *** HHHHTT E AAAa

(c) π-NHB turns:

PDBID START END CA-CA DIHEDRAL ANGLES OF THE MIDDLE RESIDUES SEQ SSE CONFOR-

DIST (i-1) (i-1) MATION

-(i+6) -(i+6)

PHI1 PSI1 PHI2 PSI2 PHI3 PSI3 PHI4 PSI4

1a12A 43 48 4.90 -53.6 -40.0 -110.7 17.0 99.9 3.1 -66.8 -17.5 QGDVGQLG *** E TTSTT AAaA

1a12A 94 99 5.46 -63.6 -32.0 -106.5 2.1 90.9 14.8 -61.1 -26.9 CNDEGALG *** TTSTT AAaA

1a12A 103 108 5.36 -59.4 -41.1 -62.9 123.6 -50.6 128.0 83.0 -7.6 DTSVEGSE *** STTGG AEEa

1a12A 199 204 5.23 -58.4 -27.1 -105.1 5.8 89.3 7.9 -71.5 -10.8 CGEQGQLG *** TTSTT AAaA

1a12A 211 216 5.83 -66.3 101.3 62.8 -84.7 -19.3 149.6 -91.6 142.8 LFANRGGR *** GTSSS GG EdXE

1a12A 267 272 5.18 -65.5 -19.5 -118.3 24.3 68.5 20.8 -67.7 -21.0 LSNYHQLG *** TTSTTS AAaA

1a12A 372 377 5.93 -43.8 -47.2 -83.3 1.2 67.9 33.7 -82.8 -14.2 MGTNYQLG *** TTSTT AAaA

1a1iA 107 112 6.41 -75.4 -9.5 -69.5 124.6 -43.2 -51.3 -75.0 -9.1 ACPVESCD *** E STT AEAA

1a34A 86 91 6.36 -62.1 156.7 -53.5 139.4 77.5 -7.4 -107.9 162.1 TPPVNEYS *** STT EEaE

1a34A 99 104 4.47 -143.8 142.1 -59.8 -31.4 -84.6 -7.4 153.8 125.2 PLFKTGDS *** E TT EAAx

1a34A 113 118 6.08 -110.2 -1.5 -133.6 126.9 -58.8 -21.3 -92.1 5.3 RASNINTR *** E SSTTS AEAA

1a3aA 91 96 4.57 -85.0 -24.4 -108.2 167.2 -51.7 -45.8 -83.9 6.4 FGEEEDDI *** SSSTT E AEAA

1a3aA 106 111 5.31 -91.8 150.6 59.0 -124.4 -111.3 27.9 -71.3 -10.8 AARNNEHI *** E STTTHH EeAA

1a4iA 269 274 4.82 -78.9 168.0 -137.0 121.3 -78.8 -172.7 87.3 13.5 ITPVPGGV *** E SSSSH EEEa

1a4yA 79 84 5.91 -78.3 69.5 -142.2 129.6 -46.8 8.2 -123.2 -9.6 GLQTPSCK *** TT STT DEAA

1a4yA 136 141 5.81 -74.8 -13.8 -60.0 134.4 -62.3 -17.2 -84.0 -13.8 GLLDPQCR *** HHTSTT AEAA

1a4yA 250 255 6.11 -82.5 -14.2 -52.5 134.2 -58.2 -29.7 -75.8 -22.0 GLLHPSSR *** HHTSTT AEAA

1a4yA 307 312 6.05 -102.2 1.8 -102.8 159.0 -73.2 156.7 100.5 -36.8 TLLEPGCQ *** HHTSTT AEEa

1a4yA 364 369 6.04 -75.1 -2.8 -71.9 165.6 -82.7 143.5 87.7 -18.1 GLGQPGSV *** HHTSTT AEEa

1a8d_ 35 40 5.68 -111.1 18.0 -68.0 -26.2 106.9 -13.6 -86.2 -8.4 SDISGFNS *** EE SSS AAaA

1a8d_ 63 68 5.94 -94.1 68.1 -145.1 164.8 -77.0 -1.5 -95.4 -0.5 LVNNESSE *** EESSTT DEAA

1a8d_ 82 87 6.37 -55.0 -50.4 -124.3 -23.9 -107.6 -179.7 -80.2 157.6 NDMFNNFT *** TTTTS EE AAEE

1a8d_ 142 147 5.68 -92.4 175.9 -75.6 -4.5 -107.6 5.7 98.1 3.8 LKDSAGEV *** EE TTS E EAAa

1a8d_ 280 285 5.72 -170.1 150.4 -56.1 139.4 81.6 -4.5 -114.7 -168.3 LKNITDYM *** ESSTTS E EEaE

1a8d_ 395 400 5.54 -76.8 -157.8 -105.5 18.5 -128.4 27.0 70.2 9.2 LYDDKNAS *** EE SSS E EAAa

1a8d_ 411 416 6.28 -106.5 120.6 69.3 -137.2 -101.4 21.1 -69.7 164.1 GQIGNDPN *** E STTS EeAE

1a8e_ 86 91 5.47 -164.2 165.4 -73.7 -23.5 -69.3 -40.5 -141.6 81.3 YGSKEDPQ *** BS SSS B EAAD

1a8e_ 171 176 5.83 -62.8 -32.1 -92.6 -11.5 -149.3 76.4 -44.5 121.8 LCQLCPGC *** GGSSSTT AADE

1a8e_ 292 297 5.03 -84.5 125.1 72.6 -45.6 -138.0 160.7 -76.4 142.9 KDLLFKDS *** SSSSS TT EaEE

1a8i_ 41 46 6.30 -145.0 154.3 -53.5 -24.9 -70.9 -36.5 -98.6 -12.9 VKDRNVAT *** TTT EAAA

1a8i_ 232 237 5.31 -82.0 101.2 55.4 31.0 -119.3 3.1 61.6 19.2 PGYRNNVV *** E SSS E EaAa

1a8i_ 432 437 5.61 -80.2 164.9 145.3 -175.6 -59.2 -40.6 -111.8 118.5 VEEGAVKR *** EE SSS E EeAE

1a8i_ 667 672 6.18 -64.7 134.6 -49.3 131.7 92.5 -5.6 -106.3 -28.5 ISTAGTEA *** TT S EEaA

1a8l_ 190 195 6.45 -132.2 -79.3 -71.6 140.3 -126.6 158.9 -75.8 144.7 NVMAVPKI *** T SS EE AEEE

1a8vA 104 109 5.94 -88.3 -166.6 -65.1 129.3 104.6 -24.8 -98.3 158.9 PPKEGERY *** TT S EEaE

1aba_ 37 42 5.68 -64.3 -25.2 -152.5 164.2 -51.1 119.6 72.8 8.7 IMPEKGVF *** S SBTTB AEEa

1ae9A 268 273 5.57 -71.9 -33.3 -90.4 143.2 -56.0 -44.5 -99.5 127.1 GGETIIAS *** SBSS AEAE

1ae9A 273 278 5.20 -78.3 178.5 -61.3 -19.2 -101.2 15.0 66.6 17.1 IASTRREP *** S TTS EAAa

1ae9A 344 349 4.15 -91.4 -154.4 -68.3 67.5 114.7 -59.4 -76.3 142.6 RDDRGREW *** EE SS EE EDaE

1af7_ 188 193 4.20 -138.7 159.7 74.1 -128.2 -60.7 -56.5 -94.4 70.9 RGTGPHEG *** E TTS S EeAD

1afwA 375 380 6.17 -80.7 94.4 -14.2 -79.0 -66.6 -17.5 -84.3 -38.1 GHPLGCTG *** B TTTHH EAAA

1aho_ 7 12 5.10 -76.9 -171.2 -72.9 -8.1 -119.9 24.4 73.6 23.0 IVDDVNaT *** BB TTS B EAAa

1ajsA 41 46 5.06 -76.8 179.8 -67.3 -19.3 -84.6 -2.9 68.3 32.9 YRTDDCQP *** TTS EAAa

1ajsA 71 76 6.27 -59.3 155.8 -50.7 -46.4 -77.6 -10.2 107.8 -174.9 YLPILGLA *** TT H EAAe

1ajsA 227 232 4.97 -87.4 -18.5 -69.1 -39.7 -133.2 -6.5 86.9 -4.4 QGFASGNL *** TTTTTS H AAAa

1ajsA 257 262 4.58 -87.7 -47.2 -71.7 -19.2 -109.3 -6.0 60.9 33.1 FSKNFGLY *** TTTS G AAAa

1ajsA 381 386 6.39 -79.8 149.8 -51.5 -30.4 -66.9 -17.7 97.3 9.2 YLLPSGRI *** E TTSEE EAAa

1ajsA 392 397 6.02 -140.2 169.3 -63.3 -30.2 -75.7 -27.3 -112.1 -6.4 GLTTKNLD *** G TTTHH EAAA

1ako_ 251 256 6.02 -84.3 -2.4 -72.7 167.4 -71.3 150.6 45.5 52.6 IRSMEKPS *** HHTSSS AEEa

1al3_ 174 179 5.32 -84.1 -9.5 -71.6 147.5 -54.7 -32.3 -77.9 -17.7 TPEHPLAT *** TTSTTTT AEAA

1al3_ 199 204 5.91 87.6 3.7 -115.9 135.7 34.8 60.7 67.3 21.8 TFGFTGRS *** TTSTTHH aEaa

1al3_ 248 253 6.01 -146.5 166.9 -76.7 -4.6 -107.9 -36.5 -80.2 -30.0 AVDPVSDP *** G TTT T EAAA

1alvA 150 155 6.33 -57.2 -39.0 -96.7 14.1 47.1 43.1 -88.2 -12.4 MDSDTTGK *** H TT SSS AAaA

1alvA 187 192 5.99 -85.5 168.3 -44.5 -48.5 -72.3 -25.5 -87.0 -23.9 TIGSSELP *** SB TTTHH EAAA

1amf_ 118 123 5.38 -47.2 112.7 -63.9 -10.3 -74.2 -26.6 -157.7 -23.0 VGDPEHVP *** EE TTT H EAAA

1amf_ 200 205 6.15 -74.1 140.6 -50.0 126.4 86.7 0.8 -114.4 18.4 AVVEGHNN *** EEBTT S EEaA

1amx_ 234 239 4.90 -151.4 174.0 90.8 -161.1 -85.4 -15.1 -103.5 4.6 YYSGQSAI *** EE SSSHH EeAA

1amx_ 300 305 6.41 -79.7 158.9 -57.8 121.2 77.1 4.9 -115.2 172.0 YQEHGKEE *** E BTTS EEaE

1anf_ 28 33 4.83 -76.4 -18.6 -116.5 8.1 -147.8 -25.1 88.4 -7.3 FEKDTGIK *** HHTTTS AAAa

1aocA 63 68 6.30 -65.0 119.8 -52.9 -33.5 -66.4 -26.2 -113.3 -19.3 KYEdRTVR *** BTTT EAAA

1aocA 101 106 5.10 -67.4 174.3 -51.9 -40.2 -78.1 -0.4 87.2 -2.5 GYTVAGEF *** EEBTT E EAAa

1aohA 60 65 6.11 -85.2 -11.5 -56.1 128.6 -53.8 -27.4 -119.1 82.4 LIVDPNPT *** T SSHH AEAD

1aohA 88 93 6.26 81.2 1.8 -117.8 -8.3 103.1 -4.7 -55.0 -36.5 DSGTGAYA *** TTSSSTT aAaA

1aohA 128 133 5.15 -85.3 -176.0 -66.9 -13.2 -89.0 3.4 61.2 28.9 FANNDLVE *** EE TT B EAAa

1aq0A 238 243 4.50 90.1 0.6 -167.0 179.4 -60.6 134.2 61.8 6.8 SGGGTAAT *** SSSSTT aEEa

1aq0A 262 267 4.47 -113.2 171.1 -52.6 -45.2 -93.2 -12.5 -134.6 73.6 RGTPRHPG *** TB SSSBS EAAD

1arb_ 199 204 4.94 -74.6 171.3 -55.7 -26.1 -85.7 7.3 73.5 33.4 IYSPEKRV *** EE TTS E EAAa

1aru_ 127 132 6.16 -85.4 -9.0 -105.2 121.7 -53.2 135.1 93.8 -10.6 MSNcPGSP *** HHTSTT AEEa

1aru_ 200 205 5.90 -91.1 -18.1 -141.3 175.8 -77.2 -1.7 -132.4 69.8 SPLDSTPQ *** EESSS TT AEAD

1aru_ 235 240 6.13 -81.8 -12.1 -169.2 157.1 -47.4 138.7 83.6 -20.7 LSPFPGEF *** BSSTT AEEa

1aru_ 251 256 5.23 -74.6 141.9 -60.1 -26.9 -73.8 -21.7 -127.8 -11.4 ARDSRTAd *** HHSTTTHH EAAA

1aru_ 282 287 6.18 -62.7 123.8 94.2 3.1 -138.1 165.3 -102.5 113.2 SVLGFDRN *** HTTTS GG EaEE

1aru_ 309 314 6.48 -69.9 156.7 45.6 45.6 84.5 -8.0 -72.1 159.6 VIPGGLTV *** EE TT G EaaE

1atg_ 111 116 5.91 -56.3 116.8 -69.5 -17.4 -74.0 -24.3 -125.1 -14.8 ISNPQIAP *** EE TTT H EAAA

1atzA 1088 1093 6.17 -71.7 -45.3 -126.5 156.4 -62.3 -31.3 -61.4 -22.5 KLQRIEDL *** EESSTTHH AEAA

1avwB 521 526 5.96 -72.3 -27.4 -100.8 158.6 -75.1 3.1 -76.9 -30.4 LSDITAFG *** EESSTT AEAA

1avwB 533 538 6.13 -142.1 148.6 57.2 -124.7 -99.4 16.3 -64.7 135.2 APTGNERg *** E TT SS EeAE

1avwB 598 603 5.73 -85.1 157.8 -46.5 -57.7 -82.8 32.5 78.6 179.0 EDLPEGPA *** S TTSSE EAAe

1avwB 623 630 5.61 -97.6 360.0 360.0 -70.6 -100.2 155.8 -89.4 85.9 RVSEFNNY *** E E AxED

1ayfA 46 51 5.82 54.6 44.3 65.9 48.0 -119.0 15.6 59.0 45.7 ACEGTLAC *** TTSSSSS aaAa

1ayl_ 74 79 5.51 -141.5 -169.5 -68.9 -2.2 -101.2 -31.8 -108.7 -14.6 VRDDTTRD *** E STTTTT EAAA

1ayl_ 237 242 5.48 -90.6 176.1 -62.8 -29.1 -79.3 -9.0 96.8 9.3 NVGEKGDV *** EE TT E EAAa

1ayl_ 273 278 4.47 -113.1 -163.6 -60.4 -24.8 -94.0 -24.2 144.0 -158.9 GWDDDGVF *** EE SS EE EAAe

1ayl_ 443 448 5.84 -142.0 -163.8 -61.0 -15.4 -78.6 -14.5 81.3 4.7 GWNGTGKR *** SB TTSSB EAAa

1az9_ 277 282 5.37 -57.7 -36.7 -90.6 5.0 85.5 1.7 -139.1 118.1 FPVNGKFT *** EETTS AAaE

1az9_ 411 416 4.26 -119.1 -175.6 -66.7 -27.1 -122.5 6.7 174.4 -115.3 VITETGNE *** EE SSSEE EAAe

1b25A 12 17 5.17 -74.7 -15.7 -80.2 -43.9 -95.2 -15.8 82.3 1.0 VNLTTGEV *** EETTT E AAAa

1b25A 51 56 5.54 -102.9 14.0 -93.8 162.1 -59.0 -19.8 -77.7 -17.8 EPLSPENK *** TTSTTS AEAA

1b25A 65 70 6.18 -75.0 143.6 87.0 -6.3 -118.6 157.1 -74.2 74.4 PFNGLPTP *** TTTTS T EaED

1b25A 431 436 6.29 -55.0 -23.8 -112.4 1.1 115.0 177.2 -49.1 113.1 TSAIGAHH *** H TTSS S AAeE

1b25A 562 567 5.20 -163.8 175.6 80.0 177.2 -65.5 -7.4 -109.8 9.7 LKSGPHKG *** SSSTTTT EeAA

1b25A 613 618 6.17 -69.0 -18.4 -114.3 -24.0 -157.7 178.1 -93.9 126.5 LEKVTNLE *** HHTTS AAEE

1b4pA 35 40 3.97 -84.2 -175.5 -69.8 158.6 -100.3 -9.5 -92.9 0.6 MGDAPDYD *** TTT EEAA

1b4pA 197 202 5.67 -105.4 0.4 -94.9 170.8 -81.5 1.0 -86.6 -13.3 YMKSGRFL *** HHTSTT AEAA

1b4pA 211 216 6.48 -54.9 161.7 -97.4 -8.7 -146.5 126.9 35.7 70.3 KMAFWNPK *** TT SSS EAEa

1b5qA 131 136 6.36 -55.2 -29.6 -66.8 -17.3 89.7 -5.0 -60.5 -20.7 LHASGRDD *** S TTSTT AAaA

1b5qA 242 247 4.32 -149.7 177.8 -66.9 -20.0 -99.6 -18.3 177.9 -166.2 KYSPGGVT *** EE SS EE EAAe

1b5qA 311 316 6.31 -132.2 154.7 130.7 -173.3 -58.8 130.9 70.9 7.6 WPEGKGRE *** S STT S EeEa

1b5qA 323 328 6.29 -77.6 -7.2 -101.5 106.4 -50.7 133.9 74.6 6.9 ASSRRGYY *** E SSTTSS AEEa

1b5qA 395 400 5.84 -78.3 118.9 -60.8 -17.2 -92.1 4.9 -138.6 -47.3 WSDRFYKG *** TT TTTSS EAAA

1b65A 47 52 5.50 -75.1 159.3 -54.6 125.1 91.9 -5.5 -108.7 160.1 EPRPGRKR *** S TT SS EEaE

1b65A 148 153 5.25 -72.1 -36.9 -109.0 -11.0 -119.9 150.2 -79.0 71.7 DGALNDIN *** TTTS GG AAED

1b65A 347 352 5.94 80.5 9.2 -88.1 175.9 -63.7 -19.5 -93.7 1.2 MGGTPFDR *** EE STT S aEAA

1b6a_ 124 129 6.41 -65.2 -24.2 -71.4 -16.9 85.1 16.7 -112.0 116.7 LYPNGVFP *** HSTTS AAaE

1b6a_ 333 338 5.04 -126.5 178.0 -65.4 119.0 59.1 23.0 -138.4 94.1 SIGQYRIH *** EEBTTBS EEaE

1b6a_ 345 350 6.15 -116.9 11.7 -135.8 165.9 -66.8 162.6 99.6 9.5 VPIVKGGE *** EESSSS AEEa

1b6a_ 464 469 4.97 -76.7 158.3 -58.7 -35.8 -93.5 -11.7 177.9 163.3 LLRPTCKE *** EE SS EE EAAe

1b8oA 31 36 5.62 -80.5 -167.3 -53.4 120.0 70.9 14.9 -113.8 15.5 ICGSGLGG *** EE TT GG EEaA

1b8oA 59 64 6.14 -113.6 0.0 -93.2 134.1 -57.5 139.5 95.0 2.6 ESTVPGHA *** STT AEEa

1b8oA 119 124 6.27 -105.3 -31.0 -71.8 119.4 -58.2 -20.9 -83.9 -5.1 GGLNPNFE *** EE STT AEAA

1bdmA 127 132 5.74 -107.2 -41.8 -52.3 134.5 -82.8 154.6 -78.4 111.2 LVVGNPAN *** EE SSSHH AEEE

1bdmA 185 190 5.60 -83.9 97.0 -166.2 172.7 -70.8 -19.3 -108.0 22.8 GNHSSIMF *** B SSTT E EEAA

1bdmA 260 265 6.16 -68.9 163.9 -67.1 135.8 81.1 2.4 -126.2 171.8 GTPEGDWV *** B TT E EEaE

1bdo_ 94 99 5.92 -80.2 -9.9 -164.2 161.0 -56.4 -23.5 -106.6 -2.7 RTPSPDAK *** SSSSTTS AEAA

1bea_ 69 74 5.70 -75.7 146.3 156.7 164.3 -47.6 -56.8 -85.2 -82.8 IPPGPDAQ *** SSS EeAA

1bea_ 82 87 4.69 -123.4 147.8 -39.9 128.1 50.1 40.7 -162.0 79.8 EDLPGcPR *** BTTB H EEaD

1bf2_ 12 17 5.71 -44.2 -48.2 -89.3 6.5 58.5 35.6 -93.3 -21.4 YDAQQANI *** E TTSSEE AAaA

1bf2_ 82 87 5.35 -163.2 -175.6 -48.4 -35.9 -83.7 -9.8 -159.7 76.6 AWGPNWPY *** EEBTTB EAAD

1bf2_ 104 109 5.46 -98.7 178.5 -55.9 -28.9 -89.7 -5.4 98.0 4.8 DVDANGDR *** SS TT EAAa

1bf2_ 130 135 4.64 -150.9 -176.6 -65.2 -15.6 -93.3 -27.7 -145.5 123.9 PLNPSNQN *** SS SS EAAE

1bf2_ 329 334 5.42 -57.1 130.8 96.1 -14.6 63.4 14.9 -90.1 -25.7 LTSGNQYF *** B TTSSSB EaaA

1bf2_ 337 342 5.83 -129.0 20.5 -116.9 -20.1 82.5 -4.7 -89.5 -21.1 YDNTGIGA *** SSSSS AAaA

1bf2_ 422 427 6.15 -67.3 163.2 -60.2 -32.0 -85.8 -8.2 109.5 -173.8 VRPAAGGS *** B TT S EAAe

1bf2_ 438 443 5.25 -125.7 120.0 86.2 173.1 53.9 -152.5 -92.5 37.6 WAIGGNSY *** SSTT EeeA

1bf2_ 597 602 6.19 -92.0 69.6 -140.3 173.1 -60.9 -26.4 -68.6 -32.2 NLDSSANW *** T STTTS DEAA

1bf2_ 638 643 6.19 -84.6 171.1 -63.5 -30.5 -81.1 -22.7 -81.0 -31.5 WYSGSQLT *** TTTEE EAAA

1bf2_ 699 704 6.44 -61.7 -32.3 -136.0 154.2 77.1 -171.7 -120.5 -25.9 APPSGTQW *** SSSS E AEeA

1bf2_ 715 720 5.23 -69.0 128.8 164.2 -158.8 -80.3 -26.2 -108.7 29.8 WNDGASTF *** GG STTSB EeAA

1bf2_ 722 727 6.24 -81.5 146.7 -48.0 134.3 93.8 -12.6 -107.8 25.0 FVAPGSET *** B TT E EEaA

1bgf_ 22 27 5.44 -148.7 -163.6 -57.8 -18.5 -91.9 -23.3 -161.2 101.8 FYDDNFPM *** G BTTB H EAAE

1bkf_ 86 91 4.79 -131.8 125.1 -55.1 122.6 77.9 0.4 -132.4 -49.3 TGVPGIIP *** T BTTTB EEaA

1bkrA 85 90 5.28 -83.7 2.1 -64.5 166.8 -59.9 -40.1 -133.1 81.1 DISVDHPD *** HHSSSS AEAD

1bm8_ 19 24 4.48 -107.4 164.6 -60.7 -44.4 -69.9 -16.9 148.3 -173.1 FIHSTGSI *** EE SS EE EAAe

1bm8_ 69 74 5.70 -51.4 144.1 121.2 -169.2 -67.5 -11.7 -73.5 -29.3 GGFGKYQG *** SS STT E EeAA

1bqcA 229 234 4.04 -101.7 10.0 -103.9 159.3 -44.0 113.2 99.3 -22.5 HDHSDGNP *** TTSTT AEEa

1bqcA 259 264 4.81 -74.2 176.1 -36.6 -50.0 -75.1 -15.9 -130.8 36.2 GNGGGVEY *** TTTGG EAAA

1brt_ 30 35 5.88 -124.5 -172.0 -71.4 143.9 -111.7 48.4 -101.7 -145.8 IHGFPLSG *** E TT G EEDX

1brt_ 131 136 5.62 -137.6 178.2 -62.7 -38.7 -85.2 -2.2 -139.1 76.1 LKTDDNPD *** B BTTBTT EAAD

1bs0A 235 240 4.98 -119.1 -102.9 -56.5 -32.8 -73.7 -15.1 74.2 21.5 FGKGFGVS *** SSSTTSS XAAa

1bs0A 316 321 6.44 -117.8 10.1 -84.3 165.1 -57.0 -19.9 -83.6 113.0 DSCSAIQP *** S SSB AEAE

1bs0A 350 355 5.94 -93.6 19.1 -66.0 -27.9 -124.0 157.0 -56.2 155.1 RPPTVPAG *** TTSS TT AAEE

1bteA 86 91 6.27 -113.5 143.8 116.2 136.5 -53.7 122.0 69.8 18.0 ceEGNMeN *** EE STTGG EeEa

1bupA 44 49 4.70 -129.4 -176.1 -62.1 -19.1 -132.4 -10.1 -157.9 168.9 AFTDTERL *** EE SS EE EAAE

1bupA 200 205 4.41 -97.7 -145.0 -87.5 -24.1 -132.2 -19.3 -132.1 157.4 DLGGGTFD *** EE SS EE EAAE

1bupA 274 279 6.07 -80.1 -14.6 -128.3 -6.4 -136.1 160.1 -91.5 0.6 TLSSSTQA *** HHTTSSEE AAEA

1bx4A 274 279 4.22 -122.7 -177.1 -56.4 -38.7 -93.2 -26.7 -164.3 176.0 MATESEVT *** EE SS EE EAAE

1bx7_ 5 10 5.54 -108.4 101.8 55.5 30.3 88.5 1.4 -124.0 158.9 NTaGGETb *** BTTB EaaE

1by2_ 11 16 5.07 91.1 -70.8 -84.8 170.1 -86.9 -14.5 -97.3 23.5 DGGATNQG *** S SSTTEE dEAA

1byi_ 45 50 4.56 -140.7 173.4 -65.9 -17.8 -95.1 -0.6 112.3 -173.3 EKTPEGLR *** B TTS B EAAe

1c1dA 32 37 5.10 -64.6 -43.0 -86.8 -12.1 161.6 -174.4 -58.2 149.8 STQLGPAA *** E SSSSEE AAeE

1c1dA 117 122 5.30 -154.8 -158.8 -61.2 127.9 76.5 8.1 -128.3 147.8 GPDVNTNS *** EE TT H EEaE

1c2aA 16 21 5.21 -86.8 79.7 -144.0 164.5 -112.3 108.7 -80.6 164.6 cTRSIPPI *** E SSSS E DEEE

1c2aA 75 80 5.24 -87.1 -5.0 -70.3 160.2 -125.8 105.5 -75.9 151.6 hTRSNPPT *** E SSSS E AEEE

1c2aA 100 105 5.87 -80.8 143.9 -72.1 -50.2 -69.0 -36.9 -136.2 86.1 LPSRSRPS *** EE SS TT EAAD

1c3cA 187 192 6.07 -65.7 -33.4 -114.4 -9.1 -145.4 135.5 -79.2 153.6 NYANVPPE *** S SSS HH AAEE

1c3cA 297 302 5.65 -76.7 125.3 51.1 -128.6 -83.2 2.4 152.3 152.5 ALWHERDI *** STT S EeAe

1c3pA 133 138 5.01 -100.0 167.0 -48.4 -40.9 -133.0 31.7 -162.2 141.7 HAFKSRAN *** T BTTB B EAAE

1c3pA 138 143 4.57 -170.9 138.7 55.0 42.3 76.4 -2.6 -103.7 136.7 RANGFCYI *** B BTTBSS EaaE

1c3pA 258 263 5.49 -70.4 -11.5 -68.5 147.4 -49.1 -42.4 -71.6 -10.9 TDPLLEDY *** STTBTT T AEAA

1c4qA 156 161 4.94 -87.9 75.2 -154.4 162.8 -58.4 125.7 90.4 -0.4 NAaHNGGG *** S STTEE DEEa

1c52_ 37 42 4.62 -70.2 -14.5 -92.9 145.4 -68.5 132.4 91.9 -8.9 ILAKEGGR *** HHTSTTHH AEEa

1c75A 42 47 3.79 -146.3 -112.2 -64.5 -34.0 -106.7 168.0 -65.9 164.1 TGASAPAI *** S SSS XAEE

1c7kA 57 62 5.65 -79.1 -2.9 74.7 9.4 -139.1 51.5 102.5 -12.6 QTDGHGRG *** EE SSS E AaDa

1cb8A 95 100 5.48 -85.8 8.0 -91.8 148.4 -74.9 5.7 -117.2 -2.6 EKDSHYYG *** TTSTTTT AEAA

1cb8A 223 228 4.87 -151.1 135.8 80.6 66.1 76.3 -168.0 -80.7 65.6 YLQHGPQL *** EE SSS B EaeD

1cb8A 363 368 6.38 -158.8 -175.2 -67.1 -17.4 -88.8 -7.2 -132.4 179.1 MVSKRTRR *** TTSB EAAE

1cb8A 372 377 4.96 -174.2 141.9 55.9 35.1 60.0 31.0 -110.7 147.9 ESGNKENL *** BTTBST EaaE

1cb8A 440 445 4.15 -104.6 9.8 98.1 -12.9 -134.9 -37.3 -123.4 166.8 VSDGVYGA *** EE SS EE AaAE

1cb8A 464 469 4.71 -120.0 -154.2 -89.6 -60.8 -81.8 -17.5 -148.3 159.9 FFFDKEIV *** EE SS EE EAAE

1cb8A 649 654 4.84 -64.6 -19.0 -105.9 41.2 174.6 -125.6 -75.7 90.8 KHINGKQV *** E TTSS E ADeE

1ccwA 62 67 5.59 -105.9 -35.3 -78.9 -5.8 115.2 -26.2 -89.0 26.0 SSLYGQGE *** EE SSTHH AAaA

1ccwB 172 177 5.59 -176.2 168.2 -74.4 -17.0 -94.9 -53.3 -70.5 -23.7 EGGGISYN *** E TTTTT EAAA

1ccwB 409 414 6.44 -68.5 -17.7 -57.5 132.4 -69.7 -14.4 -77.5 -8.4 FGPSKYNA *** T TTS AEAA

1cf3A 39 44 5.89 -67.1 -33.9 -82.3 113.8 -60.5 -17.8 -81.5 -19.7 LTENPNIS *** HTTSTT AEAA

1cf3A 72 77 5.66 94.8 -8.1 -87.6 -174.8 -75.3 -8.0 -89.7 1.2 IFGSSVDH *** TTTSTTB aEAA

1cf3A 97 102 4.50 -123.6 101.1 150.3 172.3 -62.8 122.1 107.9 -10.5 SGNGLGGS *** B STTGG EeEa

1cf3A 204 209 4.89 -96.8 4.6 -94.9 -13.3 116.4 4.7 -146.9 77.5 DFGaGDPH *** TTSS AAaD

1cf3A 219 224 5.24 -79.2 168.1 -59.9 -31.6 -79.5 11.0 71.7 19.2 TLHEDQVR *** SB TT BB EAAa

1cf3A 257 262 4.82 -104.3 49.1 -157.8 -169.9 -73.1 -47.9 -111.1 135.1 SQNGTTPR *** E SSSS E DEAE

1cf3A 289 294 5.21 107.6 157.8 -63.6 -23.3 -84.1 -16.1 -114.3 -45.9 AAGSAVSP *** S TTTHH eAAA

1cf3A 532 537 4.96 -87.5 -168.4 -75.9 4.1 -117.8 19.9 66.1 23.2 VVDNAARV *** SB TTSBB EAAa

1chd_ 252 257 4.87 -123.1 126.4 61.2 30.1 65.3 26.3 -133.4 147.7 PPVNRHRP *** BTTBSS EaaE

1chmA 70 75 4.59 -109.3 -173.7 -60.7 -28.0 -109.7 -34.0 -97.8 157.6 VVTEDDVI *** EE SS EE EAAE

1chmA 365 370 4.83 88.4 -39.4 -58.9 153.4 -60.2 149.5 49.5 34.6 PEGLPGAG *** TTSTT E aEEa

1ci9A 110 115 5.42 -79.2 178.6 -69.5 7.8 -110.6 -21.9 123.5 -19.4 PRLADGSE *** B TTS EAAa

1ci9A 249 254 5.74 -58.7 150.3 -42.3 130.8 76.9 9.6 -114.5 -12.5 PLPEGHGA *** E TTS S EEaA

1ci9A 271 276 5.82 -97.5 114.8 -65.8 -21.1 -97.7 -13.2 -151.8 3.1 YPSGGAGM *** TTT EAAA

1cjwA 126 131 6.07 -52.0 136.1 -50.9 -27.9 -87.6 0.9 -125.5 13.2 AVHRSFRQ *** EE TTSTT EAAA

1cjwA 148 153 5.79 -73.2 -18.8 -83.2 102.3 -62.4 99.0 166.0 -36.8 VGAQPAVR *** HHTSTT AEEx

1cjwA 182 187 6.20 -134.8 105.4 60.8 -129.8 -100.5 8.1 -81.7 140.9 IVVGSLTF *** BTTB EeAE

1cnv_ 115 120 4.52 -123.1 -170.3 -51.7 -31.0 -104.4 14.4 94.8 -161.5 REGPLGKV *** S BTTBS EAAe

1cnv_ 196 201 5.54 -142.9 149.9 -43.9 120.5 70.0 17.5 -147.8 103.4 QYSTGNIQ *** S BTTB H EEaE

1cozA 60 65 6.13 -95.1 -8.6 -75.3 131.9 -52.6 -36.5 -72.7 -21.9 LETIRYVD *** HTTBTT AEAA

1cpo_ 7 12 6.23 -65.7 138.4 -98.2 21.7 -83.1 -44.6 -131.7 105.6 IGYPYDNN *** TTTTT SS EAAE

1cpo_ 118 123 6.24 -79.4 -30.8 -114.1 139.7 -59.2 -23.1 -91.8 -4.6 GVANSNDF *** T SSTT AEAA

1cpo_ 168 173 6.03 -53.6 137.0 -49.7 124.0 99.8 -13.6 -83.0 -50.7 LDFPGWFT *** HB TTT EEaA

1cpo_ 194 199 5.39 -82.2 -15.9 -83.0 135.1 -54.1 -27.5 -112.6 31.5 DFNLPDND *** TTSTTTT AEAA

1cruA 44 49 5.24 -63.8 -37.9 -89.3 -36.3 -80.7 -23.2 78.8 12.2 TERATGKI *** EETTT EE AAAa

1cruA 247 252 4.21 -75.2 -108.6 -85.6 -3.7 -154.3 175.7 57.4 50.1 QGPNSDDE *** E SSS EE XAEa

1cruA 375 380 5.48 -75.7 -59.3 -71.7 -26.8 -96.3 -10.7 61.8 42.2 PSLKRGVI *** EBSSS E AAAa

1cruA 387 392 6.02 -63.6 -20.9 -95.4 0.8 57.6 42.1 -100.4 -26.4 LDPTYSTT *** E TTSSSE AAaA

1cruA 413 418 5.45 -57.5 -35.7 -75.1 -19.3 86.9 -0.6 -95.2 -2.7 ASPDGNVL *** E TTSS E AAaA

1crzA 59 64 4.98 -99.0 -3.7 -152.0 164.1 -59.6 -40.6 -63.4 -32.5 QPGSAQEV *** SSSTT AEAA

1crzA 153 158 6.13 -73.6 146.2 -86.4 -33.6 -68.1 -52.9 -127.7 71.8 TNGGQFPY *** S SSS E EAAD

1crzA 187 192 5.22 -57.8 -28.6 -86.0 8.1 67.1 8.3 -93.1 -16.7 WSPDGSKL *** E TTSSEE AAaA

1crzA 198 203 4.70 -56.2 -39.0 -70.3 -7.7 111.9 -28.8 -132.8 171.2 TFESGRSA *** E TTSS E AAaE

1crzA 231 236 5.20 -51.4 -33.6 -79.2 -13.1 93.1 -5.4 -83.9 -21.3 FSPDGSKL *** E TTSSEE AAaA

1crzA 242 247 3.55 -105.0 111.4 166.4 -1.1 108.3 -2.8 -152.1 146.7 LSKTGSLN *** E TTSS E ExaE

1crzA 275 280 5.44 -58.4 -21.4 -82.1 -8.4 64.1 30.0 -132.7 -36.0 WFPDSQNL *** E TTSSEE AAaA

1crzA 286 291 4.49 -51.3 -30.8 -109.5 21.9 101.1 -118.1 -55.0 143.1 SDQAGRPQ *** E TTSS E AAeE

1crzA 319 324 5.56 -61.0 -26.4 -97.0 3.6 65.5 9.0 -77.1 -23.7 VSSDGKFX *** E TTSSEE AAaA

1crzA 362 367 5.37 -59.7 -18.9 -100.0 5.5 62.7 15.5 -91.7 -11.3 LAPNGTXV *** E TTSSEE AAaA

1crzA 384 389 5.28 -69.5 -9.5 -92.1 -2.0 78.0 -0.9 -73.5 -31.4 VSTDGRFK *** EETTS E AAaA

1cs0A 18 23 4.62 -138.8 167.8 -60.5 129.5 90.9 -16.5 -144.9 84.8 PIVIGQAC *** S BTTB T EEaD

1cs0A 222 227 5.66 -110.1 169.4 -63.5 -21.2 -80.8 -4.0 87.9 8.7 VRDKNDNC *** EE TT E EAAa

1cs0A 570 575 4.94 -147.6 170.4 -64.8 126.0 97.6 -16.2 -133.0 63.1 PNRIGQGI *** S BTTB H EEaD

1cs0A 768 773 4.50 -115.7 11.2 91.0 -21.4 -103.6 -50.7 -138.6 166.6 ICDGEMVL *** EE SS EE AaAE

1cs0A 992 997 5.84 -67.8 162.7 -57.9 -33.9 -83.0 -14.8 -67.7 -48.7 VNKVHEGR *** EB TTT S EAAA

1cs1A 17 22 5.52 -90.0 -16.4 -84.6 -46.0 -110.4 -2.0 70.1 26.8 DDEQYGCV *** TTT BS AAAa

1cs1A 177 182 5.97 71.5 163.9 -62.0 -17.8 -73.0 -20.7 -122.8 -0.1 FLSPALQN *** TT TTT eAAA

1ct5A 222 227 3.89 -117.4 -165.0 -58.0 -39.2 -72.4 -23.5 -141.4 16.5 XGXSADFR *** TTTHH EAAA

1cv8_ 112 117 5.26 -148.6 106.5 46.4 48.7 70.6 3.3 -106.4 143.3 ESRNGMHA *** S BTTB EaaE

1cxlA 40 45 6.45 -54.2 -25.2 -83.4 -11.4 56.9 39.8 -88.1 -40.6 FDGTaTNL *** B TT ST AAaA

1cxlA 96 101 5.97 -52.4 -29.4 -71.8 -19.9 81.3 4.4 -89.7 4.2 TAYHGYWA *** STTS SE AAaA

1cxlA 149 154 5.24 -79.1 -16.6 -73.4 129.9 50.7 -136.1 -110.4 20.9 QPSFAENG *** TTSTTTT AEeA

1cxlA 462 467 6.07 -53.6 -39.3 -110.5 36.5 63.6 29.3 -74.7 173.9 GGLLNGNT *** TTTTS AAaE

1cxlA 566 571 5.01 -73.6 174.6 -55.1 -26.7 -79.8 -12.0 100.8 0.1 VANAAGTA *** EE TT B EAAa

1cxpA 15 20 6.21 -95.7 109.1 -60.4 -44.9 -68.2 -33.3 -135.2 78.1 aNNRRSPT *** TSSSSSTT EAAD

1cxpC 121 126 5.71 -146.8 146.8 -45.1 137.5 -97.5 27.1 -92.8 145.0 VQQPPbFP *** TTB EEAE

1cxpC 133 138 6.47 -55.4 138.6 -71.5 -11.6 -118.8 -55.5 -90.8 90.9 PNDPRIKN *** TT SS S EAAE

1cxpC 204 209 5.48 -106.8 94.3 47.7 45.9 87.7 -3.7 -107.5 159.9 FQDNGRAL *** BTTB EaaE

1cxpC 235 240 6.33 -64.0 -28.5 -117.7 142.7 -66.0 -8.6 -107.0 15.2 LAGDTRSS *** SSTTTT AEAA

1cxpC 352 357 4.99 -79.8 101.2 -73.3 167.6 -75.2 -10.7 -137.4 83.9 QPMEPNPR *** SB SS SE EEAD

1cxpC 392 397 5.44 -130.1 155.5 -62.3 -24.9 -113.5 18.7 -146.7 62.9 LNRQNQIA *** BTTB S EAAD

1cxpC 408 413 5.84 -77.1 -16.5 -123.0 11.3 -130.7 160.9 -61.1 -31.4 FEQVMRIG *** TTTTSSS AAEA

1cxpC 514 519 6.30 -92.9 -2.0 -68.9 133.1 -47.9 128.4 92.3 -10.1 WWENEGVF *** TTSTTTS AEEa

1cxpC 555 560 6.47 -132.6 114.8 -87.8 139.4 -91.2 -8.8 -40.2 -54.1 SNSYPRDF *** STTTTE EEAA

1d02A 35 40 5.69 -56.7 121.7 75.7 11.8 -103.6 179.1 -88.7 13.7 VFEGTKYV *** HTTTSSEE EaEA

1d02A 105 110 6.15 -135.3 -177.8 -54.5 137.6 84.5 -2.4 -121.4 159.5 GWVEGKDP *** TTS G EEaE

1d0dA 38 43 6.22 -73.9 139.9 -48.5 -41.6 -75.2 -13.6 -96.5 -22.6 WIbPEDHT *** EE TTT EAAA

1d2nA 642 647 6.23 -52.6 147.5 -42.9 127.4 92.9 -20.6 -92.0 151.8 APPQGRKL *** STT EE EEaE

1d3gA 99 104 6.21 -65.2 -32.0 -115.4 12.0 71.1 38.3 -96.4 -13.4 FDKHGEAV *** SSTTSSSH AAaA

1d3vA 41 46 6.45 -93.9 -2.2 -90.4 -173.0 -80.5 -9.3 -92.1 156.8 LKETEYNV *** HTTSSS E AEAE

1d3vA 63 68 4.64 -88.3 103.5 62.5 -109.3 -102.4 -16.4 -68.1 118.6 SPFQIVKN *** BTTB S EeAE

1d3vA 130 135 5.28 -106.8 168.5 -55.0 -16.3 -103.2 -17.3 -98.8 -27.0 INTPLTTS *** TTT S EAAA

1d3vA 236 241 6.39 -60.4 146.1 -51.8 -22.0 -69.1 -24.4 -102.4 -38.1 GLDPVFTP *** GB TTT EAAA

1d4oA 118 123 5.28 -102.9 10.7 -67.7 151.8 -63.2 -23.8 -102.8 17.9 DPNSIIAG *** TTSTTTT AEAA

1d4oA 145 150 5.67 -109.4 -156.7 -50.5 -44.7 -77.3 -6.1 63.1 41.3 GVGYAAVD *** TT EAAa

1d4tA 87 92 6.22 -74.6 -19.2 -82.1 153.4 -63.2 146.1 59.0 57.4 AFQKPDQG *** HHTSSSSS AEEa

1d5tA 39 44 6.23 96.6 -7.9 54.4 -131.5 -68.8 -29.8 -92.4 55.7 YYGGESSS *** SS TTS E aeAD

1d5tA 161 166 5.90 -98.4 103.2 -55.7 -25.2 -89.1 -7.7 -141.5 -33.7 GVDPQNTS *** T TTTSB EAAA

1d5tA 217 222 5.90 -155.5 103.3 82.5 -20.5 139.4 41.6 -103.8 -180.0 LARYGKSP *** SSS S EaaE

1d5tA 344 349 6.48 -59.8 156.7 -50.4 132.9 81.7 -1.1 -109.3 152.0 VAAQGKYI *** SS TT EE EEaE

1d5tA 357 362 5.76 -121.2 -8.5 -136.6 177.4 -88.8 3.0 -118.7 71.6 TVETTDPE *** E SS HH AEAD

1d5tA 393 398 4.82 -60.0 -37.1 -94.6 -51.1 -71.7 -25.4 59.1 25.3 DGSESQVF *** STTT EE AAAa

1d7uA 275 280 4.85 51.9 55.7 97.7 -31.7 -82.5 156.0 -80.7 99.5 LGAGLPLA *** HHTTSS E aaEE

1d8wA 149 154 5.49 -55.7 -25.2 -84.3 -2.0 84.8 11.9 -133.5 143.7 LSADGFTL *** GGTTS ST AAaE

1d8wA 238 243 4.74 -126.3 150.3 -60.5 122.6 86.6 1.9 -144.5 37.7 LFGIGAES *** S BTTB S EEaA

1d8wA 298 303 5.05 -132.7 102.7 67.9 -10.2 -176.1 176.1 -68.9 62.1 RPVRWDSD *** B SSSS EaED

1d8wA 406 411 5.66 -83.0 -28.0 -129.5 -157.9 -60.3 -7.4 -54.4 -26.0 TPAGSEWL *** STTHH AEAA

1dbxA 98 103 5.25 -98.3 149.0 -52.9 135.1 99.5 -15.3 -143.1 22.2 GYLVGGIS *** S TT EEaA

1dbxA 130 135 5.00 102.9 2.1 -154.9 155.3 -57.1 135.0 98.6 -6.5 SGGKRGLS *** E SSTTEE aEEa

1dciA 108 113 4.40 -82.7 156.6 79.5 -153.0 -67.3 -33.2 -148.3 156.2 SGAGKMFT *** EESTT SB EeAE

1dcs_ 103 108 5.75 -145.7 -157.9 -74.0 -20.0 -159.5 174.8 60.9 37.7 SMGTADNL *** EE SSS EAEa

1dcs_ 144 149 6.06 -59.1 150.2 -53.7 127.7 89.9 -1.3 92.2 162.5 TEPDGGVE *** TT HH EEae

1dekA 48 53 5.48 -40.9 -72.5 -115.9 44.8 -116.3 -177.6 -110.7 17.8 FAANTDYP *** HSTTSSS ADEA

1dekA 91 96 5.72 -82.4 -12.5 -92.1 -44.0 -162.9 123.1 -47.2 128.0 LNGKSPIK *** HHTTS T AAEE

1dekA 103 108 4.84 -129.9 66.3 63.1 82.9 57.4 15.0 -118.7 105.2 FDDEGKES *** EESSS EE DaaE

1dfmA 38 43 4.59 -73.8 140.5 -52.1 137.1 73.2 -4.0 -135.7 30.6 SDQASKVG *** STT TT EEaA

1dfmA 186 191 6.24 -138.6 159.8 -71.1 -19.3 -139.9 142.2 68.3 -18.9 YADKRYSR *** ESSSSS EAEa

1dg6A 158 163 5.62 -136.6 29.2 87.8 -42.4 -91.4 1.1 -103.7 130.6 SRSGHSFL *** S SS EE AaAE

1dg6A 268 273 5.45 -50.1 139.2 -69.3 -20.2 -88.4 -13.9 -99.9 -33.8 DMDHEASF *** E TTTSE EAAA

1dgwA 165 170 5.83 -152.8 -31.4 -68.2 -48.8 -137.8 88.9 -35.3 124.6 ITFRRPGT *** E SSTT AADE

1dgwA 179 184 3.49 -144.4 -155.0 -86.9 -25.3 -65.8 -60.3 -162.8 152.6 SSTKRLPS *** S SS EAAE

1dgwY 380 385 5.54 95.0 166.6 -73.2 -62.3 -82.4 143.3 59.4 24.3 LAGHKENV *** SSSSTTBS eAEa

1din_ 35 40 6.37 -80.0 -166.5 -79.7 2.8 -96.4 3.5 85.9 -167.4 AQEIFGVN *** E TTBS EAAe

1din_ 108 113 6.23 -104.5 7.5 -71.2 156.0 -60.5 -30.9 -85.9 0.1 ARHQPYSN *** HHTSTTEE AEAA

1din_ 207 212 5.42 -75.6 -0.4 -95.3 153.4 -72.3 -14.9 -83.1 -0.1 RTSSSGYV *** TTSTT AEAA

1dj0A 107 112 5.90 -146.8 125.2 -57.4 -29.2 -88.7 -60.2 -73.5 -19.1 DFHARFSA *** T TTTT EAAA

1dj0A 264 269 6.45 -84.5 151.7 49.1 -132.1 -72.9 -5.2 -61.4 132.3 MGPLFLAD *** STT EeAE

1dk0A 32 37 5.42 -134.8 159.9 -38.5 125.3 88.6 0.0 -140.9 16.6 NHTNGNVT *** TT EEaA

1dljA 143 148 5.30 -108.7 140.5 -60.7 145.3 83.4 -8.2 -141.9 33.5 FLRESKAL *** TTSTT EEaA

1dljA 318 323 5.66 -70.5 -18.9 -161.2 175.1 -56.9 136.1 65.3 12.9 LIMKSNSD *** SSTT S AEEa

1dmhA 158 163 4.96 -92.2 -167.7 -70.0 -9.2 -101.4 1.1 82.1 15.5 HANTKGFY *** E TTS EAAa

1dmhA 163 168 5.32 -105.2 151.3 -65.4 148.6 76.7 -7.7 -139.7 25.1 GFYSHFDP *** S TTS T EEaA

1dmhA 184 189 4.98 -84.7 -173.1 -50.2 -20.2 -112.8 16.8 67.5 24.3 ITDENGQY *** E TTSEE EAAa

1dmhA 295 300 5.28 -110.6 112.3 50.5 49.3 72.4 7.0 -134.6 153.4 RLVDGVDN *** B BTTB T EaaE

1dmmA 39 44 6.26 -141.2 109.3 -78.6 157.9 -59.6 132.7 118.6 -19.5 VEDPFGQP *** EESSTTS EEEa

1dp4A 411 416 5.93 -46.9 -42.3 -94.9 -1.2 94.1 -10.3 -139.2 153.3 YWPLGYPP *** TTSS AAaE

1dp7P 60 65 4.22 -76.4 165.6 -50.7 134.8 82.1 -5.4 -128.2 15.6 LGTRGNSK *** ESSTT E EEaA

1dqaA 501 506 6.19 -96.9 8.4 -102.3 143.4 -63.5 -29.0 -122.3 89.9 SKKLSEPS *** HTT SSTT AEAD

1dqaA 651 656 5.46 56.8 -136.9 -90.1 6.2 -90.1 154.9 -62.8 -35.0 RSGDAMGM *** EETTB H eAEA

1dqaA 826 831 6.41 -97.0 95.1 -59.0 -37.1 -69.6 -33.8 -149.3 86.9 GACKDNPG *** S SSSTT EAAD

1dqgA 91 96 5.99 -64.6 125.1 81.2 3.3 -134.9 141.1 -85.2 62.5 GIKGTELY *** EETTSS E EaED

1dqsA 97 102 4.76 -88.8 -1.8 -67.2 161.1 -123.8 102.0 -79.9 172.9 MLSQNPPC *** HHTSSS AEEE

1dqsA 358 363 4.79 -104.0 95.6 59.4 -136.1 -78.2 -2.5 -78.2 117.9 KNDGPKKK *** T BTTB E EeAE

1dqzA 214 219 6.41 -113.9 -168.0 -58.1 -22.1 -64.3 -20.4 106.4 -150.2 TPSDLGGD *** TT EAAe

1ds1A 148 153 6.24 -86.6 -5.6 -97.1 129.3 -55.9 -35.0 -96.5 23.6 MAYHRLQP *** TTTBTT AEAA

1ds1A 223 228 5.11 -53.8 123.2 -58.1 -28.5 -91.6 -6.9 -152.1 60.7 YGDADDPF *** ES TTS E EAAD

1dtdB 12 17 4.81 -119.0 169.2 -58.6 -38.1 -121.1 9.4 -168.2 168.7 cYQPDQVd *** EE SS EE EAAE

1dusA 112 117 4.89 -88.9 104.9 54.3 -122.9 -93.9 -2.3 -44.1 -39.3 HSDLYENV *** E STTTT EeAA

1duvG 128 133 4.46 -132.7 174.6 -57.9 -34.0 -95.3 -31.6 -141.1 150.7 GLTNEFHP *** S SS H EAAE

1duvG 305 310 5.65 -97.6 3.1 -75.3 163.7 -56.9 -25.6 -74.4 -18.4 VFESAASI *** HHTSTTB AEAA

1dvoA 144 149 5.46 -100.1 178.3 -61.7 -23.1 -88.5 0.8 89.3 8.4 RYDTEGYV *** EE TTS E EAAa

1dwkA 84 89 6.00 -56.5 -51.2 -146.6 68.6 72.1 -50.8 -114.1 125.9 CIDDRIPT *** SSSS ADaE

1dxrC 153 158 6.22 -59.8 -42.6 -86.9 115.9 -78.4 -2.1 -76.3 -13.0 LPLNNRET *** SSSSTTS AEAA

1dxrC 309 314 6.02 48.3 45.6 87.9 11.5 -126.0 141.9 -62.7 -28.6 CHQGVTKP *** HHTTSSSG aaEA

1dxrH 94 99 5.66 -77.7 -31.0 -39.5 134.3 -60.6 -32.9 -95.3 13.2 TDGFEGAP *** SSSSTT AEAA

1dxrH 187 192 5.86 -52.5 124.9 75.2 -1.5 -106.2 169.8 -115.0 67.6 SVAGSART *** EETTSS E EaED

1dxrH 203 208 4.69 -103.4 168.9 -58.7 -27.9 -129.2 -0.5 -169.7 161.9 DVKKDKIV *** EE SS EE EAAE

1dxrM 20 25 6.29 -93.8 164.2 -61.7 -24.3 -153.9 165.2 88.0 3.2 VSGEWGDN *** SS GG EAEa

1dy5A 20 25 5.79 -74.6 -27.7 -166.9 -179.2 -67.0 -12.5 -89.6 -1.4 AASSSNYa *** S STTHH AEAA

1dy5A 36 41 6.07 -68.3 -47.6 -113.3 -9.6 -169.9 164.4 -86.0 111.9 LTKDRbKP *** SSSSS S AAEE

1dy5A 90 95 5.25 -160.7 126.5 -49.5 133.4 -94.7 11.2 -100.8 84.2 SSKYPNbA *** T BTTB EEAD

1dypA 153 158 5.86 -111.9 -0.4 -70.5 152.8 -53.6 129.6 85.6 -8.6 SLTKEGDV *** T STT E AEEa

1dypA 195 200 6.10 -124.5 162.5 -56.6 -36.6 -71.9 -33.3 -88.5 -29.9 WXRPGSFP *** EE TTTSH EAAA

1e1hA 120 125 6.40 -98.4 6.8 -120.8 124.8 -73.4 -14.4 -84.4 -5.1 GSTIDTEL *** SSTTEE AEAA

1e1hB 324 329 5.40 -85.0 -179.4 -65.5 -20.7 -89.5 0.6 89.1 7.8 SEDTSGKF *** EE TTS E EAAa

1e1hB 377 382 5.76 -58.0 -50.0 -61.2 129.9 -65.4 -14.2 -75.4 -15.0 NIVPDENY *** SSTTT AEAA

1e1hB 383 388 4.78 -116.6 160.3 -58.2 -43.7 -69.2 -47.6 -119.2 3.5 NYTIKDGF *** T BTTTBT EAAA

1e25A 218 223 6.38 -93.5 14.8 -97.6 -112.0 -51.7 -38.5 -80.9 -9.1 GPERLKGL *** TTSTTTT AXAA

1e29A 12 17 5.62 -137.0 -157.2 -82.8 -22.0 -91.2 2.9 128.1 -17.5 PLDEAGGT *** ESSSSS E EAAa

1e29A 60 65 4.67 -91.7 -2.8 -70.8 164.9 -119.8 113.3 -68.3 169.7 LAGAEPRR *** HHTSSS AEEE

1e29A 81 86 5.22 -65.9 -34.2 -79.5 -1.0 75.1 9.4 -83.8 -36.8 KSYDGEDD *** B TTS SB AAaA

1e2wA 104 109 6.18 -53.7 -38.4 -158.1 159.2 -55.5 -25.6 -92.9 1.1 QPYSPEQK *** EESSTT T AEAA

1e30A 45 50 6.46 -72.0 151.3 -55.8 133.6 96.0 -6.5 -111.5 155.6 AVLPGFPF *** ES TTS S EEaE

1e30A 138 143 6.16 -99.7 0.0 -63.5 127.9 -46.7 128.3 89.9 -20.0 VCQIPGHA *** E STTTT AEEa

1e42A 797 802 6.45 -130.2 118.2 -70.1 161.6 -54.9 -33.5 -98.5 15.0 KMEPLNNL *** SSTTEE EEAA

1e42A 808 813 4.06 -138.1 -163.3 -77.6 -9.0 -92.5 -50.5 -166.4 -178.2 VKNNIDVF *** EE SS EE EAAE

1e4cP 23 28 5.79 -128.1 138.3 58.0 -124.1 -118.7 20.7 -73.6 -35.3 LNQGTAGN *** S TT E EeAA

1e4cP 58 63 5.54 -79.9 -178.5 -61.6 -25.7 -87.1 -5.6 89.3 11.7 FIDGNGKH *** EE TT B EAAa

1e58A 127 132 5.49 -79.8 -16.6 -57.6 139.9 -53.4 -28.6 -84.8 -6.8 TKDDERYP *** TTSTT G AEAA

1e58A 142 147 6.30 -80.1 167.9 -55.4 -43.1 -66.9 -29.1 -77.6 -32.2 KLSEKELP *** T TTTS EAAA

1e5kA 126 131 4.38 -96.1 9.3 106.4 -29.7 -117.0 -36.8 -148.2 158.0 VHDGERDH *** EE SS EE AaAE

1e5pA 22 27 4.37 -116.6 -2.9 -75.1 173.3 -54.0 133.1 94.3 -14.0 EKIEEGGP *** GGTSTT T AEEa

1e5pA 99 104 5.50 -76.4 179.6 -69.9 -12.4 -90.4 -10.5 97.7 4.4 NXDRAGQE *** EE TTS E EAAa

1e6uA 34 39 5.95 -72.5 169.6 -60.2 -37.0 -63.9 -31.8 -81.6 -30.3 LRTRDELN *** TTT EAAA

1e6uA 236 241 5.01 -127.8 154.7 -60.8 -35.6 -105.9 15.0 -147.7 67.1 NTQPMLSH *** TSBTTB EAAD

1e7lA 45 50 4.73 -129.1 147.9 92.5 176.6 -63.6 -20.7 -89.7 -12.2 ELNGPKAG *** SSTTTT EeAA

1eaqA 65 70 6.16 -74.2 -2.0 -106.3 131.7 -59.9 -16.1 -90.3 -7.3 RTDSPNFL *** E SSTTEE AEAA

1eaqA 140 145 5.30 82.4 175.8 -81.6 -14.6 -99.2 -10.0 -139.8 166.6 RSGRGKSF *** TT B eAAE

1eaqA 152 157 5.52 -86.0 52.8 -69.0 178.0 -113.7 99.4 -87.2 168.8 TVFTNPPQ *** EE SSS E DEEE

1eb6A 114 119 5.89 -92.4 -17.5 -101.4 145.7 -55.6 132.5 73.1 -1.3 LAQKcHAQ *** S STT AEEa

1eb6A 134 139 5.25 -67.6 -23.1 -76.3 -51.8 -159.0 129.3 -100.0 140.7 APGVYQPG *** TTTSSS AAEE

1ec7A 91 109 6.27 -80.0 360.0 360.0 -1.1 -95.5 -179.9 -131.7 -54.0 TFALRTTI *** HT SHH AxEA

1ec7A 429 434 5.55 -59.3 -29.4 -87.5 -46.2 -135.7 98.2 -48.3 124.7 MQYLIPGW *** HHTTSTT AAEE

1ed8A 16 21 4.70 -85.5 -7.7 -110.0 163.5 -57.6 123.4 74.0 -2.8 DITAPGGA *** TTSTTTT AEEa

1ed8A 71 76 6.25 -80.3 113.9 -62.4 -13.7 -106.1 -71.1 -48.6 -43.2 GFFKGIDA *** TTTT EAAA

1ed8A 122 127 5.09 -87.6 -172.7 -67.1 -18.9 -97.3 12.8 68.0 18.8 GVDIHEKD *** SB TT B EAAa

1ed8A 249 254 5.02 -162.7 -175.8 -56.8 -26.9 -84.0 -36.9 -133.4 71.2 EANQQKPL *** SSSS E EAAD

1ed8A 405 410 5.15 -90.3 -13.2 -72.3 174.7 -70.6 -16.1 -124.8 129.3 NSEEDSQE *** S SSSS AEAE

1edg_ 174 179 5.48 93.6 -26.7 -69.2 157.6 -63.1 -31.8 -104.0 37.3 LVGHANEW *** TTSTTTT aEAA

1eexA 515 520 6.12 -131.6 -179.9 -67.3 -15.3 -78.8 -38.6 -110.8 1.4 VLSAVNDV *** EE TTTSB EAAA

1eexB 132 137 5.66 -88.5 164.3 -58.5 -41.7 -68.3 -15.1 122.2 -14.5 GIQSKGTT *** EE TT E EAAa

1ef8A 43 48 6.49 -83.9 68.8 -129.5 153.6 -53.6 -34.4 -75.3 -13.0 DLNRPEIR *** HT STT DEAA

1ejdA 297 302 5.71 -61.2 147.7 -53.8 143.9 -80.2 16.6 -76.8 -176.2 TAPHPAFP *** STTS EEAE

1ejgA 40 45 6.28 -78.1 169.2 -64.1 -21.8 -94.4 0.3 -131.7 57.9 TaPGDYAN *** TTS EAAD

1el5A 40 45 5.47 -106.4 -9.1 -106.8 -134.5 -114.7 13.5 -116.6 7.7 HTNGSHHG *** SSSSS S AEAA

1el5A 258 263 4.40 -110.4 122.5 53.7 37.6 70.4 17.1 -72.2 -13.7 PSFGGCGL *** BSTT E EaaA

1el5A 275 280 5.69 -113.8 134.2 -60.0 -17.7 -95.6 -12.0 -120.9 3.7 KIDPDTIN *** E TTT EAAA

1eokA 172 177 4.93 -128.7 142.5 108.3 -178.0 -63.7 -18.8 -108.5 2.9 KYFGTTAP *** TTSSTTSS EeAA

1eokA 189 194 5.73 -82.3 3.1 -88.9 132.4 -50.8 -43.1 -70.2 -15.5 GIDVYAWN *** TTSTTHH AEAA

1eokA 260 265 5.17 -54.8 161.1 -65.8 -23.7 -79.4 -5.3 115.3 178.7 WTPTQGAK *** TT EAAe

1epxA 163 168 5.42 -135.7 128.8 57.7 31.3 81.9 3.8 -151.4 178.3 KIQNGTVS *** BTTB EaaE

1eqcA 22 27 5.58 -91.9 -60.9 -98.3 -9.6 -130.5 117.0 -73.6 135.8 GGWFVLEP *** TTSSS T AAEE

1eqcA 39 44 5.09 -109.2 98.9 62.1 -118.3 -100.1 0.5 -82.8 118.9 FQNGNDQS *** G BTTB T EeAE

1eqcA 385 390 5.43 -144.3 115.1 -60.5 162.0 -67.6 -11.9 -87.8 -9.7 FPQPVTDR *** S SSTT EEAA

1es9A 16 21 6.13 -107.0 10.6 -112.3 -50.3 128.5 -3.8 -95.2 -0.6 QDVQGDGK *** SSSS H AAaA

1esgA 37 42 6.15 -166.9 127.9 -68.9 176.4 -57.0 -16.9 -91.0 -5.0 PIWPATSK *** BSTT S EEAA

1esgA 86 91 5.05 -124.3 -88.7 -125.0 7.5 -90.8 -29.1 92.5 41.4 LEKKKGGP *** S SSS S AAAa

1esgA 100 105 4.43 -164.1 128.9 52.7 58.7 56.7 33.6 -165.5 153.4 FIENSELK *** EE SSSEE EaaE

1esgA 192 197 4.82 -95.9 146.0 104.5 155.1 -50.1 -20.6 -152.5 44.4 IPKGSDGM *** STTSS EeAD

1eswA 18 23 6.17 -72.6 163.9 75.3 29.0 82.0 -7.4 -163.4 150.4 PGPYGVGV *** SSSS EaaE

1eswA 52 57 6.32 -116.0 -151.8 -59.3 131.7 99.3 -21.9 63.4 16.8 PTGYGDSP *** B TT T EEaa

1eswA 248 253 4.53 -161.6 -178.6 -41.3 -37.7 -71.4 -47.9 -136.2 125.3 PPDYFSET *** SS SS EAAE

1eu1A 144 149 5.61 -159.0 -176.2 -91.0 -7.5 -86.0 -46.8 -139.9 36.6 SGDYSTAA *** ES SSSTT EAAA

1eu1A 194 199 6.07 -132.7 153.7 -70.8 -47.4 -88.0 -159.9 -79.0 121.9 EIGWVIPD *** T SSB EAEE

1eu1A 325 330 3.74 -96.3 49.2 -155.3 162.3 -54.6 133.9 59.7 36.2 IQRMHHGE *** GGSSTTTH DEEa

1eu1A 347 352 5.85 93.7 -8.6 -87.0 149.9 -55.1 118.8 75.5 5.7 QIGLPGGG *** TTSTT E aEEa

1eu1A 358 363 4.88 -101.8 -6.9 -122.3 123.9 48.8 38.7 59.1 44.4 SYHYSNGG *** TTSTTTT AEaa

1eu1A 593 598 4.47 -85.3 35.9 -104.6 166.6 -55.6 -39.0 -87.3 -18.6 PLGTPSGL *** SSTTSS AEAA

1eu1A 690 695 4.38 -152.0 -164.2 -74.8 -15.8 -90.2 -33.1 -171.3 179.0 VFNDRGQI *** EE SS EE EAAE

1eu1A 745 750 5.24 -174.6 -179.3 -71.4 -17.4 -68.3 -60.8 -103.5 -22.9 GTSKLAQG *** S TTT EAAA

1eur_ 60 65 3.85 -110.7 146.6 75.5 21.1 106.2 -9.0 -134.2 139.0 NGREGFPN *** TTSTT S EaaE

1eur_ 121 126 5.11 -79.3 -19.2 -101.0 146.0 -59.8 137.8 -71.0 116.6 GQTTAPIK *** B SSS B AEEE

1eur_ 137 142 5.19 -68.6 -21.0 -76.0 -44.9 -99.1 -20.4 89.8 7.7 VDRETGTI *** E TTT E AAAa

1eur_ 153 158 5.41 -99.1 -163.7 -61.0 -52.2 -65.1 -32.3 -92.9 -5.1 RQGFAGSR *** S TTT EAAA

1eur_ 213 218 4.75 -110.6 170.0 63.6 -167.1 -62.7 -20.6 -110.3 20.6 LRYGPHAG *** SSTTTT EeAA

1eur_ 228 233 5.47 -84.0 -176.4 -57.9 -37.3 -76.3 -13.5 98.3 10.7 IINAAGAF *** EE TT E EAAa

1eur_ 338 343 5.91 -93.2 -5.9 -111.7 -14.4 -158.2 166.3 -84.9 84.4 ASQTSRSQ *** SSSSEE AAED

1euwA 23 28 6.05 -61.9 -45.8 -141.1 165.8 -65.0 -16.2 -97.6 5.1 TYATSGSA *** SSTT AEAA

1evfA 84 89 5.27 -85.9 -169.6 -55.7 -39.2 -82.8 -6.4 89.6 14.4 WADENGDL *** T TTSB EAAa

1evfA 236 241 5.62 -69.4 -22.1 -158.3 170.1 -63.9 -15.8 -97.0 1.6 KPESIFDY *** SSTT AEAA

1evxA 45 50 5.33 -76.0 -17.8 -158.2 164.7 -52.2 141.4 -81.7 131.4 IPLAAPYG *** SSS AEEE

1ew4A 63 68 4.44 -123.3 177.4 -61.3 -33.3 -80.3 -35.9 -174.6 -156.7 LATKQGGY *** EE SS EE EAAE

1ewfA 42 47 5.01 -172.3 98.8 -20.7 178.4 146.6 -9.3 141.7 106.2 FKIKHLGK *** S SSSSS EExx

1ex0A 92 97 6.50 -133.3 113.0 -55.1 -18.2 -80.9 -20.2 -131.9 -3.3 PYDPRRDL *** TTT EAAA

1ex0A 104 109 6.34 -143.8 132.9 92.4 172.0 -77.8 -25.3 -121.8 62.3 YVIGRYPQ *** EE BSS EeAD

1ex0A 189 194 5.41 -83.2 -6.3 -82.2 141.5 -48.6 -31.0 -91.5 8.1 CEDDAVYL *** TTSTT AEAA

1ex0A 229 234 5.92 -97.3 -8.4 -65.2 159.4 -56.3 128.0 88.8 -15.3 GQFEDGIL *** TTSTTHH AEEa

1ex0A 268 273 4.05 -107.2 117.0 52.8 64.5 66.0 12.4 -113.4 -10.2 NAKDDEGV *** B STT SS EaaA

1ex0A 283 288 5.75 -44.4 -64.2 -104.5 45.6 -164.5 -170.6 -103.7 139.2 IYAYGVPP *** TTS G ADEE

1ex0A 401 406 4.42 -121.6 -161.9 -59.4 -21.9 -108.7 -21.0 145.7 -161.6 QENSDGMY *** EE SSSEE EAAe

1ex0A 558 563 5.37 -85.8 164.3 -60.8 -22.6 -69.7 1.5 86.5 -14.4 ITFYTGVP *** EE TTS E EAAa

1ex0A 710 715 4.23 -118.0 -171.2 -74.3 -12.6 -122.2 -82.3 -68.5 129.1 MSSDSLRH *** EE SS EAAE

1ey4A 45 50 5.61 -73.8 133.0 -41.3 -42.0 -121.7 -41.3 -82.4 -38.8 TKHPKKGV *** SS SSS S EAAA

1ey4A 76 81 6.34 -92.9 -167.6 -89.7 -4.4 -106.4 -145.1 -67.2 144.8 EFDKGQRT *** EE SS SB EAEE

1ey4A 82 87 5.20 -102.4 -172.7 -69.2 -13.4 -97.1 7.5 87.8 2.8 RTDKYGRG *** SB TTS E EAAa

1ezwA 68 73 6.17 -99.8 -41.6 -77.9 -8.9 -144.3 148.0 -62.3 141.7 NPYTRHPL *** SSSS HH AAEE

1ezwA 150 155 4.20 -119.6 -164.1 -84.4 -29.0 -103.9 -26.7 -139.5 135.7 YEGKYVKT *** B SSBB EAAE

1f0lA 23 28 6.01 -85.5 156.9 -56.7 138.3 101.4 -12.8 -126.4 -3.6 GTKPGYVD *** EE TT TT EEaA

1f0lA 38 43 6.26 -62.9 -31.3 -134.1 -173.0 87.8 -173.9 -81.8 -7.4 KPKSGTQG *** SSS TT AEeA

1f0lA 67 72 5.85 -35.9 115.3 -66.3 -16.3 -81.6 -18.6 -143.8 68.6 SVDNENPL *** B TTSTT EAAD

1f0lA 128 133 6.40 49.8 43.3 91.4 -10.1 -72.8 162.9 -78.0 -22.8 FGDGASRV *** HSTT SEE aaEA

1f0lA 436 441 5.49 -132.7 122.5 -35.0 101.0 77.4 -74.4 -41.0 -40.7 PTIPGKLD *** E BTTTEE EEdA

1f0lA 479 484 5.34 176.1 -167.4 -69.2 113.5 98.5 -6.7 -133.2 106.6 YVGNGVHA *** EEBTTB E eEaE

1f20A 1070 1075 5.84 -70.8 22.6 -84.0 106.2 -44.2 73.5 148.3 -27.0 RNTALGVI *** ESSSTT E AEXx

1f20A 1284 1289 6.37 -140.4 148.3 -59.6 -35.6 -91.1 -0.7 -114.4 -59.8 CRQSKIDH *** ES TTTS EAAA

1f46A 17 22 5.03 -62.9 142.1 -50.6 129.8 105.1 -13.0 -133.4 177.3 AAHHGSEL *** E TT E EEaE

1f46A 48 53 5.42 -47.2 -63.3 -82.3 169.6 -80.1 -21.8 -79.0 17.7 RHLSPDGS *** EESSTT AEAA

1f46A 63 68 4.31 -73.2 -20.3 -74.7 126.0 -63.8 145.8 -80.4 14.5 ANMVKPGT *** EESSTT AEEA

1f60A 93 98 6.41 -83.1 -7.1 -55.2 133.6 -67.7 -21.7 -77.5 -7.6 APGHRDFI *** TTHH AEAA

1f60A 192 197 4.93 -65.3 -28.0 -77.9 -37.4 -106.7 -1.9 86.0 -4.2 ISGWNGDN *** TTT BT AAAa

1f60A 215 220 4.64 -130.1 177.6 -68.6 -27.4 -95.2 -16.2 167.8 -158.9 KETKAGVV *** EE SSSEE EAAe

1f60A 326 331 5.29 -66.4 -18.5 -115.1 34.7 -168.4 87.6 46.6 67.2 GDAKNDPP *** EETTSS AADa

1f60A 411 416 6.03 -70.6 141.4 -61.8 -32.6 -67.3 -26.3 -92.2 -54.4 VEAFSEYP *** TTT G EAAA

1f60B 1129 1134 6.02 -81.9 -38.2 -154.4 -167.9 -76.0 -5.3 -105.7 -4.4 KPWDDETN *** EESSTTS AEAA

1f74A 155 160 5.75 -67.3 -26.4 -81.2 115.6 -63.9 -18.3 -84.7 -3.3 LYKNPKVL *** HHTSTTEE AEAA

1f7lA 68 73 5.72 173.0 -137.7 -60.1 -34.2 -92.1 -11.3 -157.2 126.1 GIGRQLSF *** SBTTB G eAAE

1f7lA 81 86 5.21 -84.9 -177.7 -63.2 -16.1 -95.4 -4.7 95.1 2.8 RKDQNGKP *** EE TTS E EAAa

1f86A 59 64 6.30 -124.3 159.3 -61.8 -33.8 -66.0 -43.2 -79.1 -25.4 LTTEEEFV *** S TTT EAAA

1f86A 98 103 5.83 -82.3 -6.8 -118.9 28.4 100.6 173.6 -63.2 136.8 ANDSGPRR *** ESTTS E AAeE

1f8eA 109 114 4.86 -64.2 -24.1 -147.5 44.1 -163.7 164.7 -81.8 70.5 IGEDSDVL *** HHTTS BE ADED

1f8eA 167 171 5.71 -100.6 165.0 -60.4 -35.6 -87.8 -12.1 -124.6 3.9 PPTVYNSR *** BTTT E EAAA

1f8eA 184 189 4.06 -97.6 4.5 108.2 -30.7 -99.7 -52.8 -153.7 178.7 dHDGKTRM *** EE SSSEE AaAE

1f8eA 196 201 4.16 -76.7 165.8 -52.2 -38.9 -97.2 11.1 -155.3 39.8 SGPNNNAS *** ES TTS E EAAA

1f8eA 245 250 5.11 -103.3 -2.1 -134.0 29.2 -176.6 -175.2 -57.2 140.3 GSATGPAE *** E SSS EE AAEE

1f8eA 336 341 5.87 -86.9 -33.7 -127.6 24.5 -156.8 158.8 -68.1 137.8 GKhNDPYP *** SSS AAEE

1f8eA 355 360 4.64 -145.4 63.2 60.7 -128.6 -71.5 -23.3 -84.3 46.8 YLDGVNTW *** E TTS E DeAD

1f8eA 367 372 5.47 -93.5 -8.4 -104.8 -32.7 -142.5 172.9 -80.8 70.5 ISIASRSG *** S SSSSEE AAED

1f8eA 429 434 5.98 -70.8 150.9 -86.3 -13.5 -70.0 -32.3 -121.6 83.5 RGRPKEDK *** EETTT TT EAAD

1f94A 56 61 6.22 -126.2 24.3 -129.4 162.3 -52.0 126.4 53.7 37.5 deTTNLeN *** EE STT AEEa

1f9yA 9 14 5.06 -139.3 18.3 -136.9 152.9 59.1 49.3 65.7 80.7 GSNLASPL *** EE SS HH AEaa

1f9yA 107 112 4.84 -130.7 174.4 -60.6 -39.0 -83.5 -27.6 -138.6 127.1 INTERLTV *** EE SS EE EAAE

1fc6A 156 161 5.63 -125.2 -78.1 -129.8 143.8 59.8 -16.1 -111.7 117.6 GTAGSVTG *** SSSS BB AEaE

1fc6A 420 425 5.22 -72.3 166.0 -50.0 -36.2 -72.0 3.7 76.9 19.5 YQTPAGVD *** EE TTS B EAAa

1fcjA 20 25 5.16 -105.6 -39.0 -79.0 114.6 87.1 -173.2 -89.0 13.2 LNRIGNGR *** SSSSSS AEeA

1fcyA 246 251 5.83 -89.0 -6.0 -78.3 127.4 -52.6 127.3 87.4 -12.9 AKRLPGFT *** HHTSTTGG AEEa

1fd3A 20 25 6.18 -73.7 172.9 -40.2 127.7 83.0 -11.6 -96.5 148.7 FcPRRYKQ *** TT EE EEaE

1fd3A 30 35 5.40 88.2 -61.9 -54.2 140.7 -55.0 134.2 84.1 -10.0 TbGLPGTK *** B SSTT E dEEa

1fdr_ 202 207 5.74 -144.4 167.2 -64.1 -18.5 -79.6 -30.9 -122.1 -15.3 PMNKETSH *** TTTEE EAAA

1fdr_ 234 239 4.82 -130.9 153.0 -58.8 -38.6 -84.6 -1.3 -166.4 84.4 KHLRRRPG *** B BTTB EAAD

1fi2A 48 53 5.28 -90.0 76.6 -133.3 164.6 -55.7 -41.3 -73.3 -24.6 NTSTPNGS *** STTSE DEAA

1fi2A 59 64 5.74 -87.7 -175.0 -60.3 -27.3 -67.0 -29.4 -101.5 -21.0 ELDVAEWP *** EESTTT G EAAA

1fi2A 67 72 6.40 -48.6 130.7 72.5 -7.3 -88.5 -6.9 75.1 20.1 GTNTLGVS *** GGTTSS E EaAa

1fiuA 24 29 5.48 -87.8 179.8 -72.8 4.8 -113.4 9.6 82.8 6.0 ATNNRGVV *** B TTS B EAAa

1fiuA 154 159 5.45 -49.1 -42.3 -103.2 -26.6 -148.1 155.1 -78.4 90.2 INRNEFLV *** HTSSS AAEE

1fj2A 23 28 5.79 -103.8 -174.2 -65.8 134.1 84.7 -57.8 -69.9 167.1 LHGLGDTG *** E SSS H EEaE

1fj2A 72 77 5.30 -125.1 42.4 -102.5 162.4 -63.6 -24.9 -79.8 -10.0 IGLSPDSQ *** STT DEAA

1fj2A 152 157 6.37 66.7 -164.3 72.9 -163.6 -69.8 -29.2 -106.5 25.8 PIGGANRD *** STTTT eeAA

1fjsL 126 131 5.75 -99.6 -18.8 -135.2 177.9 -68.7 -25.5 -126.3 62.8 IPTGPYPc *** EESSSS T AEAD

1fkmA 439 444 4.92 -116.2 2.3 -75.1 162.5 -46.7 126.4 93.1 -8.7 DNYIHGQP *** GGSSTT H AEEa

1fleI 38 43 6.08 -72.4 153.2 50.2 -116.7 -86.4 69.3 -62.0 103.6 DhPGIKKg *** GSSSS EE EeDE

1fn9A 26 31 5.06 -135.4 150.2 -59.0 -41.5 -65.7 -42.7 -114.4 2.8 IYSAQEGW *** EEBTTTBT EAAA

1fn9A 263 268 4.70 -66.4 -45.4 -134.2 140.5 61.2 24.9 55.1 30.9 TPILGKMP *** STT AEaa

1fnlA 33 38 4.82 -89.5 160.2 -51.8 -39.1 -94.9 2.9 -156.1 104.4 AYSPEDNS *** BTTB EAAE

1fnlA 72 77 5.34 -128.0 -177.2 -82.6 -2.8 -117.0 -23.9 -114.3 116.6 aQTNLSTL *** EE SSS EAAE

1fnlA 112 117 6.30 -57.1 147.0 -37.4 128.7 64.2 16.3 -115.6 154.6 HSWKNTAL *** EE TT EEaE

1fo8A 174 179 6.49 -75.7 165.7 -56.6 -22.2 -91.8 2.4 -124.2 26.0 AVQPDHRK *** TT GG EAAA

1fo8A 417 422 4.65 -141.0 118.3 50.8 40.5 76.3 -1.8 -95.6 124.7 AGYRGIVT *** T BTTBEE EaaE

1fp3A 32 37 5.73 -68.9 -22.1 -79.6 -37.6 -121.4 -8.2 91.2 158.1 HDREHGGF *** B TTTSSB AAAe

1fp3A 303 308 6.41 -84.9 -29.9 -70.2 130.5 -56.5 -41.6 -85.2 11.9 CPTQLEWA *** SSTTTT AEAA

1fp3A 352 357 6.35 -76.7 1.2 -80.0 -54.6 -109.9 -28.3 117.7 161.9 RDPEYGEW *** B TTTSSB AAAe

1fp3A 374 379 5.33 -172.3 -151.1 -59.7 -32.2 -124.2 11.5 -131.2 120.3 KGGPFKGC *** SS SS S EAAE

1fs7A 118 123 4.77 -110.9 -10.6 -161.5 -171.4 -78.4 0.2 -114.0 3.5 APVDGKTG *** SSTT S AEAA

1fs7A 196 201 5.87 -72.6 170.9 -57.3 -46.4 -65.6 -25.1 -89.5 -9.3 FKTFAEST *** TTTS EAAA

1fs7A 226 231 5.27 -77.3 179.4 -59.6 -26.5 -88.7 9.4 77.1 1.6 WKDDKGVD *** EE TT E EAAa

1fs7A 498 503 4.47 -164.7 -177.0 -78.9 -14.1 -112.9 4.6 -162.1 152.3 EKSSYYDK *** SB SS B EAAE

1fsgA 34 39 5.66 -69.9 158.5 -55.2 -36.7 -72.0 -16.8 -101.5 -23.7 LVPPHCKP *** TTTTT EAAA

1fsgA 206 211 5.39 -124.4 106.6 49.6 46.0 78.5 -1.6 -92.9 150.0 YDFNEMFR *** B BTTBST EaaE

1fsjB 26 31 3.88 -96.1 2.6 -98.3 155.8 7.2 -87.8 -109.4 36.0 DAGKDSGA *** GTTSTT E AExA

1fsjB 92 97 4.80 -138.4 112.9 51.4 -128.7 -75.7 -1.8 -100.5 106.5 QQVGGRKV *** G BTTB B EeAE

1ft5A 68 73 5.31 73.7 7.2 -117.1 156.2 -61.9 130.5 79.9 -3.8 GFGQKGGY *** STTSTT aEEa

1ft5A 74 79 5.68 -129.5 145.2 -65.7 -33.8 -68.3 -31.4 -144.5 77.6 GYTIESPK *** SSS EAAD

1ft5A 139 144 6.09 -97.3 81.1 -147.3 165.6 -63.1 130.0 72.3 13.8 HLNYEGSP *** HT STT S DEEa

1ft5A 175 180 5.22 -70.9 -8.6 -107.0 -15.7 -100.4 -87.2 178.9 157.5 EVKAMHEH *** TTTS AAAe

1fviA 239 244 4.57 52.7 78.8 40.9 60.6 82.8 -26.2 -94.1 113.0 VDYDGVVF *** E STT E aaaE

1fx2A 1035 1040 4.70 92.9 -39.0 161.9 -21.9 -65.3 -18.2 67.6 22.7 HDEVTKGY *** E TTSSSE axAa

1fxoA 55 60 4.96 -75.4 167.3 -56.3 -43.1 -71.5 -40.4 -85.1 -22.2 ISTPQDTP *** EE TTTHH EAAA

1fyeA 10 15 6.17 -75.9 152.8 -57.3 134.7 69.8 7.0 -114.5 176.8 STLPGKAW *** S TTS T EEaE

1fyeA 125 130 6.45 -73.0 -16.9 -82.3 176.4 -63.2 -34.1 -145.9 170.6 NLACPTIR *** HHTSSBST AEAE

1fyeA 198 203 4.20 -150.2 142.7 47.1 35.2 87.6 40.1 -160.3 125.7 QVSNGQAV *** EE SS EE EaaE

1g1tA 83 88 6.11 -99.8 67.2 -154.3 153.9 -53.6 137.9 64.2 25.5 NNRQKDED *** STT DEEa

1g1tA 96 101 5.60 -73.2 148.5 -53.8 -47.2 -120.7 160.3 51.9 38.1 IKREKDVG *** TT SSSTT EAEa

1g2bA 2 7 5.91 -153.6 -143.4 -71.3 135.5 76.1 -147.6 -77.8 160.4 DSGTGKEL *** SSS E EEeE

1g2bA 17 22 5.22 -106.2 -10.7 -153.4 156.7 -54.8 -31.3 -73.1 -8.3 QEKSPREV *** SSTTB AEAA

1g2rA 28 33 5.52 -90.9 178.7 -71.0 -18.4 -85.3 1.8 81.3 15.6 VKNKEGQV *** EE TTS E EAAa

1g3mA 182 187 4.94 -71.5 -19.3 -75.2 151.8 -71.9 -28.3 -84.7 3.3 KGKSPRVL *** HTTSTTEE AEAA

1g3mA 235 240 5.88 -76.7 -27.7 -114.4 -7.4 -100.8 11.9 57.2 11.1 NPSTNYTT *** TTTS TT AAAa

1g3mA 242 247 6.26 -66.1 154.8 -57.2 -26.5 -70.3 -20.1 -98.4 -53.2 TLPDEIMN *** TS TTTB EAAA

1g3p_ 47 52 5.60 -58.4 -40.4 -76.5 -1.2 58.4 21.6 -89.0 -19.5 bTGDETQb *** E TTSSEE AAaA

1g5aA 138 143 6.10 -57.0 146.3 -77.6 143.9 87.1 -75.8 -122.1 114.8 KCPEGKSD *** SS ST EEdE

1g5aA 294 299 5.67 -51.4 138.0 88.9 -5.9 -119.2 172.5 -75.0 -8.2 KQMGTSCE *** TTS SS EaEA

1g5aA 439 444 5.37 -57.1 -31.4 -78.7 -19.9 -119.8 -21.9 85.7 -2.9 YNPSTGDC *** TTT AAAa

1g5aA 500 505 5.63 -91.0 113.1 -55.8 -20.2 -90.8 -9.3 -125.5 -23.1 SQDSNKSD *** GG TTTTT EAAA

1g5aA 602 607 5.62 -69.7 -22.2 -74.8 -55.6 -90.6 -19.0 85.0 1.1 HDLIGGKT *** EETTT E AAAa

1g5hA 253 258 5.29 -101.4 177.8 -41.5 -42.6 -92.7 11.9 81.8 7.8 CQDELGRK *** EE TTS E EAAa

1g5hA 348 353 5.30 -53.5 139.4 -47.8 -34.7 -74.4 -21.1 -101.8 -30.9 KLHPCLAP *** TTT S EAAA

1g61A 2008 2013 4.80 -101.0 103.4 47.4 46.6 87.4 -7.0 -96.8 124.5 KYFSGIPT *** E BTTB EaaE

1g61A 2052 2057 5.17 -132.3 121.5 66.4 22.0 89.7 -4.3 -107.0 151.1 TNIGGSSL *** BTTBS EaaE

1g61A 2143 2148 4.56 -129.9 117.7 54.9 39.7 75.4 -0.2 -98.4 133.6 GTIAELPT *** BTTBS EaaE

1g61A 2188 2193 5.33 -139.5 168.7 52.8 43.8 61.2 54.7 106.5 -1.9 GTANKGTT *** BTTTBS Eaaa

1g61A 2202 2207 4.05 -136.7 -168.1 -70.6 -12.2 -102.0 -25.4 -176.8 177.5 IANSKGAV *** EE SS EE EAAE

1g66A 15 20 5.48 -65.5 158.4 -51.0 140.6 122.5 -146.2 -91.5 -19.2 TASPGYGS *** T SS GG EEeA

1g6sA 67 72 5.19 -52.4 -40.4 -92.3 7.4 61.9 19.9 -103.6 -6.3 LSADRTRC *** E TTS E AAaA

1g6sA 395 400 5.31 -87.4 42.1 -141.1 -166.3 -84.8 -2.4 -114.7 141.1 VALSDTPV *** GGSSSS E DEAE

1g8kA 90 95 5.61 -77.4 125.0 -61.8 -24.8 -64.2 -54.7 -111.1 -13.0 KACVVNSG *** TT TTTTT EAAA

1g8kA 126 131 4.33 -80.7 72.2 67.1 -44.3 -146.9 17.6 -170.9 148.4 LYAADEWV *** EE SSSEE DaAE

1g8kA 388 393 5.29 -104.0 30.3 -92.1 -163.0 -76.7 89.2 75.4 77.3 IIWGNDNY *** HHTSTTHH AEDa

1g8kA 533 538 5.98 108.5 -36.0 -90.8 -10.4 -154.6 161.8 -79.7 152.4 HPGEMNLT *** BTTB EE aAEE

1g8kA 541 546 4.74 -100.6 -165.0 -67.7 -2.8 -122.9 25.6 64.2 23.4 SMNGERRI *** EE TT EE EAAa

1g8kA 661 666 6.08 -67.7 -14.8 -111.2 6.3 76.4 17.7 -95.6 101.0 LYTEGKFD *** S TTS S AAaE

1g8kA 666 671 4.57 -87.7 68.1 -142.9 170.5 -45.7 -52.0 -100.1 2.5 KFDTDDGK *** SSTTSS DEAA

1g8kA 749 754 4.21 -159.4 -162.4 -66.8 -25.6 -83.0 -25.7 175.8 -177.2 VYNDFGST *** EE SS EE EAAe

1g8kA 775 780 5.55 -84.0 169.5 -84.5 -8.2 -142.7 92.0 100.6 88.2 FGYVNGIQ *** S SSS G EAEa

1g8mA 111 116 6.11 -85.8 6.7 -104.4 157.3 -40.0 85.8 70.7 35.9 TVSSPGVT *** HHTSTT AEXa

1g8mA 160 165 5.68 -90.5 5.8 -90.4 137.2 -58.1 -28.4 -111.3 12.9 XAASKDKD *** HHTSTTSS AEAA

1g8mA 210 215 5.17 -61.1 -33.0 -140.8 156.5 -46.8 -38.1 -77.2 -3.4 YGXNPHQS *** SSSTTS AEAA

1g8mA 222 227 6.02 -86.7 0.5 -88.1 166.4 -55.3 -33.1 -150.3 147.1 YTTRPKLP *** E SSSS S AEAE

1g8mA 404 409 6.05 -91.3 -6.0 -87.8 167.1 -44.7 -65.7 -116.2 108.1 NIVTKNKT *** B SSS AEAE

1g9gA 90 95 6.14 -77.0 -179.6 -58.6 -28.4 -79.5 -27.0 -87.0 -23.8 IPTEKDQP *** S TTTS EAAA

1g9gA 160 165 5.81 -67.1 -32.5 -79.6 -22.6 89.9 8.3 -76.4 -24.8 DNWYGFGA *** T SSS S AAaA

1g9gA 165 170 3.68 -107.4 125.1 48.9 49.1 67.5 16.8 -84.9 -8.0 FGARADGT *** STT SS EaaA

1g9gA 305 310 5.42 -97.4 -39.8 -112.3 8.4 -149.3 147.1 -92.0 111.2 GGIDSTWS *** EESSS E AAEE

1g9gA 340 345 5.25 -68.7 -24.3 -63.8 156.5 -59.5 -43.5 -106.3 7.3 KPKSSNGA *** SSSSHH AEAA

1g9gA 564 569 5.44 -81.2 163.4 -49.3 -35.0 -83.8 1.6 101.9 -10.9 GKMPNGDV *** EE TT E EAAa

1ga6A 175 180 5.14 -88.3 -2.6 -94.8 75.2 53.9 41.2 57.6 61.3 YEaNNRGY *** BTTTTTT ADaa

1ga6A 224 229 5.00 -82.7 -176.6 -71.0 -14.4 -83.3 -13.1 95.6 7.8 GLDSNGKL *** EE TTS E EAAa

1ga6A 335 340 3.82 -156.5 148.5 69.3 22.2 94.7 -5.1 -97.3 170.5 NGYGGYGY *** SSSTT S EaaE

1ga8A 120 125 6.43 56.8 -133.6 -99.7 18.6 -72.9 154.5 -71.5 -35.4 DLGDNWLG *** TT SEE eAEA

1ga8A 245 250 5.96 -62.7 151.4 102.9 175.5 -65.4 -21.8 -86.3 103.9 HYCGPAKP *** E SS T EeAE

1ga8A 251 256 5.67 -89.9 1.6 -99.5 179.6 -79.0 155.6 38.4 49.2 KPWHRDCT *** TTSTT AEEa

1gai_ 21 26 5.29 -121.3 129.2 -73.8 166.8 -105.8 16.2 -117.9 3.1 NNIGADGA *** HTBTTT T EEAA

1gai_ 343 348 5.91 -118.5 175.3 -59.9 -33.4 -68.3 -42.0 -97.3 0.6 EITDVSLD *** EE TTTHH EAAA

1gai_ 392 397 5.97 -67.6 159.3 -56.3 -29.6 -94.1 7.9 95.7 -3.9 HAASNGSL *** HS TT EAAa

1garA 110 115 6.10 -95.8 -107.8 -113.2 21.6 157.1 -38.4 -117.6 138.8 PSLLPKYP *** S SSS XAxE

1garA 203 208 5.87 -55.0 160.9 -47.2 -20.4 -109.2 16.3 94.7 -148.0 RLPPQGYA *** E TT EAAe

1gbs_ 80 85 5.36 -117.6 100.4 56.4 42.0 83.5 -9.7 -92.9 142.0 VLKNGWGD *** T BTTB T EaaE

1gbs_ 85 90 4.94 -67.8 -10.4 -107.5 8.7 70.6 7.1 -95.0 -43.1 WGDRGNGF *** B TTS EE AAaA

1gbs_ 98 103 5.98 -78.8 -18.0 -107.3 -32.2 -144.9 139.7 -71.8 120.8 DKRSHKPQ *** ETTTS AAEE

1gbs_ 155 160 6.34 -158.5 170.7 -79.4 -47.2 -63.4 -39.2 -107.9 16.3 VRSYARMD *** STTTT EAAA

1gca_ 196 201 4.53 -108.4 3.3 -107.9 -176.2 -63.4 -20.8 -92.0 1.6 WLSGPNAN *** HHTSTTGG AEAA

1gca_ 279 284 6.14 -52.9 126.6 74.5 16.6 -126.4 171.0 -98.0 5.9 AADGTSWK *** TTTTS EaEA

1gca_ 298 303 6.07 -143.8 -162.3 -65.3 -25.1 -77.1 -29.3 -117.5 24.3 GVDKDNLS *** EE TTTGG EAAA

1ggxA 86 91 6.10 -81.7 160.4 -61.8 -28.4 -51.5 -52.9 102.3 -164.4 LSFPEGFK *** HTTTT EE EAAe

1ggxA 205 210 5.81 -57.8 -39.9 -102.9 11.7 58.3 43.4 -101.0 -12.3 HNEDYTIV *** E TTSSEE AAaA

1gk9B 6 11 5.87 -93.5 -177.6 -50.7 -39.6 -77.1 -9.3 -117.6 -10.6 VIGKSKAQ *** EE TTTBS EAAA

1gk9B 11 16 5.94 -111.2 127.6 67.4 23.3 -167.6 166.0 -91.6 -29.3 KAQDAKAI *** TBSSSSEE EaEA

1gk9B 108 113 5.51 -62.9 117.4 66.8 31.8 -152.2 177.8 -83.3 135.9 TVKNGQAE *** TTS E EaEE

1gk9B 210 215 6.27 -99.2 12.8 -80.3 -4.6 130.4 -36.6 -65.1 -27.0 VPGTGKWD *** EESSSTTS AAaA

1gk9B 333 338 5.65 -71.5 -18.9 -65.6 -23.3 73.6 21.4 -134.2 -32.8 LNDDGKTW *** B TTSSEE AAaA

1gk9B 378 383 4.44 -142.9 174.0 -74.9 -14.1 -87.0 5.1 87.0 173.5 ETTQDGPT *** TT S EAAe

1gk9B 414 419 6.33 53.8 44.7 84.3 -9.9 -84.7 152.6 -64.5 139.0 LFAGKPQQ *** TTTTS HH aaEE

1gkmA 107 112 5.99 -75.0 -4.1 -74.1 -12.6 90.4 14.4 -102.5 7.4 SLSRGFSG *** HHTTS S AAaA

1gkmA 139 145 5.11 -105.6 8.9 67.2 360.0 360.0 -1.6 81.9 -135.1 GSVGSGDL *** BSH Aaxe

1gkmA 504 509 5.95 -95.4 -50.4 -91.4 145.2 -48.1 -45.9 -99.2 6.6 AGVLPSLC *** TTSSTT AEAA

1gmxA 65 70 6.35 -63.3 -46.4 -142.3 -2.4 100.7 8.9 -97.0 -60.7 MCYHGNSS *** E SSSSHH AAaA

1gnlA 7 12 5.35 -80.3 -13.8 -119.2 168.7 -63.4 -28.1 -88.4 8.1 CYQCQETV *** B STT G AEAA

1gnlA 72 77 5.67 -99.7 4.2 -71.5 150.1 -51.1 130.7 66.0 24.4 FATITNAN *** HHTSTTTB AEEa

1gnyA 107 112 5.70 -108.9 -3.7 -123.9 -148.1 -96.1 10.1 -120.4 129.7 NASGSTSH *** EE SSS AEAE

1gnyA 127 132 5.97 -71.1 -30.0 -171.6 177.6 -56.7 131.5 83.9 2.9 AALGDGVG *** E STTEE AEEa

1gp1A 18 23 5.21 -66.9 142.4 -50.4 -46.0 -57.4 -13.6 130.8 117.4 ARPLAGGE *** E BTTS S EAAx

1gp1A 43 48 5.88 -123.7 48.3 -93.4 162.5 -69.2 -26.2 -102.3 11.3 ASLXGTTV *** SSSTTHH DEAA

1gp1A 75 80 5.62 -79.1 -14.8 -115.7 86.4 73.4 20.5 46.1 49.5 CNQFGHQE *** TTTT ADaa

1gp1A 131 136 5.26 -67.3 134.1 -47.0 -31.8 -104.9 -3.5 -159.2 82.9 PTPSDDAT *** S TT SS EAAD

1gp6A 130 135 4.94 -132.8 28.4 -162.4 171.0 -54.6 -27.3 -62.7 -34.9 LANNASGQ *** STT AEAA

1gp6A 321 326 5.52 -159.4 177.2 -58.2 -44.1 -79.5 -15.9 -129.2 72.1 MVSVESPA *** G SSS EAAD

1gppA 445 450 5.47 -74.5 172.1 -64.7 -22.7 -88.4 11.0 74.0 3.9 FLLANQVV *** EEBTT BE EAAa

1gpqA 57 62 5.00 -62.1 119.7 -50.9 128.7 63.7 12.9 -150.9 47.2 AaKPHDaG *** EE TT TT EEaD

1gpqA 72 77 5.44 -68.1 -38.4 -47.3 -45.9 -106.5 -8.4 78.2 -22.5 WSEKSNQM *** E TTT E AAAa

1gpr_ 64 69 5.66 -58.0 -36.6 -75.3 -6.9 63.4 18.0 -98.8 -9.3 VFPTKHAI *** E TTSSEE AAaA

1gpuA 106 111 5.39 -56.4 -33.8 -71.8 133.3 -62.8 132.6 99.6 -17.6 EFELPGVE *** TTSTT AEEa

1gpuA 115 120 6.38 138.2 -49.4 -78.8 114.1 -53.9 142.5 91.8 -20.7 TTGPLGQG *** STTHH aEEa

1gpuA 140 145 5.16 -139.2 162.5 -54.6 128.0 79.6 -2.2 -133.7 83.0 YNKPGFTL *** H BTTB EEaD

1gpuA 252 257 5.11 108.0 -34.1 -71.8 154.9 -64.1 -25.1 -108.7 27.0 GYGSLHAG *** TTTSTTTT aEAA

1gpuA 400 405 6.36 -72.7 -9.4 -65.4 -49.3 94.1 3.3 -112.0 -1.3 PPSSGSGN *** TTTSSEE AAaA

1gq8A 64 69 6.33 153.8 133.5 -55.9 -32.0 -81.2 -24.5 -113.2 -6.2 GDGRTSTI *** ES TTTEE eAAA

1gq8A 132 137 6.29 -172.5 -160.5 -118.9 -97.3 -79.2 129.3 65.8 40.2 ILAYQDSL *** EE STT E EXEa

1gq8A 208 213 5.56 -77.1 172.0 -64.1 -28.4 -85.0 -8.2 -101.6 -40.3 GATSDLQP *** EE TTTGG EAAA

1gq8A 226 231 5.45 -59.5 -42.7 -123.0 162.3 -49.3 -35.0 -116.3 35.2 RPWKEYSR *** SSTT E AEAA

1gqiA 76 81 5.26 -111.7 163.4 -56.6 -27.9 -75.9 -27.3 -117.6 -2.2 IGTPDNSP *** EE TTT H EAAA

1gqiA 96 101 6.27 70.2 -163.8 -76.7 -11.3 -130.5 -2.3 -85.0 174.5 ALGAEGYL *** TTTTT EE eAAE

1gqiA 418 423 5.65 -131.2 52.4 81.7 -170.2 -60.6 146.6 94.0 1.5 HARGEGST *** TTSTT B DeEa

1gqiA 523 528 5.13 -55.5 132.3 -105.7 140.1 -99.3 9.9 -72.4 -32.8 LYSQGDHY *** B TTT S EEAA

1gqzA 18 23 5.78 -97.3 -46.8 -95.9 -7.1 -123.2 152.2 -99.7 108.0 DGVTQNVF *** EESSS AAEE

1gqzA 51 56 6.03 47.8 47.9 -172.1 114.3 -65.9 -11.4 -94.7 -0.2 APYDPELD *** SSTT S aEAA

1gqzA 98 103 4.37 -131.4 178.5 -56.3 -21.2 -122.4 -8.1 162.2 156.7 VSTQKGNM *** EE SS EE EAAe

1gqzA 142 147 4.26 -120.7 167.3 -51.2 -29.8 -119.4 -9.9 -170.6 144.6 LRTDIQTV *** EE SS EE EAAE

1gsa_ 61 66 5.68 -140.9 123.8 -82.5 -1.1 -98.3 -39.4 -148.1 102.0 KQNYEEWF *** S SS E EAAE

1gsa_ 227 232 6.16 -126.9 126.4 71.1 -118.5 -96.6 58.0 -136.7 127.8 IPQGGETR *** E SS S EeDE

1gsjA 68 73 6.25 -57.2 158.0 -61.9 -29.3 -80.7 0.7 -121.4 -11.5 VTPADQID *** B TTTHH EAAA

1gsjA 150 155 5.37 -81.2 164.0 -60.0 -21.7 -87.3 4.4 84.6 23.2 GVTDEGQL *** EE TTS E EAAa

1gsjA 186 191 4.90 -88.1 -173.6 -58.2 -17.5 -102.0 5.5 80.7 13.8 ILDGKGQR *** B TTS B EAAa

1gtkA 265 270 5.60 -52.0 -28.9 -79.9 3.9 79.5 3.3 -67.0 -26.7 GAPDGSQI *** E TTSS E AAaA

1gtkA 304 309 5.12 -80.3 -7.9 -101.0 -53.6 -67.0 -17.9 56.2 90.0 LAEVYNGD *** HHTTTTS AAAa

1gu7A 186 191 5.35 -72.6 122.3 -51.4 134.0 77.2 -18.6 -110.4 -53.3 KLTPGKDW *** TTT E EEaA

1guiA 6 11 6.46 -70.0 -16.5 -93.1 -13.9 62.0 35.0 -78.0 -16.6 NNGTFDEP *** SSTT SS AAaA

1guiA 16 21 6.43 -57.3 -38.7 -66.5 -41.5 -112.1 -14.7 -143.6 57.9 NDQANNPD *** TTT TT AAAD

1guiA 97 102 4.75 -94.3 18.8 -157.4 163.6 -55.6 134.2 -93.3 23.1 LQNHDPWT *** E SSTT AEEA

1guqA 4 9 6.50 -145.4 96.3 -44.2 -29.6 -68.0 -30.2 -127.4 -18.7 QFNPVDHP *** TTTS EAAA

1guqA 53 58 6.16 -103.5 -10.8 -70.7 154.1 -63.1 142.7 65.0 11.7 CFLCAGNV *** TTSTT B AEEa

1guqA 227 232 5.94 -98.0 -58.5 -74.8 149.4 -37.2 -65.3 -108.9 33.6 WAAWPFET *** T STT E AEAA

1gv9A 83 88 4.15 -136.9 -178.3 -62.3 -37.4 -111.1 -7.7 -170.6 172.8 IPSADQIR *** EE SS EE EAAE

1gv9A 122 127 6.35 -85.8 152.5 64.6 -167.7 -95.0 4.8 -130.6 146.0 TGRGRIGA *** E SSS EeAE

1gv9A 161 171 5.16 -67.9 118.9 -125.3 12.9 51.2 360.0 360.0 11.2 DSFDNNPA *** E E EAax

1gveA 61 66 6.17 72.8 -0.9 -94.8 140.8 -58.5 115.9 122.0 -7.6 GLGRSGCK *** TTSTT aEEa

1gveA 76 81 3.90 -147.2 -179.8 55.5 32.4 79.8 4.5 -103.4 159.6 APMFGKTL *** S TT S EaaE

1gveA 168 173 5.74 -154.6 179.9 -57.5 -25.2 -118.5 21.3 -143.2 90.4 MYNAITRQ *** E BTTB G EAAE

1gweA 57 62 5.45 -59.2 145.5 -125.8 116.0 -78.6 -164.5 73.8 32.8 PERRPHAK *** SS S EEEa

1gweA 208 213 5.57 -89.6 178.5 -62.5 -29.0 -86.5 -3.2 93.5 -0.1 WVNAQGEK *** EE TT E EAAa

1gwmA 42 47 6.42 -47.1 131.1 83.1 -5.7 -98.1 -11.4 47.3 49.7 PQEGKYGA *** E TTS E EaAa

1gwuA 7 12 5.87 -60.9 -22.1 -104.5 -42.1 -77.7 -27.0 -160.7 69.4 FYDNSaPN *** TTTTT TT AAAD

1gwuA 57 62 4.93 -137.6 168.3 -72.1 -13.6 -103.4 -16.7 -148.3 142.8 DNTTSFRT *** SSS EAAE

1gwuA 290 295 6.01 -139.9 176.9 67.7 -112.9 -110.0 7.3 -76.3 141.5 PLTGTQGQ *** B TTSSB EeAE

1gx5A 267 272 5.47 -83.8 177.5 -64.9 -11.4 -97.2 -3.9 91.0 0.7 LTNSKGQN *** EE TTS E EAAa

1gx5A 282 287 5.96 -102.1 28.0 -103.8 163.2 -60.3 -34.9 -84.5 5.6 ASGVLTTS *** TTSTTHH AEAA

1gx5A 364 369 4.95 -144.4 137.8 62.0 38.5 53.6 24.1 -137.7 167.2 ITSCSSNV *** BTTBEE EaaE

1gx5A 374 379 5.19 -88.2 -174.3 -49.7 -47.6 -77.7 3.0 85.0 2.0 AHDASGKR *** EE TTS E EAAa

1gxmA 522 527 5.53 -123.9 -110.2 -68.9 173.3 -56.4 -35.6 -98.6 12.0 KKARAYEL *** STT XEAA

1gxmA 563 568 6.13 -91.7 13.3 -96.9 139.4 -70.2 -10.4 -82.1 -5.0 WFNSPRTY *** HHT TTTS AEAA

1gxmA 579 584 4.62 -66.0 -19.2 -49.6 -40.2 -117.5 -49.8 -163.0 111.2 SLAATNPI *** GGTTT E AAAE

1gxmA 608 613 5.31 -162.8 -178.0 -66.8 -19.1 -82.5 -0.9 74.6 12.8 FSDRDGSK *** EE TT E EAAa

1gxuA 17 22 5.72 -147.2 140.7 76.6 18.4 -131.7 5.9 81.5 23.9 KVQGVGFR *** E SSSSHH EaAa

1gxuA 41 46 4.47 -106.9 18.1 93.9 -13.0 -125.2 51.9 87.9 -160.9 CNDGDGVE *** EE SSSEE AaDe

1gybA 74 79 5.42 -166.7 -179.7 -80.4 -1.3 -103.3 32.1 94.2 -18.1 PASPYGDV *** ESSTTS E EAAa

1h03P 23 28 6.21 -121.1 21.2 -83.4 169.8 -49.0 135.8 90.5 -12.6 GGILFGAT *** T SBTT E AEEa

1h03P 97 102 6.04 -74.6 171.6 -51.0 123.9 88.4 2.2 -115.9 150.7 AdNKGFTM *** EE TT EE EEaE

1h05A 17 28 4.46 -120.8 -22.1 -39.9 360.0 360.0 5.1 -73.8 131.8 LGRRGTTH *** TTT H AAxE

1h0aA 112 117 5.43 -76.4 179.7 -68.4 -23.1 -80.8 10.4 70.5 9.8 YVDRDGKD *** B TTS B EAAa

1h0hB 44 49 6.24 -141.2 175.8 -50.9 -27.7 -107.2 7.5 -136.4 101.8 DFNFHTYK *** S BTTBS EAAE

1h0hB 95 100 5.08 -60.4 -41.9 -67.4 -33.0 -112.4 -22.5 87.2 2.2 HDDATGCV *** E TTT E AAAa

1h0hB 156 161 6.11 -79.9 -24.8 -102.8 4.8 77.8 14.0 -64.7 -41.3 SCPTGAMN *** H SSS EE AAaA

1h16A 101 106 6.37 -87.2 -1.7 -141.6 144.8 -55.6 131.5 64.9 3.3 QTEAPLKR *** SSSSTTEE AEEa

1h16A 169 174 5.47 -102.6 0.7 -145.6 20.9 -164.8 162.5 -63.1 138.6 LPDAYGRG *** TTSSB AAEE

1h16A 548 553 5.04 -90.1 -177.3 -59.0 -2.6 -113.5 0.8 89.5 3.1 IRDEDGLA *** EE TTS E EAAa

1h16A 618 623 5.87 -113.7 159.5 -55.6 -22.5 -75.9 -12.6 108.3 -9.4 GNTPDGRR *** TTS EAAa

1h16A 630 635 6.20 -74.7 141.4 49.0 -124.4 -95.3 13.6 -67.7 151.2 FGPGANPM *** STT S EeAE

1h16A 635 640 6.11 -64.0 151.7 -40.6 118.6 71.5 1.5 -80.9 -24.3 NPMHGRDQ *** STT EEaA

1h16A 695 700 5.15 -149.0 179.1 -51.6 -40.5 -96.2 -0.4 -132.6 113.5 HHEASIEG *** BTTB EAAE

1h16A 731 736 4.13 -131.1 -10.7 -175.6 -0.1 135.6 9.3 -158.3 175.3 IRVSGYAV *** EE SSSEE AAaE

1h1nA 286 291 5.21 -75.4 119.3 -60.7 152.6 -98.6 -5.7 -119.8 6.6 SMEPDNGI *** TTT H EEAA

1h2cA 103 108 5.99 -72.7 -26.0 -112.3 -47.8 -127.8 138.9 -67.8 157.1 DQKTYSFD *** SSSS HH AAEE

1h2cA 126 131 5.22 -77.2 148.4 -61.4 -19.0 -86.5 -12.6 -155.9 63.2 FGKATNPL *** ES TTS E EAAD

1h4gA 31 36 4.83 -60.0 147.5 57.6 -130.2 -86.6 4.8 -89.0 157.4 LNHGGTFS *** E STT EE EeAE

1h4rA 27 32 4.47 -100.2 174.6 -69.5 -24.9 -78.1 -47.9 -169.5 -176.9 IVTMDAEM *** EE SS EE EAAE

1h4rA 87 92 6.45 -95.4 0.8 -63.1 110.3 -63.4 137.0 -82.0 136.5 VSKEEPVT *** SSSEE AEEE

1h4rA 212 217 5.70 -87.6 -7.6 -67.5 139.9 -55.4 -34.9 -90.4 -5.5 AQDLEMYG *** HTTSTTTT AEAA

1h4rA 244 249 6.25 -71.7 139.2 -56.4 -28.7 -85.8 2.2 -141.2 92.1 IYDPENRL *** EE TT SS EAAE

1h4rA 275 280 6.39 -74.1 -26.7 -84.5 100.8 -54.2 -19.3 -70.7 -21.3 KPLDKKID *** EESSTTS AEAA

1h65A 217 222 5.08 -89.9 -175.5 -67.0 -13.3 -97.0 13.9 68.2 8.1 NKNDSDEK *** B TTS B EAAa

1h6lA 61 66 6.31 -80.2 123.9 -68.4 -24.0 -110.2 -27.6 -143.9 89.0 WLDPKNPQ *** E SS GG EAAD

1h6lA 75 80 6.50 -57.7 -16.5 -100.1 -5.7 -146.6 -158.8 -88.5 -171.1 TNKKSGLA *** EETTS E AAEE

1h6lA 122 127 4.71 -65.2 158.0 -49.1 104.5 79.6 16.9 -99.1 -77.6 NRSEGKNT *** E TTT E EEaA

1h6lA 145 150 6.37 -70.7 118.2 -65.6 -19.8 -94.2 -23.0 -139.2 81.8 ITDPNRPI *** S SSS E EAAD
[truncated: 228,049 more chars]
